# Inverse hydride shuttle catalysis enables the stereoselective one-step synthesis of complex frameworks

**DOI:** 10.1038/s41557-022-00991-4

**Published:** 2022-10-20

**Authors:** Immo Klose, Giovanni Di Mauro, Dainis Kaldre, Nuno Maulide

**Affiliations:** Institute of Organic Chemistry, University of Vienna, Vienna, Austria

**Keywords:** Homogeneous catalysis, Synthetic chemistry methodology, Synthetic chemistry methodology

## Abstract

The rapid assembly of complex scaffolds in a single step from simple precursors identifies as an ideal reaction in terms of efficiency and sustainability. Indeed, the direct single-step synthesis of complex alkaloid frameworks remains an unresolved problem at the heart of organic chemistry in spite of the tremendous progress of the discipline. Herein, we present a broad strategy in which dynamically assembled ternary complexes are converted into valuable azabicyclic scaffolds based on the concept of inverse hydride shuttle catalysis. The ternary complexes are readily constructed in situ from three simple precursors and enable a highly modular installation of various substitution patterns. Upon subjection to a unique dual-catalytic system, the transient intermediates undergo an unusual hydride shuttle process that is initiated by a hydride donation event. Furthermore, we show that, in combination with asymmetric organocatalysis, the product alkaloid frameworks are obtained in excellent optical purity.

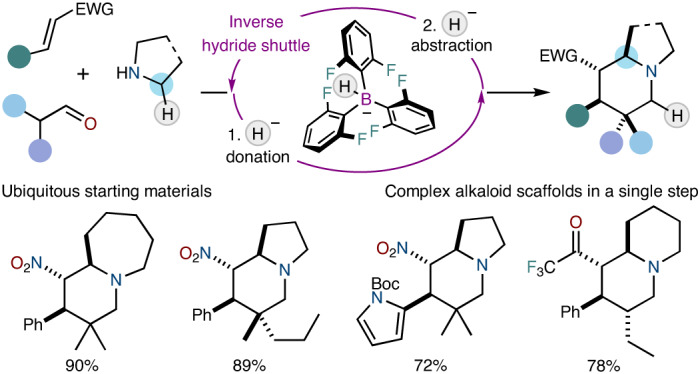

## Abstract (web summary)

The rapid assembly of complex scaffolds in a single step from simple precursors would be an ideal reaction in terms of efficiency and sustainability. Now, the single-step construction of alkaloid-like frameworks from three dynamically assembled precursors has been reported. Using a dual-catalytic system, the transformation involves a hydride shuttle process initiated by a hydride donation event.

## Main

**Fig. 1:**
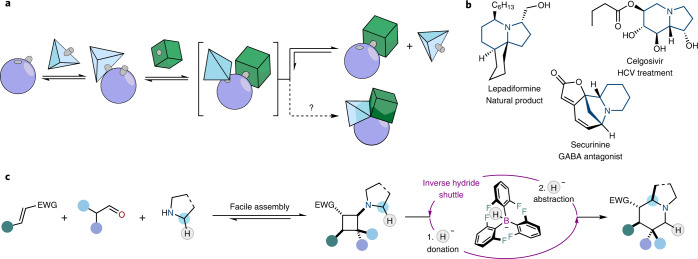
Harnessing dynamically formed ternary complexes through inverse hydride shuttle catalysis. **a**, Dynamically assembled complexes with unexploited synthetic potential: reversible preorganisation into ternary complexes and interrogation of routes able to convert the ternary complex directly into a complex scaffold. **b**, Azabicycles in natural products and pharmaceuticals. HCV, hepatitis C virus; GABA, γ-aminobutyric acid receptor. **c**, Azabicycles are formed in a single step, enabled by inverse hydride shuttle catalysis starting from ubiquitous starting materials. Fleeting cyclobutene intermediates are converted into complex frameworks with up to 99% yield, forming up to four new stereocentres with excellent enantioselectivity (up to 99% e.e.). EWG, electron-withdrawing group.

Contemporary organic synthesis aims to further our understanding of nature by developing laboratory routes to molecules that mimic those produced by living organisms with ever-increasing levels of complexity. The ideal synthesis of a target compound is most often described in terms of the number of synthetic operations required to reach said product—with ideality lying as close to one single step as possible^1^. Due to their modular nature and their ability to rapidly generate multiple new bonds, multi-component reactions often equate with highly efficient syntheses^2,3^; however, it is notoriously difficult to design multi-component reactions leading to valuable targets. In particular, we believe large untapped potential exists in dynamically assembled complexes, formed under equilibrium, that preorganise multi-component arrays of reactants (Fig. 1a). Converting these complexes into templates for the deployment of catalytic methods offers a challenging but potentially rewarding avenue towards highly complex products^4,5^.

Alkaloids remain a highly prized and elaborate subset of natural products and drug candidates^6^. Azabicyclic alkaloids, structures where two fused rings share a nitrogen atom, are prevalent throughout nature with potent and diverse biological activities (Fig. 1b)^7^. Indeed, over 1,800 secondary metabolites contain the indolizidine core, and more than 2,000 naturally occurring pyrrolizidine and quinolizidine derivatives are known^8^. While synthetic chemistry has risen to the challenge of preparing such targets in the laboratory, their complexity still renders such efforts as multistep endeavours^9–12^.

Herein, we report the one-step, multi-component conversion of bulk chemicals (cyclic amines, electron-deficient olefins and aldehydes) into complex bicyclic alkaloid scaffolds via inverse hydride shuttle catalysis (so termed because it is initiated by a hydride donation event rather than an abstraction event)^13–16^. This asymmetric transformation harnesses dynamically formed complexes that assemble the precursors and forges valuable products carrying up to four new stereocentres with excellent enantio- and diasteroselectivities, in a synthetically ideal manner (Fig. 1c).

## Results and discussion

Interested in leveraging skeletal reorganisation of reversibly assembled, dynamic complexes formed by a multi-component equilibrium process, we turned to the reversible addition of enamines to Michael acceptors, known to transiently generate fleeting donor–acceptor cyclobutanes^17–20^, and explored a range of sterically constrained, boron-based Lewis acids to evoke a formal ring expansion (Table 1). Interestingly, B(C_6_F_5_)_3_ **1a**—a commonly employed Lewis acid^21–24^—failed to provide the desired product, returning only unreacted starting material. At the opposite end of the Lewis acidity scale, Ph_3_B **1e** similarly did not lead to the formation of any observable product. Given that boron-based Lewis acids at both ends of the Lewis acidity scale had given poor results, it was clear that careful tuning of the electronic properties of the Lewis acid was required. We thus found that stoichiometric amounts of tris-(2,6-difluorophenyl)borane **1c** (refs. ^25,26^) can promote the desired skeletal rearrangement. Converting this process into a catalytic variant (Supplementary Section 2 for details) required the combination of catalytic quantities of both Lewis acid **1c** and its preformed tetraalkylammonium hydride **1c–H**.

**Table 1:**
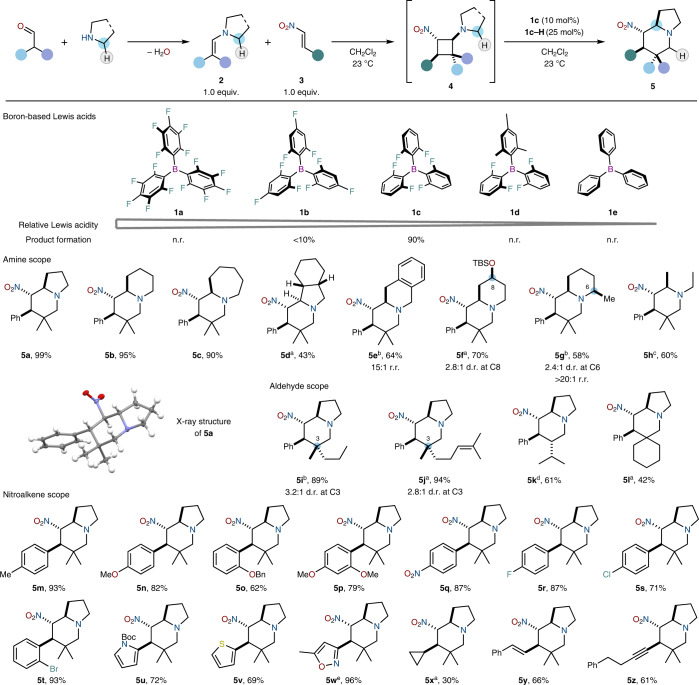
Rapid synthesis of alkaloid-like azabicycles by inverse hydride shuttle catalysis

Reaction conditions: CH_2_Cl_2_, 25 °C, 0.5 to 3 h, then slow addition over 30 min to **1c** (10 mol%), **1c–H** (25 mol%), followed by stirring of the reaction mixture at 25 °C for 1 h. Products were formed as single diastereomers, unless stated otherwise. ^a^30 mol% **1c** and 30 mol% **1c–H** were used. ^b^50 mol% **1c** and 50 mol% **1c–H** were used. ^c^40 mol% **1c** and 40 mol% **1c–H** were used. ^d^The solution of **4** was cooled to 0 °C. ^e^20 mol% **1c** and 20 mol% **1c–H** were used. TBS, *tert*-butyldimethylsilyl; n.r., no reaction; r.r., regioisomeric ratio.

Several complex azabicyclic structures are accessible through this transformation. As shown in Table 1, bicyclo[4.3.0], –[4.4.0] and –[5.4.0] systems can be prepared in a single step, affording the products as single diastereomers featuring three contiguous s tereogenic centres (**5a**–**5c**). The use of fused-ring reactants (**5d** and **5e**), as well as the introduction of additional substituents, allows a rapid increase in the complexity of these alkaloid-like products (**5f** and **5g**). Acyclic secondary amines were also amenable to this method, leading to multi-substituted piperidine derivatives (**5h**). While the use of linear aldehydes enables the diastereospecific formation of azabicyclic cores carrying an additional stereogenic centre (**5k**), employing cycloalkanecarbaldehydes readily affords spirocyclic structures (**5l**). We were pleased to find that the efficiency of the reaction is not adversely affected by the electronic nature of the nitroolefin: electron-donating (**5n**–**5p**) or electron-withdrawing (**5q**–**5t**) groups are well tolerated, as are heteroaromatics (**5u**–**5w**) and alkenyl (**5y**) and alkynyl (**5z**) substituents.

**Fig. 2:**
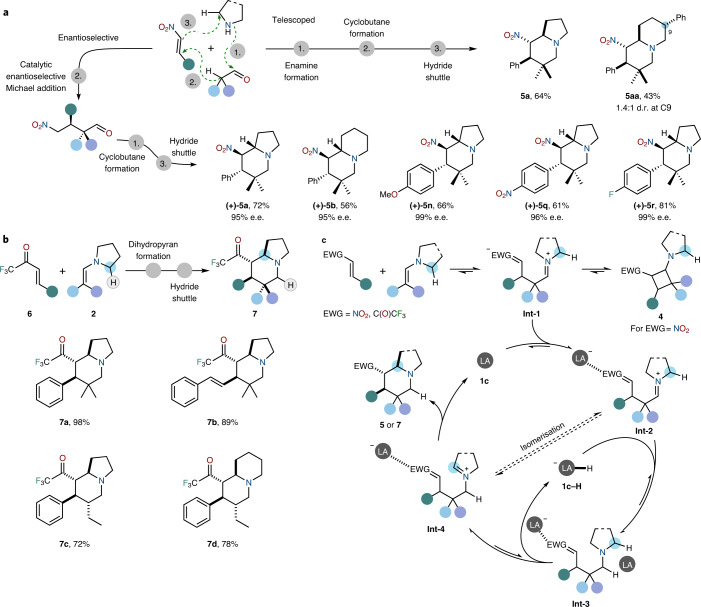
Extensions of the inverse hydride shuttle concept: enantioselective synthesis, additional substrate class and mechanistic proposal. **a**, Telescoped and enantioselective approaches for the synthesis of indolizidine building blocks. Telescoped approach starting directly from amine, aldehyde and nitroolefin. Enantioselective access to the azabicyclic frameworks is based on an organocatalysed enantioselective Michael addition prior to cyclobutane formation. **b**, Alternative Michael acceptors: synthesis of indolizidine derivatives bearing a trifluoromethyl ketone. **c**, Proposed mechanism for the conversion of enamine–Michael acceptor complexes, formed through dynamic assembly, into indolizidines via inverse hydride shuttle catalysis featuring both a Lewis acid and its respective hydride. LA, Lewis acid. Supplementary Sections 4.5, 4.6 and 4.7 for details. Products were formed as single diastereomers, unless stated otherwise.

Performing the reaction in a telescoped fashion is also possible (Fig. 2a). To this effect, a sequence of (1) enamine condensation, (2) cyclobutane formation and (3) hydride shuttle ring reorganisation can be carried out in a single step, leading to product yields comparable to those of the standard procedure. Furthermore, a change in the order of events enables an enantioselective approach. As also shown in Fig. 2a, if a catalytic enantioselective Michael addition first couples the aldehyde and the Michael acceptor, the events of cyclobutane formation and the hydride shuttle deliver virtually enantiopure azabicyclic products (**(+)-5a**–**(+)-5r**).

The range of electron-deficient olefins also encompasses trifluoromethyl ketones (**6**), showcasing the potential breadth of the concept presented herein. When used in combination with enamine **2**, such substrates elicit transient formation of a dihydropyran (**8**; Supplementary Fig. 2) which is then cleanly converted into azabicycles **7** in high yields upon addition to the catalyst (Fig. 2b).

Our mechanistic proposal is outlined in Fig. 2c. Depending on the nature of the two reactants, either a cyclobutane or a dihydropyran is transiently formed (detected by ^1^H NMR; Supplementary Sections 4.4.2 and 4.6.10 for details). These transient species have been shown to reside in dynamic equilibrium with their respective precursors via open, zwitterionic forms **Int-1** (refs. ^18,19^). When exposed to the catalytic system, the iminium moiety is swiftly reduced by hydride species **1c–H** to form tertiary amine **Int-3**. Hydride abstraction at the sterically most accessible position affords the formally reorganised iminium ion **Int-4** while regenerating **1c–H** (refs. ^27–30^). Subsequently, **Int-4** spontaneously collapses to the product, releasing **1c**, thereby closing the dual-catalytic cycle.

**Fig. 3:**
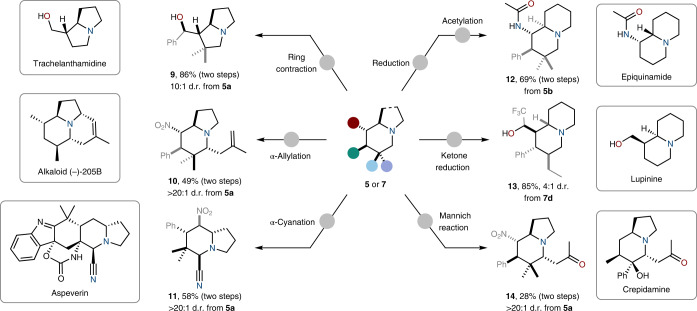
Targeted functionalisation reactions of the azabicyclic framework towards natural-product-like scaffolds. Diazonium extrusion followed by ring contraction to forge 5,5-bicyclic scaffold **9** resembling the trachelanthamidine family. α-Oxidation followed by highly stereoselective nucleophilic allylation affording **10**. Formation of a truncated aspeverin derivative **11** by α-cyanation. Reduction of the nitro-group and acetylation lead to an epiquinamide derivative **12**. Trifluoroketone reduction affords **13**, featuring a decorated lupinine framework. Polonovski–Potier reaction and acetone addition furnish **14**, resembling crepidamine. Supplementary Section 4.8 for details.

The rapidly assembled alkaloid cores can be easily converted into a variety of natural-product-like scaffolds (Fig. 3). Other ring architectures such as the trachelanthamidine framework (substructure for over 250 alkaloids^31^) are accessible by ring contraction (**9**) leading to the corresponding pyrrolizidine core. In addition, a variety of naturally occurring substituents are introduced with high regio- and stereoselectivity adjacent to the bicyclic nitrogen atom via the Polonovski–Potier reaction (**10**, **11** and **14**; Fig. 3). Moreover, reduction of **5b** and **7d** leads to frameworks related to epiquinamide and lupinine, respectively.

In summary, we have developed a modular protocol for the one-step synthesis of complex frameworks deploying inverse hydride shuttle catalysis onto dynamically assembled complexes generated at equilibrium^32,33^. The method results in a variety of alkaloid-like products formed in an enantio- and diastereoselective manner. We believe that the approach presented herein has the potential to revolutionise the design of multi-component reactions, facilitating the breakthrough advances in biology and medicine that modern society relies on.

## Methods

### Caution statement when working with LAH in large scale

Quenching of reactions with LiAlH_4_ must not be performed using hydrochloric acid. In this case we recommend cooling the reaction mixture to 0 °C and quenching potentially unreacted LAH by slow addition of an excess of ethyl acetate.

### General procedure for the inverse hydride shuttle

To a 4.00 ml vial containing enamine **2** (250 µmol, 1.00 equiv.) was added a solution of nitrostyrene **3** in CH_2_Cl_2_ (0.70 ml of a 2.8 M solution, 250 µmol, 1.00 equiv.) at room temperature (23 °C) and the solution was stirred for 1–3 h. Over the course of 20 min, the solution was added to a solution of Lewis acid **1c** (10 mol%) and its hydride **1c–H** (25 mol%) in CH_2_Cl_2_ (200 µl) using a syringe pump. After the addition was complete, the reaction was stirred for 1 h at room temperature before the solvent was removed under reduced pressure. Analysis of the crude mixture by ^1^H NMR showed the formation of a single diastereomer unless stated otherwise. The residue was purified by flash column chromatography (heptane/CH_2_Cl_2_ 1:1 grading to pure CH_2_Cl_2_, unless stated otherwise) to afford the final product.

## Online content

Any methods, additional references, Nature Research reporting summaries, source data, extended data, supplementary information, acknowledgements, peer review information; details of author contributions and competing interests; and statements of data and code availability are available at 10.1038/s41557-022-00991-4.

## Supplementary information

Supplementary Information Supplementary Figs. 1–7, Tables 1–8, Discussion, optimisation details, experimental and procedural details, synthesis and characterisation data, HPLC traces, NMR spectra and X-ray crystallographic data.

Supplementary Data 1 Crystallographic data for compound 5a; CCDC reference 1973786.

Supplementary Data 2 Crystallographic data for compound 9; CCDC reference 2079162

## Data Availability

All data in support of the findings of this study are available within the article and its Supplementary Information. Crystallographic data for the structures reported in this article have been deposited at the Cambridge Crystallographic Data Centre (CCDC) under deposition numbers CCDC 1973786 (**5a**) and CCDC 2079162 (**9**).
