# Supplementary material for: Inverse hydride shuttle catalysis enables the stereoselective one-step synthesis of complex frameworks
Source: Nat Chem. 2022 Oct 20;14(11):1306–10. doi: 10.1038/s41557-022-00991-4 (PMC9630112; doi:10.1038/s41557-022-00991-4)
Supplement: Supplementary file 1 — Supplementary Figs. 1–7, Tables 1–8, Discussion, optimisation details, experimental and procedural details, synthesis and characterisation data, HPLC traces, NMR spectra and X-ray crystallographic data. [file 41557_2022_991_MOESM1_ESM.pdf]

nature portfolio

https://doi.org/10.1038/s41557-022-00991-4

Supplementary information

Inverse hydride shuttle catalysis enables the stereoselective one-step synthesis of complex frameworks

In the format provided by the authors and unedited

Inverse hydride shuttle catalysis enables the stereoselective one-step synthesis of complex frameworks

Authors: Immo Klose[1], Giovanni Di Mauro[1], Dainis Kaldre[1] and Nuno Maulide[1]*

Supplementary Information

Affiliations:

[1] Institute of Organic Chemistry, University of Vienna, Währinger Strasse 38, 1090 Vienna, Austria

*Correspondence to: nuno.maulide@univie.ac.at

1 Contents

Materials and Methods 3

Optimisation study 4

Origin of Diastereo– and Regioselectivity 6

Experimental Procedures, Characterisation Data 7

- **Synthesis of enamines** 7
- **Synthesis of electron-deficient olefins** 11
- **Synthesis of Boronic Lewis Acids and its hydride** 14
- **Inverse hydride shuttle catalysis** 15
- **Telescoped approach: One step reaction** 31
- **Enantioselective approach** 32
- **Alternative Michael acceptors** 40
- **Functionalisation** 44

References 50

NMR-Spectra 52

X-ray Analysis 121

1. Materials and Methods

Unless otherwise stated, all glassware was flame-dried before use and all reactions were performed under an atmosphere of argon. All reagents were used as received from commercial suppliers unless otherwise stated. Anhydrous solvents were obtained from commercial suppliers and used as received. Reaction progress was monitored by thin layer chromatography (TLC) performed on aluminum plates coated with silica gel F_{254} with 0.2 mm thickness. Chromatograms were visualised by fluorescence quenching with UV light at 254 nm or by staining using potassium permanganate. Flash column chromatography was performed using silica gel 60 (230-400 mesh, Merck and co.). Neat infrared spectra were recorded using a Perkin-Elmer Spectrum 100 FT-IR spectrometer. Wavenumbers (ν_{max}) are reported in cm^{-1}. Mass spectra were obtained using a Bruker maXis UHR-TOF (Qq-TOF) spectrometer, using electrospray ionisation (ESI^+). Optical rotations were measured on a Perkin Elmer 341 polarimeter using a 100 mm path-length cell at 589 nm (c given in g/100 mL). Chiral HPLC was performed using AGILENT Infinity 1260 with Chiralpak IC or Lux-3 Cellulose-3 columns. Details on chromatographic conditions are indicated under each compound. All ^1H NMR and ^{13}C NMR spectra were recorded using a Bruker AV-400, AV-600 or AV-700 spectrometer at 300K. Chemical shifts are given in parts per million (ppm, δ), referenced to the solvent peak of $CDCl_3$ or toluene-d_8 defined at δ = 7.26 ppm or δ = 2.08 ppm respectively (^1H NMR) and δ = 77.16 (^{13}C NMR). Coupling constants are quoted in Hz (*J*). ^1H NMR splitting patterns are designated as singlet (s), doublet (d), triplet (t), quartet (q), septet (sept) as they appeared in the spectrum. If the appearance of a signal differs from the expected splitting pattern, the observed pattern is designated as apparent (app). Splitting patterns that could not be interpreted or easily visualised are designated as multiplet (m) or broad (br). Carbon signals that were only observed in HSQC or HMBC are reported indicated by HSQC or HMBC in parenthesis.

2 Optimisation study

Lewis acid screening

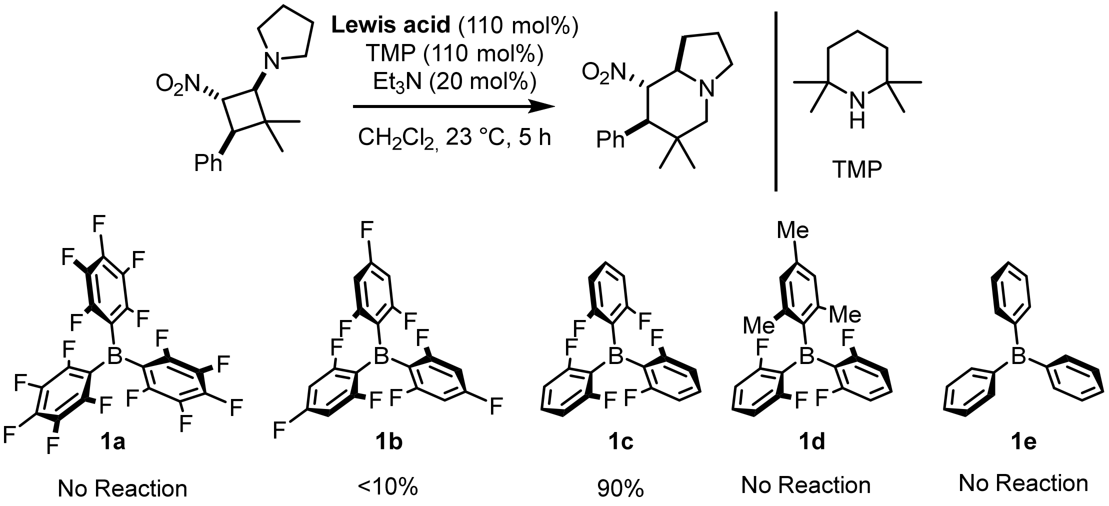

Table S1. Stoichiometric reaction with different Lewis acids.

Screening of reaction conditions

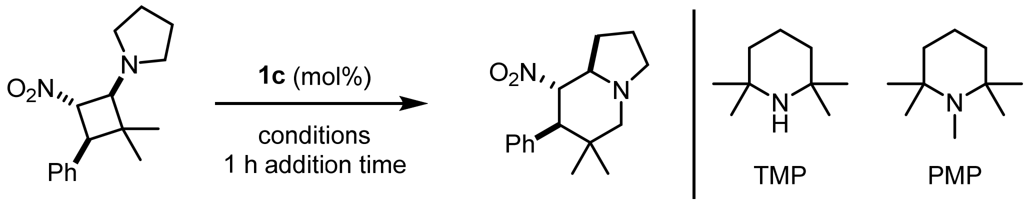

Entry	**1c**/mol%	additive (mol%)	solvent	temperature	yield (NMR)*
1†	110	TMP (110)<br>Et_3N (20)	CH_2Cl_2	rt	90
2	50	Et_3N (50)	CH_2Cl_2	rt	46
3	50	Et_3N (10)	CH_2Cl_2	rt	46
4	50	Et_3N (50)	CH_2Cl_2	–20 °C	0
5	50	Et_3N (50)	$C_2H_4Cl_2$	50 °C	33
6	50	PMP (50)	CH_2Cl_2	rt	10
7	50	$CyNMe_2$ (50)	CH_2Cl_2	rt	45

Table S2. Screening of reaction conditions with slow addition of a solution of *in situ*-formed cyclobutane to the solution of **1c** and additives. * NMR yield using diiodomethane as internal standard. † A solution of **1c** and TMP was added in one portion to a solution of *in situ*-formed cyclobutane followed by addition of Et_3N.

After the initial results of stoichiometric experiments (Table S2, Entry 1), we investigated a possible catalytic variant. However, reducing the amount of Lewis acid led to drastically reduced product formation. It was found that by slow addition of cyclobutane solution to the Lewis acid and a suitable amine additive, the product was obtained with close to one turnover (Entry 2, 3). Reasoning that the amine functions as hydride donor in these reactions, the reaction was investigated using a preformed hydride species directly (see Table S3).

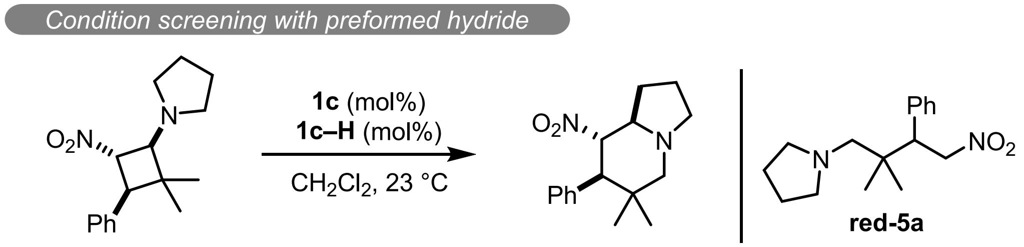

Entry	**1c**/mol%	**1c–H**/mol%	addition time	yield (NMR)*
1	30	30	40 min addition	71
2	30	30	20 min addition	81
3	15	15	20 min addition	52
4	35	5	20 min addition	28
5	5	35	20 min addition	96
6	5	25	20 min addition	67
**7**	**10**	**25**	**20 min addition**	**quant. (99)**†
8	0	25	20 min addition	<5‡

Table S3. Screening of reaction conditions with **1c** and its preformed hydride **1c–H**. * NMR yield using trimethoxybenzene as internal standard. † Isolated yield. ‡ Only trace amounts of product detected, **red-5a** was detected as major product.

Using the preformed hydride **1c–H** in combination with **1c** enabled the catalytic hydride shuttle (Table S3, Entry 1). Lowering the loading to 10 mol% of **1c** in combination with 25 mol% of **1c–H** was found to be optimal for this reaction, leading to a cleaner reaction profile and an isolated yield of 99% (Entry 7). **1c–H** alone led to a low conversion and the formation of **red-5a** (Entry 8).

3 Origin of Diastereo– and Regioselectivity

Diastereoselectivity during cyclobutane or dihydropyrane formation

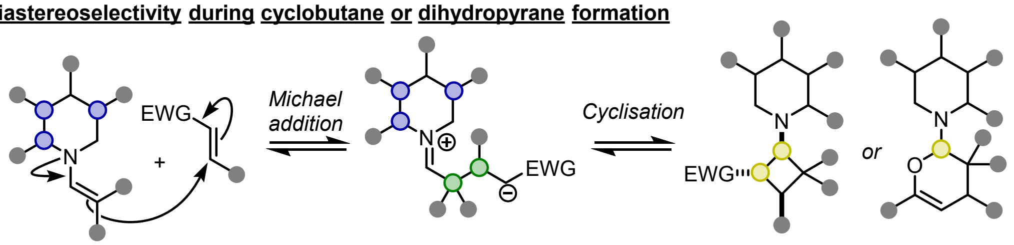

Generic substituent

Absolute stereochemistry dependent on starting secondary amine used for enamine synthesis. Relative stereochemistry determined during Michael-addition.

Stereochemistry determined during Michael-addition. Due to the reversible nature of the formation of these intermediates, the diastereomeric ratio can change over time until the thermodynamic ratio is reached.

Stereochemistry determined during cyclisation, **inconsequental** for the stereochemistry of the final azabicycles.
For amino-nitrocyclobutanes: the depicted *anti-anti* cyclobutane configuration has been the only observable diastereomer in all cases.
For dihydropyranes: with adjecent tertiary carbon: carbon depicted in yellow forms as a single diasteromer. With adjecent quaternary carbon: carbon depicted in yellow forms a mixture of two inconsequential epimers.

Regioselectivity

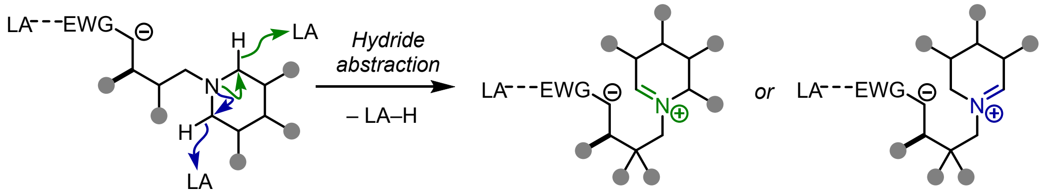

LA Lewis Acid
LA–H Lewis Acid Hydride

Regioselectivity in unsymmetric amines is determined by the location of hydride abstraction by the Lewis acid catalyst.

Diastereoselectivity during final cyclisation event

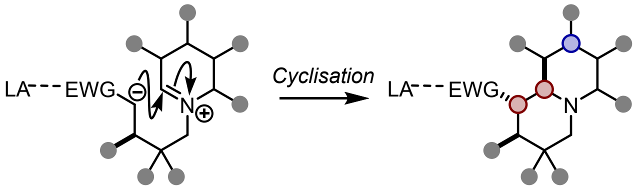

Relative stereochemistry is determined during the cyclisation event. The depicted *anti-anti* configuration has been the only observable diastereomer in all cases.

If a symmetric amine is used: relative stereochemistry is determined during the cyclisation event.

Figure S1. Overview of stereodeterimining elementary steps towards azabicyclic frameworks.

4 Experimental Procedures, Characterisation Data

4.1 Synthesis of enamines

Amines and aldehydes used for enamine formation were all commercially available and used as received.

General procedure for enamine formation from branched aldehydes:

In a round-bottom flask, branched aldehyde (12.0 mmol, 1.00 equiv.) was dissolved in anhydrous ether (4.0 mL, 3.0 M), and amine (12.5 mmol, 1.04 equiv.) and $MgSO_4$ (5.0 g) were added sequentially. The resulting suspension was stirred for 1 h at ambient temperature (23 °C). The mixture was filtered and the filtrate was treated with $MgSO_4$ (5.0 g) and stirred for 1 h at ambient temperature (23 °C). The mixture was filtered again and the filtrate was stirred for 12 h at ambient temperature (23 °C) over dried 4Å molecular sieves. The solvent was removed under reduced pressure and the residue was subjected to Kugelrohr distillation to afford the corresponding enamines.

General procedure for enamine formation from linear aldehydes:

In a round-bottom flask, linear aldehyde (10.0 mmol, 1 equiv) was added dropwise to the amine (15.0 mmol, 1.5 equiv) cooled at 0 °C under stirring and argon. K_2CO_3 (5.0 g) was then added and the suspension was stirred 14 h at ambient temperature (23 °C). The suspension was then filtered, washed with Et_2O (10.0 mL) and concentrated. The residue was subjected to Kugelrohr distillation was distilled to give the pure enamine to afford the corresponding enamines.

Enamines are prone to hydrolysis. A sample was checked after distillation by ^1H NMR and the material was stored in a glovebox (–20 °C) to allow long-term usage. Alternatively, enamines can be stored under protective atmosphere for several months in a conventional freezer.

4.1.1 2a – 1-(2-methylprop-1-en-1-yl)pyrrolidine[1]

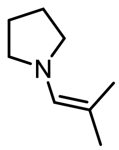

^{1}H NMR (400 MHz, $CDCl_3$) δ 5.66 – 5.55 (m, 1H), 2.98 – 2.88 (m, 5H), 1.80 – 1.74 (m, 4H), 1.69 (d, *J* = 0.7 Hz, 3H), 1.63 (d, *J* = 0.8 Hz, 3H).

4.1.2 2b – 1-(2-methylprop-1-en-1-yl)piperidine[2]

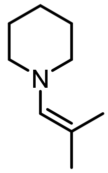

^{1}H NMR (400 MHz, $CDCl_3$) δ 5.35 – 5.30 (m, *J* = 5.4 Hz, 1H), 2.57 – 2.50 (m, 4H), 1.66 (d, *J* = 0.9 Hz, 3H), 1.63 – 1.51 (m, 7H), 1.48 – 1.37 (m, 2H).

4.1.3 2c – 1-(2-methylprop-1-en-1-yl)azepane[1]

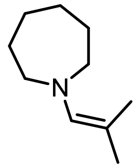

^{1}H NMR (400 MHz, $CDCl_3$) δ 5.67 – 5.53 (m, 1H), 2.99 – 2.90 (m, 4H), 1.66 (d, *J* = 0.7 Hz, 3H), 1.66 – 1.62 (m, 4H), 1.61 (d, *J* = 0.7 Hz, 3H), 1.60 – 1.55 (m, 4H).

4.1.4 2d – 2-(2-methylprop-1-en-1-yl)octahydro-*1H*-isoindole

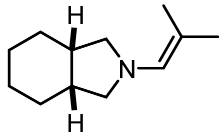

A round-bottom flask was charged with isobutyraldehyde (2.19 mL, 24.0 mmol, 1.50 equiv.), octahydroisoindole (2.00 g, 16.0 mmol, 1.00 equiv.) and 100 mL of toluene. A Dean-Stark trap was installed and the reaction was heated at reflux until water stopped condensing (5 h). The solvent was removed under reduced pressure and the residue was subjected to Kugelrohr distillation to afford the desired product (2.03 g, 71%) as a colourless oil.

^{1}H NMR (400 MHz, $CDCl_3$) δ 5.73 – 5.62 (m, 1H), 3.14 (dd, *J* = 9.1, 6.8 Hz, 2H), 2.94 (dd, *J* = 9.2, 5.5 Hz, 2H), 2.14 – 2.05 (m, 2H), 1.69 (d, *J* = 0.6 Hz, 3H), 1.62 (d, *J* = 0.6 Hz, 3H), 1.57 – 1.40 (m, 6H), 1.38 – 1.27 (m, 2H).

4.1.5 2e – 2-(2-methylprop-1-en-1-yl)-1,2,3,4-tetrahydroisoquinoline[3]

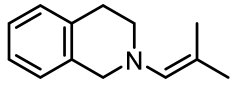

^{1}H NMR (400 MHz, $CDCl_3$) δ 7.21 – 7.05 (m, 3H), 7.05 – 6.94 (m, 1H), 5.61 – 5.44 (m, 1H), 3.82 (s, 2H), 2.91 (s, 4H), 1.74 (d, *J* = 0.9 Hz, 3H), 1.66 (d, *J* = 1.0 Hz, 3H).

4.1.6 2f – 4-((*tert*-butyldimethylsilyl)oxy)-1-(2-methylprop-1-en-1-yl)piperidine

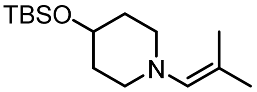

Synthesis of 4-((*tert*-butyldimethylsilyl)oxy)piperidine was performed according to literature procedure.[4]

2f was prepared according to the procedure for **2d** using isobutyraldehyde (1.00 mL, 11.0 mmol, 2.40 equiv.), 4-((*tert*-butyldimethylsilyl)oxy)piperidine (1.0 g, 4.60 mmol, 1.00 equiv.) and toluene (30.0 mL). Obtained as a pale orange oil (690 mg, 55%).

^{1}H NMR (400 MHz, $CDCl_3$) δ 5.39 – 5.34 (m, 1H), 3.69 (tt, *J* = 8.0, 4.0 Hz, 1H), 2.85 – 2.77 (m, 2H), 2.40 (ddd, *J* = 12.0, 9.1, 3.1 Hz, 2H), 1.78 – 1.71 (m, 2H), 1.66 (d, *J* = 0.9 Hz, 3H), 1.64 – 1.52 (m, 5H), 0.89 (s, 9H), 0.05 (s, 6H).

4.1.7 2g – 2-methyl-1-(2-methylprop-1-en-1-yl)piperidine[5]

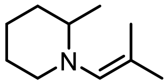

^{1}H NMR (400 MHz, $CDCl_3$) δ 5.22 – 5.14 (m, 1H), 2.72 (dtd, *J* = 11.4, 3.6, 1.5 Hz, 1H), 2.23 – 2.12 (m, 2H), 1.70 – 1.64 (m, 4H), 1.64 – 1.51 (m, 6H), 1.30 – 1.21 (m, 2H), 0.93 (d, *J* = 6.3 Hz, 3H).

4.1.8 2h – *N*,*N*-diethyl-2-methylprop-1-en-1-amine[6]

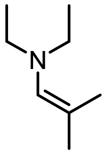

^{1}H NMR (400 MHz, $CDCl_3$) δ 5.27 – 4.95 (m, 1H), 2.54 (q, *J* = 7.2 Hz, 4H), 1.65 (d, *J* = 10.2 Hz, 6H), 0.96 (t, *J* = 7.2 Hz, 6H).

4.1.9 2i – *(E)-/(Z)*-1-(2-methylpent-1-en-1-yl)pyrrolidine[1]

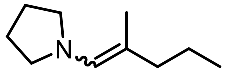

^{1}H NMR (400 MHz, $CDCl_3$) δ 5.66 – 5.61 (m, 0.7H), 5.61 – 5.56 (m, 0.3H), 3.05 – 2.86 (m, 4.0H), 2.13 – 2.06 (m, 0.6H), 1.93 – 1.84 (m, 1.4), 1.82 – 1.72 (m, 4.0H), 1.66 (d, *J* = 1.2 Hz, 2.1H), 1.60 (d, *J* = 1.3 Hz, 0.9H), 1.48 – 1.33 (m, 2.0H), 0.92 (t, *J* = 7.3 Hz, 0.9H), 0.85 (t, *J* = 7.3 Hz, 2.1H).

4.1.10 2j – (*E*)-/(*Z*)-1-(2,6-dimethylhepta-1,5-dien-1-yl)pyrrolidine

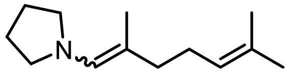

2j was prepared according to the general procedure using branchend aldehydes on a 30.3 mmol scale using 2,6-dimethyl-5-heptanal (melonal, 5.00 mL, 30.3 mmol, 1.00 equiv.) and pyrrolidine (2.60 mL, 31.7 mmol, 1.05 equiv.). The title compound (1.23 g, 21%, 2.5:1 mixture of *E*-/*Z*-isomers) was obtained as a colourless oil.

^{1}H NMR (600 MHz, $CDCl_3$) δ 5.68 – 5.63 (m, 0.7H), 5.60 (s br, 0.3H), 5.19 – 5.13 (m, 0.3H), 5.13 – 5.07 (m, 0.7H), 2.99 – 2.89 (m, 4.0H), 2.17 – 2.12 (m, 0.6H), 2.12 – 2.07 (m, 0.6H), 2.07 – 2.02 (m, 1.4H), 1.96 – 1.90 (m, 1.4H), 1.80 – 1.74 (m, 4H), 1.72 – 1.65 (m, 5.1H), 1.63 – 1.61 (m, 1.8H), 1.60 (s, 2.1H).

4.1.11 2k – (*E*)-1-(3-methylbut-1-en-1-yl)pyrrolidine[7]

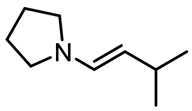

^{1}H NMR (600 MHz, $CDCl_3$) δ 6.16 (d, *J* = 13.8 Hz, 1H), 4.12 (dd, *J* = 13.8, 7.0 Hz, 1H), 2.95 (s br, 4H), 2.36 – 2.15 (m, 1H), 1.83 (s br, 4H), 0.98 (d, *J* = 6.7 Hz, 6H).

4.1.12 2l – 1-(cyclohexylidenemethyl)pyrrolidine[2]

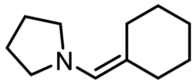

^{1}H NMR (400 MHz, $CDCl_3$) δ 5.57 (s br, 1H), 2.97 – 2.87 (m, 4H), 2.28 – 2.17 (m, 2H), 2.01 – 1.95 (m, 2H), 1.79 – 1.69 (m, 4H), 1.56 – 1.42 (m, 6H).

4.1.13 2m – (*E*)-1-(but-1-en-1-yl)pyrrolidine[2]

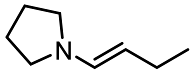

^{1}H NMR (400 MHz, $CDCl_3$) δ 6.18 (d, *J* = 13.7 Hz, 1H), 4.16 (dt, *J* = 13.6, 6.7 Hz, 1H), 3.07 – 2.90 (m, 4H), 1.99 (td, *J* = 7.4, 6.7 Hz, 2H), 1.87 – 1.76 (m, 4H), 0.96 (t, *J* = 7.4 Hz, 3H).

4.1.14 2n – (*E*)-1-(but-1-en-1-yl)piperidine[8]

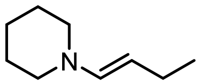

^{1}H NMR (400 MHz, $CDCl_3$) δ 5.82 (d, *J* = 13.9 Hz, 1H), 4.41 (dt, *J* = 13.7, 6.7 Hz, 1H), 2.76 – 2.69 (m, 4H), 2.01 – 1.92 (m, 2H), 1.61 – 1.52 (m, 4H), 1.52 – 1.43 (m, 3H), 0.95 (t, *J* = 7.4 Hz, 3H).

4.2 Synthesis of electron-deficient olefins

Starting Materials for **3a**, **3m**, **3n**, **3o**, **3p**, **3q**, **3r**, **3s**, **3t**, **3v**, **3w** are commercially available and were used as received. Starting Materials for **3u**,[9] **3x,**[10] **3y,**[11,12] were prepared according to literature procedures.

Trifluoromethyl ketones **7aSM** and **7bSM** for the synthesis of **7a (7c, 7d)** and **7b** respectively, were prepared according to literature procedure.[12]

General procedure for nitroolefins:[10]

A slurry of $LiAlH_4$ (8.00 mg, 0.20 mmol, 0.10 equiv.) in anhydrous THF (7.00 mL) was stirred at 0 °C for 30 min, after which nitromethane (540 µL, 10.0 mmol, 5.00 equiv.) was added. After 30 min, aldehyde (2.00 mmol, 1.00 equiv.) was added at 0 °C, and the resulting mixture was stirred for 12 hours, during which time the reaction mixture reached 23 °C. Aqueous HCl (1.0 M, 3.00 mL) and water (5.00 mL) were added and the aqueous layer was extracted two times with CH_2Cl_2 (2 x 10.0 mL).

Safety disclaimer: on larger scales, quenching of reactions with $LiAlH_4$ must not be performed using hydrochloric acid. In this case we recommend cooling the reaction mixture to 0 °C and quenching potentially unreacted $LiAlH_4$ by slow addition of an excess of ethyl acetate.

The combined organic layers were dried over anhydrous Na_2SO_4, filtered and concentrated under reduced pressure to afford a crude residue which is used without purification for the next step.

To a solution of the nitro-alcohol (1.50 mmol, 1.00 equiv.) obtained above in anhydrous CH_2Cl_2 (4.00 mL) was added trifluoroacetic anhydride (220 µL, 1.58 mmol, 1.05 equiv.) and NEt_3 (440 µL, 3.15 mmol, 2.10 equiv.) at 0 °C. The reaction mixture was stirred for 1 h at 0 °C. CH_2Cl_2 was added and then the organic phases were washed with water (5.00 mL), saturated solution of NH_4Cl (5.00 mL) and brine (5.00 mL). The organic layer was dried over anhydrous Na_2SO_4, filtered and concentrated under reduced pressure. The crude mixture was purified by flash column chromatography on silica gel (heptane/ethyl acetate 10:1 to 2:1) to afford the desired nitroolefine.

4.2.1 3u – *tert*-butyl (*E*)-2-(2-nitrovinyl)-1H-pyrrole-1-carboxylate[9]

Following a reported procedure,[9] a solution of aldehyde (3.70 mmol, 1.00 equiv.) in distilled methanol (11.0 mL) was treated with nitromethane (0.60 mL, 11.1 mmol, 3.00 equiv), sodium acetate (334 mg, 4.07 mmol, 1.10 equiv.), and methylamine hydrochloride (275 mg, 4.07 mmol, 1.10 equiv.). The mixture was stirred at ambient temperature (23 °C) for 21 h under argon. The methanol was removed *in vacuo* without heating to give a yellow solid. The solid was dissolved in CH_2Cl_2 (100 mL), and the resulting solution was washed with water. The organic extract was dried (Na_2SO_4), filtrated, concentrated and purified by flash column chromatography on silica gel (CH_2Cl_2) to give the depicted product (323 mg, 37%) as yellow crystalline solid.

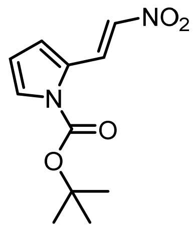

^{1}H NMR (400 MHz, $CDCl_3$) δ 8.75 (d, *J* = 13.5 Hz, 1H), 7.53 (dd, *J* = 3.2, 1.5 Hz, 1H), 7.47 (d, *J* = 13.5 Hz, 1H), 6.83 (dd, *J* = 3.6, 1.5 Hz, 1H), 6.30 (app t, *J* = 3.5 Hz, 1H), 1.65 (s, 9H); **^{13}C NMR** (101 MHz, $CDCl_3$) δ 148.6, 135.1, 130.0, 127.7, 126.3, 118.1, 112.3, 86.1, 28.1 (3C).

4.2.2 3x – (*E*)-(2-nitrovinyl)cyclopropane[10]

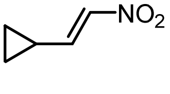

^{1}H NMR (400 MHz, $CDCl_3$) δ 7.13 (d, *J* = 13.1 Hz, 1H), 6.78 (dd, *J* = 13.1, 10.8 Hz, 1H), 1.62 (dtt, *J* = 10.8, 8.0, 4.5 Hz, 1H), 1.26 – 1.06 (m, 2H), 0.93 – 0.66 (m, 2H); **^{13}C NMR** (101 MHz, $CDCl_3$) δ 148.9, 137.5, 11.6, 9.8 (2C).

4.2.3 3y – ((1*E*,3*E*)-4-nitrobuta-1,3-dien-1-yl)benzene[11,12]

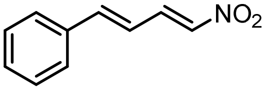

^{1}H NMR (400 MHz, $CDCl_3$) δ 7.78 (ddd, *J* = 13.0, 11.6, 0.7 Hz, 1H), 7.55 – 7.48 (m, 2H), 7.44 – 7.36 (m, 3H), 7.24 (d, *J* = 13.1 Hz, 1H), 7.16 (d, *J* = 15.5 Hz, 1H), 6.87 (ddd, *J* = 15.5, 11.6, 0.6 Hz, 1H); **^{13}C NMR** (101 MHz, $CDCl_3$) δ 146.2, 139.3, 138.8, 135.3, 130.5, 129.2 (2C), 127.9 (2C), 120.7.

4.2.4 3z – (*E*)-(6-nitrohex-5-en-3-yn-1-yl)benzene

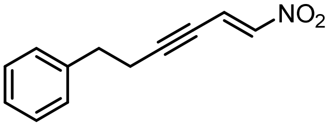

Synthesis of 5-phenylpent-2-ynal was performed according to literature procedure.[13]

3z was synthesised according to general procedure.[10] The final compound (205 mg, 51%) was obtained as a yellow oil.

^{1}H NMR (400 MHz, $CDCl_3$) δ 7.35 – 7.29 (m, 2H), 7.27 – 7.15 (m, 4H), 7.10 (dt, J = 13.4, 2.3 Hz, 1H), 2.90 (t, J = 7.3 Hz, 2H), 2.74 (td, J = 7.3, 2.2 Hz, 2H); **^{13}C NMR** (101 MHz, $CDCl_3$) δ 145.8, 139.7, 128.6 (2C), 128.4 (2C), 126.7, 121.6, 107.1, 74.4, 34.2, 22.2; **HRMS (ESI$^+$):** exact mass calculated for $[M+Na]^+$ ($C_{12}H_{11}NO_2Na^+$) requires *m/z* 224.0682, found *m/z* 224.0680; **IR** (thin film) ν 2219, 1619, 1523, 1342, 939, 699 cm^{-1}.

General procedure for trifluoromethyl ketones:

To a stirred solution of aldehyde (12.5 mmol), acetic acid (1.08 mL, 18.8 mmol, 1.5 equiv.) and piperidine (1.24 mL, 12.5 mmol, 1.00 equiv.) in dry toluene (10 mL) at 0 °C was added dropwise a solution of trifluoroacetone (4.63 mL, 50.1 mmol, 4.00 equiv.) in dry toluene (10 mL). The mixture was stirred for 2 h at this temperature and 24 h at ambient temperature (23 °C). The reaction was quenched with a saturated aqueous solution of ammonium chloride (15 mL) and was extracted with EtOAc (3x 20 mL). The organic layer was washed with brine (20 mL) and then dried over anhydrous sodium sulfate. The solution was filtrated and concentrated under reduced pressure. The residue was purified by flash column chromatography (n-pentate to n-pentane/Et2O 2:1) to afford the respective trifluoroketone.

4.2.5 S1a – (*E*)-1,1,1-trifluoro-4-phenylbut-3-en-2-one[12]

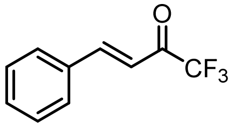

^{1}H NMR (400 MHz, $CDCl_3$) δ 7.98 (d, *J* = 16.0 Hz, 1H), 7.65 (d, *J* = 7.5 Hz, 2H), 7.56 – 7.39 (m, 3H), 7.02 (d, *J* = 16.0 Hz, 1H); **^{13}C NMR** (151 MHz, $CDCl_3$) δ 180.2 (q, *J* = 35.5 Hz), 150.3, 133.5, 132.5, 129.4 (4C), 116.8, 116.5 (q, *J* = 290.7 Hz); **^{19}F NMR** (565 MHz, $CDCl_3$) δ -77.59.

4.2.6 S1b – (3*E*,5*E*)-1,1,1-trifluoro-6-phenylhexa-3,5-dien-2-one[12]

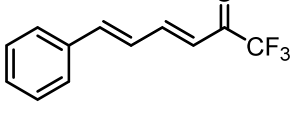

^{1}H NMR (600 MHz, $CDCl_3$) δ 7.74 (dd, *J* = 15.2, 11.2 Hz, 1H), 7.58 – 7.47 (m, 2H), 7.45 – 7.35 (m, 3H), 7.16 (d, *J* = 15.5 Hz, 1H), 7.03 – 6.94 (m, 1H), 6.56 (d, *J* = 15.1 Hz, 1H); **^{13}C NMR** (151 MHz, $CDCl_3$) δ

180.2 (q, J = 35.1 Hz), 150.1, 146.4, 135.5, 130.5, 129.2 (2C), 128.0 (2C), 126.1, 120.0, 116.6 (q, J = 290.9 Hz); ^{19}**F NMR** (565 MHz, $CDCl_3$) δ -77.42.

4.3 Synthesis of Boronic Lewis Acids and its hydride

4.3.1 1c–THF – tris(2,6-difluorophenyl)borane-THF adduct

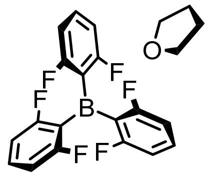

Synthesis of B(2,6-$F_2C_6H_3$)$_3$–THF was performed according to literature procedure. All spectroscopic data were in accordance with those reported in the literature.[14]

To magnesium turnings (1.20 g, 50.0 mmol, 1.00 equiv.) in THF (50.0 mL) at –20 °C was added a solution of 2,6-difluorbromobenzene (9.64 g, 50.0 mmol, 1.00 equiv.) in THF (20.0 mL). After stirring for 4 hours, the resulting suspension was allowed to warm to room temperature and stirred overnight. The solution was consequently added dropwise at –20 °C to a solution of BF_3·OEt_2 (2.41 g, 17.0 mmol, 0.34 equiv.) in toluene (70.0 mL) and stirred for 4 h. After warming to room temperature, the solvent was evaporated under reduced pressure.Hexane (100 mL) was added to the residue and the mixture heated to reflux for 4 h. The precipitated magnesium salts were filtered while the solution was still hot and the filtrate was then allowed to reach room temperature, during which time the product crystalises. After a recrystallisation from hexanes, the product (1.59 g, 7.5%) was obtained as a white needles.

^{1}**H NMR** (600 MHz, Tol-d_8) δ 6.78 – 6.71 (m, 3H), 6.49 (app t, J = 7.9 Hz, 6H), 3.45 – 3.39 (m, 4H), 1.31 – 1.27 (m, 4H). ^{13}**C NMR** (151 MHz, Tol-d_8) δ 166.2 (dd, J = 247.9, 13.4 Hz), 133.1 (t, J = 10.3 Hz), 111.5 (d, J = 28.4 Hz), 68.6 (s), 25.8 (s).

4.3.2 1c – tris(2,6-difluorophenyl)borane

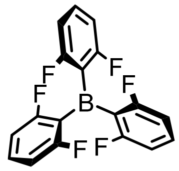

Synthesis of B(2,6-$F_2C_6H_3$)$_3$ was performed according to literature procedure. All spectroscopic data were in accordance with those reported in the literature.[14]

1c –THF (100 mg, 240 µmol, 1.00 equiv.) was dissolved in Me_2SiClH (2.00 mL) and stirred for 3 h at room temperature. All volatile components were removed under reduced pressure and the residue was washed with a minimum amount of pentane (200 µL). After drying under reduced pressure, the product (68.0 mg, 82%) was obtained as a white powder.

^{1}H NMR (600 MHz, Tol-d_8) δ 6.79 – 6.70 (m, 3H), 6.45 (app t, *J* = 7.7 Hz, 6H). **^{13}C NMR** (151 MHz, Tol-d_8) δ 165.9 (dd, *J* = 250.3, 11.9 Hz), 135.1 (t, *J* = 11.2 Hz), 111.7 (dd, *J* = 23.6, 4.4 Hz).

4.3.3 1c–H – tetrabutylammonium tris(2,6-difluorophenyl)hydroborate

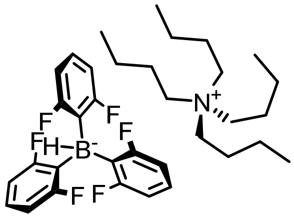

Synthesis of $H–B(2,6-F_2C_6H_3)_3$•NBu_4 was performed according to literature procedure. All spectroscopic data were in accordance with those reported in the literature.[15]

^{1}H NMR (600 MHz, $CDCl_3$) δ 6.99 – 6.90 (m, 1H), 6.90 – 6.82 (m, 2H), 6.55 (dt, *J* = 8.4, 7.0 Hz, 6H), 3.68 (1:1:1:1 q*, *J* = 167.8, 77.1 Hz, 1H), 3.06 – 2.98 (m, 8H), 1.54 – 1.42 (m, 8H), 1.31 – 1.22 (m, 8H), 0.90 (t, *J* = 7.4 Hz, 12H); *multiplicity and intensity resulting from coupling with ^{11}B (I = 3/2); **^{13}C NMR** (151 MHz, $CDCl_3$) δ 168.5 – 165.9 (m), 124.2 (t, *J* = 10.6 Hz), 110.1 (d, *J* = 30.8 Hz), 109.4 (d, *J* = 31.1 Hz), 58.7 (s), 24.0 (s), 19.6 (s), 13.7 (s).

4.4 Inverse hydride shuttle catalysis

4.4.1 General procedure

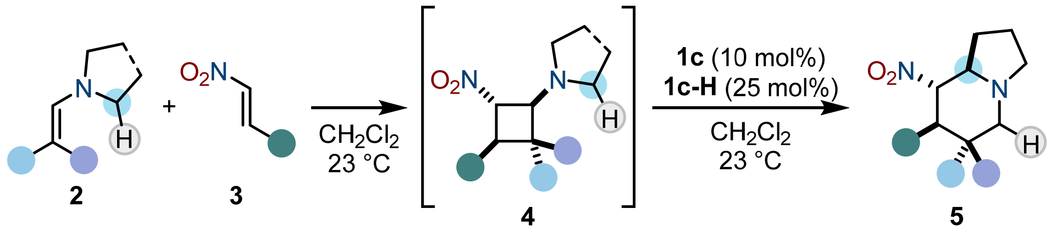

To a 4.00 mL vial containing enamine **2** (250 µmol, 1.00 equiv.) was added a solution of nitrostyrene **3** in CH_2Cl_2 (0.70 mL of a 2.8 M solution, 250 µmol, 1.00 equiv.) at room temperature (23 °C) and the solution was stirred for 1–3 h.* Over the course of 20 min, the solution was added to a solution of Lewis acid **1c** (10 mol%) and its hydride **1c–H** (25 mol%) in CH_2Cl_2 (200 µL) using a syringe pump. After the addition was complete, the reaction was stirred for 1 h at room temperature before the solvent was removed under reduced pressure. Analysis of the crude mixture by ^{1}H NMR showed the formation of a single diastereomer unless stated otherwise. The residue was purified by flash column chromatography (heptane/CH_2Cl_2 1:1 to CH_2Cl_2 unless stated otherwise) to afford the final product.

* As judged by ^{1}H-NMR, the cyclobutane (for a characterisation of representative cyclobutane **4a**, *vide infra*) forms during this time. The corresponding cyclobutanes are formed as single diastereomer unless stated otherwise.

4.4.2 4a – 1-(2,2-dimethyl-4-nitro-3-phenylcyclobutyl)pyrrolidine[16]

Represenative cyclobutane characterisation: Obtained upon mixing equimolar amounts of 1-(2-methylprop-1-en-1-yl)pyrrolidine and (*E*)-ß-nitrostyrene in CH_2Cl_2 at 23 °C. After 1 hour, an aliquot was withdrawn, concentrated in vacuo, redissolved in $CDCl_3$ and analysed by ^{1}H-NMR. The cyclobutanes are fleeting intermediates and prone to hydrolysis and other side reactions depending on their substitution pattern.

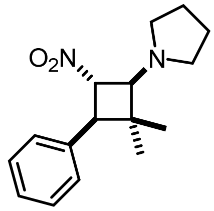

^{1}H NMR (400 MHz, $CDCl_3$) δ 7.37 – 7.32 (m, 2H), 7.30 – 7.23 (m, 1H), 7.17 (d, *J* = 7.3 Hz, 2H), 5.14 (dd, *J* = 9.7, 7.7 Hz, 1H), 3.58 (d, *J* = 9.6 Hz, 1H), 3.10 (d, *J* = 7.6 Hz, 1H), 2.61 – 2.46 (m, 4H), 1.81 – 1.75 (m, 4H), 1.40 (s, 3H), 0.76 (s, 3H).

4.4.3 5a – 6,6-dimethyl-8-nitro-7-phenyloctahydroindolizine

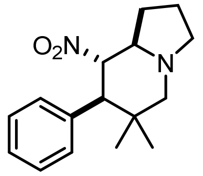

Performed on a 0.250 mmol scale using 10 mol% of **1c** (8.70 mg) and 25 mol% of **1c–H** (37.1 mg) delivering the product in 99% yield (68.4 mg) as a white crystalline solid.

^{1}H NMR (600 MHz, $CDCl_3$) δ 7.40 – 7.25 (m, 3H), 7.18 (d br, *J* = 6.2 Hz, 2H), 4.91 (dd, *J* = 11.6, 9.5 Hz, 1H), 3.13 (t, *J* = 8.2 Hz, 1H), 3.05 (d, *J* = 11.7 Hz, 1H), 2.83 (d, *J* = 11.0 Hz, 1H), 2.45 (dd, *J* = 15.6, 9.2 Hz, 1H), 2.28 (q, *J* = 8.7 Hz, 1H), 2.18 (d, *J* = 11.0 Hz, 1H), 2.05 – 1.83 (m, 2H), 1.78 (ddd, *J* = 13.2, 9.6, 3.4 Hz, 2H), 1.00 (s, 3H), 0.85 (s, 3H); **^{13}C NMR** (151 MHz, $CDCl_3$) δ 135.7, 128.6 (2C, HSQC), 128.0 (2C), 127.4, 90.3, 68.1, 65.6, 56.3, 54.0, 36.5, 28.2, 27.2, 21.8, 20.9; **HRMS (ESI$^+$)** exact mass calculated for $[M+H]^+$ ($C_{16}H_{23}N_2O_2^+$) requires *m/z* 275.1760, found *m/z* 275.1759; **IR** (thin film) ν 3032, 2963, 2797, 1547, 1266 cm^{-1}.

4.4.4 5b – 3,3-dimethyl-1-nitro-2-phenyloctahydro-*2H*-quinolizine

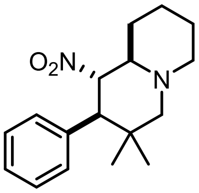

Performed on a 0.250 mmol scale using 30 mol% of **1c** (26.3 mg) and 30 mol% of **1c–H** (44.5 mg) delivering the product in 95% yield (68.5 mg) as a white crystalline solid.

^{1}H NMR (600 MHz, $CDCl_3$) δ 7.35-7.21 (m, 3H), 7.15 (s br, 2H), 4.88 (dd, *J* = 12.1, 9.4 Hz, 1H), 3.04 (d, *J* = 12.1 Hz, 1H), 2.84 (dt, *J* = 11.2, 3.9 Hz, 1H), 2.51 (d, *J* = 11.5 Hz, 1H), 2.32 (td, *J* = 10.7, 2.7 Hz, 1H), 2.19 (d, *J* = 11.5 Hz, 1H), 2.08 (td, *J* = 11.6, 3.4 Hz, 1H), 1.82 – 1.76 (m, 1H), 1.61 – 1.55 (m, 3H), 1.43 (tdd, *J* = 13.1, 10.8, 3.9 Hz, 1H), 1.29 – 1.20 (m, 1H), 0.98 (s, 3H), 0.78 (s, 3H); **^{13}C NMR** (151 MHz, $CDCl_3$) δ 135.7, 128.5 (2C, HSQC), 128.1 (2C), 127.6, 91.8, 69.9, 65.8, 56.4, 56.2, 35.1, 29.2, 27.5, 25.4, 23.6, 21.3; **HRMS (ESI$^+$)**: exact mass calculated for $[M+H]^+$ ($C_{17}H_{25}N_2O_2^+$) requires *m/z* 289.1911, found *m/z* 289.1912; **IR** (thin film) ν 3055, 2984, 2944, 2766, 2358, 1550, 1497, 1264, 1172, 1135, 1066, 731, 702 cm^{-1}.

4.4.5 5c – 3,3-dimethyl-1-nitro-2-phenyldecahydropyrido[1,2-a]azepine

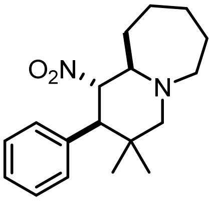

Performed on a 0.250 mmol scale using 30 mol% of **1c** (26.3 mg) and 30 mol% of **1c–H** (44.5 mg) delivering the product in 90% yield (68.2 mg) as a white crystalline solid.

^{1}H NMR (600 MHz, $CDCl_3$) δ 7.30 – 7.26 (m, 2H), 7.25 – 7.21 (m, 1H), 7.16 (s br, 2H), 4.95 (dd, *J* = 11.8, 9.4 Hz, 1H), 3.00 (d, *J* = 11.9 Hz, 1H), 2.87 (dt, *J* = 9.4, 4.7 Hz, 1H), 2.79 – 2.77 (m, 2H), 2.75 – 2.765 (m, 2H), 2.58 (d, *J* = 11.6 Hz, 1H), 1.84 – 1.73 (m, 1H), 1.71 – 1.60 (m, 4H), 1.60 – 1.49 (m, 1H), 1.49 – 1.39 (m, 1H), 0.97 (s, 3H), 0.75 (s, 3H); **^{13}C NMR** (151 MHz, $CDCl_3$) δ 136.0, 128.5 (2C, HSQC), 128.1 (2C), 127.6, 90.2, 70.6, 67.3, 57.0, 55.3, 35.8, 29.9, 29.3, 29.1, 27.0, 23.8, 21.4; **HRMS (ESI$^+$)**: exact mass calculated for $[M+H]^+$ ($C_{18}H_{27}N_2O_2^+$) requires *m/z* 303.2067, found *m/z* 303.2069; **IR** (thin film) ν 2925, 2853, 1543, 1496, 1469, 1156, 1134, 748, 720 cm^{-1}.

4.4.6 5d – 3,3-dimethyl-1-nitro-2-phenyldodecahydropyrido[2,1-a]isoindole

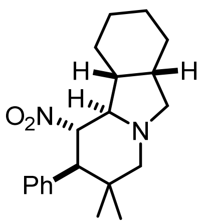

Performed on a 0.150 mmol scale using 30 mol% of **1c** (15.8 mg) and 30 mol% of **1c–H** (26.7 mg) delivering the product in 43% yield (21.1 mg) as a yellow oil.

^{1}H NMR (400 MHz, $CDCl_3$) δ 7.35 – 7.20 (m, 3H), 7.15 (s br, 2H), 4.89 (dd, *J* = 11.7, 9.2 Hz, 1H), 3.23 (dd, *J* = 8.7, 7.4 Hz, 1H), 3.01 (d, *J* = 11.7 Hz, 1H), 2.75 (d, *J* = 11.2 Hz, 1H), 2.51 (t, *J* = 8.7 Hz, 1H), 2.28 (d, *J* = 11.1 Hz, 1H), 2.25 – 2.18 (m, 1H), 2.16 – 2.10 (m, 1H), 2.08 (dd, *J* = 8.7, 7.0 Hz, 1H), 1.65 – 1.52 (m, 2H), 1.47 – 1.19 (m, 6H), 0.95 (s, 3H), 0.79 (s, 3H); **^{13}C NMR** (101 MHz, $CDCl_3$) δ 135.6, 128.3 (2C, HSQC), 128.0 (2C), 127.4, 89.8, 70.6, 65.4, 59.3, 57.1, 42.0, 36.4, 35.6, 27.1, 26.6, 23.9, 21.7, 21.5, 20.7; **HRMS (ESI$^+$):** exact mass calculated for $[M+H]^+$ ($C_{20}H_{29}N_2O_2^+$) requires *m/z* 329.2224, found *m/z* 329.2226; **IR** (thin film) ν 2928, 1680, 1547, 1452, 1368, 1264, 736, 702 cm^{-1}.

4.4.7 5e – 3,3-dimethyl-1-nitro-2-phenyl-1,3,4,6,11,11a-hexahydro-2H-pyrido[1,2-b]isoquinoline

The observed regioselectivity between **5e** and **5e'** was found to be dependent on the catalytic loading. Higher loadings of catalysts led to increased regiomeric ratios in favor of the linear fused ring system **5e**.

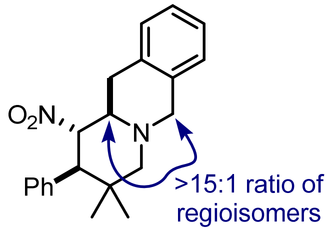

Performed on a 0.100 mmol scale using 50 mol% of **1c** (17.5 mg) and 50 mol% of **1c–H** (29.7 mg) delivering the product in 64% yield (21.4 mg) as an off-white crystalline solid (analysis of the crude mixture by ^{1}H NMR showed a ratio of regioisomers of >15:1). Eluent for purification by flash column chromatography (toluene/CH_2Cl_2 100:0 to 50:50).

Major regioisomer: **^{1}H NMR** (600 MHz, $CDCl_3$) δ 7.36 – 7.26 (m, 3H), 7.20 (s br, 2H), 7.18 – 7.12 (m, 2H), 7.11 – 7.06 (m, 1H), 7.06 – 7.02 (m, 1H), 5.04 (dd, *J* = 12.2, 9.1 Hz, 1H), 3.96 (d, *J* = 15.1 Hz, 1H), 3.51 (d, *J* = 15.0 Hz, 1H), 3.17 (d, *J* = 12.2 Hz, 1H), 3.05 (dd, *J* = 15.7, 9.9 Hz, 1H), 2.91 – 2.80 (m, 3H), 2.34 (d, *J* = 11.5 Hz, 1H), 1.06 (s, 3H), 0.85 (s, 3H); **^{13}C NMR** (151 MHz, $CDCl_3$) δ 135.1, 133.1, 131.5, 128.2, 128.1 (2C), 127.8 (2C, HSQC), 127.7, 126.7, 126.2, 125.8, 92.1, 69.1, 61.8, 57.6, 55.7, 35.0, 33.1, 27.4, 20.8; **HRMS (ESI$^+$):** exact mass calculated for

[M+H]$^+$ ($C_{21}H_{25}N_2O_2^+$) requires *m/z* 337.1911, found *m/z* 337.1905; **IR** (thin film) ν 2968, 1654, 1550, 1455, 1365, 748, 730 cm^{-1}.

4.4.8 5e' – 3,3-dimethyl-1-nitro-2-phenyl-1,3,4,6,7,11b-hexahydro-2H-pyrido[2,1-a]isoquinoline

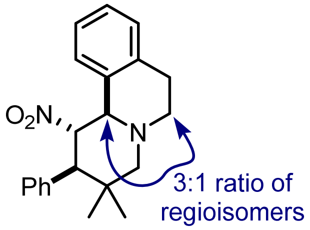

Performed on a 0.100 mmol scale using 15 mol% of **1c** (5.30 mg) and 15 mol% of **1c–H** (8.90 mg) delivering the product in 51% yield (17.1 mg) as a yellow crystalline solid (analysis of the crude mixture by ^{1}H NMR showed a ratio of regioisomers of 3:1). Eluent for purification by flash column chromatography (toluene/CH_2Cl_2 100:0 to 50:50).

Major regioisomer: **^{1}H NMR** (600 MHz, $CDCl_3$) δ 7.32 – 7.21 (m, 3H), 7.21 – 7.02 (m, 5H), 6.87 (d, *J* = 8.0 Hz, 1H), 5.01 (dd, *J* = 11.7, 9.1 Hz, 1H), 4.16 (d, *J* = 9.0 Hz, 1H), 3.32 (d, *J* = 11.7 Hz, 1H), 3.15 (ddd, *J* = 15.3, 10.2, 4.8 Hz, 1H), 3.05 – 2.98 (m, 1H), 2.86 – 2.78 (m, 2H), 2.77 (d, *J* = 12.0 Hz, 1H), 2.71 (dt, *J* = 15.5, 3.2 Hz, 1H), 1.02 (s, 3H), 0.83 (s, 3H); **^{13}C NMR** (151 MHz, $CDCl_3$) δ 136.1, 135.5, 133.1, 129.4, 128.1 (2C, HSQC), 128.1 (2C), 127.7, 127.4, 126.5, 126.0, 90.7, 69.2, 66.2, 58.8, 50.8, 35.9, 31.2, 27.1, 22.2; **HRMS (ESI$^+$):** exact mass calculated for [M+H]$^+$ ($C_{21}H_{25}N_2O_2^+$) requires *m/z* 337.1911, found *m/z* 337.1912; **IR** (thin film) ν 2963, 1547, 1370, 1147, 731, 703 cm^{-1}.

4.4.9 5f – 8-((tert-butyldimethylsilyl)oxy)-3,3-dimethyl-1-nitro-2-phenyloctahydro-2H-quinolizine

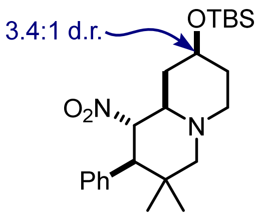

Performed on a 0.150 mmol scale using 30 mol% of **1c** (15.8 mg) and 30 mol% of **1c–H** (26.7 mg) delivering the product in 70% yield (43.9 mg) as a white crystalline solid (analysis of the crude mixture by ^{1}H NMR showed a d.r. of 3.4:1). Eluent for purification by flash column chromatography (toluene/CH_2Cl_2/*i*-PrOH 50:50:0 to 0:95:5).

Major diastereomer: **^{1}H NMR** (600 MHz, $CDCl_3$) δ 7.32 – 7.22 (m, 3H), 7.20 – 7.08 (s br, 2H), 4.89 (dd, *J* = 12.0, 9.4 Hz, 1H), 3.60 – 3.51 (m, 1H), 2.99 (d, *J* = 12.1 Hz, 1H), 2.84 (dt, *J* = 11.6, 3.4 Hz, 1H), 2.55 (d, *J* = 11.5 Hz, 1H), 2.46 – 2.34 (m, 1H), 2.21 – 2.07 (m, 2H), 1.87 – 1.79 (m, 1H), 1.74 – 1.69 (m, 1H), 1.69 – 1.61 (m, 1H), 1.50 (dd, *J* = 23.3, 11.1 Hz, 1H), 0.97 (s, 3H), 0.86

(s, 9H), 0.77 (s, 3H), 0.04 (s, 3H), 0.03 (s, 3H); **^{13}C NMR** (151 MHz, $CDCl_3$) δ 135.3, 128.0 (2C, HSQC), 128.0 (2C), 127.6, 91.1, 68.8, 68.7, 63.6, 56.0, 53.9, 38.5, 35.1, 35.0, 27.3, 25.8 (3C), 21.1, 18.0, -4.6, -4.6; **HRMS (ESI$^+$):** exact mass calculated for $[M+H]^+$ ($C_{23}H_{39}N_2O_3Si^+$) requires *m/z* 419.2724, found *m/z* 419.2722; **IR** (thin film) ν 2951, 2929, 1550, 1255, 1102, 837, 776 cm^{-1}.

4.4.10 5g – 3,3,6-trimethyl-1-nitro-2-phenyloctahydro-2H-quinolizine

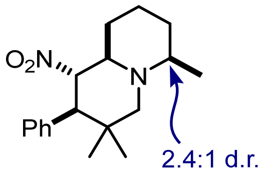

Performed on a 0.300 mmol scale using 50 mol% of **1c** (52.5 mg) and 50 mol% of **1c–H** (72.2 mg) delivering the product in 58% yield (52.7 mg) as a white crystalline solid (analysis of the crude mixture by ^{1}H NMR showed a d.r. of 2.4:1). Before inverse hydride shuttle, the respective cyclobutane was formed as a mixture of two diastereomers in a ratio of 2.5:1 (measured after 2 h).

^{1}H NMR (600 MHz, $CDCl_3$) δ 7.32 – 7.21 (m, 3.8H), 7.15 (s br, 2.5H), 4.88 (dd, *J* = 12.1, 9.4 Hz, 1.0H), 4.80 (dd, J = 12.2, 9.1 Hz, 0.3H), 3.12 – 3.07 (m, 0.3H), 3.05 (d, *J* = 12.1 Hz, 3.0H), 2.96 (d, *J* = 12.2 Hz, 0.3H), 2.96 (d, *J* = 11.5 Hz, 1.0H), 2.88 – 2.81 (m, 0.3H), 2.54 (d, *J* = 11.5 Hz, 0.3H), 2.47 – 2.41 (m, 1.0H), 2.32 (d, *J* = 11.5 Hz, 0.3H), 2.13 – 2.06 (m, 1.0H), 1.93 (d, *J* = 11.5 Hz, 1.0H), 1.84 – 1.77 (m, 0.3H), 1.76 – 1.70 (m, 1.0H), 1.68 – 1.60 (m, 1.3H), 1.60 – 1.50 (m, 1.8H), 1.49 – 1.39 (m, 1.0H), 1.39 – 1.24 (m, 2.3H), 1.11 (d, *J* = 6.0 Hz, 3.0H), 1.03 (d, *J* = 6.7 Hz, 0.8H), 0.97 (s, 0.8H), 0.95 (s, 3.0H), 0.79 (s, 3.0H), 0.77 (s, 0.8H); Major diastereomer: **^{13}C NMR** (151 MHz, $CDCl_3$) δ 135.7, 127.9 (2C), 127.9 (2C, HSQC), 127.5, 92.2, 65.5, 64.7, 57.4, 56.3, 34.8, 34.6, 29.5, 27.5, 23.0, 21.5, 21.2; Minor diastereomer: **^{13}C NMR** (151 MHz, $CDCl_3$) δ 135.6, 127.9 (2C), 127.9 (2C, HSQC), 127.4, 93.0, 65.9, 57.6, 56.1, 54.5, 34.9, 31.9, 30.0, 27.4, 20.6, 17.5, 9.4; **HRMS (ESI$^+$):** exact mass calculated for $[M+H]^+$ ($C_{18}H_{27}N_2O_2^+$) requires *m/z* 303.2067, found *m/z* 303.2065; **IR** (thin film) ν 2934, 1725, 1542, 1371, 1270, 1130, 1086, 749, 703 cm^{-1}.

4.4.11 5h – 1-ethyl-2,5,5-trimethyl-3-nitro-4-phenylpiperidine

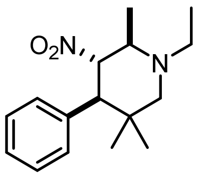

Performed on a 0.150 mmol scale using 40 mol% of **1c** (21.0 mg) and 40 mol% of **1c–H** (35.6 mg) delivering the product in 60% yield (24.7 mg) as a white crystalline solid.

^{1}H NMR (600 MHz, $CDCl_3$) δ 7.29 – 7.22 (m, 3H), 7.15 (s br, 2H), 4.83 (dd, *J* = 11.9, 9.4 Hz, 1H), 3.01 (d, *J* = 12.1 Hz, 1H), 2.90 – 2.76 (m, 1H), 2.71 (dq, *J* = 13.8, 6.9 Hz, 2H), 2.59 (d, *J* = 11.6 Hz, 1H), 2.39 (d, *J* = 11.6 Hz, 1H), 1.19 (d, *J* = 6.0 Hz, 3H), 1.01 (t, *J* = 7.1 Hz, 3H), 0.96 (s, 3H), 0.78 (s, 3H); **^{13}C NMR** (151 MHz, $CDCl_3$) δ 135.8, 128.4 (2C, HSQC), 128.1 (2C), 127.6, 92.8, 65.4, 60.0, 56.7, 46.7, 35.1, 27.4, 21.1, 16.4, 9.3; **HRMS (ESI$^+$):** exact mass calculated for $[M+H]^+$ ($C_{16}H_{25}N_2O_2^+$) requires 277.1991 *m/z*, found 277.1913 *m/z;* **IR** (thin film) ν 2962, 2925, 1549, 1450, 1372 cm^{-1}.

4.4.12 5i – 6-methyl-8-nitro-7-phenyl-6-propyloctahydroindolizine

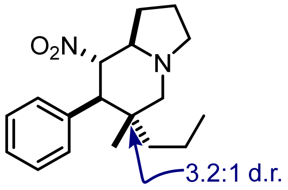

Performed on a 0.150 mmol scale using 50 mol% of **1c** (26.3 mg) and 50 mol% of **1c–H** (44.5 mg) delivering the product in 88% yield (40.1 mg) as a white crystalline solid (analysis of the crude mixture by ^{1}H NMR showed a d.r. of 3.2:1). Before inverse hydride shuttle, the respective cyclobutane was formed as a mixture of two diastereomers in a ratio of 3.6:1 (measured after 2 h).

Major diasteroisomer: **^{1}H NMR** (600 MHz, $CDCl_3$) δ 7.28 (t, *J* = 7.3 Hz, 2H), 7.26 – 7.21 (m, 1H), 7.17 (s, 2H), 4.91 (dd, *J* = 11.7, 9.4 Hz, 1H), 3.10 (td, *J* = 8.4, 1.7 Hz, 1H), 3.08 (d, *J* = 11.7 Hz, 1H), 2.90 (d, *J* = 11.0 Hz, 1H), 2.40 (td, *J* = 9.3, 6.5 Hz, 1H), 2.25 (q, *J* = 8.8 Hz, 1H), 2.13 (d, *J* = 11.0 Hz, 1H), 1.97 – 1.81 (m, 2H), 1.81 – 1.64 (m, 2H), 1.35 – 1.23 (m, 1H), 1.22 – 1.04 (m, 3H), 1.00 (s, 3H), 0.82 (t, *J* = 7.0 Hz, 3H); **^{13}C NMR** (151 MHz, $CDCl_3$) δ 136.0, 128.2 (2C), 128.0 (2C, HSQC), 127.6, 90.9, 68.1, 63.2, 55.9, 54.5, 42.7, 39.3, 28.4, 21.1, 20.1, 16.4, 14.9, **HRMS (ESI$^+$):** exact mass calculated for $[M+H]^+$ ($C_{18}H_{27}N_2O_2^+$) requires 303.2067 *m/z*, found 303.2064 *m/z;* **IR** (thin film) ν 2958, 2872, 2796, 1545, 1496, 1454, 1357,732, 701 cm^{-1}.

4.4.13 5j – 6-methyl-6-(4-methylpent-3-en-1-yl)-8-nitro-7-phenyloctahydroindolizine

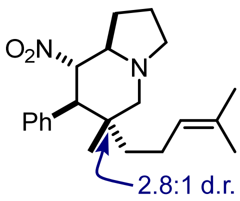

Performed on a 0.200 scale using 30 mol% of **1c** (21.0 mg) and 30 mol% of **1c–H** (35.6 mg) delivering the product in 94% (48.5 mg) as a white crystalline solid (analysis of the crude mixture by ^{1}H NMR showed a d.r. of 2.8:1). Before inverse hydride shuttle, the respective cyclobutane was formed as a mixture of two diastereomers in a ratio of 2.5:1 (measured after 1 h).

Major diastereomer: **^{1}H NMR** (600 MHz, $CDCl_3$) δ 7.28 (t, *J* = 7.3 Hz, 2H), 7.26 – 7.21 (m, 1H), 7.17 (s, 2H), 4.95 (t, *J* = 6.9 Hz, 1H), 4.91 (dd, *J* = 11.6, 9.4 Hz, 1H), 3.17 – 3.03 (m, 2H), 2.94 (d, *J* = 11.0 Hz, 1H), 2.45 – 2.37 (m, 1H), 2.26 (q, *J* = 8.7 Hz, 1H), 2.16 (d, *J* = 11.0 Hz, 1H), 1.96 – 1.72 (m, 6H), 1.64 (s, 3H), 1.55 (s, 3H), 1.23 – 1.16 (m, 1H), 1.16 – 1.09 (m, 1H), 1.02 (s, 3H); **^{13}C NMR** (151 MHz, $CDCl_3$) δ 135.7, 131.5, 128.0 (2C), 127.6 (2C, HSQC), 127.5, 124.2, 90.7, 67.9, 62.9, 55.8, 54.3, 40.1, 39.0, 28.3, 25.6, 21.7, 20.9, 19.7, 17.6; **HRMS (ESI^{+}):** exact mass calculated for $[M+H]^{+}$ ($C_{21}H_{31}N_2O_2^{+}$) requires *m/z* 343.2380, found *m/z* 343.2383; **IR** (thin film) ν 2976, 1550, 1456, 1359, 1274, 1268, 750 cm^{-1}.

4.4.14 5k – 6-isopropyl-8-nitro-7-phenyloctahydroindolizine

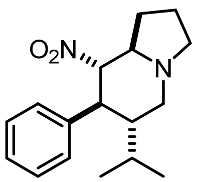

Performed on a 0.150 mmol scale using 10 mol% of **1c** (5.30 mg) and 25 mol% of **1c–H** (22.3 mg) and using a double syringe setup in which the solution of cyclobutane in the inner syringe was cooled to 0 °C by an ice/water mixture in the outer syringe during addition, delivering the product in 61% (26.2 mg) as a white crystalline solid. Eluent for purification by flash column chromatography (CH_2Cl_2/*i*-PrOH 100:0 to 95:5).

^{1}H NMR (400 MHz, $CDCl_3$) δ 7.29 (dd, *J* = 19.8, 12.2 Hz, 3H), 7.18 (d, *J* = 7.0 Hz, 2H), 4.50 (dd, *J* = 10.8, 9.6 Hz, 1H), 3.21 – 3.05 (m, 3H), 2.48 (td, *J* = 9.5, 6.1 Hz, 1H), 2.33 (q, *J* = 8.8 Hz, 1H), 2.17 – 1.99 (m, 2H), 1.99 – 1.85 (m, 2H), 1.84 – 1.76 (m, 1H), 1.72 – 1.65 (m, 1H), 1.43 (septd, *J* = 7.0, 2.8 Hz, 1H), 0.87 (d, *J* = 7.0 Hz, 3H), 0.80 (d, *J* = 7.0 Hz, 3H); **^{13}C NMR** (101 MHz, $CDCl_3$) δ 138.1, 128.9 (2C), 128.2 (br, 2C), 127.6, 95.0, 66.7, 54.2, 50.9, 50.6, 45.9, 28.0, 26.4, 21.5, 21.1, 15.8; **HRMS (ESI$^+$):** exact mass calculated for $[M+H]^+$ ($C_{17}H_{25}N_2O_2^+$) requires *m/z* 289.1911, found *m/z* 289.1912; **IR** (thin film) ν 2958, 2800, 1545, 1455, 762, 734, 699 cm^{-1}.

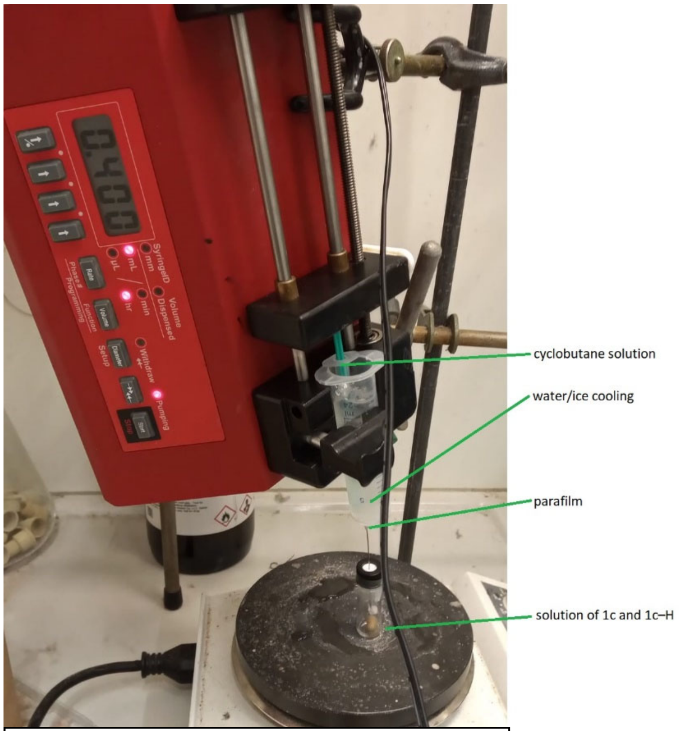

Figure S2. Double syringe pump setup for temperature controlled addition

4.4.15 5l – 8'-nitro-7'-phenylhexahydro-5'H-spiro[cyclohexane-1,6'-indolizine]

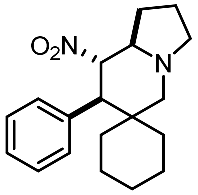

Performed on a 0.150 mmol scale using 30 mol% of **1c** (11.0 mg) and 30 mol% of **1c–H** (18.0 mg) delivering the product in 42% yield (13.3 mg) as a white crystalline solid.

^{1}H NMR (400 MHz, $CDCl_3$) δ 7.35 – 7.21 (m, 3H), 7.14 (s br, 2H), 4.92 (dd, *J* = 11.8, 9.4 Hz, 1H), 3.56 (d, *J* = 11.6 Hz, 1H), 3.12 (t, *J* = 8.7 Hz, 1H), 2.98 (d, *J* = 11.9 Hz, 1H), 2.42 (td, *J* = 9.2, 6.3 Hz, 1H), 2.29 (app q, *J* = 8.5 Hz, 1H), 1.98 – 1.67 (m, 6H), 1.55 (d, *J* = 9.9 Hz, 1H), 1.49 – 1.25 (m, 5H), 1.23 – 1.10 (m, 2H), 0.94 – 0.75 (m, 1H); **^{13}C NMR** (101 MHz, $CDCl_3$) δ 135.4, 132.9 (2C, HSQC), 128.0 (2C), 127.57, 90.1, 68.3, 58.2, 57.9, 54.4, 39.4, 35.3,

28.6, 27.4, 25.9, 21.6, 21.4, 21.1; **HRMS (ESI$^+$):** exact mass calculated for $[M+H]^+$ ($C_{19}H_{27}N_2O_2^+$) requires *m/z* 315.2067, found *m/z* 315.2066*;* **IR** (thin film) ν 2926, 2855, 2794, 1545, 1495, 1451, 1136, 1120,778, 730, 702 cm^{-1}.

4.4.16 5m – 6,6-dimethyl-8-nitro-7-(*p*-tolyl)octahydroindolizine

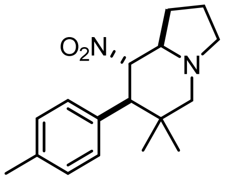

Performed on a 0.250 mmol scale using 10 mol% of **1c** (8.70 mg) and 25 mol% of **1c–H** (37.1 mg) affording the product in 93% yield (67.3 mg) as a white crystalline solid.

^{1}H NMR (600 MHz, $CDCl_3$) δ 7.08 (t, *J* = 14.0 Hz, 4H), 4.86 (dd, *J* = 11.6, 9.5 Hz, 1H), 3.10 (t, *J* = 8.6 Hz, 1H), 2.99 (d, *J* = 11.7 Hz, 1H), 2.80 (d, *J* = 11.0 Hz, 1H), 2.47 – 2.37 (m, 1H), 2.30 (s, 3H), 2.24 (q, *J* = 8.8 Hz, 1H), 2.14 (d, *J* = 11.0 Hz, 1H), 1.96 – 1.82 (m, 2H), 1.81 – 1.66 (m, 2H), 0.96 (s, 3H), 0.81 (s, 3H); **^{13}C NMR** (151 MHz, $CDCl_3$) δ 137.2, 132.7, 128.9 (2C), 128.7 (2C, HSQC), 90.5, 68.3, 65.7, 56.0, 54.2, 36.6, 28.3, 27.3, 21.9, 21.2, 21.0; **HRMS (ESI$^+$):** exact mass calculated for $[M+H]^+$ ($C_{17}H_{25}N_2O_2^+$) requires *m/z* 289.1911, found *m/z* 289.1910*;* **IR** (thin film) ν (cm^{-1}) 2957, 2875, 2804, 1537, 1331, 827 738, 561 cm^{-1}.

4.4.17 5n – 7-(4-methoxyphenyl)-6,6-dimethyl-8-nitrooctahydroindolizine

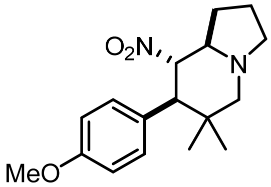

Performed on a 0.250 mmol scale using 10 mol% of **1c** (8.70 mg) and 25 mol% of **1c–H** (37.1 mg) affording the product in 82% yield (62.2 mg) as a colourless oil.

^{1}H NMR (600 MHz, $CDCl_3$) δ 7.07 (d, *J* = 6.6 Hz, 2H), 6.82 (d, *J* = 8.4 Hz, 2H), 4.83 (dd, *J* = 11.6, 9.4 Hz, 1H), 3.77 (s, 3H), 3.09 (t, *J* = 8.7 Hz, 1H), 2.96 (d, *J* = 11.8 Hz, 1H), 2.80 (d, *J* = 11.0 Hz, 1H), 2.40 (dd, *J* = 15.7, 9.2 Hz, 1H), 2.24 (app q, *J* = 8.7 Hz, 1H), 2.13 (d, *J* = 11.0 Hz, 1H), 1.95 – 1.80 (m, 2H), 1.80 – 1.66 (m, 2H), 0.95 (s, 3H), 0.80 (s, 3H); **^{13}C NMR** (151 MHz, $CDCl_3$) δ 158.9, 127.7, 113.5 (2C), 113.2 (2C, HSQC), 90.6, 68.3, 65.7, 55.6, 55.3, 54.2, 36.6, 28.3, 27.3, 21.9, 21.0; **HRMS (ESI+):** exact mass calculated for $[M+H]^+$ ($C_{17}H_{25}N_2O_3^+$) requires *m/z* 305.3975, found *m/z* 305.3960; **IR** (thin film) ν 2961, 2796, 1612, 1545, 1512, 1249, 833, 736, 564 cm^{-1}.

4.4.18 5o – 7-(2-(benzyloxy)phenyl)-6,6-dimethyl-8-nitrooctahydroindolizine

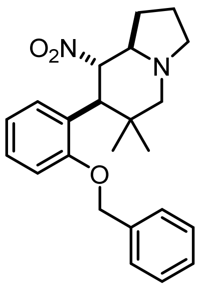

Performed on a 0.250 mmol scale using 10 mol% of **1c** (8.70 mg) and 25 mol% of **1c–H** (37.1 mg) affording the product in 62% yield (59.3 mg, 8:1 mixture of rotamers – cross-peaks detected by NOESY confirming isomerisation between the two rotamers, see NMR-Spectra) as a yellow oil.

Signals are assigned to the two rotamers as R1 and R2, respectively. **^{1}H NMR** (600 MHz, $CDCl_3$) δ 7.53 (d, *J* = 7.3 Hz, 2H, R2), 7.47 (d, *J* = 7.5 Hz, 2H, R1), 7.44 – 7.38 (m, 2H, R1+R2), 7.38 – 7.30 (m, 1H, R1+R2), 7.23 – 7.14 (m, 2H, R1+R2), 7.02 (dd, *J* = 7.6, 1.6 Hz, 1H, R2), 6.96 – 6.90 (m, 1H, R1+R2), 6.90 (d, *J* = 8.2 Hz, 1H, R1), 6.86 (td, *J* = 7.4, 1.0 Hz, 1H, R2), 5.65 (dd, *J* = 11.5, 9.5 Hz, 1H, R2), 5.07 (s, 2H, R1), 5.06 (s, 2H, R2), 4.84 (dd, *J* = 11.9, 9.3 Hz, 1H, R1), 3.95 (d, *J* = 12.0 Hz, 1H, R1), 3.10 (td, *J* = 8.6, 1.7 Hz, 1H, R1), 3.03 (td, *J* = 8.6, 2.1 Hz, 1H, R2), 2.96 (d, *J* = 11.5 Hz, 1H, R2), 2.78 (d, *J* = 11.0 Hz, 1H, R1), 2.72 (d, *J* = 11.0 Hz, 1H, R2), 2.46 (td, *J* = 9.3, 6.3 Hz, 1H, R1), 2.32 – 2.22 (m, 1H, R1+R2), 2.21 – 2.14 (m, 1H, R1+R2), 2.09 (d, *J* = 11.0 Hz, 1H, R2), 1.97 – 1.82 (m, 2H, R1+R2), 1.81 – 1.66 (m, 2H, R1+R2), 1.01 (s, 3H, R1), 0.96 (s, 3H, R2), 0.85 (s, 3H, R2), 0.82 (s, 3H, R1); **^{13}C NMR** (151 MHz, $CDCl_3$) δ 157.7 (R2), 157.4 (R1), 137.4 (R1), 137.0 (R2), 135.0 (R2), 128.8 (R2), 128.7 (R1), 128.6 (R2), 128.5 (R1), 128.2 (R1), 128.0 (R2), 127.8 (R2), 127.7 (R1), 127.1 (R1), 125.1 (R1), 123.5 (R2), 120.4 (R2), 120.3 (R1), 112.9 (R2), 112.6 (R1), 90.5 (R1), 88.7 (R2), 70.7 (R2), 70.6 (R1), 68.4 (R2), 68.1 (R1), 66.0 (R2), 65.7 (R1), 57.9 (R2), 54.1 (R2), 54.0 (R1), 46.2 (R1), 37.9 (R2), 37.2 (R1), 28.3 (R2), 28.2 (R1), 28.1 (R2), 26.7 (R1), 23.4 (R2), 22.1 (R1), 21.0 (R1), 20.9 (R2); **HRMS (ESI^{+}):** exact mass calculated for $[M+H]^{+}$ ($C_{23}H_{29}N_2O_3^{+}$) requires *m/z* 381.2173, found *m/z* 381.2166; **IR** (thin film) ν 2964, 1545, 1493, 1452, 1361, 1240, 1227, 1119, 754, 730 cm^{-1}.

4.4.19 5p – 7-(2,4-dimethoxyphenyl)-6,6-dimethyl-8-nitrooctahydroindolizine

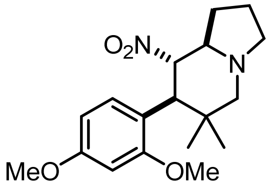

Performed on a 0.250 mmol scale using 10 mol% of **1c** (8.70 mg) and 25 mol% of **1c–H** (37.1 mg) affording the product in 79% yield (66.2 mg, 7:1 mixture of rotamers – cross-peaks detected by NOESY confirming isomerisation between the two rotamers, see NMR-Spectra) as a white crystalline solid.

Signals are assigned to the two rotamers as R1 and R2 respectively. **^{1}H NMR** (700 MHz, $CDCl_3$) δ 7.05 (d, *J* = 8.4 Hz, 1H, R1), 6.89 (d, *J* = 8.4 Hz, 1H, R2), 6.45 (dd, *J* = 8.4, 2.5 Hz, 1H, R1), 6.43 – 6.42 (m, 1H, R1+R2), 6.38 (dd, *J* = 8.3, 2.5 Hz, 1H, R2), 5.57 (dd, *J* = 11.5, 9.5 Hz, 1H, R2), 4.77 (dd, *J* = 12.0, 9.3 Hz, 1H, R1), 3.77 (s, 6H, R1+R2), 3.72 (d, *J* = 12.0 Hz, 1H, R1), 3.13 – 3.05 (m, 1H, R1+R2), 2.87 (d, *J* = 11.5 Hz, 1H, R2), 2.77 (d, *J* = 11.0 Hz, 1H, R1+R2), 2.43 (td, *J* = 9.3, 6.4 Hz, 1H, R1), 2.30 (td, *J* = 9.4, 6.3 Hz, 1H, R2), 2.24 (app q, *J* = 8.7 Hz, 1H, R1), 2.21 – 2.17 (m, *J* = 6.7 Hz, 1H, R1+R2), 2.08 (d, *J* = 11.0 Hz, 1H, R2), 1.94 – 1.81 (m, 2H, R1+R2), 1.79 – 1.69 (m, 2H, R1+R2), 0.98 (s, 1H, R2), 0.96 (s, 3H, R1), 0.83 (s, 1H, R2), 0.77 (s, 3H, R1); **^{13}C NMR** (176 MHz, $CDCl_3$) δ 160.3 (R2), 159.7 (R1), 159.2 (R2), 159.2 (R1), 135.1 (R2), 129.0 (R1), 117.0 (R1), 115.8 (R2), 104.1 (R2), 103.9 (R1), 99.5 (R2), 98.8 (R1), 90.6 (R1), 88.9 (R2), 68.7 (R2), 68.1 (R1), 66.2 (R2), 65.6 (R1), 57.3 (R2), 55.6 (R1), 55.2 (R2), 55.2 (R1), 55.0 (R2), 54.3 (R2), 54.0 (R1), 45.7 (R1), 37.8 (R2), 37.1 (R1), 28.2 (R1), 28.2 (R2), 28.1 (R2), 26.5 (R1), 23.2 (R2), 22.0 (R1), 21.0 (R1+R2); **HRMS (ESI$^+$):** exact mass calculated for $[M+H]^+$ ($C_{18}H_{27}N_2O_4^+$) requires *m/z* 335.1965, found *m/z* 335.1962; **IR** (thin film) ν 2962, 1612, 1547, 1508, 1208, 1124, 1036 cm^{-1}.

4.4.20 5q – 6,6-dimethyl-8-nitro-7-(4-nitrophenyl)octahydroindolizine

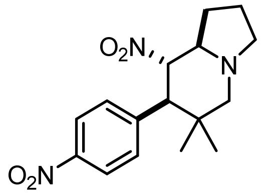

Performed on a 0.250 mmol scale using 10 mol% of **1c** (8.70 mg) and 25 mol% of **1c–H** (37.1 mg) affording the product in 87% yield (69.2 mg) as a yellow crystalline solid.

^{1}H NMR (600 MHz, $CDCl_3$) δ 8.17 (d, *J* = 8.3 Hz, 2H), 7.35 (d, *J* = 6.3 Hz, 2H), 4.88 (dd, *J* = 11.4, 9.6 Hz, 1H), 3.17 (d, *J* = 11.7 Hz, 1H), 3.11 (t, *J* = 8.6 Hz, 1H), 2.83 (d, *J* = 11.1 Hz, 1H), 2.43 (dd, *J* = 15.4, 9.0 Hz, 1H), 2.27 (app q, *J* = 8.6 Hz, 1H), 2.19 (d, *J* = 11.1 Hz, 1H), 2.00 – 1.84 (m, 2H), 1.84 – 1.68 (m, 2H), 0.97 (s, 3H), 0.83 (s, 3H); **^{13}C NMR** (151 MHz, $CDCl_3$) δ 147.5, 143.7, 132.6 (2C, HSQC), 123.4 (2C), 89.9, 67.9, 65.4, 56.3, 54.0, 36.9, 28.3, 27.2, 21.8, 21.0; **HRMS (ESI$^+$):** exact mass calculated for $[M+H]^+$ ($C_{16}H_{22}N_3O_4^+$) requires *m/z* 320.1605, found *m/z* 320.1604; **IR** (thin film) ν 2956, 2797, 1604, 1545, 1518, 1494, 1394, 1224, 1119, 749, 734, 712 cm^{-1}.

4.4.21 5r – 7-(4-fluorophenyl)-6,6-dimethyl-8-nitrooctahydroindolizine

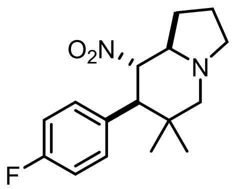

Performed on a 0.250 mmol scale using 10 mol% of **1c** (8.70 mg) and 25 mol% of **1c–H** (37.1 mg) affording the product in 87% yield (63.5 mg) as a white crystalline solid.

^{1}H NMR (600 MHz, $CDCl_3$) δ 7.13 (s, 2H), 6.98 (t, *J* = 8.4 Hz, 2H), 4.82 (dd, *J* = 11.7, 9.4 Hz, 1H), 3.09 (t, *J* = 8.9 Hz, 1H), 3.01 (d, *J* = 11.8 Hz, 1H), 2.80 (d, *J* = 11.1 Hz, 1H), 2.41 (dd, *J* = 15.7, 9.2 Hz, 1H), 2.24 (app q, *J* = 8.7 Hz, 1H), 2.14 (d, *J* = 11.1 Hz, 1H), 1.94 – 1.82 (m, 2H), 1.80 – 1.70 (m, 2H), 0.95 (s, 3H), 0.80 (s, 3H); **^{13}C NMR** (151 MHz, $CDCl_3$) δ 162.3 (d, *J* = 246.1 Hz), 131.5 (d, *J* = 3.3 Hz), 115.1 (d, *J* = 21.2 Hz, 2C), 114.7 (2C, HSQC), 90.4, 68.2, 65.6, 55.7, 54.1, 36.6, 28.3, 27.2, 21.8, 21.0; **^{19}F NMR** (565 MHz, $CDCl_3$) δ -115.08 (m); **HRMS (ESI$^+$):** exact mass calculated for $[M+H]^+$ ($C_{16}H_{22}FN_2O_2^+$) requires *m/z* 293.1660, found *m/z* 293.1661; **IR** (thin film) ν (cm^{-1}) 2964, 2797, 1606, 1545, 1223, 873, 736, 561 cm^{-1}.

4.4.22 5s – 7-(4-chlorophenyl)-6,6-dimethyl-8-nitrooctahydroindolizine

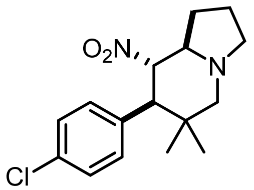

Performed on a 0.250 mmol scale using 10 mol% of **1c** (8.70 mg) and 25 mol% of **1c–H** (37.1 mg) affording the product in 71% yield (54.5 mg) as a white crystalline solid.

^{1}H NMR (700 MHz, $CDCl_3$) δ 7.31 – 7.23 (m, *J* = 7.9 Hz, 2H), 7.10 (d, *J* = 7.4 Hz, 2H), 4.82 (dd, *J* = 11.7, 9.3 Hz, 1H), 3.09 (td, *J* = 8.4, 1.7 Hz, 1H), 3.01 (d, *J* = 11.7 Hz, 1H), 2.80 (d, *J* = 11.1 Hz, 1H), 2.41 (td, *J* = 9.3, 6.6 Hz, 1H), 2.25 (app q, *J* = 8.7 Hz, 1H), 2.14 (d, *J* = 11.1 Hz, 1H), 1.95 – 1.82 (m, 2H), 1.80 – 1.69 (m, 2H), 0.95 (s, 3H), 0.81 (s, 3H); **^{13}C NMR** (176 MHz, $CDCl_3$) δ 134.3, 133.5, 128.3 (2C), 128.1 (2C, HSQC), 90.2, 68.0, 65.5, 55.8, 54.0, 36.5, 28.2, 27.1, 21.7, 20.9; **HRMS (ESI$^+$):** exact mass calculated for $[M+H]^+$ ($C_{16}H_{22}N_2O_2Cl^+$) requires *m/z* 309.1364, found *m/z* 309.1357; **IR** (thin film) ν 2962, 1545, 1491, 1366, 1088, 1013, 826, 737 cm^{-1}.

4.4.23 5t – 7-(2-bromophenyl)-6,6-dimethyl-8-nitrooctahydroindolizine

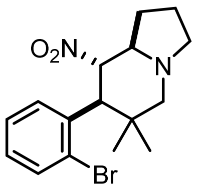

Performed on a 0.250 mmol scale using 10 mol% of **1c** (8.70 mg) and 25 mol% of **1c–H** (37.1 mg) affording the product in 93% yield (81.7 mg) as a white crystalline solid.

^{1}H NMR (600 MHz, $CDCl_3$) δ 7.59 – 7.55 (m, 1H), 7.30 – 7.24 (m, 2H), 7.09 (m, 1H), 4.79 (dd, *J* = 11.7, 9.3 Hz, 1H), 3.88 (d, *J* = 11.7 Hz, 1H), 3.11 (td, *J* = 8.5, 1.6 Hz, 1H), 2.80 (d, *J* = 11.1 Hz, 1H), 2.49 (td, *J* = 9.2, 6.3 Hz, 1H), 2.30 – 2.25 (m, 2H), 1.95 – 1.86 (m, 2H), 1.82 – 1.67 (m, 2H), 1.04 (s, 3H), 0.92 (s, 3H); **^{13}C NMR** (151 MHz, $CDCl_3$) δ 135.6, 133.7, 129.4, 128.9, 127.5, 127.1, 90.9, 68.1, 65.9, 54.1, 52.9, 38.1, 28.4, 26.5, 22.6, 21.1; **HRMS (ESI$^+$):** exact mass calculated for $[M+H]^+$ ($C_{16}H_{22}N_2O_2Br^+$) requires *m/z* 353.0859, found *m/z* 353.0857; **IR** (thin film) ν 2963, 2932, 2799, 1544, 1394, 1264, 1215, 1023, 940, 735 cm^{-1}.

4.4.24 5u – tert-butyl 2-(6,6-dimethyl-8-nitrooctahydroindolizin-7-yl)-1H-pyrrole-1-carboxylate

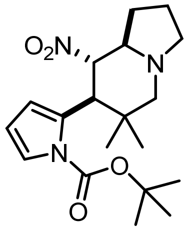

Performed on a 87 μmol scale using 10 mol% of **1c** (3.00mg) and 25 mol% of **1c–H** (12.0 mg) affording the product in 72% yield (21.0 mg) as an off-white crystalline solid.

^{1}H NMR (600 MHz, $CDCl_3$) δ 7.18 (dd, *J* = 3.2, 1.8 Hz, 1H), 6.13 – 6.05 (m, 2H), 4.63 – 4.56 (m, 2H), 3.07 (m, 1H), 2.78 (d, *J* = 11.0 Hz, 1H), 2.44 – 2.34 (m, 1H), 2.22 (m, 1H), 2.17 (d, *J* = 11.0 Hz, 1H), 1.97 – 1.78 (m, 2H), 1.78 – 1.68 (m, 2H), 1.61 (s, 9H), 0.97 (s, 3H), 0.85 (s, 3H); **^{13}C NMR** (151 MHz, $CDCl_3$) δ 149.7, 130.7, 122.5, 113.6, 109.8, 91.5, 84.0, 68.3, 65.2, 54.1, 45.9, 37.3, 28.4, 28.2, 28.1, 26.1, 22.5, 21.1; **HRMS (ESI$^+$):** exact mass calculated for $[M+H]^+$ ($C_{19}H_{30}N_3O_4^+$) requires *m/z* 364.2231, found *m/z* 364.2229; **IR** (thin film) ν 2970, 2932, 2794, 1742, 1547, 1314, 1147, 1119, 769 cm^{-1}.

4.4.25 5v – 6,6-dimethyl-8-nitro-7-(thiophen-2-yl)octahydroindolizine

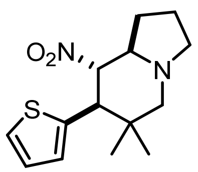

Performed on a 0.250 mmol scale using 10 mol% of **1c** (8.70 mg) and 25 mol% of **1c–H** (37.1 mg) affording the product in 69% yield (48.3 mg) as an off-white crystalline solid.

^{1}H NMR (600 MHz, $CDCl_3$) δ 7.19 (d, *J* = 5.1 Hz, 1H), 6.94 (dd, *J* = 5.1, 3.6 Hz, 1H), 6.85 (d, *J* = 3.2 Hz, 1H), 4.71 (dd, *J* = 11.5, 9.4 Hz, 1H), 3.32 (d, *J* = 11.6 Hz, 1H), 3.19 – 2.97 (m, 1H), 2.82 (d, *J* = 11.1 Hz, 1H), 2.45 – 2.32 (m, 1H), 2.24 (app q, *J* = 8.7 Hz, 1H), 2.13 (d, *J* = 11.1 Hz, 1H), 1.97–1.81 (m, 2H), 1.80 – 1.65 (m, 2H), 1.06 (s, 3H), 0.91 (s, 3H); **^{13}C NMR** (151 MHz, $CDCl_3$) δ 138.0, 127.1, 126.7, 124.7, 91.9, 68.3, 65.3, 54.1, 51.8, 36.6, 28.3, 27.5, 22.1, 21.0.; **HRMS (ESI$^+$):** exact mass calculated for $[M+H]^+$ ($C_{14}H_{21}N_2O_2S^+$) requires *m/z* 281.1318, found *m/z* 281.1316; **IR** (thin film) ν 2965, 2877, 2796, 1547, 1464, 1393, 1363, 832, 763, 749, 699 cm^{-1}.

4.4.26 5w – 3-(6,6-dimethyl-8-nitrooctahydroindolizin-7-yl)-5-methylisoxazole

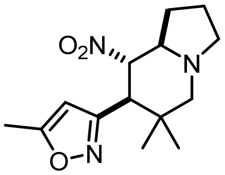

Performed on a 0.250 mmol scale using 20 mol% of **1c** (17.5 mg) and 20 mol% of **1c–H** (29.7 mg) affording the product in 96% yield (66.9 mg) as a white crystalline solid.

^{1}H NMR (400 MHz, $CDCl_3$) δ 5.86 (s, 1H), 4.81 (dd, *J* = 11.6, 9.6 Hz, 1H), 3.21 (d, *J* = 11.6 Hz, 1H), 3.15 – 3.01 (m, *J* = 8.5 Hz, 1H), 2.77 (d, *J* = 11.2 Hz, 1H), 2.40 – 2.29 (m, 4H), 2.24 (app q, *J* = 8.6 Hz, 1H), 2.13 (d, *J* = 11.2 Hz, 1H), 1.96 – 1.65 (m, 4H), 0.98 (s, 3H), 0.97 (s, 3H); **^{13}C NMR** (151 MHz, $CDCl_3$) δ 169.4, 160.2, 102.4, 89.2, 67.8, 65.0, 54.1, 48.3, 36.3, 28.4, 27.3, 22.2, 21.0, 12.4; **HRMS (ESI$^+$):** exact mass calculated for $[M+H]^+$ ($C_{14}H_{22}N_3O_3^+$) requires *m/z* 280.1656, found *m/z* 280.1659*;* **IR** (thin film) ν (cm^{-1}) 2946, 1551, 1369, 1277, 747 cm^{-1}.

4.4.27 5x – 7-cyclopropyl-6,6-dimethyl-8-nitrooctahydroindolizine

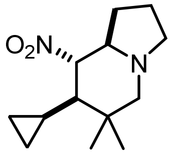

Performed on a 0.100 mmol scale using 30 mol% of **1c** (10.5 mg) and 30 mol% of **1c–H** (17.8 mg) affording the product in 30% yield (7.20 mg) as a white crystalline solid. Eluent for purification by flash column chromatography (toluene/CH_2Cl_2/EtOAc 1:1:0 to 3:3:1).

^{1}H NMR (600 MHz, $CDCl_3$) δ 4.35 (t, *J* = 10.3 Hz, 1H), 3.02 (t, *J* = 8.1 Hz, 1H), 2.62 (d, *J* = 11.0 Hz, 1H), 2.29 (dd, *J* = 15.3, 9.1 Hz, 1H), 2.14 (dd, *J* = 17.4, 8.6 Hz, 1H), 1.95 (d, *J* = 11.0 Hz, 1H), 1.87 – 1.76 (m, 2H), 1.74 – 1.64 (m, 1H), 1.61 – 1.51 (m, 1H), 1.08 (s, 3H), 1.07 – 1.01 (m, 4H), 0.66 – 0.59 (m, 1H), 0.59 – 0.52 (m, 1H), 0.40 – 0.33 (m, 1H), 0.24 – 0.17 (m, 1H), 0.08 – -0.02 (m, 1H); **^{13}C NMR** (151 MHz, $CDCl_3$) δ 92.6, 67.7, 65.8, 54.1, 53.2, 37.2, 28.0, 27.8, 22.1, 21.1, 9.7, 3.7, 1.8; **HRMS (ESI$^+$):** exact mass calculated for $[M+H]^+$ ($C_{13}H_{23}N_2O_2^+$) requires *m/z*

239.1754, found *m/z* 239.1756*;* **IR** (thin film) ν (cm^{-1}) 2788, 1543, 1393, 1275, 1222, 835, 733 cm^{-1}.

4.4.28 5y – 6,6-dimethyl-8-nitro-7-((E)-styryl)octahydroindolizine

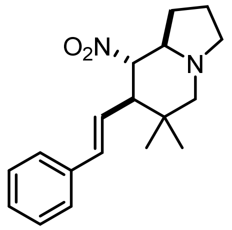

Performed on a 0.250 mmol scale using 10 mol% of **1c** (8.70 mg) and 25 mol% of **1c–H** (37.1 mg) affording the product in 66% yield (49.2 mg) as a white crystalline solid.

^{1}H NMR (600 MHz, $CDCl_3$) δ 7.33 (d, *J* = 7.3 Hz, 2H), 7.30 (app t, *J* = 7.6 Hz, 2H), 7.23 (t, *J* = 7.2 Hz, 1H), 6.42 (d, *J* = 15.7 Hz, 1H), 6.02 (dd, *J* = 15.7, 9.7 Hz, 1H), 4.41 (dd, *J* = 11.0, 9.7 Hz, 1H), 3.07 (td, *J* = 8.5, 1.9 Hz, 1H), 2.75 (d, *J* = 11.1 Hz, 1H), 2.48 (t, *J* = 10.2 Hz, 1H), 2.38 (td, *J* = 9.6, 6.1 Hz, 1H), 2.21 (app q, *J* = 9.0 Hz, 1H), 2.05 (d, *J* = 11.1 Hz, 1H), 1.87 (tdd, *J* = 12.4, 7.3, 3.7 Hz, 2H), 1.80 – 1.70 (m, 1H), 1.69 – 1.49 (m, 1H), 1.07 (s, 3H), 0.95 (s, 3H); **^{13}C NMR** (151 MHz, $CDCl_3$) δ 136.7, 135.5, 128.6 (2C), 127.9, 126.6 (2C), 123.9, 91.3, 67.1, 65.4, 54.7, 54.1, 36.3, 28.1, 27.3, 21.8, 21.0; **HRMS (ESI$^+$):** exact mass calculated for $[M+H]^+$ ($C_{18}H_{25}N_2O_2^+$) requires *m/z* 301.1911, found *m/z* 301.1911; **IR** (thin film) ν 2923, 1734, 1551, 1459, 1369, 746 cm^{-1}.

4.4.29 5z – 6,6-dimethyl-8-nitro-7-(4-phenylbut-1-yn-1-yl)octahydroindolizine

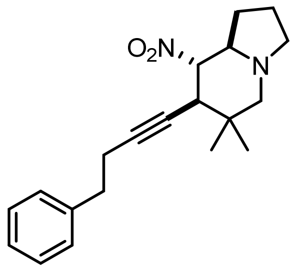

Performed on a 0.250 mmol scale using 10 mol% of **1c** (8.70 mg) and 25 mol% of **1c–H** (37.1 mg) affording the product in 61% yield (49.4 mg) as a yellow oil.

^{1}H NMR (600 MHz, $CDCl_3$) δ 7.30 – 7.26 (m, 2H), 7.22 – 7.16 (m, 3H), 4.26 (dd, *J* = 11.1, 9.7 Hz, 1H), 3.02 (td, *J* = 8.4, 1.7 Hz, 1H), 2.78 (t, *J* = 7.4 Hz, 2H), 2.71 (d, *J* = 11.1 Hz, 1H), 2.69 (dt, *J* = 11.1, 3.3, 1H), 2.45 (td, *J* = 7.4, 2.2 Hz, 2H), 2.22 (td, *J* = 9.6, 6.2 Hz, 1H), 2.17 (app q, *J* = 8.7 Hz, 1H), 1.93 (d, *J* = 11.2 Hz, 1H), 1.90 – 1.75 (m, 2H), 1.75 – 1.59 (m, 2H), 1.01 (s, 3H), 0.99 (s, 3H); **^{13}C NMR** (151 MHz, $CDCl_3$) δ 140.7, 128.6 (2C), 128.5 (2C), 126.3, 91.1, 85.4, 76.1, 67.0, 63.7, 53.9, 44.2, 36.3, 35.2, 28.1, 27.4, 21.8, 21.0, 20.9; **HRMS (ESI$^+$):** exact mass calculated for $[M+H]^+$ ($C_{20}H_{27}N_2O_2^+$) requires *m/z* 327.2067, found *m/z* 326.2063; **IR** (thin film) ν 2962, 2927, 2880, 2796, 1547, 1465, 746, 697 cm^{-1}.

4.5 Telescoped approach: One step reaction

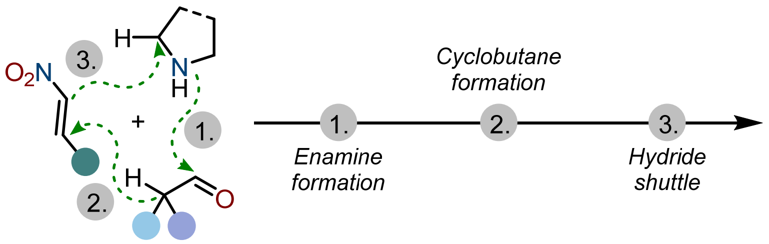

4.5.1 Procedure for the telescoped formation of 2a:

To flame dried Schlenk tube containing 4Å molecular sieves were added CH_2Cl_2 (1.20 mL), pyrrolidine (10.7 mg, 0.150 mmol, 1.00 equiv.), isobutyraldehyde (10.8 mg, 0.150 mmol, 1.00 equiv.) and trans-ß-nitrostyrene (22.4 mg, 0.150 mmol, 1.00 equiv.). The mixture was stirred at 23 °C for 2 h, after which the solution was taken up into a syringe and added to a vial containing **1c** (7.90 mg, 23.0 µmol, 15 mol%), **1c-H** (13.4 mg, 22.5 µmol, 15 mol%) and CH_2Cl_2 (0.30 mL) over the course of 30 min using a syringe pump. The reaction was then stirred for further 30 min, after which the solvent was removed under reduced pressure. Analysis of the crude mixture by NMR showed 64% yield of **2a** using 1,3,5-trimethoxybenzene as an internal standard.

4.5.2 5aa – 3,3-dimethyl-1-nitro-2,7-diphenyloctahydro-2H-quinolizine

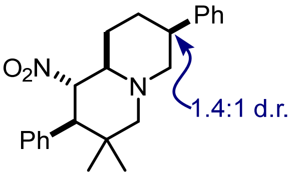

To a flame dried Schlenk tube containing dried 4Å molecular sieves were added CH_2Cl_2 (2.50 mL), 3-phenylpiperidine (80.6 mg, 500 µmol, 1.00 equiv.) and isobutyraldehyde (46.0 µL, 0.500 µmol, 1.00 equiv.). The reaction was stirred for 4 h at ambient temperature (23 °C) after which time trans-β-nitrostyrene (74.6 mg, 500 µmol, 1.00 equiv.) was added and the reaction was stirred for an additional 60 h. An aliquot was withdrawn to measure the d.r. of the cyclobutane (1.4:1 after 60 h at 23 °C). The solution was taken up into a syringe and was added to a vial containing **1c** (52.5 mg, 15 µmol, 30 mol%), **1c–H** (72.2 mg, 150 µmol, 30 mol%) and CH_2Cl_2 (400 µL) over the course of 30 min using a syringe pump. The reaction was further stirred for 30 minutes, the solvent was evaporated and the residue was purified by flash column chromatography (CH_2Cl_2) to give the desired product in 43% yield (77.6 mg, 1.4:1 d.r.) as a white crystalline solid.

Major diastereomer: **^{1}H NMR** (600 MHz, $CDCl_3$) δ 7.36 – 7.22 (m, 8H), 7.18 (s br, 2H), 4.95 (dd, J = 12.1, 9.4 Hz, 1H), 3.09 (d, J = 12.1 Hz, 1H), 2.98 (ddd, J = 11.2, 3.6, 1.9 Hz, 1H), 2.91 (tt, J = 11.8, 3.6 Hz, 1H), 2.55 (d, J = 11.5 Hz, 1H), 2.43 (td, J = 10.6, 2.8 Hz, 1H), 2.25 (d, J = 11.5 Hz, 1H), 2.20 (t, J = 11.3 Hz, 1H), 2.05 – 1.95 (m, 1H), 1.77 (ddd, J = 12.7, 6.3, 3.1 Hz, 1H), 1.71 – 1.61 (m, 1H), 1.55 (app qd, J = 12.9, 3.7 Hz, 1H), 1.02 (s, 3H), 0.80 (s, 3H); **^{13}C NMR** (151 MHz, CDCl3) δ 143.6, 135.4, 128.5 (2C), 128.1 (2C, HSQC), 128.0 (2C), 127.6, 127.1 (2C), 126.6, 91.5, 69.4, 65.1, 62.7, 56.3, 42.2, 35.1, 30.3, 29.3, 27.3, 21.2; **HRMS (ESI$^+$):** exact mass calculated for $[M+H]^+$ ($C_{23}H_{29}N_2O_2^+$) requires *m/z* 365.2224, found *m/z* 365. 2223; **IR** (thin film) ν 2922, 1543, 1380, 1123, 907, 767, 729, 701 cm^{-1}.

4.6 Enantioselective approach

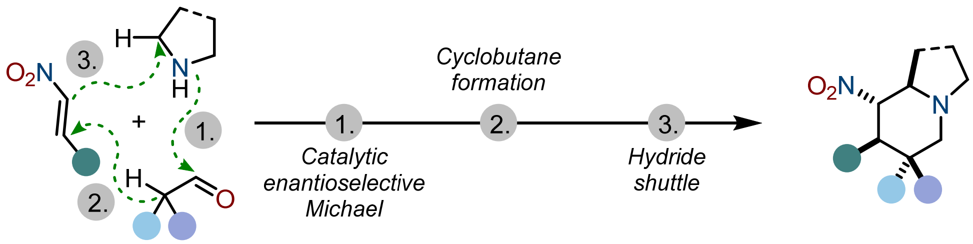

Asymmetric organocatalysed Michael addition: Chiral aldehydes are readily available through well-developed enantioselective Michael addition. For α-branched aldehydes, methods by Nugent and coworkers[17] and Yoshida and coworkers[18] deliver excellent results (Figure S3).

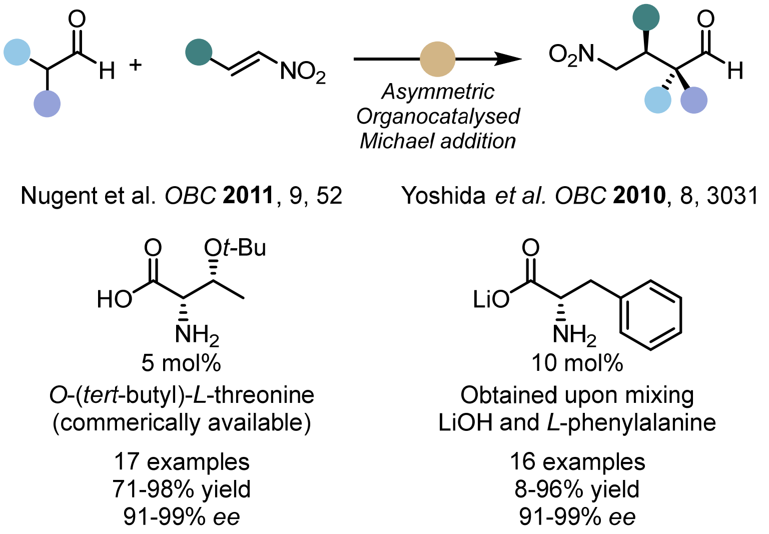

Figure S3. Asymmetric organocatalysed Michael addition

Aldehydes were prepared according to literature:

4.6.1 S2a – (*S*)-2,2-dimethyl-4-nitro-3-phenylbutanal

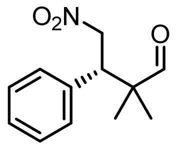

S2a was prepared according to the literature procedure.[18] Spectroscopic data were in accordance with reported data. Enantiomeric excess (97%) was determined by chiral HPLC analysis.

^{1}H NMR (600 MHz, $CDCl_3$) δ 9.54 (s, 1H), 7.36 – 7.28 (m, 3H), 7.23 – 7.17 (m, 2H), 4.86 (dd, *J* = 13.0, 11.3 Hz, 1H), 4.69 (dd, *J* = 13.1, 4.2 Hz, 1H), 3.78 (dd, *J* = 11.3, 4.2 Hz, 1H), 1.14 (s, 3H), 1.01 (s, 3H); **^{13}C NMR** (151 MHz, $CDCl_3$) δ 204.2, 135.3, 129.1 (2C), 128.7 (2C), 128.2, 76.3, 48.5, 48.2, 21.7, 18.9.

4.6.2 S2b – (*S*)-3-(4-methoxyphenyl)-2,2-dimethyl-4-nitrobutanal

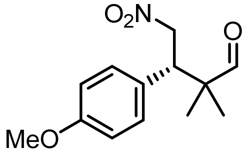

S2b was prepared according to the literature procedure.[17] Spectroscopic data were in accordance with reported data. Enantiomeric excess (98%) was determined by chiral HPLC analysis.

^{1}H NMR (700 MHz, $CDCl_3$) δ 9.53 (s, 1H), 7.18 – 7.00 (m, 2H), 6.89 – 6.74 (m, 2H), 4.80 (dd, *J* = 12.9, 11.4 Hz, 1H), 4.66 (dd, *J* = 12.9, 4.2 Hz, 1H), 3.79 (s, 3H), 3.73 (dd, *J* = 11.4, 4.2 Hz, 1H), 1.12 (s, 3H), 1.01 (s, 3H); **^{13}C NMR** (176 MHz, $CDCl_3$) δ 204.4, 159.3, 130.1 (2C), 127.05, 114.1 (2C), 76.5, 55.2, 48.4, 47.9, 21.6, 18.9.

4.6.3 S2c – (*S*)-2,2-dimethyl-4-nitro-3-(4-nitrophenyl)butanal

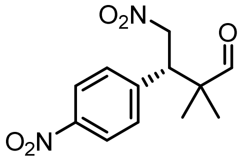

S2c was prepared according to the literature procedure.[18] Spectroscopic data were in accordance with reported data. Enantiomeric excess (99%) was determined by chiral HPLC analysis.

^{1}H NMR (700 MHz, $CDCl_3$) δ 9.49 (s, 1H), 8.21 (d, *J* = 8.7 Hz, 2H), 7.43 (d, *J* = 8.7 Hz, 2H), 4.92 (dd, *J* = 13.5, 11.5 Hz, 1H), 4.77 (dd, *J* = 13.5, 3.9 Hz, 1H), 3.93 (dd, *J* = 11.5, 3.9 Hz, 1H), 1.16 (s, 3H), 1.06 (s, 3H); **^{13}C NMR** (176 MHz, $CDCl_3$) δ 203.0, 147.7, 143.3, 130.2 (2C), 123.9 (2C), 75.8, 48.2, 48.2, 22.0, 19.2.

4.6.4 S2d – (*S*)-3-(4-fluorophenyl)-2,2-dimethyl-4-nitrobutanal

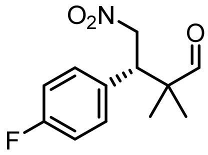

S2d was prepared according to the literature procedure.[17] Spectroscopic data was in accordance with reported data. Enantiomeric excess (97%) was determined by chiral HPLC analysis.

^{1}H NMR (700 MHz, $CDCl_3$) δ 9.51 (s, 1H), 7.23 – 7.09 (m, 2H), 7.09 – 6.90 (m, 2H), 4.82 (dd, *J* = 13.1, 11.5 Hz, 1H), 4.69 (dd, *J* = 13.1, 4.1 Hz, 1H), 3.78 (dd, *J* = 11.5, 4.1 Hz, 1H), 1.13 (s, 3H), 1.02 (s, 3H); **^{13}C NMR** (176 MHz, $CDCl_3$) δ 203.9, 162.4 (d, *J* = 247.6 Hz), 131.1 (d, *J* = 3.3 Hz), 130.7 (d, *J* = 8.1 Hz, 2C), 115.8 (d, *J* = 21.5 Hz, 2C), 76.4, 48.2, 47.8, 21.7, 19.0; **^{19}F NMR** (659 MHz, $CDCl_3$) δ -113.72.

General procedure for the enantioselective approach:

General Procedure B:

To a 7.00 mL vial equipped with a stirring bar was added enantioenriched aldehyde **S2** (100 µmol, 1.00 equiv.). Cyclohexane (500 µL, 0.20 M) and amine (1.10 equiv.) were subsequently added and the reaction was stirred at ambient temperature (23 °C) for 1 to 24 h (optimal reaction time was determined by ^{1}H-NMR analysis of aliquots). The solvent was removed on a rotary evaporator followed by drying on a high vacuum pump. The vial was refilled with argon and was subsequently sealed with a septa-cap. CH_2Cl_2 (0.20 mL, 0.50 M) was added and the solution was taken up with a syringe and added to a solution of Lewis acid **1c** (7.00 mg, 20 mol%) and its hydride **1c–H** (11.9 mg, 20 mol%) in CH_2Cl_2 (50.0 µL) over the course 20 min using a syringe pump. After the addition was complete, the reaction was stirred for 1 h at room temperature before the solvent was removed under reduced pressure. The residue was purified using column chromatography (CH_2Cl_2/toluene 1:1 to 4:1) to afford the final product.

General Procedure C:

To a 5.00 mL round bottom flask equipped with a stirring bar was added enantioenriched aldehyde **S2** (100 µmol, 1.00 equiv.). Cyclohexane (500 µL, 0.20 M) and amine (1.10 equiv.) were subsequently added and the flask was closed with an adapter with a closed stopcock and vacuum connector and placed in a sand bath (70 °C) for 1.5 to 24 h (optimal reaction time was determined by ^{1}H-NMR analysis of aliquots). The flask was allowed to cool at ambient

temperature (23 °C) for 2 minutes and then connected to a high vacuum pump to remove volatiles. The residue was dissolved in CH_2Cl_2 (200 µL, 0.50 M) and the solution was taken up with a syringe and added to a solution of Lewis acid **1c** (7.00 mg, 20 mol%) and its hydride **1c–H** (11.9 mg, 20 mol%) in CH_2Cl_2 (50.0 µL) over the course of 20 min using a syringe pump. After the addition was complete the reaction was stirred for 1 h at room temperature before the solvent was removed under reduced pressure. The residue was purified using flash column chromatography (CH_2Cl_2/toluene 1:1 to 4:1) to afford the final product.

4.6.5 (+)-5a – (7*S*,8*R*,8a*S*)-6,6-dimethyl-8-nitro-7-phenyloctahydroindolizine

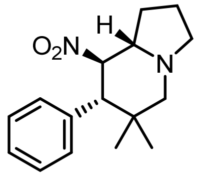

Performed according to the general procedure **C** (1.5 h reaction time) on a 0.250 mmol scale using 20 mol% of **1c** (3.00 mg) and 20 mol% of **1c–H** (6.0 mg), delivering the product in 68% yield (9.30 mg) as a white crystalline solid. $[\alpha]_D^{20}$= + 32.0 (c = 0.50, $CHCl_3$). Enantiomeric excess (95%) was determined by chiral HPLC analysis: Chiralpak IC, *n*-heptane+1% *i*-PrOH/EtOH 85:15, 1.0 mL/min, 25 °C, detection at 210 nm, retention time (min): 4.7 (major) and 5.0 (minor).

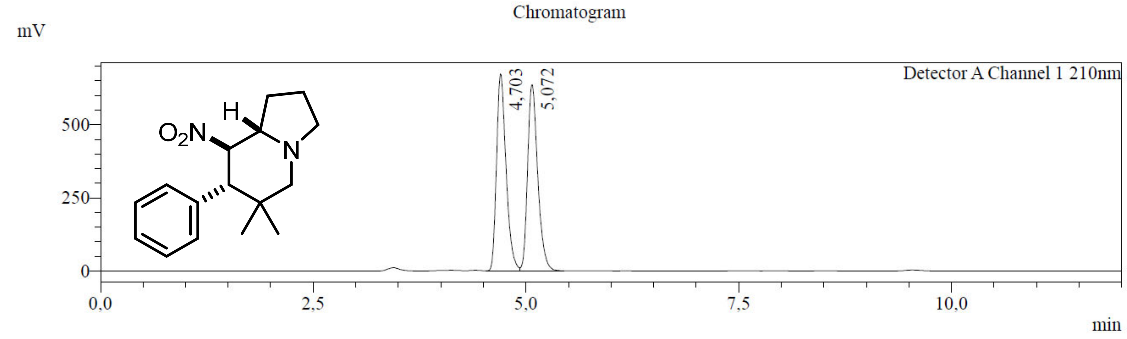

Detector A Channel 1 210nm

Peak#	Ret. Time	Area	Area%
1	4,703	5241398	49,887
2	5,072	5265211	50,113
Total		10506609	100,000

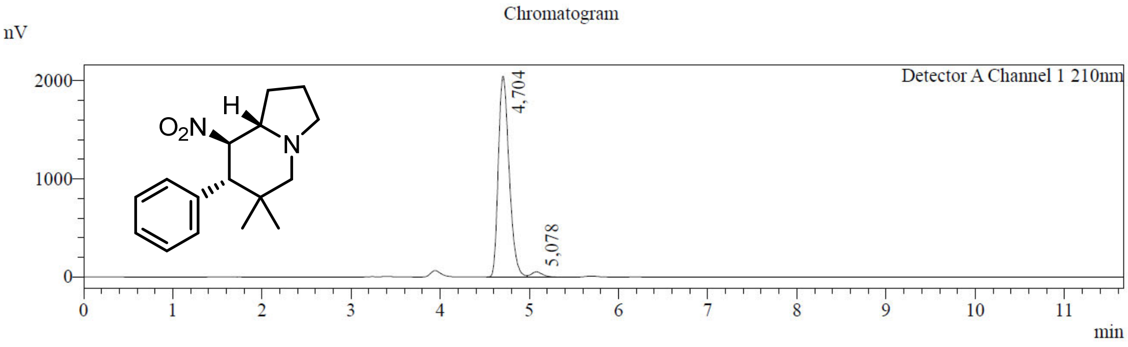

Detector A Channel 1 210nm			
Peak#	Ret. Time	Area	Area%
1	4,704	17248479	97,416
2	5,078	457499	2,584
Total		17705978	100,000

4.6.6 (+)-5n – (7*S*,8*R*,8a*S*)-7-(4-methoxyphenyl)-6,6-dimethyl-8-nitrooctahydroindolizine

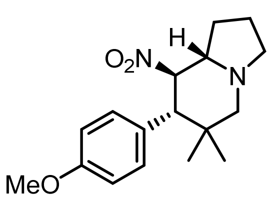

Performed according to the general procedure **B** (3.5 h reaction time) on a 100 µmol scale using 20 mol% of **1c** (7.00 mg) and 20 mol% of **1c–H** (11.9 mg), delivering the product in 66% yield (20.2 mg) as a white crystalline solid. $[\alpha]_D^{20}$= + 24.8 (c = 1.0, CHCl₃). Enantiomeric excess (99%) was determined by chiral HPLC analysis: Chiralpak IC, *n*-heptane+1% *i*-PrOH/*i*-PrOH 92:8, 0.7 mL/min, 25 °C, detection at 210 nm, retention time (min): 9.3 (major) and 10.1 (minor).

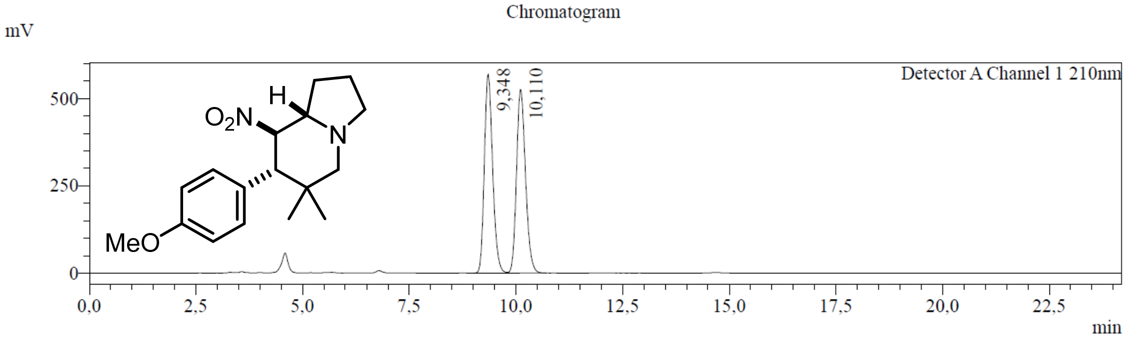

Detector A Channel 1 210nm			
Peak#	Ret. Time	Area	Area%
1	9,348	7705885	49,987
2	10,110	7709750	50,013
Total		15415635	100,000

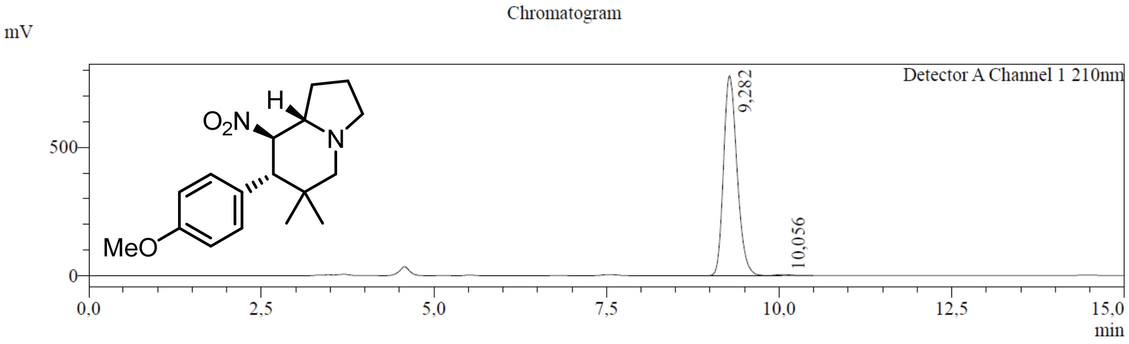

Detector A Channel 1 210nm

Peak#	Ret. Time	Area	Area%
1	9.282	10577574	99.444
2	10.056	59156	0.556
Total		10636730	100.000

4.6.7 (+)-5q – (7*S*,8*R*,8a*S*)-6,6-dimethyl-8-nitro-7-(4-nitrophenyl)octahydroindolizine

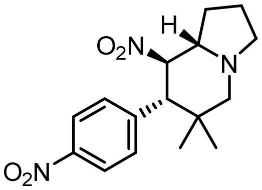

Performed according to the general procedure **B** (6 h reaction time) on a 0.250 mmol scale using a cyclohexane/CH_2Cl_2 mixture (10/1, 1.25 mL, 0.20 M) due to low solubility of the aldehyde in cyclohexane. The use of 20 mol% of **1c** (17.5 mg) and 20 mol% of **1c–H** (30.0 mg) afforded the product in 61% yield (48.7 mg) as a pale yellow crystalline solid. $[\alpha]_D^{20}$= + 24.3 (c = 1.0, $CHCl_3$). Enantiomeric excess (96%) was determined by chiral HPLC analysis: Chiralpak IC, *n*-heptane+1% *i*-PrOH/*i*-PrOH 90:10, 1.0 mL/min, 25 °C, detection at 210 nm, retention time (min): 15.6 (minor) and 28.0 (major).

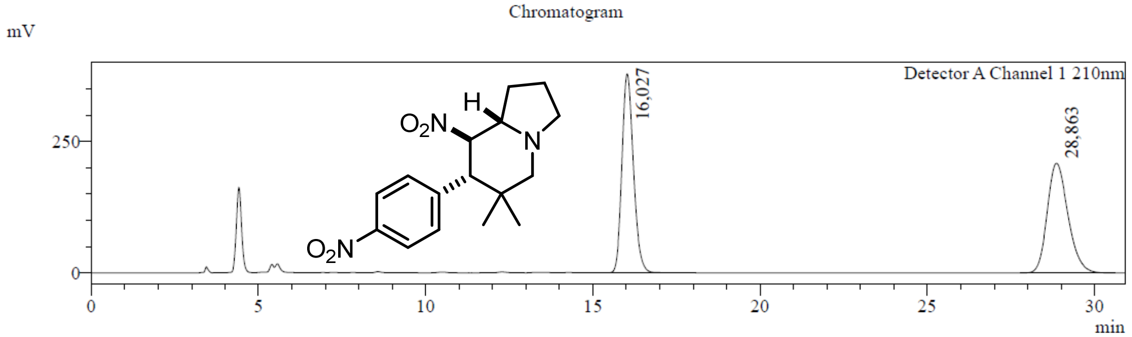

Detector A Channel 1 210nm

Peak#	Ret. Time	Area	Area%
1	16.027	9003511	49.934
2	28.863	9027219	50.066
Total		18030730	100.000

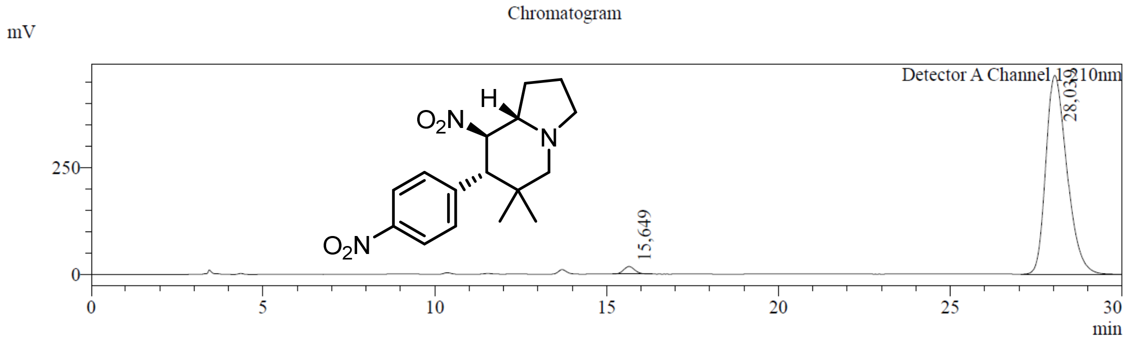

Detector A Channel 1 210nm

Peak#	Ret. Time	Area	Area%
1	15,649	396087	1,957
2	28,039	19846317	98,043
Total		20242404	100,000

4.6.8 (+)-5r – (7*S*,8*R*,8a*S*)-7-(4-fluorophenyl)-6,6-dimethyl-8-nitrooctahydroindolizine

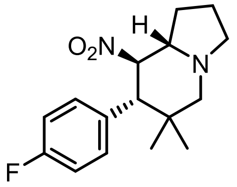

Performed according to the general procedure **B** (3 h reaction time) on a 100 µmol scale using 20 mol% of **1c** (7.00 mg) and 20 mol% of **1c–H** (11.9 mg) delivering the product in 81% yield (23.7 mg) as a white crystalline solid. $[\alpha]_D^{20}$= + 34.4 (c = 1.0, $CHCl_3$). Enantiomeric excess (98%) was determined by chiral HPLC analysis: Lux-3 Cellulose-3, *n*-heptane/EtOH 95:5, 0.5 mL/min, 25 °C, detection at 210 nm, retention time (min): 10.3 (major) and 11.7 (minor).

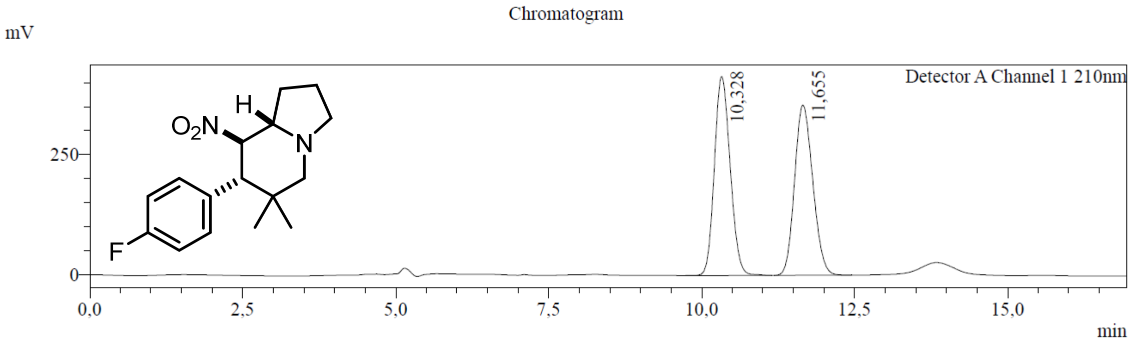

Detector A Channel 1 210nm

Peak#	Ret. Time	Area	Area%
1	10,328	7462626	50,264
2	11,655	7384230	49,736
Total		14846856	100,000

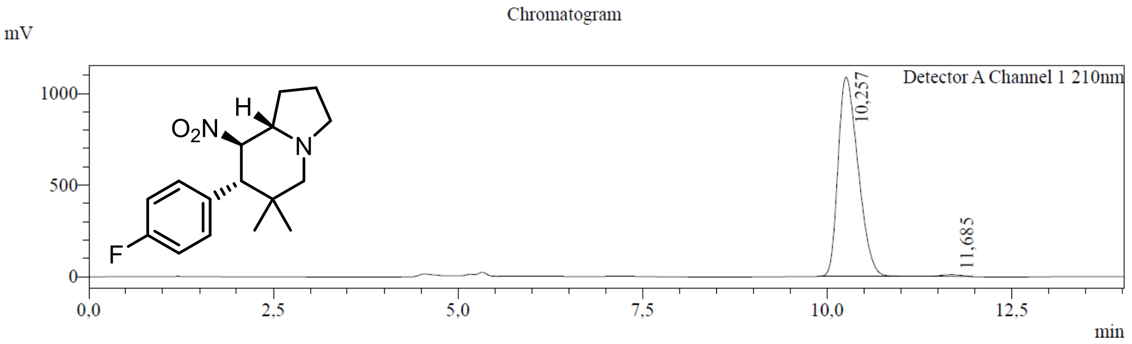

Detector A Channel 1 210nm			
Peak#	Ret. Time	Area	Area%
1	10,257	20516997	99,238
2	11,685	157515	0,762
Total		20674512	100,000

4.6.9 (+)-5b – (1*R*,2*S*,9a*S*)-3,3-dimethyl-1-nitro-2-phenyloctahydro-2H-quinolizine

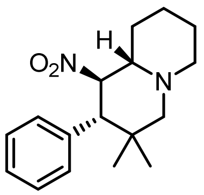

Performed according to the general procedure **C** (24 h reaction time) on a 50.0 µmol scale using 20 mol% of **1c** (3.50 mg) and 20 mol% of **1c–H** (6.00 mg) delivering the product in 56% yield (8.10 mg) as a white crystalline solid. $[\alpha]_D^{20}$= + 38.8 (c = 0.5, $CHCl_3$). Enantiomeric excess (97%) was determined by chiral HPLC analysis: Lux-3 Cellulose-3, *n*-heptane/*i*-PrOH/EtOH 98:1:1, 0.7 mL/min, 25 °C, detection at 210 nm, retention time (min): 19.1 (major) and 23.2 (minor).

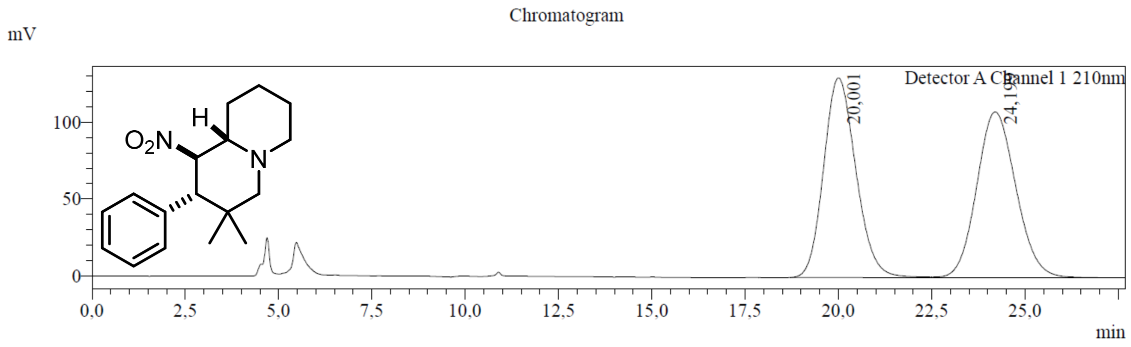

Detector A Channel 1 210nm			
Peak#	Ret. Time	Area	Area%
1	20,001	7816833	49,826
2	24,199	7871487	50,174
Total		15688320	100,000

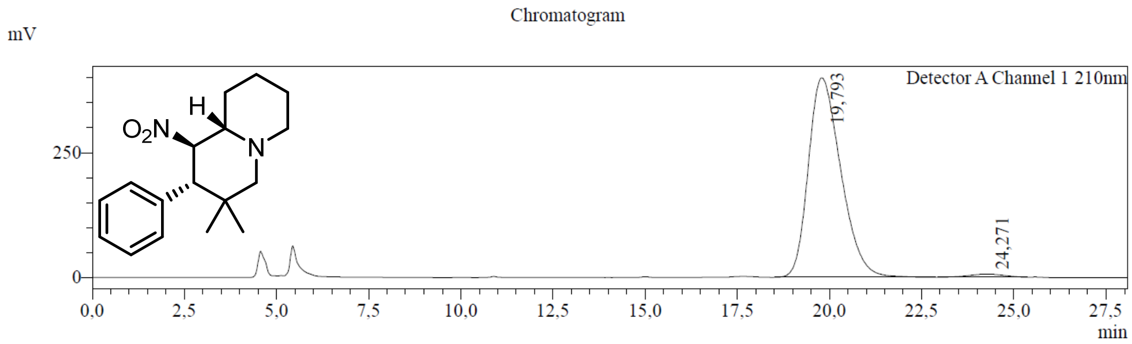

Detector A Channel 1 210nm			
Peak#	Ret. Time	Area	Area%
1	19,793	24544359	98,401
2	24,271	398845	1,599
Total		24943204	100,000

4.7 Alternative Michael acceptors

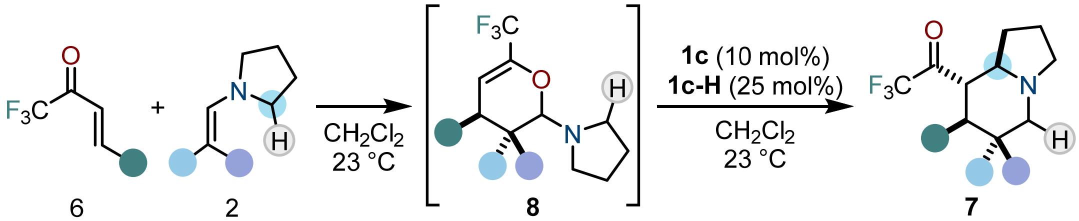

Figure S4. Synthesis of indolizidine derivatives bearing a trifluoromethyl ketone.

With the respective unsaturated trifluoroketones instead of nitroolefins, dihydropyrans such as **8** are formed quantitatively by ^{1}H-NMR. Only one diasteromer of **8** was observed by ^{1}H-NMR unless stated otherwise.

4.7.1 8c – 1-(3-ethyl-4-phenyl-6-(trifluoromethyl)-3,4-dihydro-2H-pyran-2-yl)pyrrolidine

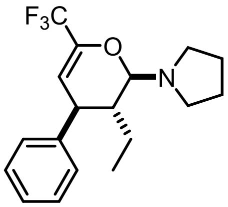

Obtained upon mixing equimolar amounts of (*E*)-1-(but-1-en-1-yl)pyrrolidine and (*E*)-1,1,1-trifluoro-4-phenylbut-3-en-2-one in CH_2Cl_2 at 23 °C. After 1 hour, an aliquot was withdrawn, concentrated in vacuo, redissolved in $CDCl_3$ and analysed by ^{1}H-NMR.

As this compound proved to be unstable no clean ^{13}C NMR spectrum could be obtained. For this reason, only ^{1}H NMR and ^{19}F NMR are reported.

^{1}H NMR (400 MHz, $CDCl_3$) δ 7.32 (app t, *J* = 7.4 Hz, 2H), 7.28 – 7.22 (m, 1H), 7.19 (d, *J* = 7.4 Hz, 2H), 5.31 (s, 1H), 4.84 (d, *J* = 9.8 Hz, 1H), 3.49 – 3.40 (m, 1H), 3.02 – 2.90 (m, 4H), 1.99 – 1.91 (m, 1H), 1.79 (t, *J* = 6.4 Hz, 4H), 1.60 – 1.48 (m, 1H), 1.37 – 1.26 (m, 1H), 0.84 (t, *J* = 7.5 Hz, 3H); **^{19}F NMR** (376 MHz, $CDCl_3$) δ -72.37.

4.7.2 7a – 1-(6,6-dimethyl-7-phenyloctahydroindolizin-8-yl)-2,2,2-trifluoroethan-1-one

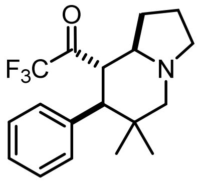

Performed according to the general procedure on a 0.250 mmol scale using 10 mol% of **1c** (8.60 mg) and 25 mol% of **1c–H** (36.4 mg) delivering the product in 98% yield (77.8 mg) as a white crystalline solid. Eluent for purification by flash column chromatography (toluene/CH_2Cl_2/EtOAc 5:5:1

to 3:3:1). Before inverse hydride shuttle, the respective dihydropyran was formed as a mixture of two epimers in a ratio of 1.1:1 with inconsequential stereochemistry.

^{1}H NMR (600 MHz, $CDCl_3$) δ 7.26 – 7.17 (m, 3H), 7.10 (s br, 2H), 3.60 (dd, *J* = 11.3, 9.9 Hz, 1H), 3.06 (td, *J* = 8.7, 1.8 Hz, 1H), 2.82 (d, *J* = 11.0 Hz, 1H), 2.78 (d, *J* = 11.8 Hz, 1H), 2.10 (app q, *J* = 8.9 Hz, 1H), 2.05 (d, *J* = 11.0 Hz, 1H), 2.04 – 1.98 (m, 1H), 1.91 – 1.82 (m, 1H), 1.81 – 1.73 (m, 1H), 1.72 – 1.58 (m, 2H), 1.02 (s, 3H), 0.75 (s, 3H); **^{13}C NMR** (151 MHz, $CDCl_3$) δ 194.2 (q, *J* = 35.4 Hz), 138.1, 131.8 (2C), 127.8 (2C), 127.1, 114.7 (q, *J* = 292.3 Hz), 68.7, 66.4, 55.3, 53.4, 51.0, 34.9, 28.8, 27.1, 21.7, 20.8; **^{19}F NMR** (565 MHz, $CDCl_3$) δ -78.51; **HRMS (ESI$^+$):** exact mass calculated for $[M+H]^+$ ($C_{18}H_{23}F_3NO^+$) requires *m/z* 326.1726, found *m/z* 326.1727; **IR** (thin film) ν 2964, 1750, 1203, 1155, 706 cm^{-1}.

4.7.3 7b – 1-(6,6-dimethyl-7-((*E*)-styryl)octahydroindolizin-8-yl)-2,2,2-trifluoroethan-1-one

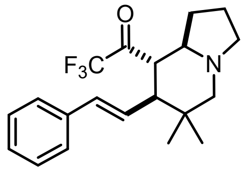

Performed according to the general procedure on a 0.150 mmol scale using 10 mol% of **1c** (5.30 mg) and 25 mol% of **1c–H** (22.3 mg) affording the product in 89% yield (47.0 mg) as a white crystalline solid. Eluent for purification by flash column chromatography (toluene/CH_2Cl_2/EtOAc 5:5:1 to 3:3:1). Before inverse hydride shuttle, the respective dihydropyran was formed as a mixture of two epimers in a ratio of 1.5:1 with inconsequential stereochemistry.

^{1}H NMR (700 MHz, $CDCl_3$) δ 7.30 (s, 2H), 7.29 (s, 2H), 7.24 – 7.20 (m, 1H), 6.30 (d, *J* = 15.7 Hz, 1H), 5.95 (dd, *J* = 15.7, 10.1 Hz, 1H), 3.13 (t, *J* = 10.5 Hz, 1H), 3.03 (td, *J* = 8.8, 2.0 Hz, 1H), 2.77 (d, *J* = 11.1 Hz, 1H), 2.26 (t, *J* = 10.6 Hz, 1H), 2.11 – 2.07 (m, 1H), 2.07 – 2.03 (m, 1H), 1.96 (d, *J* = 11.1 Hz, 1H), 1.88 – 1.80 (m, 1H), 1.75 (dddd, *J* = 12.0, 9.1, 5.8, 3.1 Hz, 1H), 1.70 – 1.63 (m, 1H), 1.50 (ddd, *J* = 22.1, 11.3, 7.1 Hz, 1H), 1.09 (s, 3H), 0.89 (s, 3H). **^{13}C NMR** (151 MHz, $CDCl_3$) δ 194.0 (q, *J* = 35.4 Hz), 136.9, 134.3, 128.7 (2C), 127.7, 126.6, 126.4 (2C), 115.1 (q, *J* = 291.8 Hz), 67.1, 65.9, 54.5, 53.6, 51.3, 34.8, 28.7, 27.1, 21.4, 20.9; **^{19}F NMR** (659 MHz, $CDCl_3$) δ -80.18; **HRMS (ESI$^+$):** exact mass calculated for $[M+H]^+$ ($C_{20}H_{25}F_3NO^+$) requires *m/z* 352.1883, found *m/z* 352.1879; **IR** (thin film) ν 2960, 2880, 2792, 1753, 1462, 1449, 1392, 1366, 1117, 1030, 744, 709 cm^{-1}.

4.7.4 7c – 1-(6-ethyl-7-phenyloctahydroindolizin-8-yl)-2,2,2-trifluoroethan-1-one

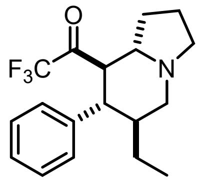

Performed according to the general procedure on a 0.100 mmol scale using 15 mol% of **1c** (10.5 mg) and 30 mol% of **1c–H** (35.6 mg) delivering the product in 72% yield (46.9 mg) as a white crystalline solid. Eluent for purification by flash column chromatography (toluene/CH_2Cl_2/EtOAc 3:3:1).

^{1}H NMR (600 MHz, $CDCl_3$) δ 7.26 (s br, 2H), 7.20 (t, *J* = 7.3 Hz, 1H), 7.10 (s br, 2H), 3.36 (dd, *J* = 10.5, 3.2 Hz, 1H), 3.23 (app t, *J* = 10.4 Hz, 1H), 3.15 (td, *J* = 8.9, 1.9 Hz, 1H), 2.61 (app t, *J* = 11.0 Hz, 1H), 2.21 (q, *J* = 9.0 Hz, 1H), 2.16 (td, *J* = 9.9, 5.8 Hz, 1H), 2.02 – 1.84 (m, 3H), 1.82 – 1.69 (m, 2H), 1.60 – 1.50 (m, 1H), 1.25 – 1.17 (m, 1H), 1.01 – 0.91 (m, 1H), 0.76 (t, *J* = 7.5 Hz, 3H); **^{13}C NMR** (151 MHz, $CDCl_3$) δ 193.9 (q, *J* = 35.9 Hz), 140.3, 128.9 (2C, HSQC), 128.7 (2C), 127.2, 114.5 (q, *J* = 291.9 Hz), 66.7, 56.6, 56.4, 53.4, 52.3, 42.0, 28.5, 23.7, 20.9, 11.0; **^{19}F NMR** (565 MHz, $CDCl_3$) δ -80.41; **HRMS (ESI$^+$):** exact mass calculated for $[M+H]^+$ ($C_{18}H_{23}NOF_3^+$) requires *m/z* 326.1726, found *m/z* 326.1729*;* **IR** (thin film) ν 2965, 2796, 1752, 1454, 1208, 1150, 1030, 761, 717, 701 cm^{-1}.

4.7.5 7d – 1-(3-ethyl-2-phenyloctahydro-2H-quinolizin-1-yl)-2,2,2-trifluoroethan-1-one

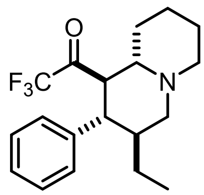

Performed according to the general procedure on a 0.200 mmol scale using 30 mol% of **1c** (21.0 mg) and 30 mol% of **1c–H** (35.6 mg) delivering the product in 78% yield (53.2 mg) as a colourless crystalline solid. Eluent for purification by flash column chromatography (toluene/CH_2Cl_2/EtOAc 3:3:1).

^{1}H NMR (500 MHz, $CDCl_3$) δ 7.26 (s br, 3H), 7.20 (t, *J* = 7.0 Hz, 1H), 6.90 (s br, 1H), 3.29 (t, *J* = 10.5 Hz, 1H), 3.11 – 3.00 (m, 1H), 2.94 (d, *J* = 11.3 Hz, 1H), 2.58 (t, *J* = 11.0 Hz, 1H), 2.20 (t, *J* = 9.5 Hz, 1H), 2.13 (td, *J* = 11.5, 3.9 Hz, 1H), 1.98 (d, *J* = 5.0 Hz, 2H), 1.74 (d, *J* = 12.9 Hz, 1H), 1.71 – 1.58 (m, 2H), 1.47 – 1.39 (m, 1H), 1.39 – 1.29 (m, 1H), 1.29 – 1.14 (m, 2H), 0.96 – 0.86 (m, 1H), 0.76 (t, *J* = 7.5 Hz, 3H); **^{13}C NMR** (126 MHz, $CDCl_3$) δ 196.0 (q, *J* = 33.6 Hz), 139.9, 130.2 (br), 128.9 (2C), 127.5, 125.9 (br), 114.4 (q, J = 292.1 Hz), 65.4, 60.9, 57.88, 56.6, 52.8, 41.3, 31.0, 25.5, 24.2, 23.8, 11.1; **^{19}F NMR** (565 MHz, $CDCl_3$) δ -80.21; **HRMS (ESI$^+$):** exact mass calculated for $[M+H]^+$ ($C_{19}H_{25}NOF_3^+$) requires *m/z* 340.1880, found *m/z* 340.1883*;* **IR** (thin film) ν 2936, 1749, 1455, 1204, 1154, 1015, 761, 713, 700 cm^{-1}.

Other Michael acceptors investigated:

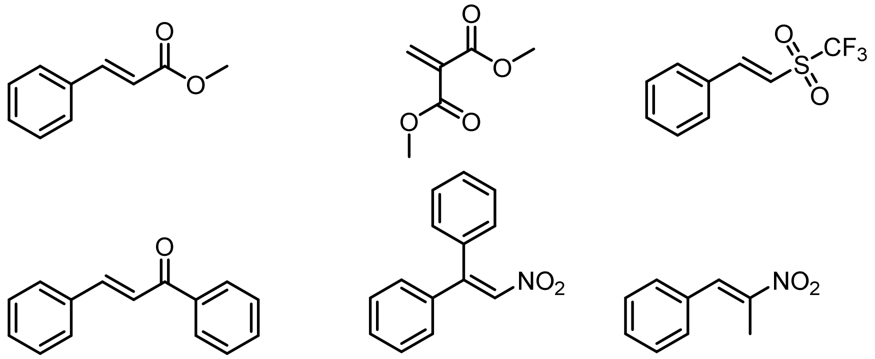

No product was detected under standard reaction conditions with these Michael acceptors.

(*Z*)-Nitroolefins were not investigated as it was anticipated that they will lead to the same cyclobutane species after isomerisation.[19]

4.8 Functionalisation

4.8.1 S3 – 6,6-dimethyl-7-phenyloctahydroindolizin-8-amine

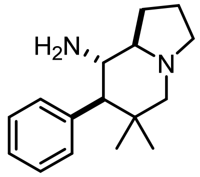

To a solution of the indolizidine **5a** (40.0 mg, 0.150 mmol) in *i*-PrOH (2.50 mL, 0.06 M), 700 µL of an aqueous solution of HCl (1.0 M, 700 µmol) was added, followed by Zn dust (143 mg, 2.19 mmol, 15.0 equiv.). The suspension was stirred for 2 h at 23 °C, after which excess acid was quenched by addition of a saturated aqueous $NaHCO_3$ solution (5.00 mL) and stirred vigorously for 20 min. The mixture was then filtered through a plug of Celite which was further washed with ethyl acetate (3.00 mL). The solution was extracted with EtOAc (3 x 3.00 mL) and the combined organic layers were dried over Na_2SO_4, filtered and the solvent was removed under reduced pressure. The residue was purified by flash column chromatography (heptane/DMA mix (CH_2Cl_2/MeOH/aq. NH_3 100:10:1) 2:3) to afford the desired compound in 94 % yield (33.5 mg) as a white crystalline solid.

^{1}H NMR (400 MHz, $CDCl_3$) δ 7.52 – 6.80 (m, 5H), 3.05 (dd, *J* = 10.6, 8.8 Hz, 1H), 2.99 (td, *J* = 8.7, 1.8 Hz, 1H), 2.70 (d, *J* = 10.8 Hz, 1H), 2.11 – 1.87 (m, 4H), 1.88 – 1.73 (m, 1H), 1.72 – 1.58 (m, 2H), 1.51 (app qd, J = 11.0, 6.7 Hz, 1H), 1.08 (s br, 2H), 0.89 (s, 3H), 0.66 (s, 3H). **^{13}C NMR** (101 MHz, $CDCl_3$) δ 139.4, 133.3 (br), 128.0 (2C), 127.4 (br), 126.6, 72.1, 67.0, 61.6, 54.7, 53.7, 36.0, 29.1, 27.9, 22.6, 21.1; **HRMS (ESI$^+$):** exact mass calculated for $[M+H]^+$ ($C_{16}H_{25}N_2^+$) requires *m/z* 245.2012, found *m/z* 245.2025; **IR** (thin film) ν 2958, 2924, 2870, 2784, 1452, 1364, 1171, 742, 703 cm^{-1}.

4.8.2 9 – 2,2-dimethylhexahydro-1H-pyrrolizin-1-yl)(phenyl)methanol

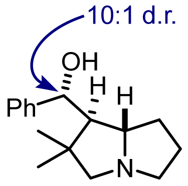

To a solution of aminoindolizidine **S3** (122 mg, 0.500 mmol) in water (1.00 mL) and HCl (1.0 M in H_2O, 1.20 mL, 2.40 equiv.), a solution of $NaNO_2$ (37.9 mg in 1.00 mL H_2O, 0.550 mmol, 1.10 equiv.) was added over 1 hour using a syringe pump. The solution was left stirring at ambient temperature (23 °C) for 16 h. The solution was then basified with a sat. Na_2CO_3 solution (2.00 mL) and extracted with CH_2Cl_2 (3 x 5.00 mL). The combined organic phases were dried with Na_2SO_4, filtered and concentrated under reduced pressure. The crude residue (d.r. 10:1) was

purified by flash column chromatography (gradient from CH_2Cl_2 to CH_2Cl_2/MeOH/aq. NH_3 100:10:1) to afford the desired product 86% (105 mg) as a pale yellow crystalline solid.

Major diastereomer: **^{1}H NMR** (600 MHz, $CDCl_3$) δ 7.41 – 7.32 (m, 4H), 7.32 – 7.27 (m, 1H), 4.61 (d, *J* = 8.9 Hz, 1H), 3.72 (dd, *J* = 14.9, 7.1 Hz, 1H), 3.03 – 2.93 (m, 1H), 2.77 (d, *J* = 8.8 Hz, 1H), 2.52 – 2.46 (m, 1H), 2.34 (d, *J* = 8.8 Hz, 1H), 2.08 – 2.01 (m, 1H), 1.93 – 1.85 (m, 1H), 1.85 – 1.76 (m, 2H), 1.69 – 1.62 (m, 2H), 1.12 (s, 3H), 0.37 (s, 3H); **^{13}C NMR** (151 MHz, $CDCl_3$) δ 144.1, 128.6 (2C), 128.2, 127.3 (2C), 75.7, 71.3, 69.2, 61.5, 55.3, 43.2, 34.3, 26.9, 25.8, 22.6; **HRMS (ESI$^+$):** exact mass calculated for $[M+H]^+$ ($C_{16}H_{24}NO^+$) requires *m/z* 246.1852, found *m/z* 246.1852; **IR** (thin film) ν 3100 br, 2952, 2876, 1455, 1369, 1092, 1074, 1014, 911, 764, 732, 701 cm^{-1}.

4.8.3 10 – 6,6-dimethyl-5-(2-methylallyl)-8-nitro-7-phenyloctahydroindolizine

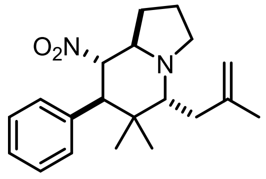

To a solution of indolizidine **5a** (137.0 mg, 0.500 mmol) in anhydrous CH_2Cl_2 (10.00 mL) was added *m*-chloroperbenzoic acid (77 %, 118.0 mg, 0.525 mmol, 1.05 equiv.) at 0 °C. The mixture was stirred at 0 °C for 2 h, quenched with a saturated aqueous $NaHCO_3$ solution (10.00 mL) and extracted with CH_2Cl_2 (3 x 10.00 mL). The combined organic phases were dried ($MgSO_4$), filtered and evaporated to give the crude *N*-oxide which was used without further purification in the next step (145.0 mg, quant.).

A solution of the crude *N*-oxide (16.0 mg, 0.055 mmol), methallyltri-*n*-butyltin (50.0 µL, 0.215 mmol, 3.92 equiv.) in anhydrous CH_2Cl_2 (1.00 mL) was cooled to 0 °C and trifluoroacetic anhydride (15.0 µL, 0.108 mmol, 1.96 equiv.) and potassium carbonate (22.8 mg, 0.165 mmol, 3.00 equiv.) were added. The suspension was stirred at 0 °C for 15 minutes then warmed to ambient temperature (40 °C) and stirred for 15 h. Anhydrous acetonitrile (1.00 mL) was added and the reaction was stirred for another 10 h at 40 °C. The reaction was subsequently concentrated in vacuo and the residue was purified by flash column chromatography (heptane/CH_2Cl_2 2:1 to 1:2) to afford homoallylic amine **10** in 49% yield (8.9 mg) over two steps as single diastereomer (>20:1 d.r.) as a colourless crystalline solid.

^{1}H NMR (600 MHz, $CDCl_3$) δ 7.29 – 7.21 (m, 3H), 7.18 (s br, 2H), 4.84 (dd, *J* = 12.1, 9.5 Hz, 1H), 4.82 (app d, *J* = 5.3 Hz, 2H), 3.32 (d, *J* = 11.9 Hz, 1H), 3.16 (dt, *J* = 8.9, 6.9 Hz, 1H), 3.00 – 2.92

(m, 2H), 2.84 (app q, J = 8.0 Hz, 1H), 2.46 (dd, J = 15.0, 5.3 Hz, 1H), 2.37 (dd, J = 15.0, 5.4 Hz, 1H), 1.94 – 1.85 (m, 2H), 1.82 (s, 3H), 1.80 – 1.69 (m, 2H), 1.10 (s, 3H), 0.72 (s, 3H); **^{13}C NMR** (151 MHz, $CDCl_3$) δ 145.1, 136.0, 132.3 (2C, HSQC), 128.1 (2C), 127.5, 112.2, 91.3, 63.6, 58.9, 51.0, 50.8, 39.9, 31.7, 29.1, 26.5, 23.9, 23.0, 22.0; **HRMS (ESI$^+$):** exact mass calculated for $[M+H]^+$ ($C_{20}H_{29}N_2O_2^+$) requires *m/z* 329.2224, found *m/z* 329.2222; **IR** (thin film) ν 2968, 2927, 1547, 1454, 1365, 890, 733, 703 cm^{-1}.

4.8.4 11 – 6,6-dimethyl-8-nitro-7-phenyloctahydroindolizine-5-carbonitrile

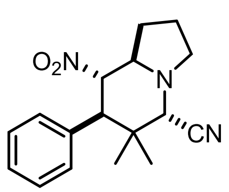

The crude *N*-oxide of indolizidine **5a** was prepared according to the procedure described for the synthesis of **10**.

Trifluoroacetic anhydride (50.0 µL, 0.360 mmol, 1.60 equiv.) was added to a cooled solution (0 °C) of the crude N-oxide (63.9 mg, 0.220 mmol). After stirring for 1 h at 0 °C and 15 minutes at 24 °C, a solution of KCN (44.0 mg, 0.680 mmol, 3.00 equiv.) in water (30.0 µL) was added and the reaction was stirred for 30 min at 23 °C. After the addition of a saturated aqueous Na_2CO_3 solution (2.00 mL), the mixture was extracted with CH_2Cl_2 (3 x 3.00 mL) and the combined organic phases were dried (Na_2SO_4). The solvent was evaporated under reduced pressure and the residue purified by flash column chromatography (heptane/CH_2Cl_2 1:1 to 3:1) to afford α-cyanoindolizidine **11** in 58% yield (43.3 mg) over two steps as single diastereomer (>20:1 d.r.) as a colourless crystalline solid.

^{1}H NMR (600 MHz, $CDCl_3$) δ 7.35 – 7.26 (m, 3H), 7.18 (d br, J = 6.9 Hz, 2H), 4.86 (dd, J = 11.8, 9.4 Hz, 1H), 3.72 (s, 1H), 3.36 (d, J = 11.8 Hz, 1H), 3.08 (td, J = 8.5, 2.5 Hz, 1H), 3.01 (td, J = 9.2, 6.3 Hz, 1H), 2.68 (app q, J = 8.7 Hz, 1H), 2.05 – 1.93 (m, 2H), 1.91 – 1.78 (m, 2H), 1.09 (s, 3H), 1.03 (s, 3H); **^{13}C NMR** (151 MHz, $CDCl_3$) δ 134.0, 128.3 (2C), 128.0, 127.7 (2C, HSQC), 114.7, 89.0, 63.1, 61.9, 52.2, 51.3, 38.8, 28.1, 26.4, 21.5, 20.8; **HRMS (ESI$^+$):** exact mass calculated for $[M+Na]^+$ ($C_{17}H_{21}N_3O_2Na^+$) requires *m/z* 322.1526, found *m/z* 322.1529; **IR** (thin film) ν 2974, 2826, 1551, 1360, 704cm^{-1}.

4.8.5 12 – *N-3*,3-dimethyl-2-phenyloctahydro-2H-quinolizin-1-yl)acetamide

To a solution of the indolizidine **5b** (10.1 mg, 35.0 µmol, 1.00 equiv.) in *i*-PrOH (100 µL, 0.35 M), 100 µL of an aqueous solution of HCl (1.0 M, 20.0 equiv.) was added, followed by Zn dust

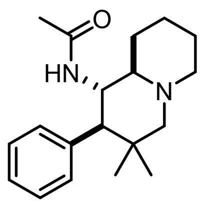

(34.4 mg, 525 µmol, 15.0 equiv.). The suspension was stirred for 2 h at 23 °C, after which excess acid was quenched by addition of a saturated aqueous $NaHCO_3$ solution (5.00 mL) and stirred vigorously for 20 min. The mixture was then filtered through a plug of Celite which was further washed with ethyl acetate (3.00 mL). The solution was extracted with ethyl acetate (3 x 1.00 mL) and the combined organic layers were dried over Na_2SO_4, filtered and the solvent was removed under reduced pressure. The residue was dissolved in anhydrous dichloromethane (200 µL), after which acetic anhydride (4.3 µL, 45.5 µmol, 1.3 equiv.) and trimethylamine (20.0 µL, 140 µmol, 4.0 equiv.) were added and the resulting solution was stirred for 5 h at ambient temperature (23 °C). A solution of saturated aqueous $NaHCO_3$ (1.00 mL) and the mixture was extracted three times with dichloromethane (3 x 1.00 mL). The combined organic layers were dried over anhydrous Na_2SO_4, filtered and concentrated under reduced pressure. The crude residue was purified by flash column chromatography (CH_2Cl_2 / DMA(CH_2Cl_2/MeOH/aq. NH_3 100:10:1) 10:1 to 1:1) to afford the desired product **12** in 68% yield (7.4 mg) as a white crystalline solid.

^{1}H NMR (600 MHz, $CDCl_3$) δ 7.22 (s br, 3H), 7.19 (t, *J* = 7.2 Hz, 1H), 7.02 (s, 1H), 4.69 (d, *J* = 9.8 Hz, 1H), 4.34 (app dt, *J* = 11.6, 9.5 Hz, 1H), 2.80 (d, *J* = 10.9 Hz, 1H), 2.47 (d, *J* = 11.3 Hz, 1H), 2.26 (d, *J* = 12.0 Hz, 1H), 1.99 (d, *J* = 11.3 Hz, 1H), 1.94 (td, *J* = 11.2, 4.1 Hz, 1H), 1.80 – 1.74 (m, 2H), 1.64 – 1.59 (m, 5H), 1.57 – 1.52 (m, 1H), 1.46 – 1.38 (m, 1H), 1.19 – 1.10 (m, 1H), 0.98 (s, 3H), 0.73 (s, 3H); **^{13}C NMR** (151 MHz, $CDCl_3$) δ 169.8, 138.6, 131.9 (2C, HSQC), 127.6 (2C, HSQC), 126.6, 70.4, 69.3, 59.5, 56.6, 51.4, 35.1, 29.4, 28.1, 25.8, 24.6, 23.3, 21.3;**HRMS (ESI$^+$):** exact mass calculated for $[M+H]^+$ ($C_{19}H_{29}N_2O^+$) requires *m/z* 301.2274, found *m/z* 342.1675; **IR** (thin film) ν 3286, 2932, 1647, 1559, 1373, 1313, 741, 702 cm^{-1}.

4.8.6 13 – 1-(3-ethyl-2-phenyloctahydro-2H-quinolizin-1-yl)-2,2,2-trifluoroethan-1-ol

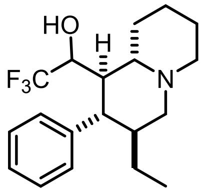

To a solution of sodium borohydride (11.3 mg, 0.30 mmol, 5.00 equiv) at 0 °C was added 1-(3-ethyl-2-phenyloctahydro-2H-quinolizin-1-yl)-2,2,2-trifluoroethan-1-one **7d** (20.4 mg, 0.06 mmol, 1.00 equiv.). The cooling bath was removed and the reaction was stirred at ambient temperature (23 °C) for 3 h. Concentrated HCl (0.10 mL, 20.0 equiv.) was added and the mixture was concentrated under reduced pressure. The white solid (3.7:1 d.r.) was purified by flash

column chromatography (CH_2Cl_2/MeOH/$NH_{3(aq)}$ 400:10:1) to give the desired product **13** in 85% yield (17.2 mg, 7.5:1 d.r.) as a white crystalline solid.

Major diastereomer: **^{1}H NMR** (700 MHz, $CDCl_3$) δ 7.31 (t, *J* = 6.9 Hz, 2H), 7.41 – 7.07 (s br, 2H), 7.23 (t, *J* = 7.3 Hz, 1H), 3.89 (q, *J* = 8.3 Hz, 1H), 3.29 (s br, 1H, OH), 2.99 (s br, 2H), 2.37 (s br, 3H), 2.24 (s br, 2H), 1.95 (s br, 2H), 1.80 (d, *J* = 11.6 Hz, 1H), 1.66 (s br, 2H), 1.39 (s br, 1H), 1.31 (d br, *J* = 12.7 Hz, 1H), 1.11 – 1.00 (m, 1H), 0.90 – 0.76 (m, 1H), 0.68 (t, *J* = 7.5 Hz, 3H); **^{13}C NMR** (101 MHz, $CDCl_3$) δ 142.1, 129.0 (2C), 128.8 (2C), 127.0, 125.7 (q, *J* = 284.4 Hz), 70.4 (q, *J* = 29.6 Hz); 65.0, 60.4, 57.2, 50.1, 49.9, 42.1, 30.9, 24.9, 24.3, 24.1, 10.8; **^{19}F NMR** (565 MHz, $CDCl_3$) δ -71.56; **HRMS (ESI$^+$):** exact mass calculated for $[M+H]^+$ ($C_{19}H_{27}NOF_3^+$) requires *m/z* 342.2039, found *m/z* 342.2040*;* **IR** (thin film) ν 2935, 2768 br, 1453, 1271, 1147, 1138, 1117, 1101, 762, 703 cm^{-1}.

4.8.7 14 – 1-(6,6-dimethyl-8-nitro-7-phenyloctahydroindolizin-5-yl)propan-2-one

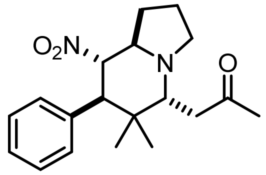

The crude *N*-oxide of indolizidine **5a** was prepared according to the procedure described for the synthesis of **10**.

To a solution of the crude *N*-oxide (29.0 mg, 0.100 mmol) in anhydrous CH_2Cl_2 (1.00 mL) in a 4 mL glass vial cooled to 0 °C was added trifluoroacetic anhydride (20.0 µL, 0.144 mmol, 1.44 equiv.). The mixture was stirred for 15 minutes at 0 °C then acetone (1.00 mL, 136 equiv.) was added, the vial sealed and the reaction heated to 70 °C for 36 h. The vial was cooled to room temperature, solid $NaHCO_3$ was added until no further gas evolution was observed and the mixture was subsequently concentrated under reduced pressure. The residue was extracted with CH_2Cl_2 (3 x 2.00 mL) and the combined extracts were concentrated. The crude material was purified by column chromatography (heptane/CH_2Cl_2 2:1 to CH_2Cl_2) to afford the product **14** in 28% yield (9.1 mg) over two steps as single diastereomer (>20:1 d.r.) as a colourless crystalline solid.

^{1}H NMR (600 MHz, $CDCl_3$) δ 7.31 – 7.21 (m, 3H), 7.15 (s br, 2H), 4.86 (dd, *J* = 11.9, 9.4 Hz, 1H), 3.51 (dd, *J* = 5.9, 4.4 Hz, 1H), 3.15 (d, *J* = 11.9 Hz, 1H), 2.95 (td, *J* = 8.6, 3.0 Hz, 1H), 2.93 – 2.88 (m, 1H), 2.82 (dd, *J* = 17.6, 6.1 Hz, 1H), 2.67 (dd, *J* = 17.6, 4.2 Hz, 1H), 2.42 (app q, *J* = 8.3 Hz, 1H), 2.28 (s, 3H), 1.93 – 1.83 (m, 2H), 1.79 – 1.66 (m, 2H), 1.12 (s, 3H), 0.63 (s, 3H); **^{13}C NMR** (101 MHz, $CDCl_3$) δ 207.3, 135.5, 131.1 (2C, HSQC), 128.2 (2C), 127.7, 91.1, 60.8, 59.7, 51.4,

50.4, 39.1, 36.6, 30.7, 28.8, 25.9, 23.3, 21.5; **HRMS (ESI$^+$):** exact mass calculated for $[M+H]^+$ ($C_{19}H_{27}N_2O_3^+$) requires *m/z* 331.2016, found *m/z* 331.2018; **IR** (thin film) ν 2967, 1713, 1546, 1352, 1275, 1261, 751 cm^{-1}.

5 References

1. Brannock, K. C., Bell, A., Burpitt, R. D. & Kelly, C. A. Enamine Chemistry. IV. Cycloaddition Reactions of Enamines Derived from Aldehydes and Acyclic Ketones 1,2. *J. Org. Chem.* **29**, 801–812 (1964).

2. Opitz, G. & Kleemann, M. Enamine, XIV. Cyclobutanon-Basen aus Enaminen und Carbonsäurechloriden. *Justus Liebigs Ann. Chem.* **665**, 114–125 (1963).

3. Lavallo, V., Frey, G. D., Kousar, S., Donnadieu, B. & Bertrand, G. Allene formation by gold catalyzed cross-coupling of masked carbenes and vinylidenes. *Proc. Natl. Acad. Sci. U. S. A.* **104**, 13569–13573 (2007).

4. Dupont-Passelaigue, E., Mialhe, S., Rieu, J.-P., Junquero, D. & Valeille, K. Derivatives of 2h pyridazin- 3 -ones, their preparation and their use as scd-1 inhibitors. (2011).

5. Li, J., Cai, S., Chen, J., Zhao, Y. & Wang, D. Z. Visible light induced photocatalytic conversion of enamines into amides. *Synlett* **25**, 1626–1628 (2014).

6. Opitz, G., Hellmann, H. & Schubert, H. W. Enamine, I: Einfache Enamine und ihre Spektren. *Justus Liebigs Ann. Chem.* **623**, 112–117 (1959).

7. Kurihara, H. & Mishima, H. A convenient synthesis of 2-aminopyridine-3-carbonitriies. *Journal of Heterocyclic Chemistry* **14**, 1077–1079 (1977).

8. Wittig, P. & Mayer, R. Über das Verhältnis Enamin zu Aminal bei der Umsetzung des n-Butyraldehyds mit sekundären Aminen. *Zeitschrift für Chemie* **7**, 57–58 (1967).

9. Neuhaus, J. D., Angyal, P., Oost, R. & Maulide, N. (3+2) Cycloadditions of Thiouronium Ylides: A Room-Temperature, One-Pot Approach to Dihydrothiophenes. *J. Org. Chem.* **83**, 2479–2485 (2018).

10. Tissot, M., Müller, D., Belot, S. & Alexakis, A. Enantioselective and Regiodivergent Copper-Catalyzed Conjugate Addition of Trialkylaluminium Reagents to Extended Nitro-Michael Acceptors. *Org. Lett.* **12**, 2770–2773 (2010).

11. Dhakal, R. C. & Dieter, R. K. Regioselective 1,4-Conjugate Addition of Grignard Reagents to Nitrodienes in the Presence of Catalytic Amounts of Zn(II) Salts. *Org. Lett.*

16, 1362–1365 (2014).

12. Wang, Y., Han, J., Chen, J. & Cao, W. An efficient route to 3-trifluoromethylpyrazole via cyclization/1,5-H shift and its applications in the synthesis of bioactive compounds. *Tetrahedron* **71**, 8256–8262 (2015).

13. Gómez-Bengoa, E. *et al.* Asymmetric synthesis of propargylic alcohols via aldol reaction of aldehydes with ynals promoted by prolinol ether–transition metal–Brønsted acid cooperative catalysis. *Chem. Sci.* **4**, 3198–3204 (2013).

14. Greb, L., Daniliuc, C.-G., Bergander, K. & Paradies, J. Functional-Group Tolerance in Frustrated Lewis Pairs: Hydrogenation of Nitroolefins and Acrylates. *Angew. Chem. Int. Ed.* **52**, 5876–5879 (2013).

15. Morozova, V., Mayer, P. & Berionni, G. Scope and Mechanisms of Frustrated Lewis Pair Catalyzed Hydrogenation Reactions of Electron-Deficient C=C Double Bonds. *Angew. Chem. Int. Ed.* **54**, 14508–14512 (2015).

16. Castro-Alvarez, A. *et al.* NMR and Computational Studies on the Reactions of Enamines with Nitroalkenes That May Pass through Cyclobutanes. *ACS Omega* **4**, 18167–18194 (2019).

17. Nugent, T. C., Shoaib, M. & Shoaib, A. Practical access to highly enantioenriched quaternary carbon Michael adducts using simple organocatalysts. *Org. Biomol. Chem.* **9**, 52–56 (2011).

18. Yoshida, M., Sato, A. & Hara, S. Asymmetric Michael addition of aldehydes to nitroalkenes using a primary amino acid lithium salt. *Org. Biomol. Chem.* **8**, 3031–3036 (2010).

19. Hayashi, Y., Gotoh, H., Hayashi, T. & Shoji, M. Diphenylprolinol silyl ethers as efficient organocatalysts for the asymmetric Michael reaction of aldehydes and nitroalkenes. *Angew. Chem. Int. Ed.* **44**, 4212–4215 (2005).

6 NMR-Spectra

6.1.1.1 2a – 1-(2-methylprop-1-en-1-yl)pyrrolidine – ^{1}H NMR (400 MHz)

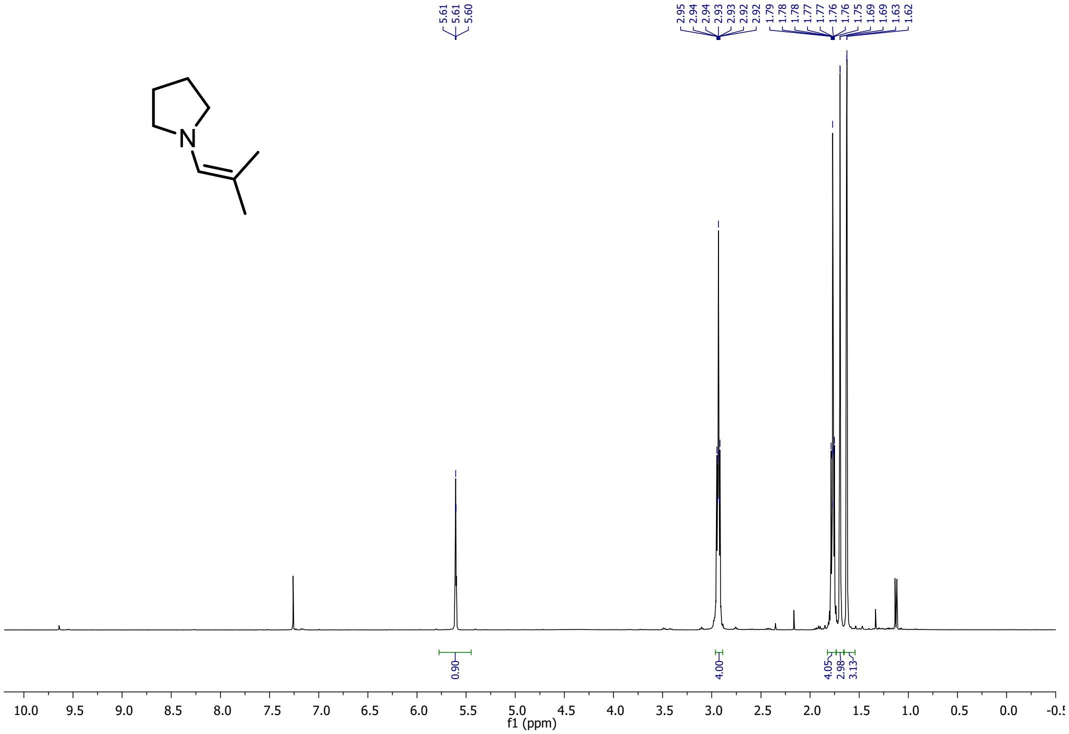

6.1.1.2 2b – 1-(2-methylprop-1-en-1-yl)piperidine – ^{1}H NMR (400 MHz)

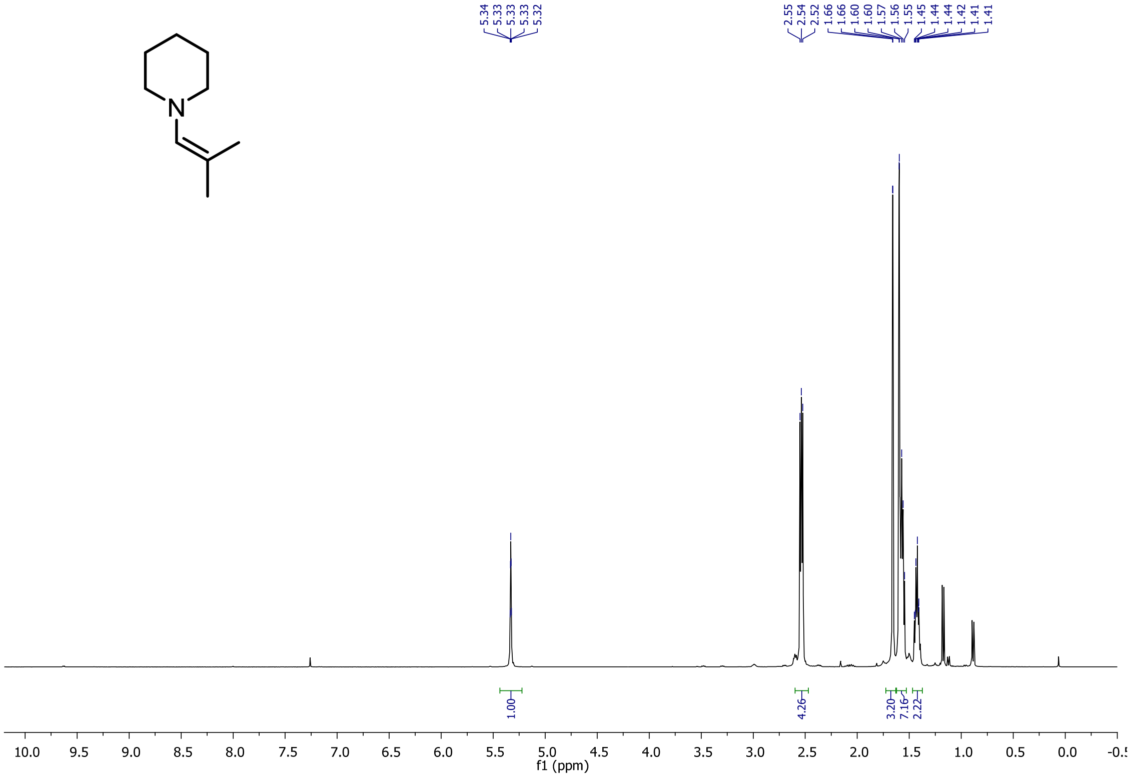

6.1.1.3 2c – 1-(2-methylprop-1-en-1-yl)azepane – ^{1}H NMR (400 MHz)

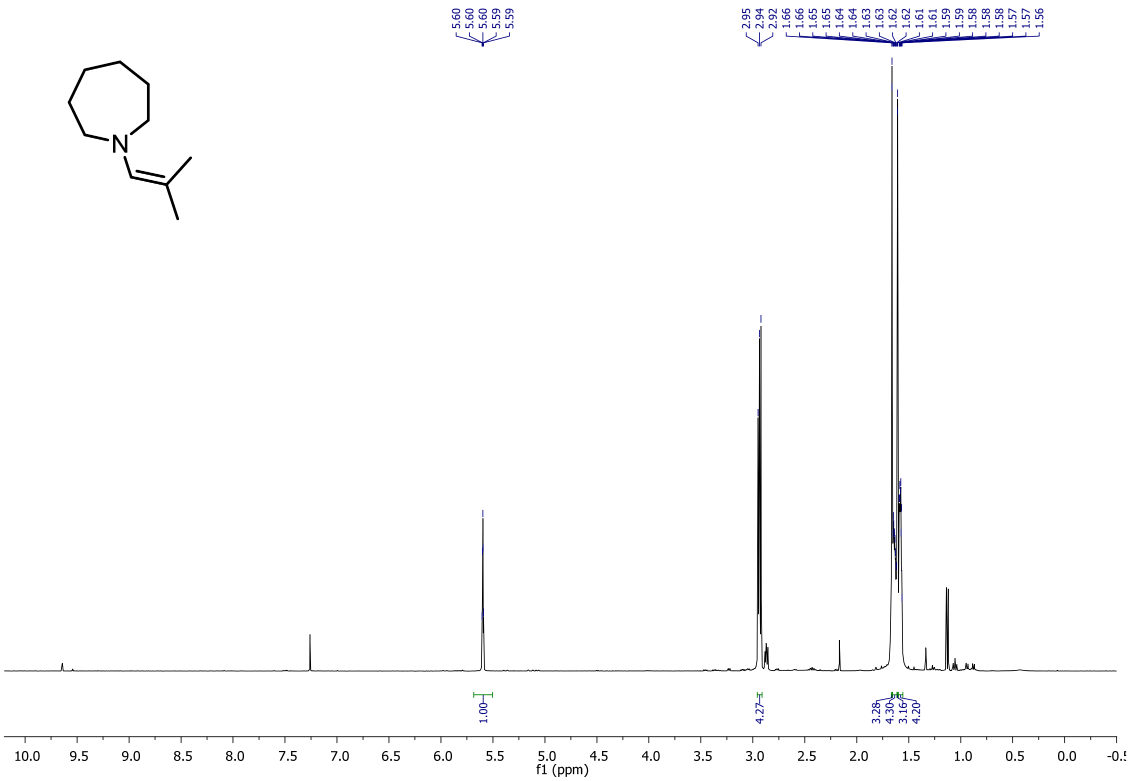

6.1.1.4 2d – 2-(2-methylprop-1-en-1-yl)octahydro-*1H*-isoindole – ^{1}H NMR (400 MHz)

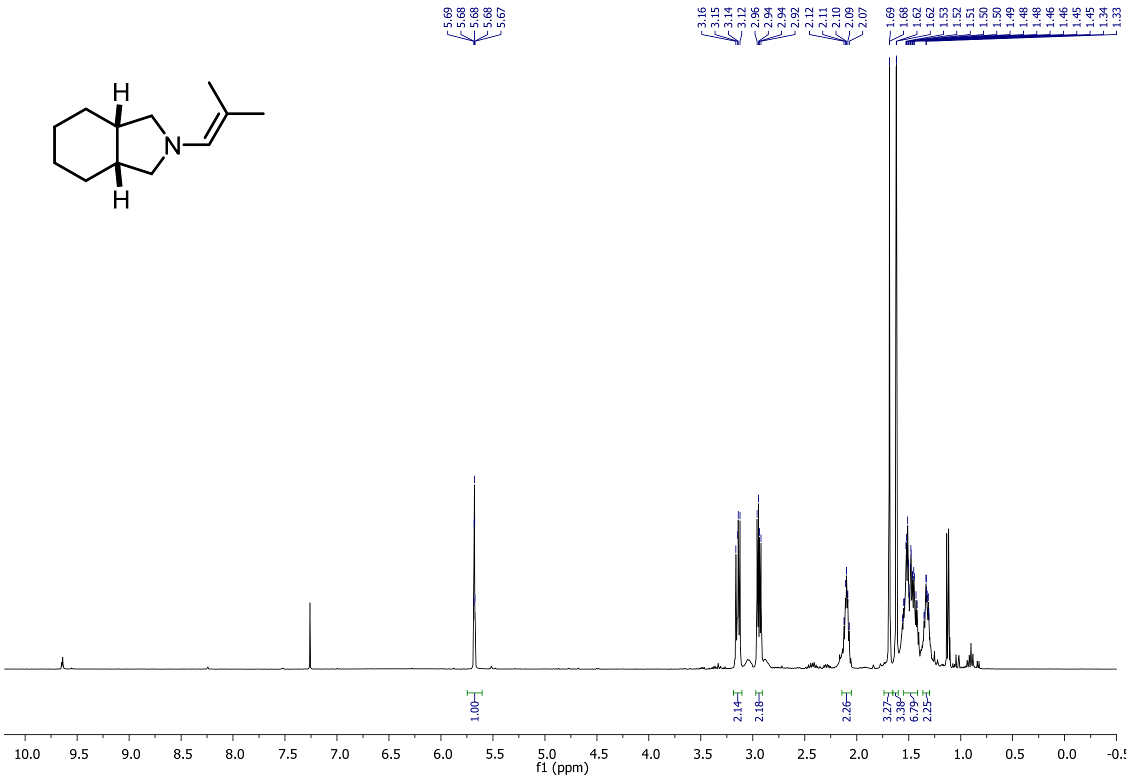

6.1.1.5 2e – 2-(2-methylprop-1-en-1-yl)-1,2,3,4-tetrahydroisoquinoline – ^{1}H NMR (400 MHz)

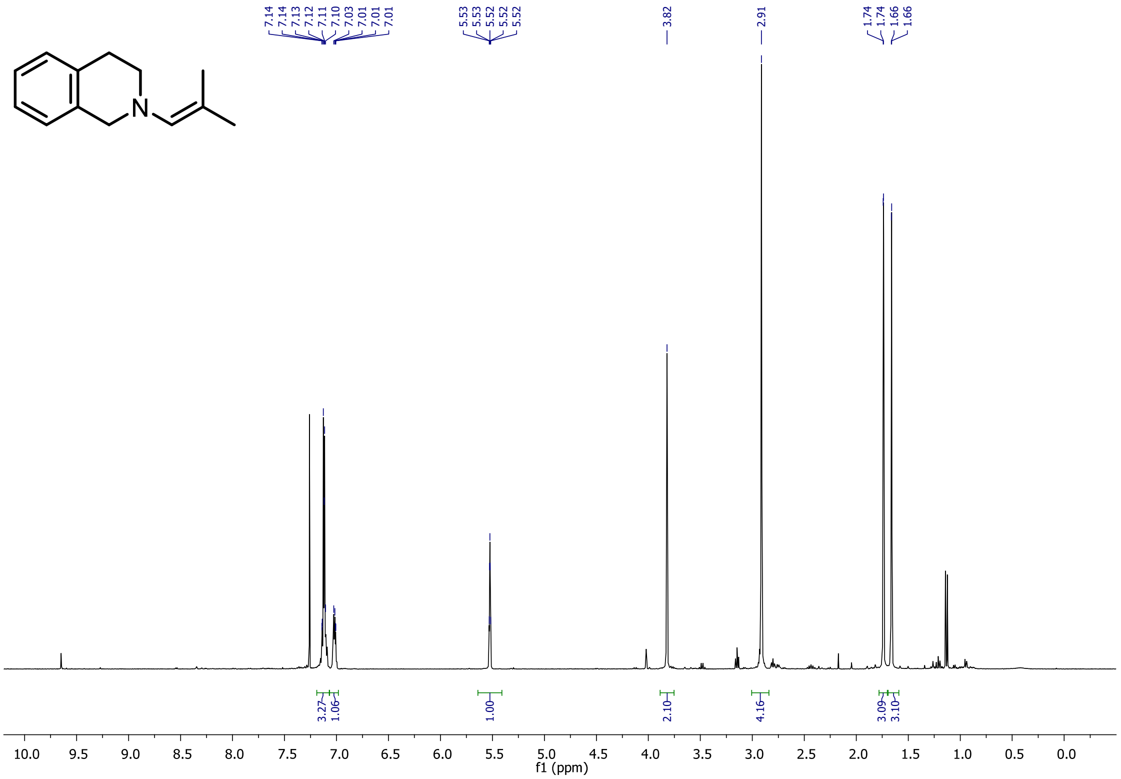

6.1.1.6 2f – 4-((*tert*-butyldimethylsilyl)oxy)-1-(2-methylprop-1-en-1-yl)piperidine – ^{1}H NMR (400 MHz)

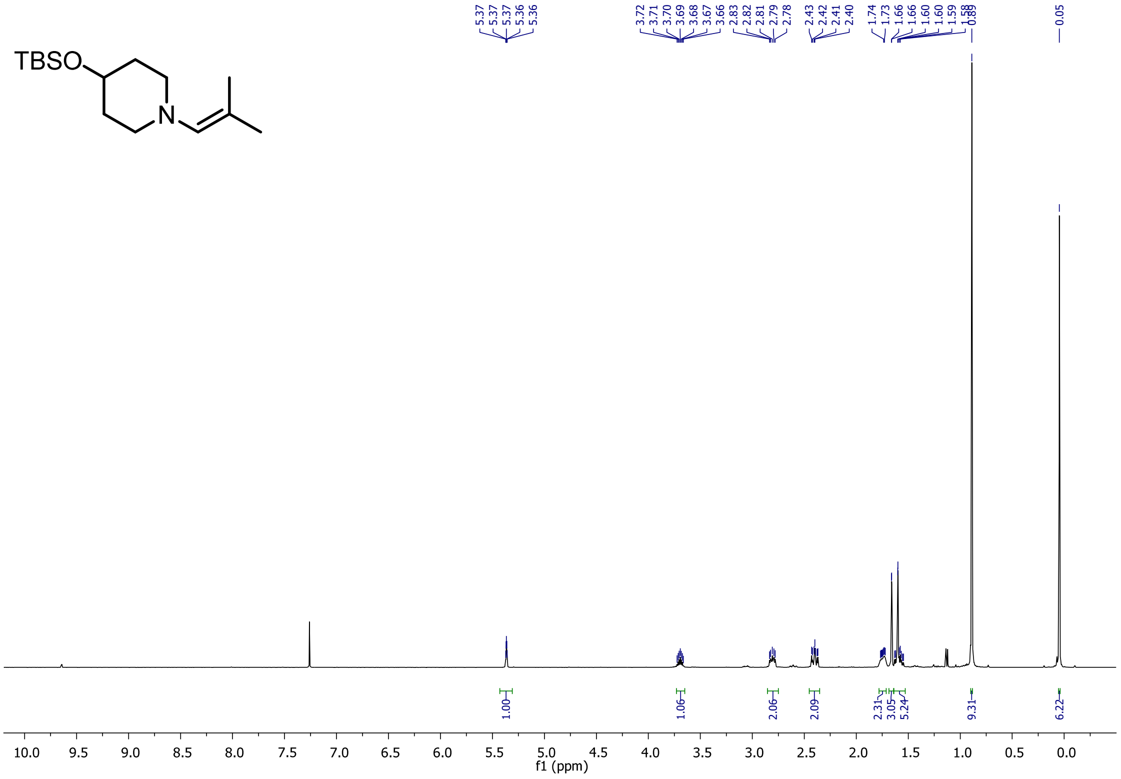

6.1.1.7 2g – 2-methyl-1-(2-methylprop-1-en-1-yl)piperidine – ^{1}H NMR (400 MHz)

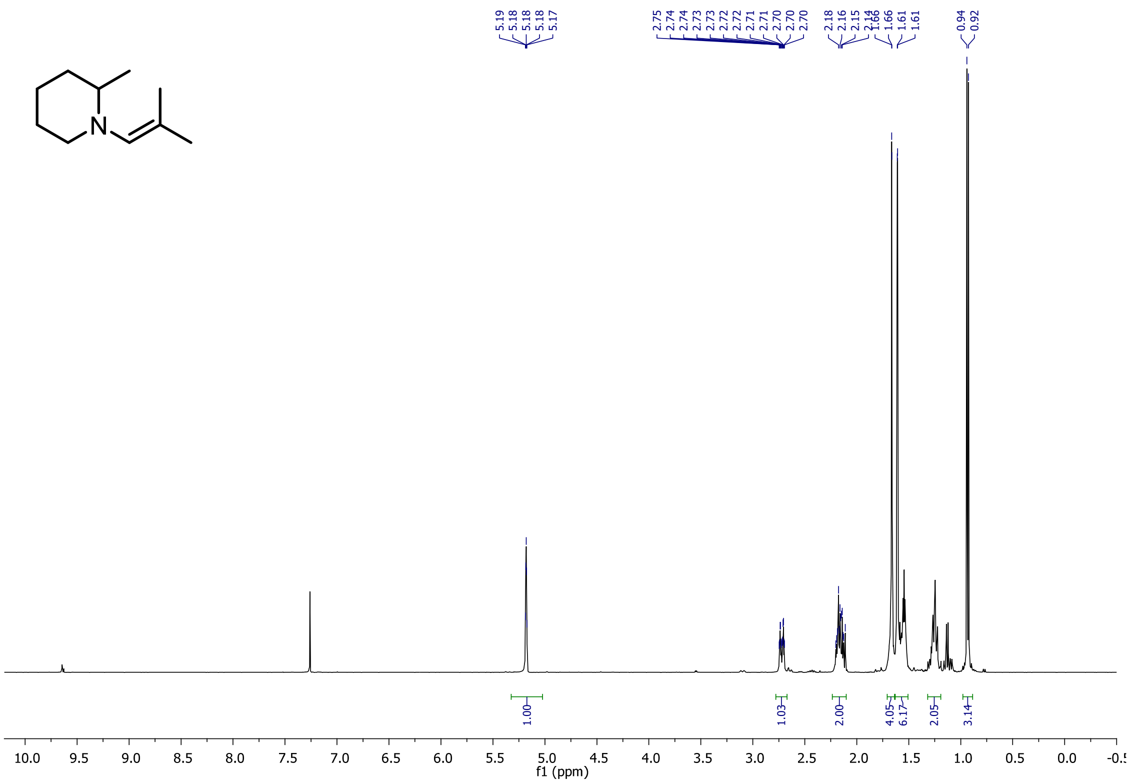

6.1.1.8 2h – N,N-diethyl-2-methylprop-1-en-1-amine – ^{1}H NMR (400 MHz)

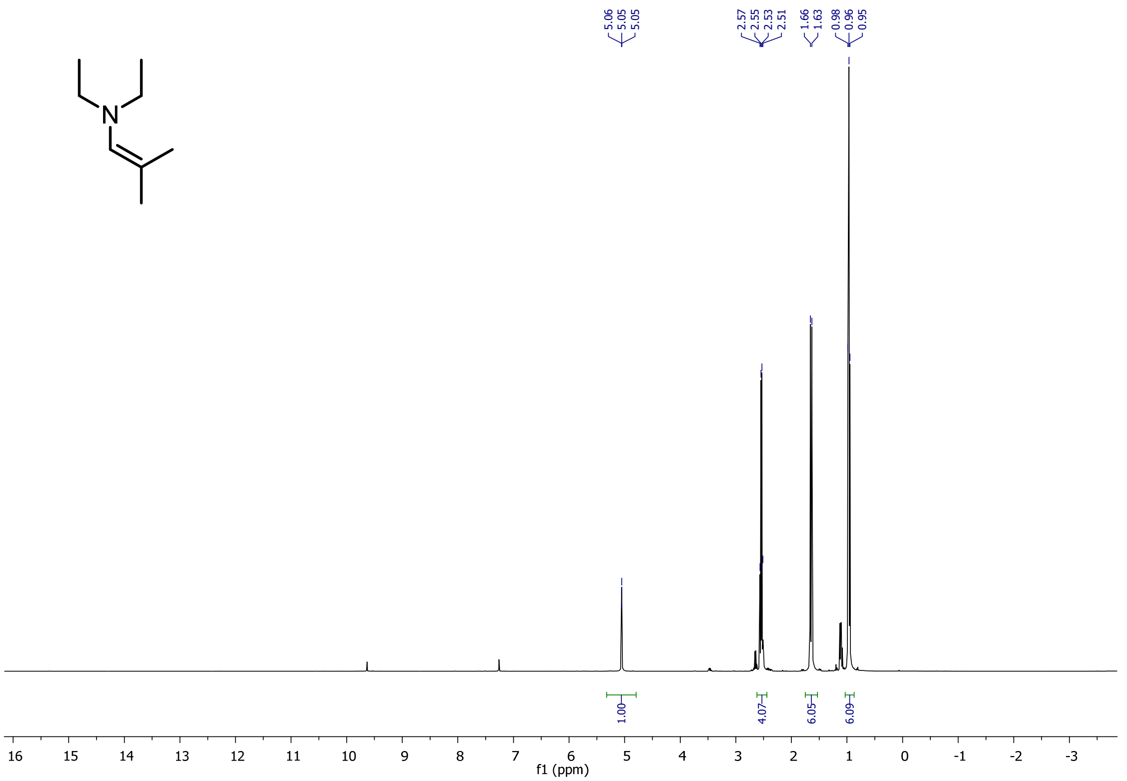

6.1.1.9 2i – (*E*)-/(*Z*)-1-(2-methylpent-1-en-1-yl)pyrrolidine – ^{1}H NMR (400 MHz)

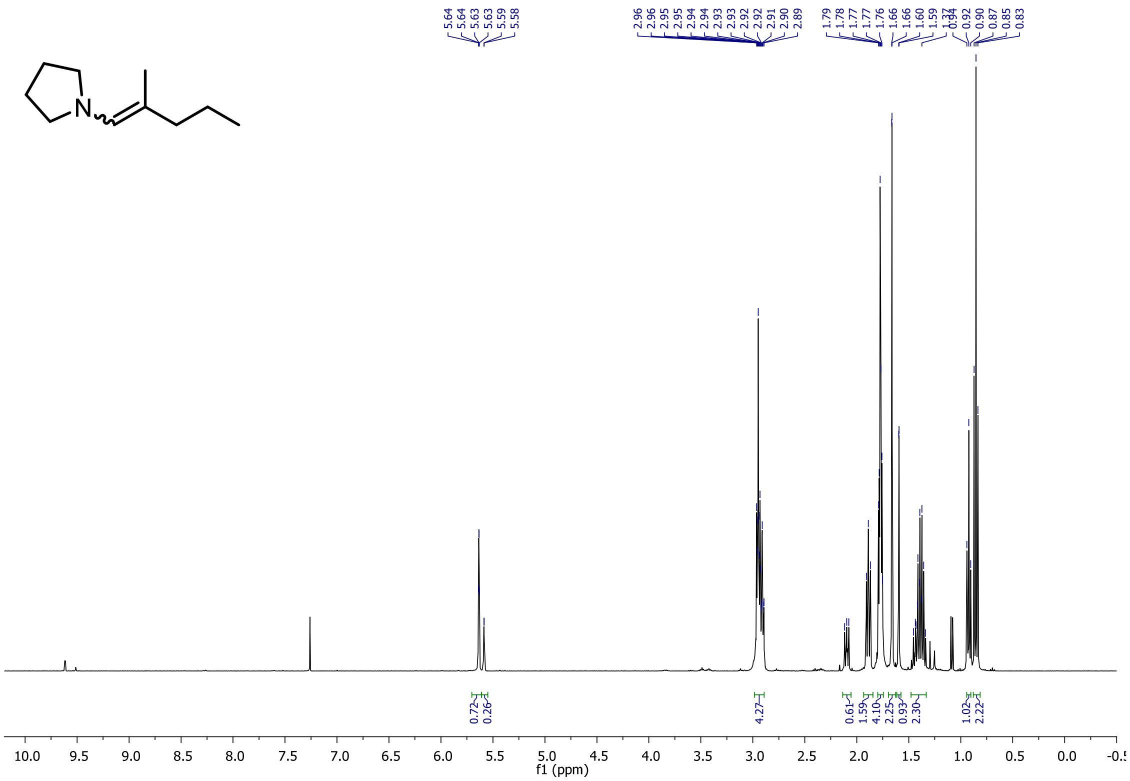

6.1.1.10 2j – (*E*)-/(*Z*)-1-(2,6-dimethylhepta-1,5-dien-1-yl)pyrrolidine – ^{1}H NMR (600 MHz)

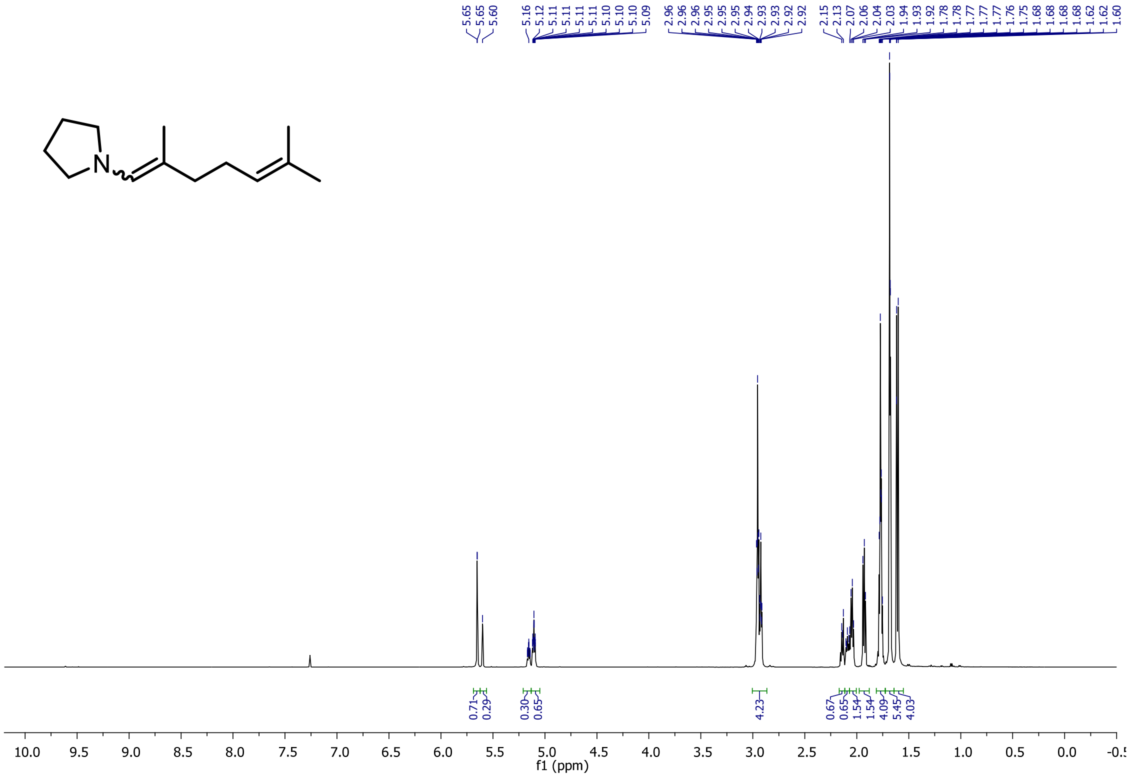

6.1.1.11 2k – (*E*)-1-(3-methylbut-1-en-1-yl)pyrrolidine – ^{1}H NMR (600 MHz)

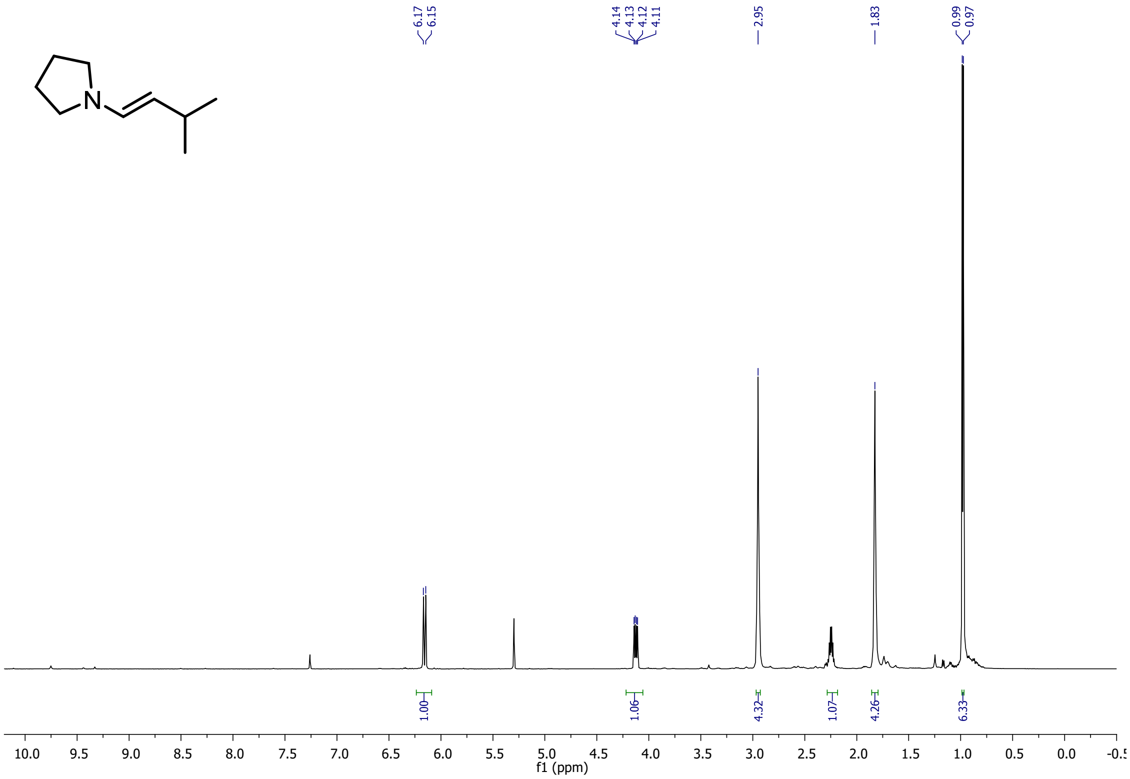

6.1.1.12 2l – 1-(cyclohexylidenemethyl)pyrrolidine – ^{1}H NMR (400 MHz)

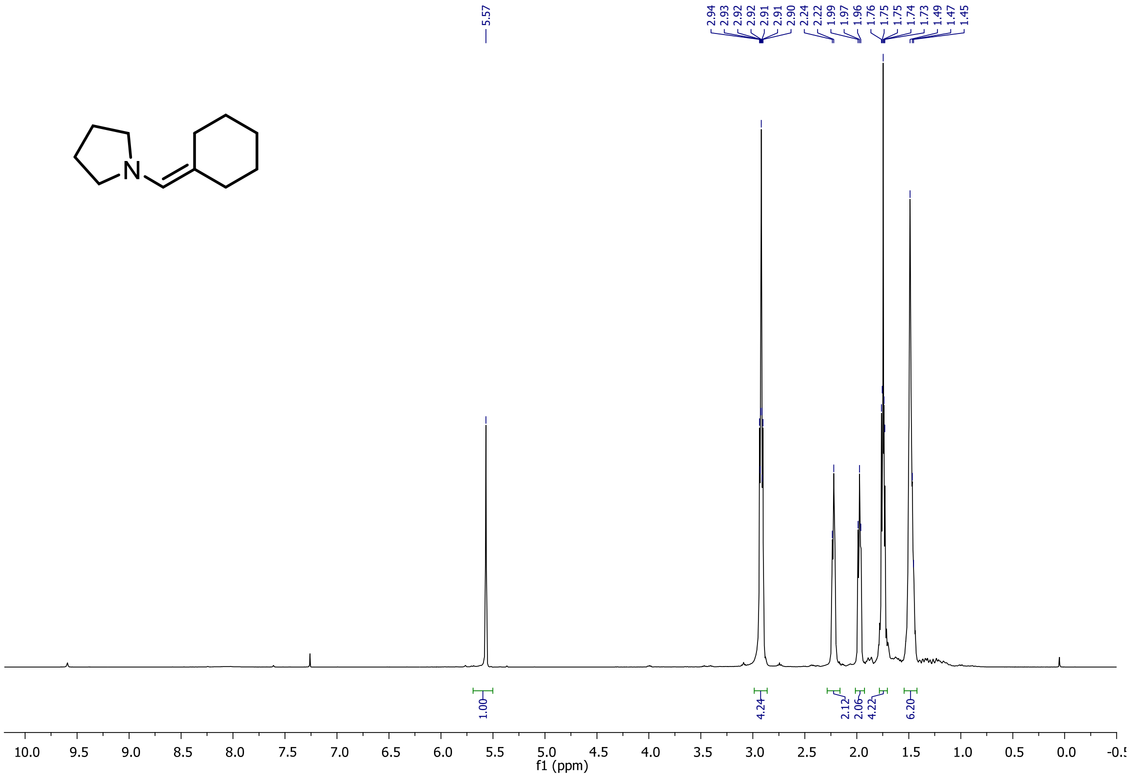

6.1.1.13 2m – (*E*)-1-(but-1-en-1-yl)pyrrolidine – ^{1}H NMR (400 MHz)

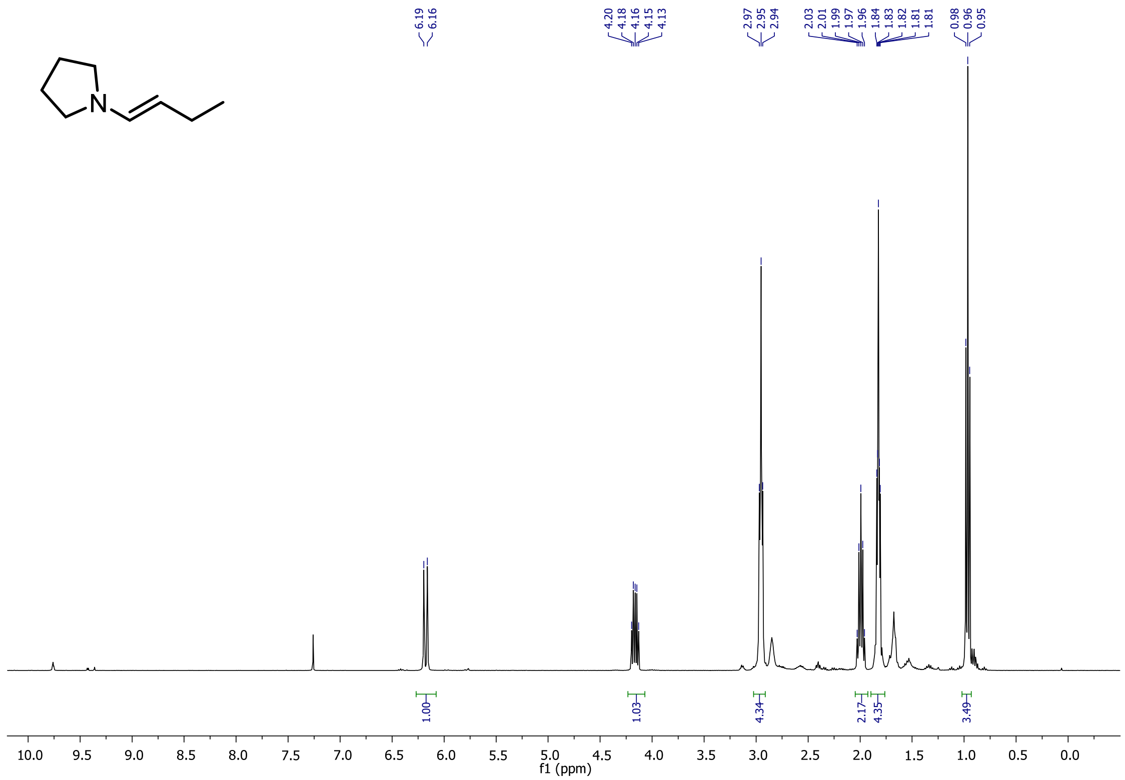

6.1.1.14 2n – (*E*)-1-(but-1-en-1-yl)piperidine – ^{1}H NMR (400 MHz)

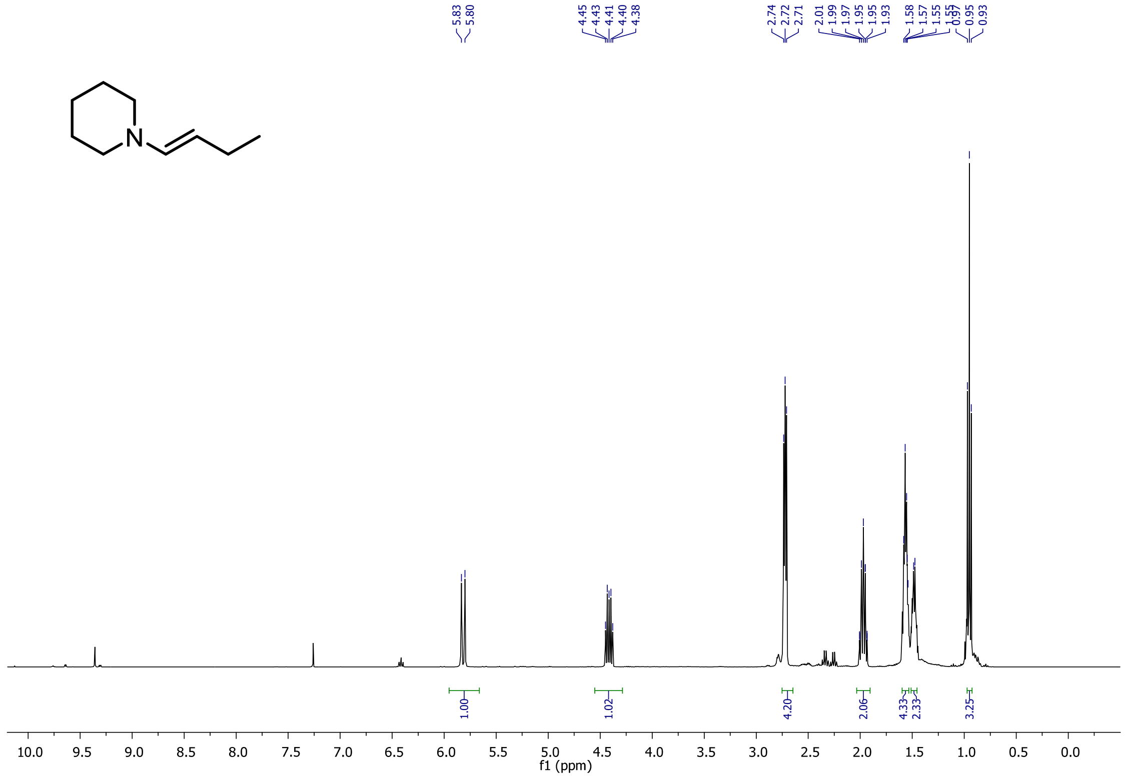

6.1.1.15 3u – *tert*-butyl (*E*)-2-(2-nitrovinyl)-1*H*-pyrrole-1-carboxylate – ^{1}H NMR (400 MHz)

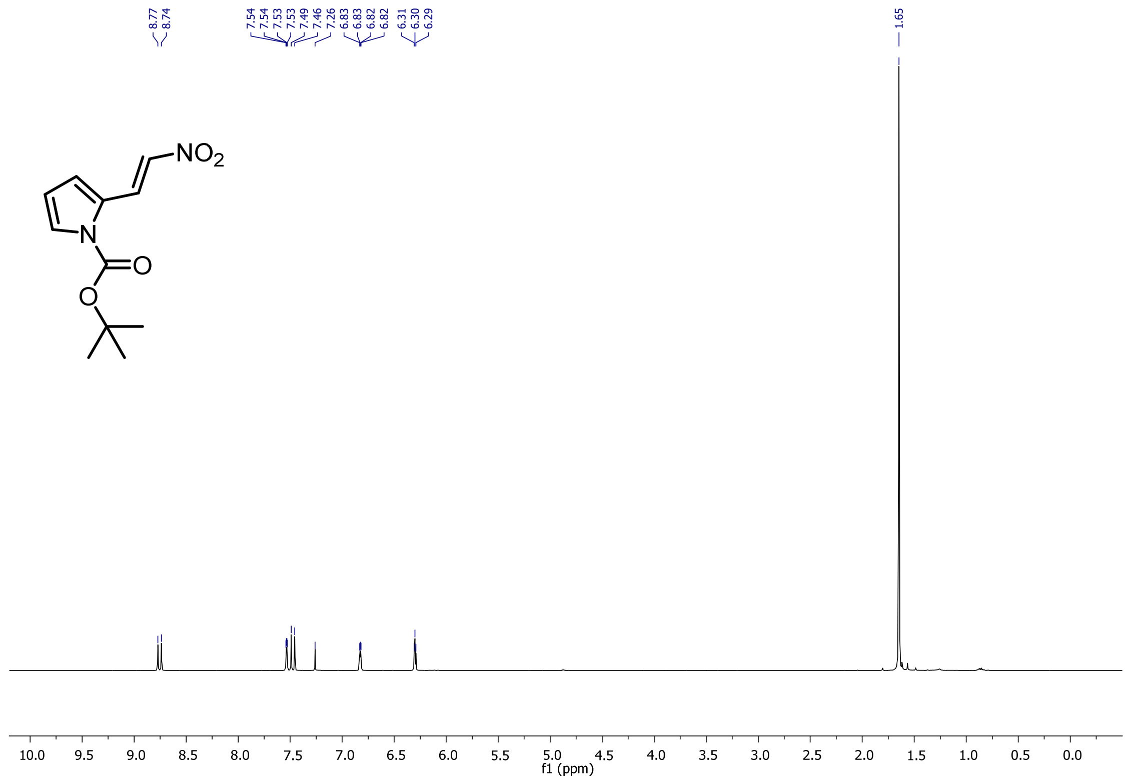

3u – *tert*-butyl (*E*)-2-(2-nitrovinyl)-1*H*-pyrrole-1-carboxylate – ^{13}C NMR (101 MHz)

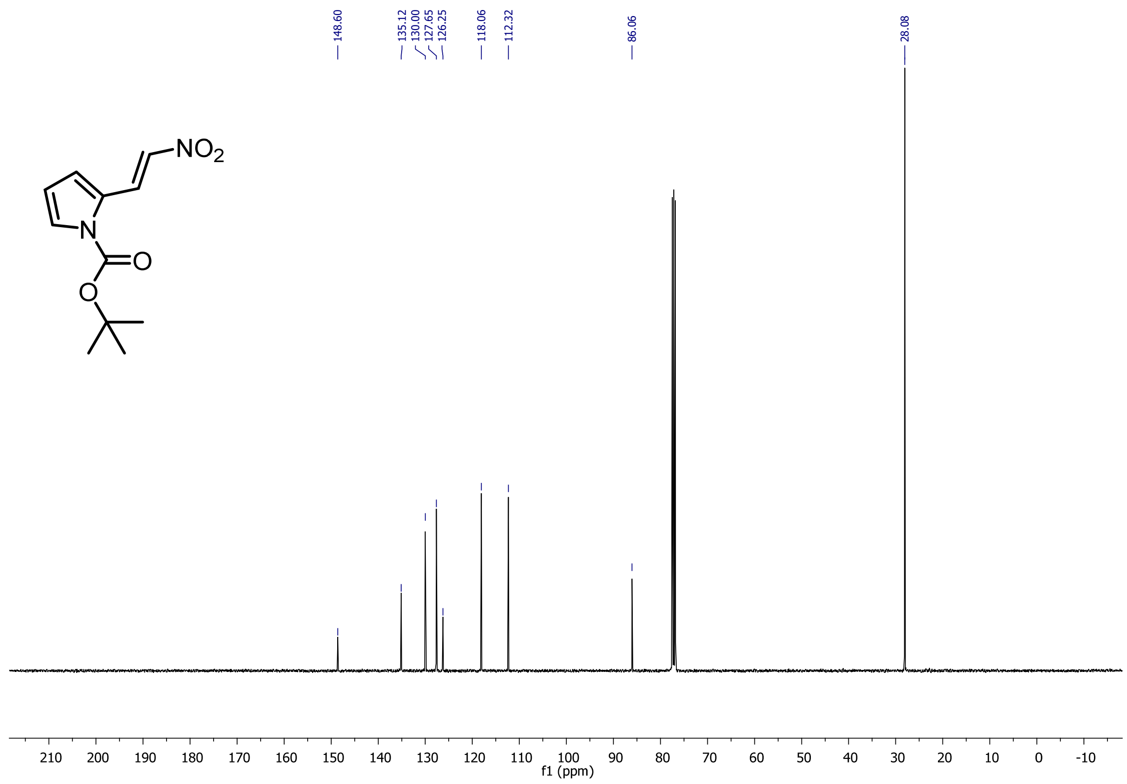

6.1.1.16 3x – (*E*)-(2-nitrovinyl)cyclopropane – ^{1}H NMR (400 MHz)

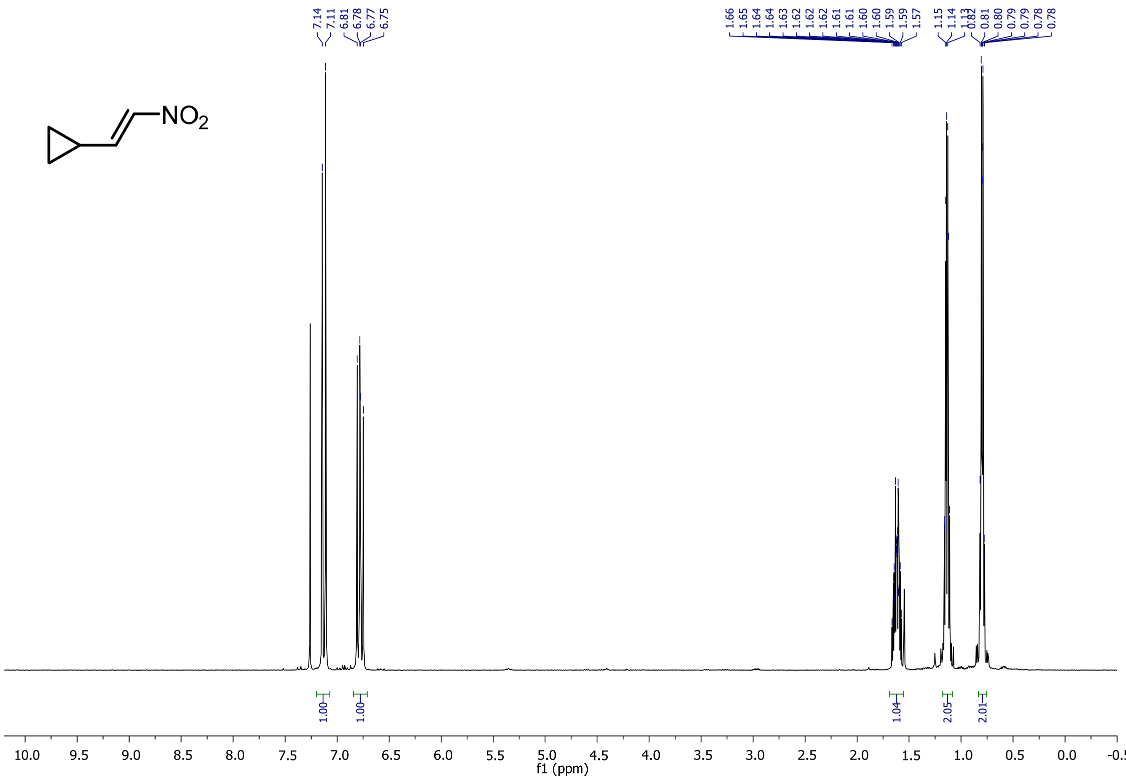

3x – (*E*)-(2-nitrovinyl)cyclopropane – ^{13}C NMR (101 MHz)

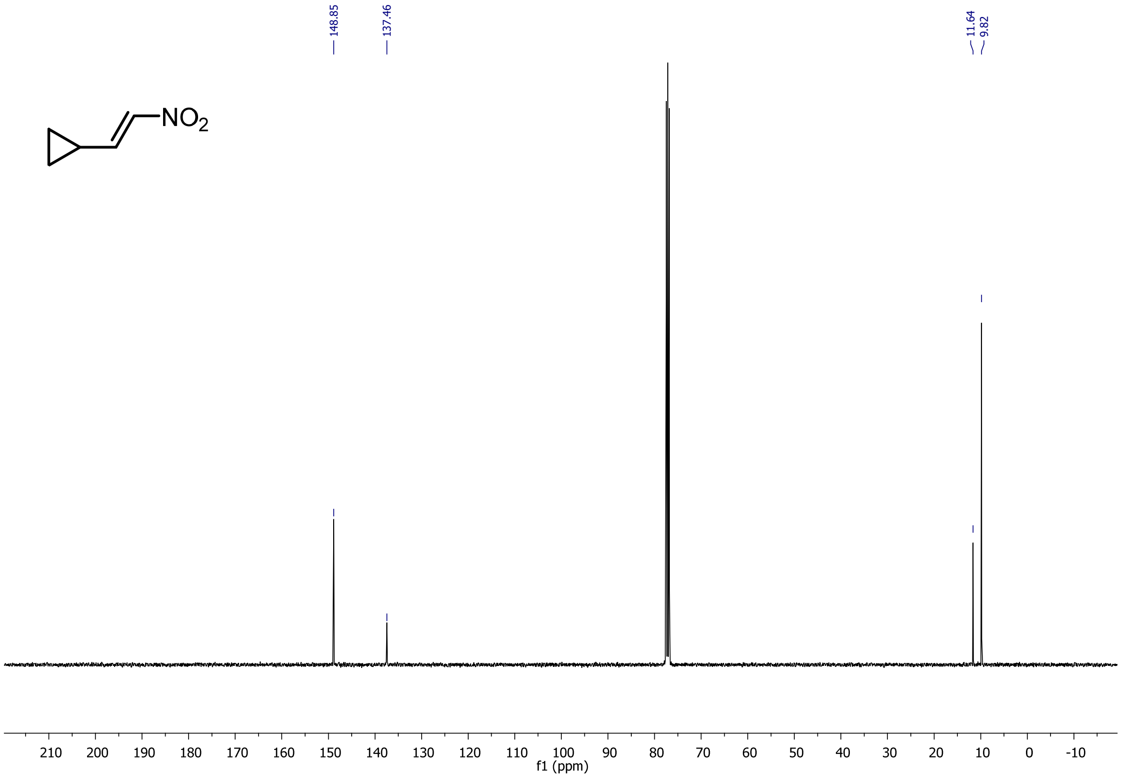

6.1.1.17 3y – ((1*E*,3*E*)-4-nitrobuta-1,3-dien-1-yl)benzene – ^{1}H NMR (400 MHz)

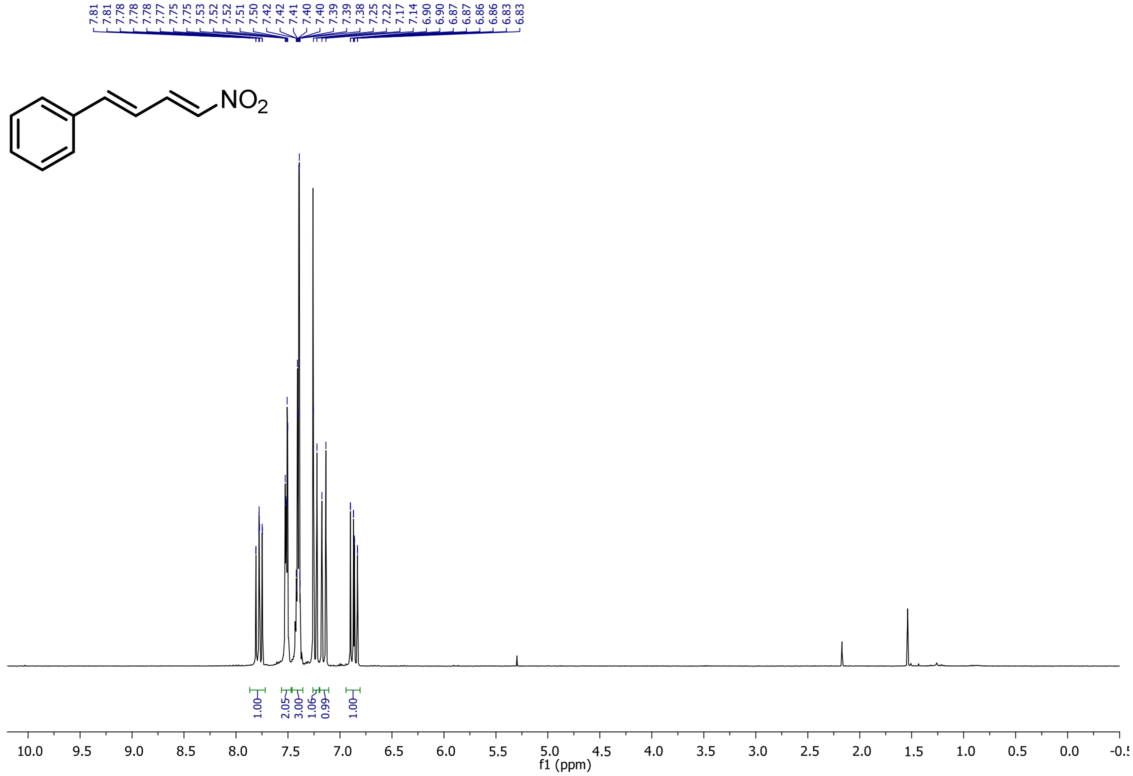

3y – ((1*E*,3*E*)-4-nitrobuta-1,3-dien-1-yl)benzene – ^{13}C NMR (101 MHz)

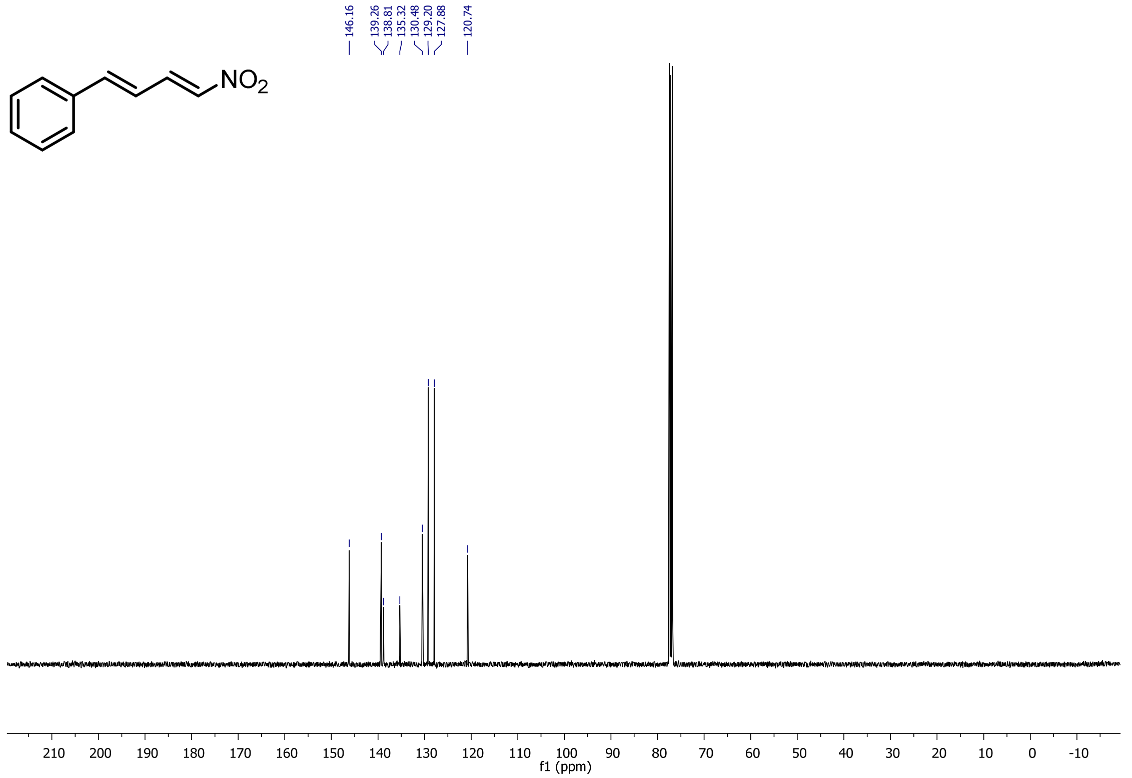

6.1.1.18 3z – (*E*)-(6-nitrohex-5-en-3-yn-1-yl)benzene – ^{1}H NMR (400 MHz)

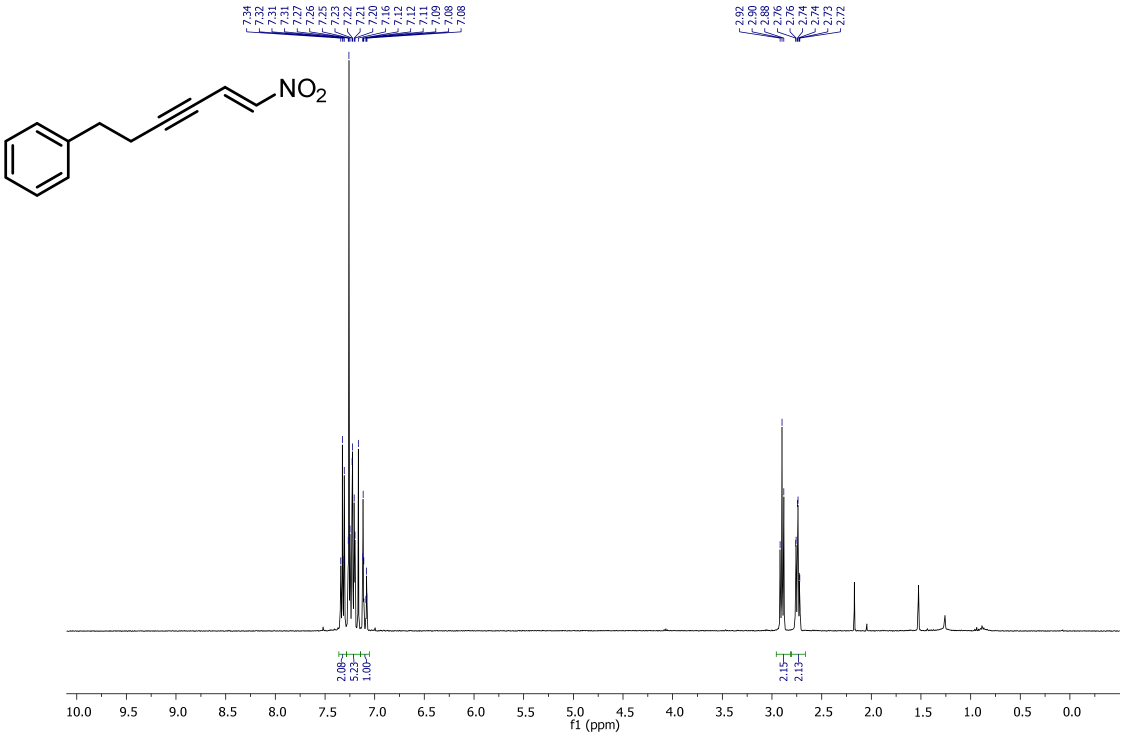

3z – (*E*)-(6-nitrohex-5-en-3-yn-1-yl)benzene – ^{13}C NMR (101 MHz)

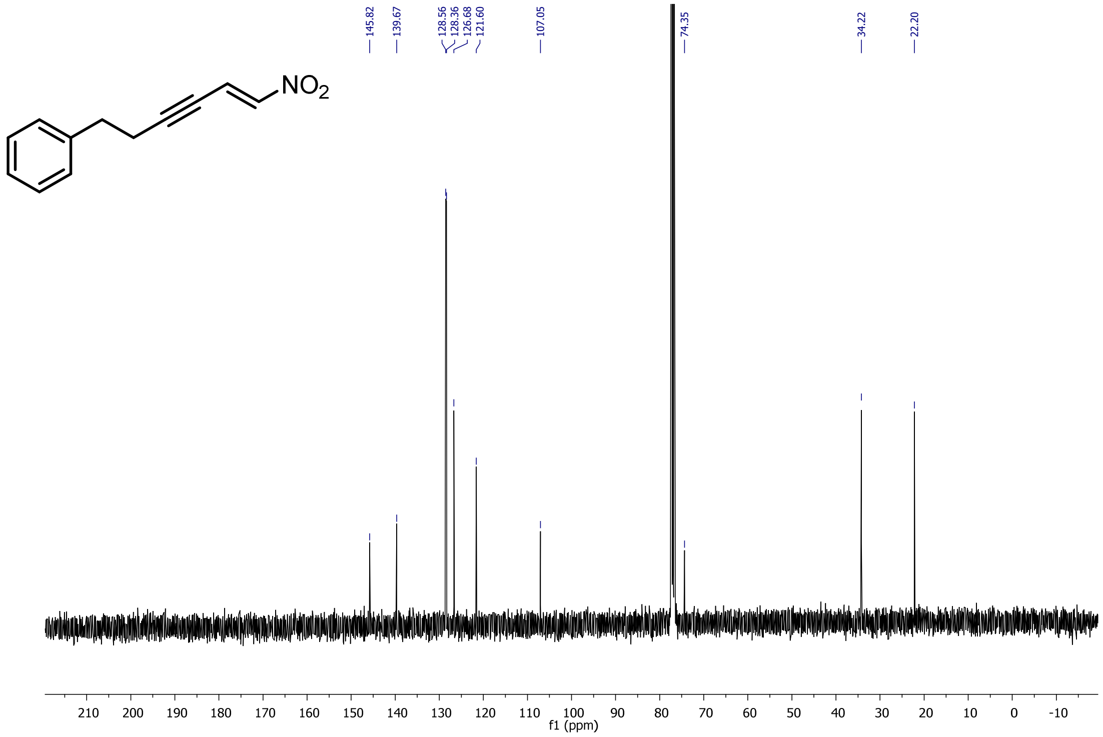

6.1.1.19 S1a – (*E*)-1,1,1-trifluoro-4-phenylbut-3-en-2-one – ^{1}H NMR (400 MHz)

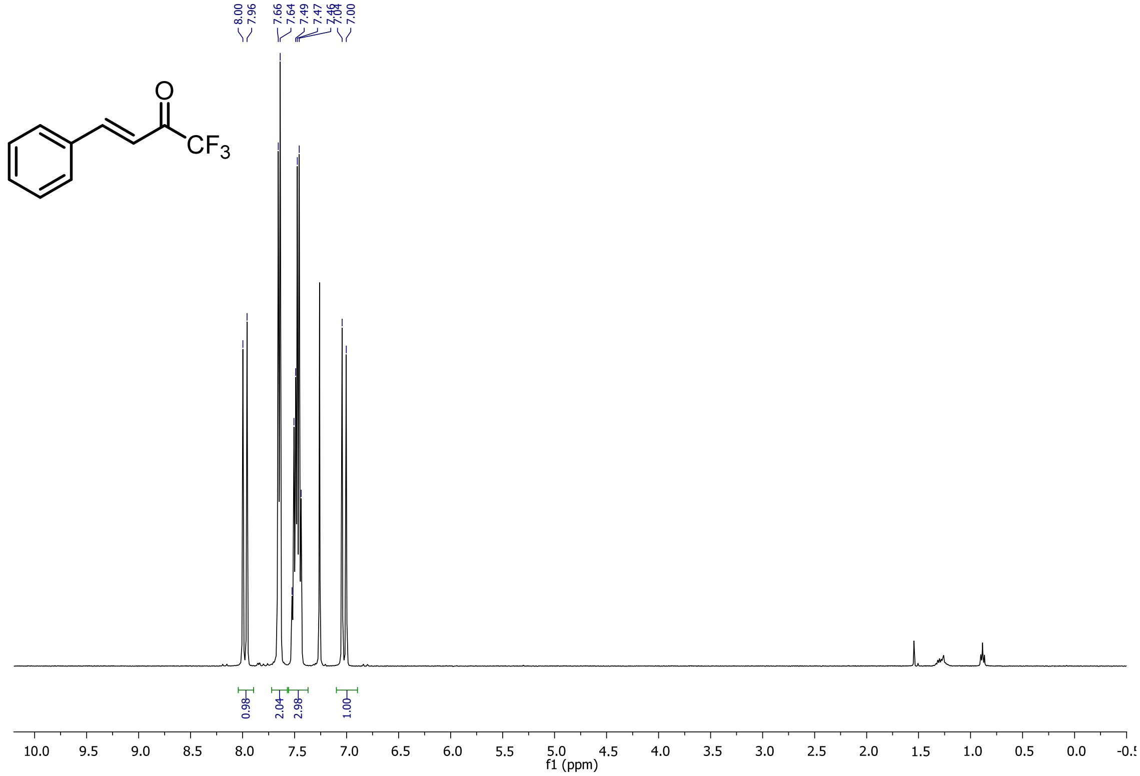

S1a – (*E*)-1,1,1-trifluoro-4-phenylbut-3-en-2-one – ^{13}C NMR (151 MHz)

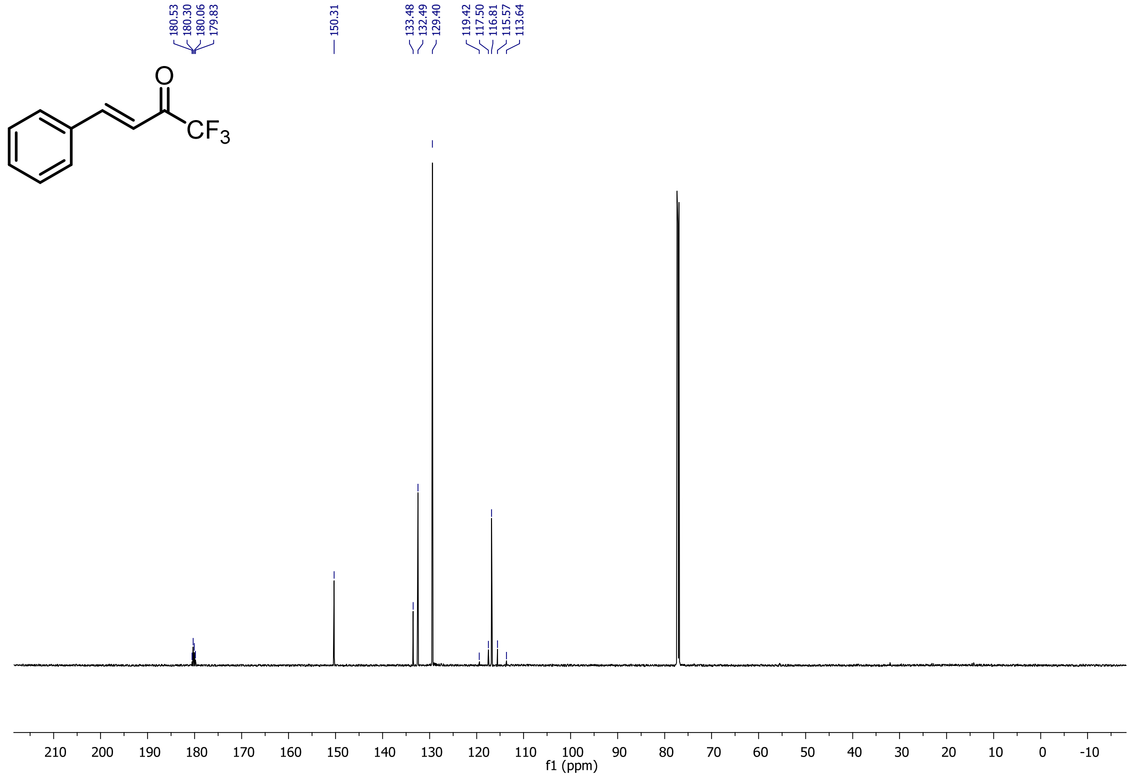

S1a – (*E*)-1,1,1-trifluoro-4-phenylbut-3-en-2-one – ^{19}F NMR (565 MHz)

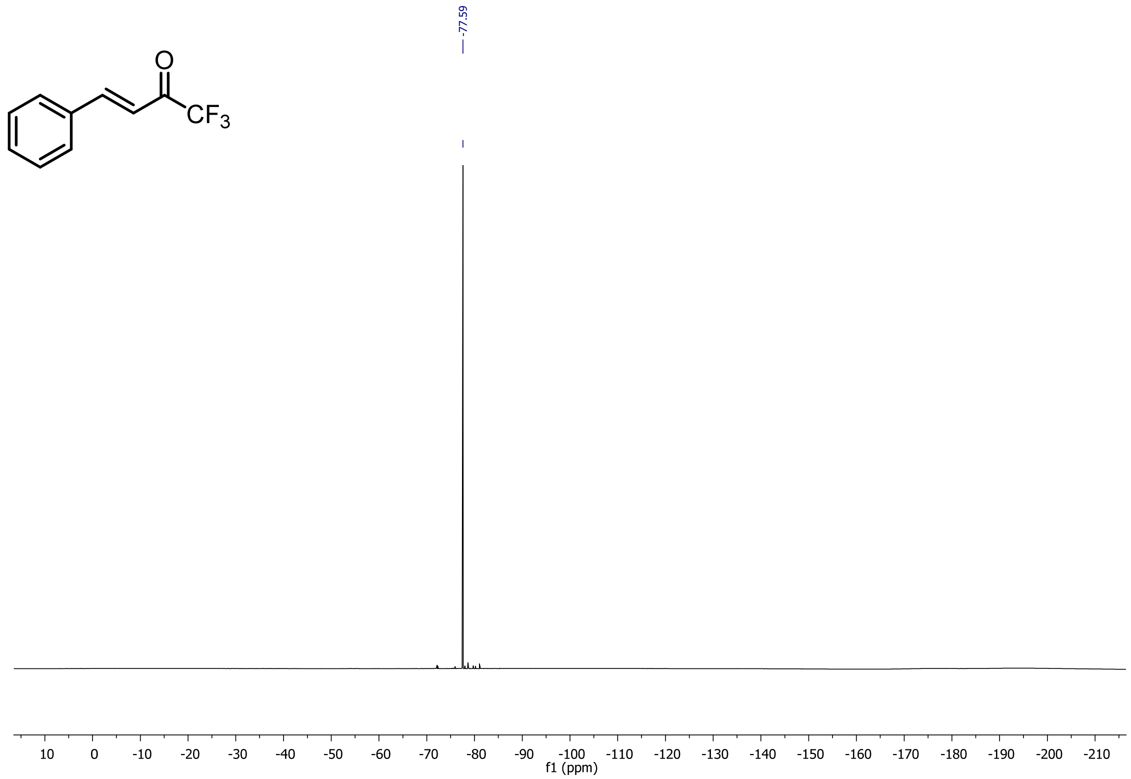

6.1.1.20 S1b – (3*E*,5*E*)-1,1,1-trifluoro-6-phenylhexa-3,5-dien-2-one – ^{1}H NMR (600 MHz)

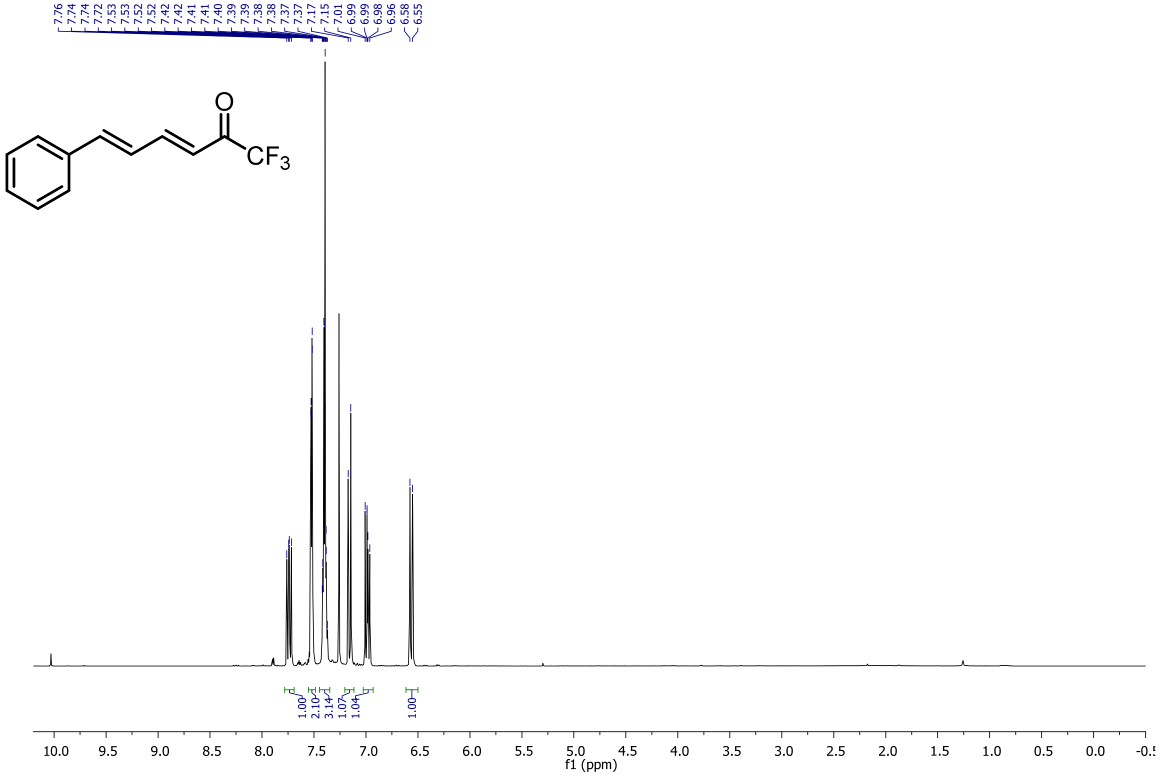

S1b – (3*E*,5*E*)-1,1,1-trifluoro-6-phenylhexa-3,5-dien-2-one – ^{13}C NMR (151 MHz)

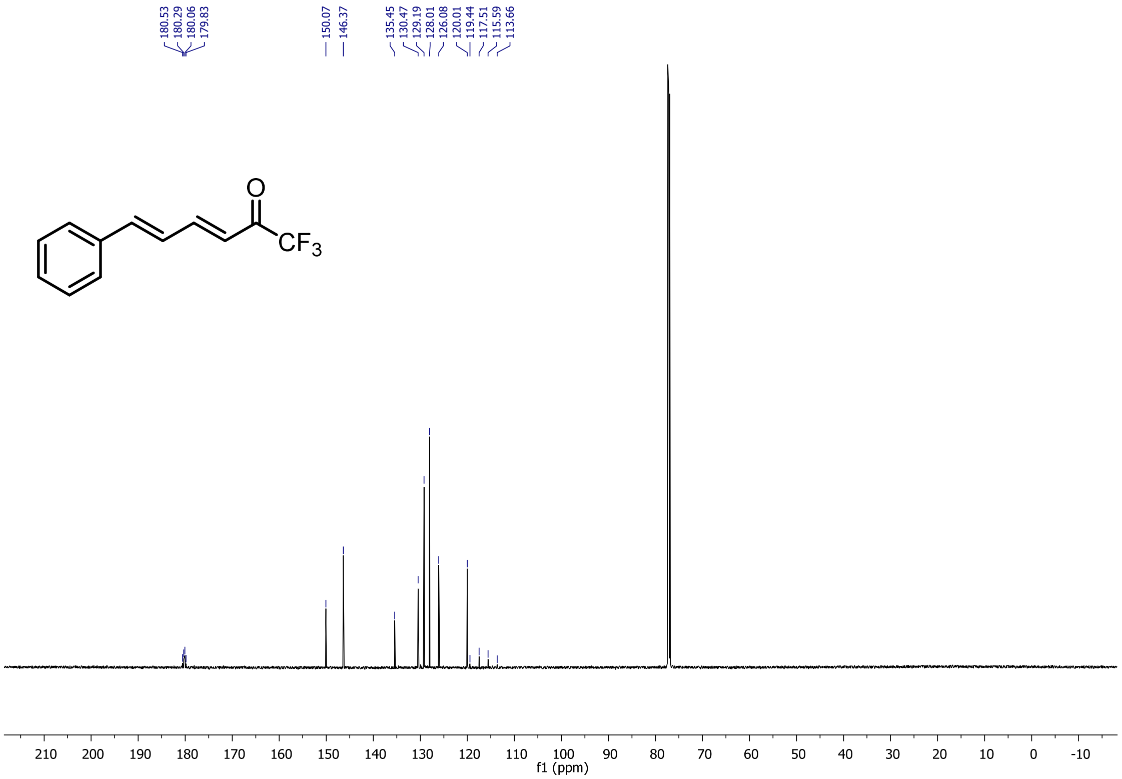

S1b – (3*E*,5*E*)-1,1,1-trifluoro-6-phenylhexa-3,5-dien-2-one – ^{19}F NMR (565 MHz)

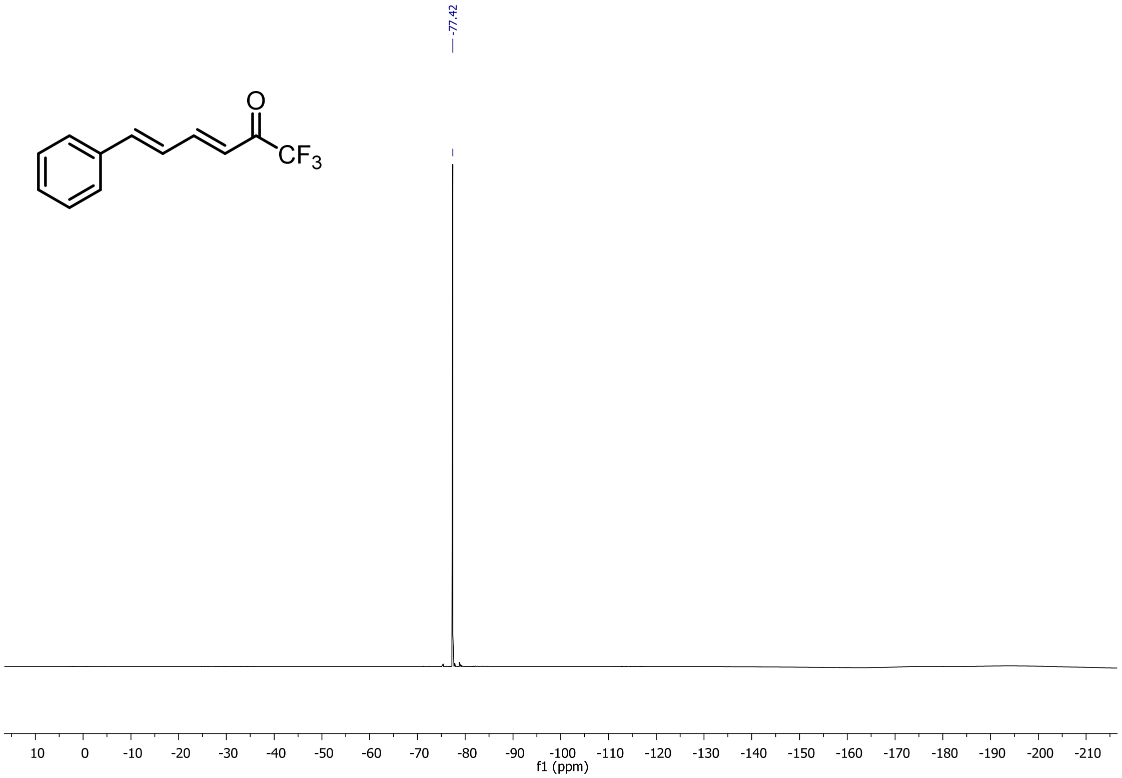

6.1.1.21 1c–THF – tris(2,6-difluorophenyl)borane-THF adduct – ^{1}H NMR (600 MHz, Tol-d_8)

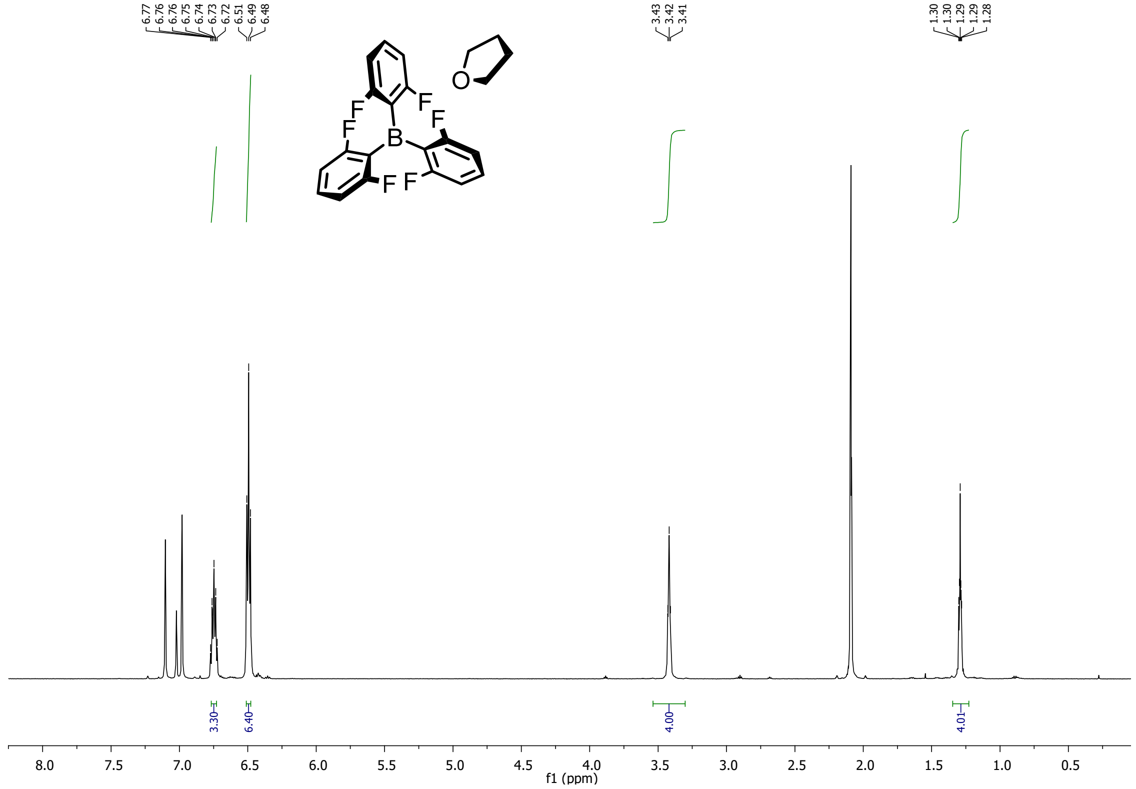

1c–THF – tris(2,6-difluorophenyl)borane-THF adduct – ^{13}C NMR (151 MHz, Tol-d_8)

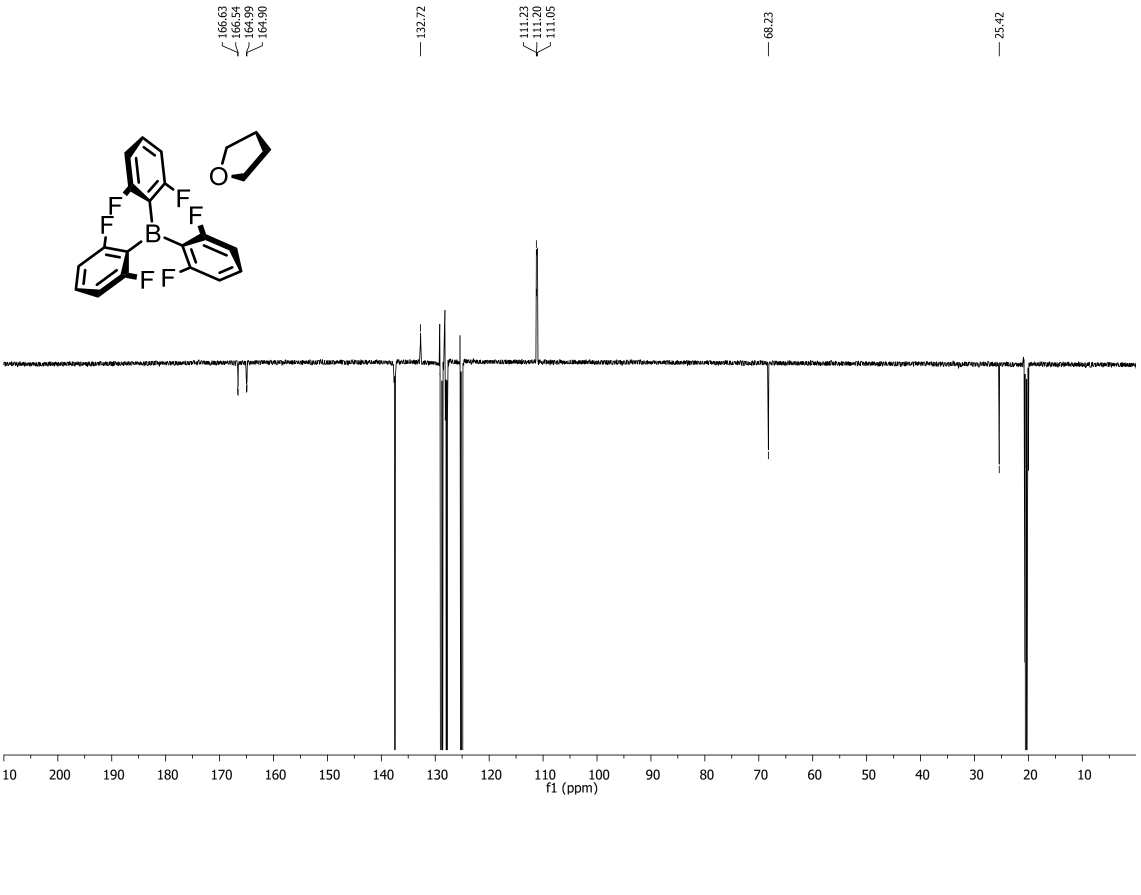

6.1.1.22 1c – tris(2,6-difluorophenyl)borane – ^{1}H NMR (600 MHz, Tol-d$_8$)

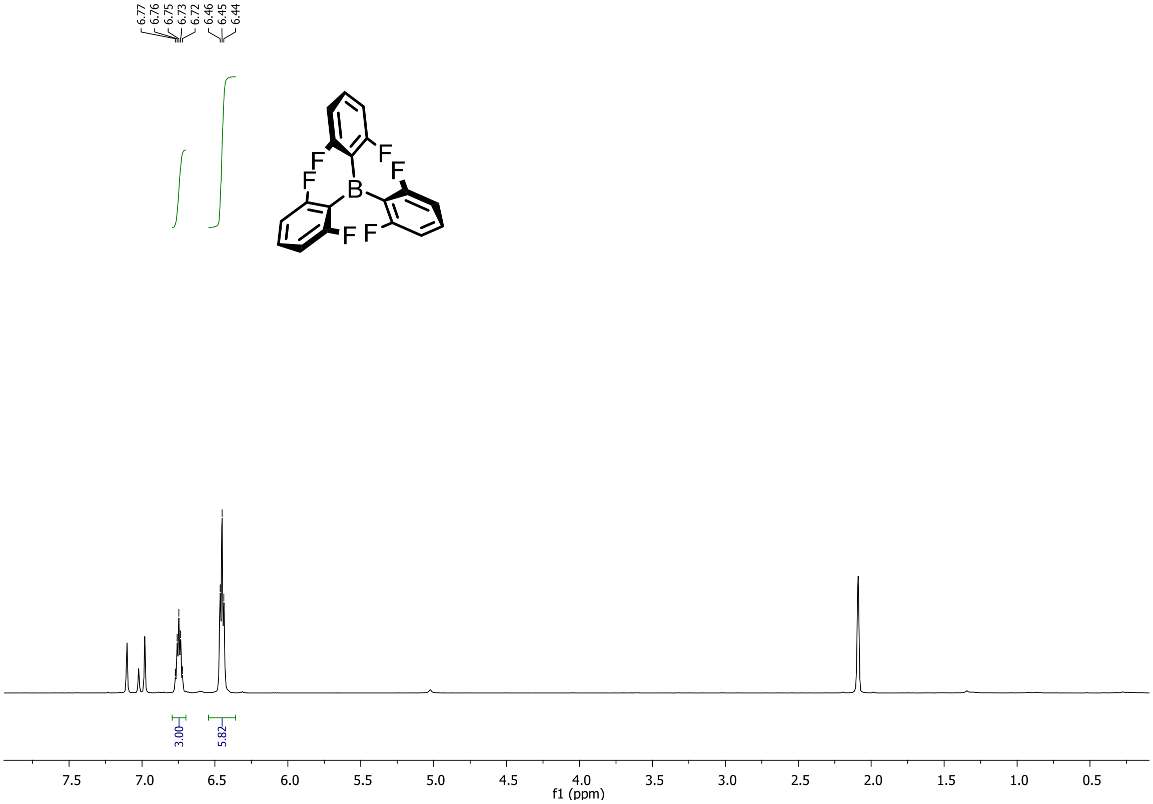

1c – tris(2,6-difluorophenyl)borane – ^{13}C NMR (151 MHz, Tol-d$_8$)

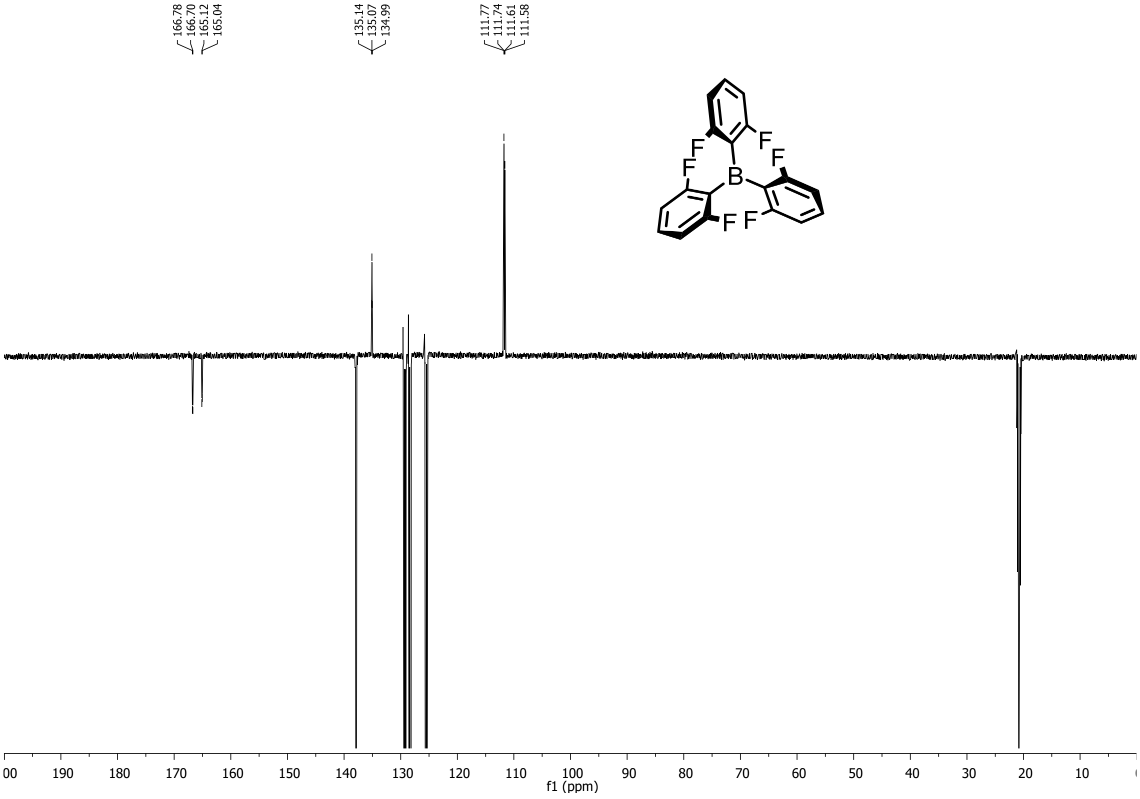

6.1.1.23 1c–H – tetrabutylammonium tris(2,6-difluorophenyl)hydroborate – ^{1}H NMR (600 MHz, $CDCl_3$)

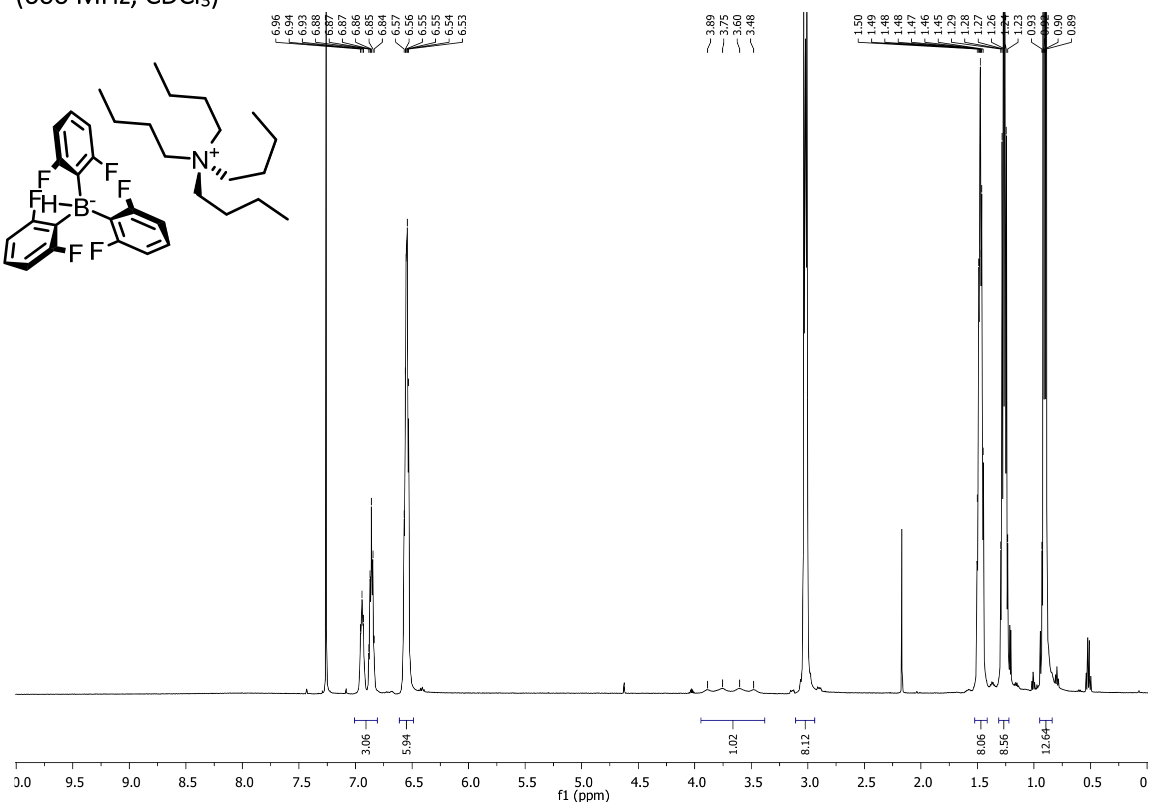

1c–H – tetrabutylammonium tris(2,6-difluorophenyl)hydroborate – ^{13}C NMR (151 MHz, $CDCl_3$)

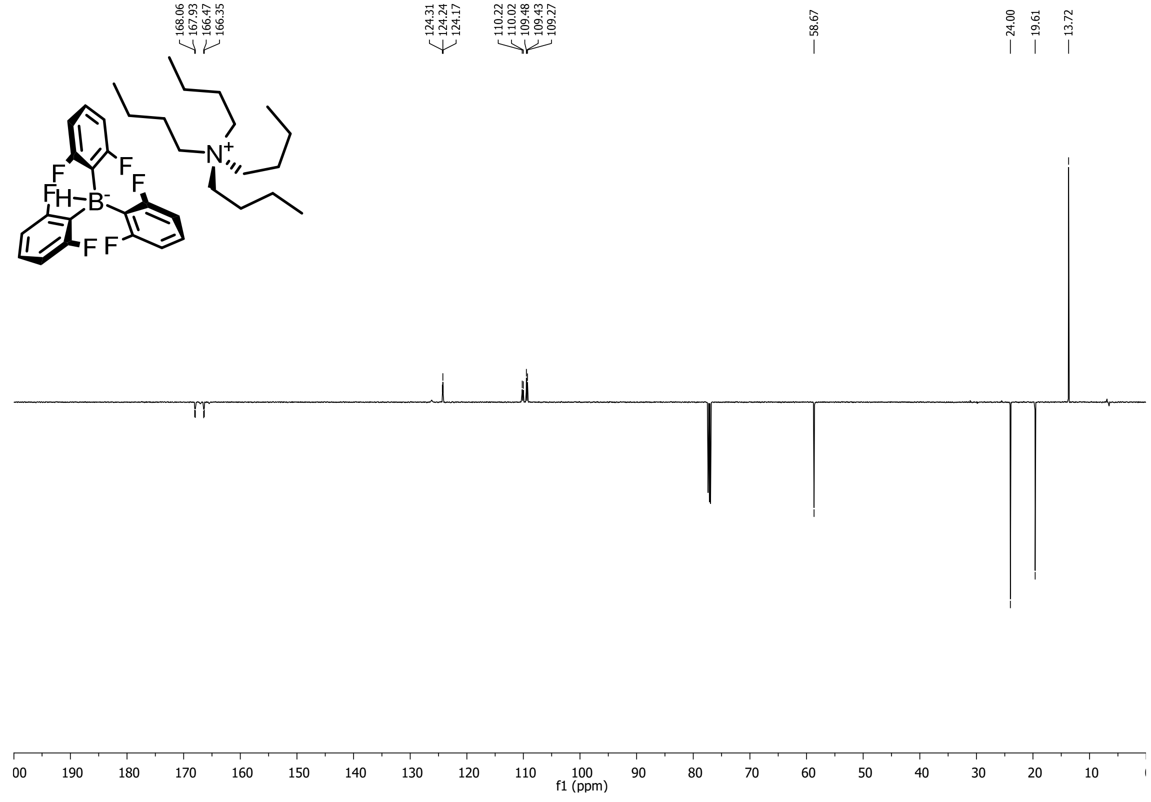

6.1.1.24 4a – 1-(2,2-dimethyl-4-nitro-3-phenylcyclobutyl)pyrrolidine – ^{1}H NMR (400 MHz, $CDCl_3$)

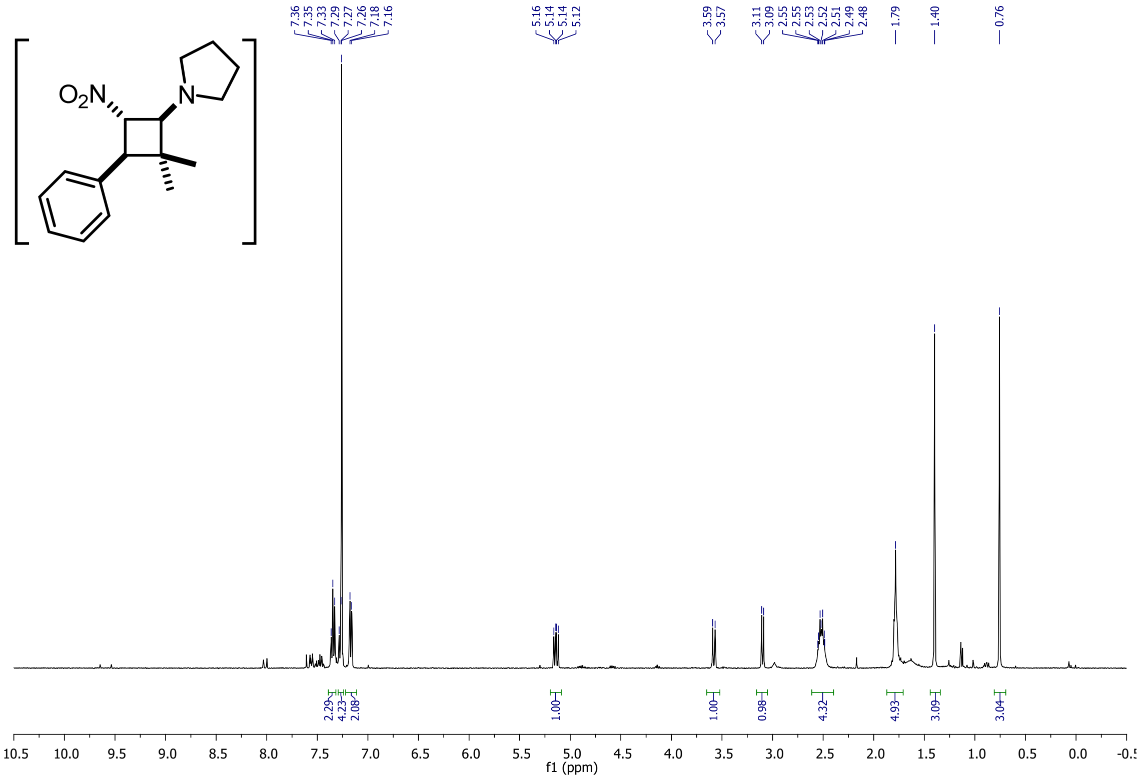

6.1.1.25 5a – **6,6-dimethyl-8-nitro-7-phenyloctahydroindolizine** – ^{1}H NMR (600 MHz, $CDCl_3$)

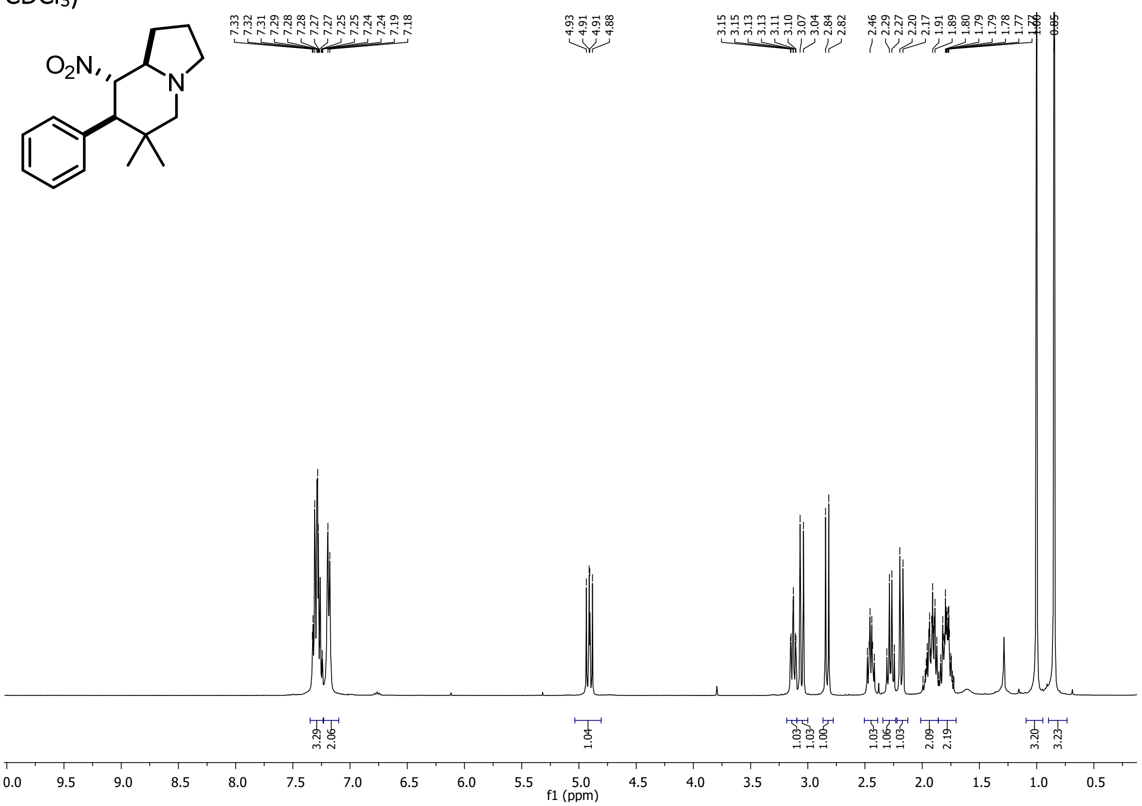

5a – 6,6-dimethyl-8-nitro-7-phenyloctahydroindolizine – ^{13}C NMR (151 MHz, $CDCl_3$)

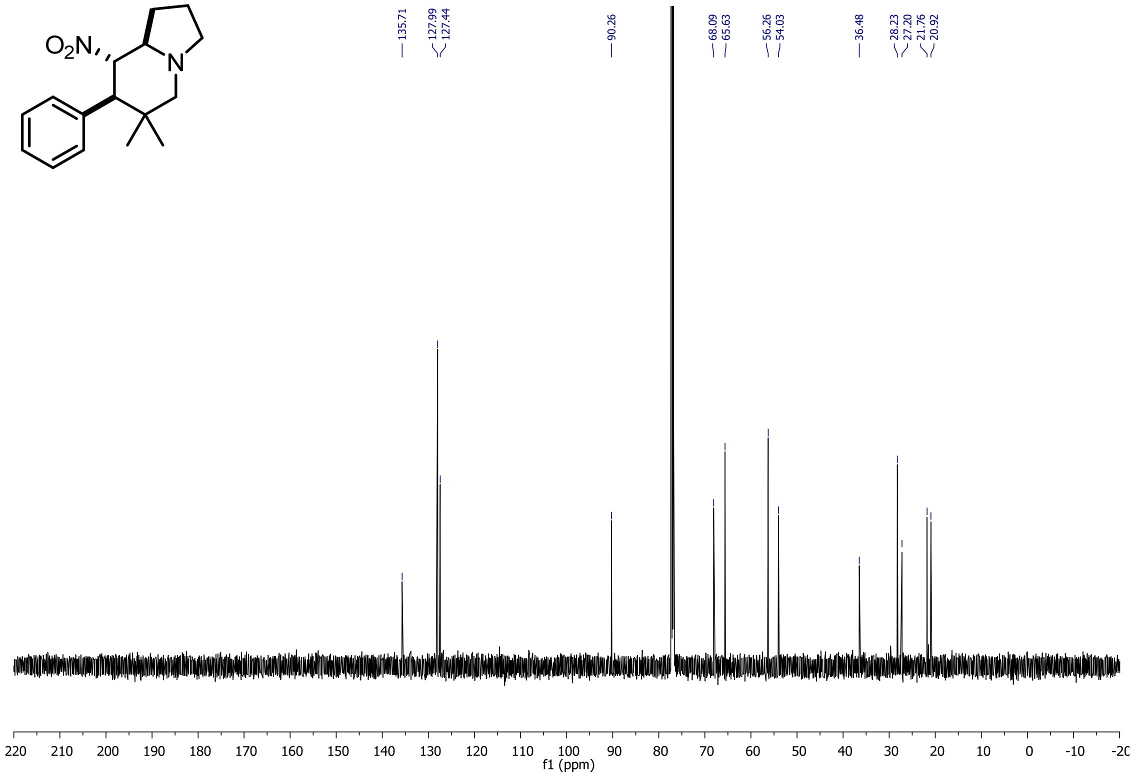

6.1.1.26 5b – 6,6-dimethyl-8-nitro-7-phenyloctahydroindolizine – ^{1}H NMR (600 MHz, $CDCl_3$)

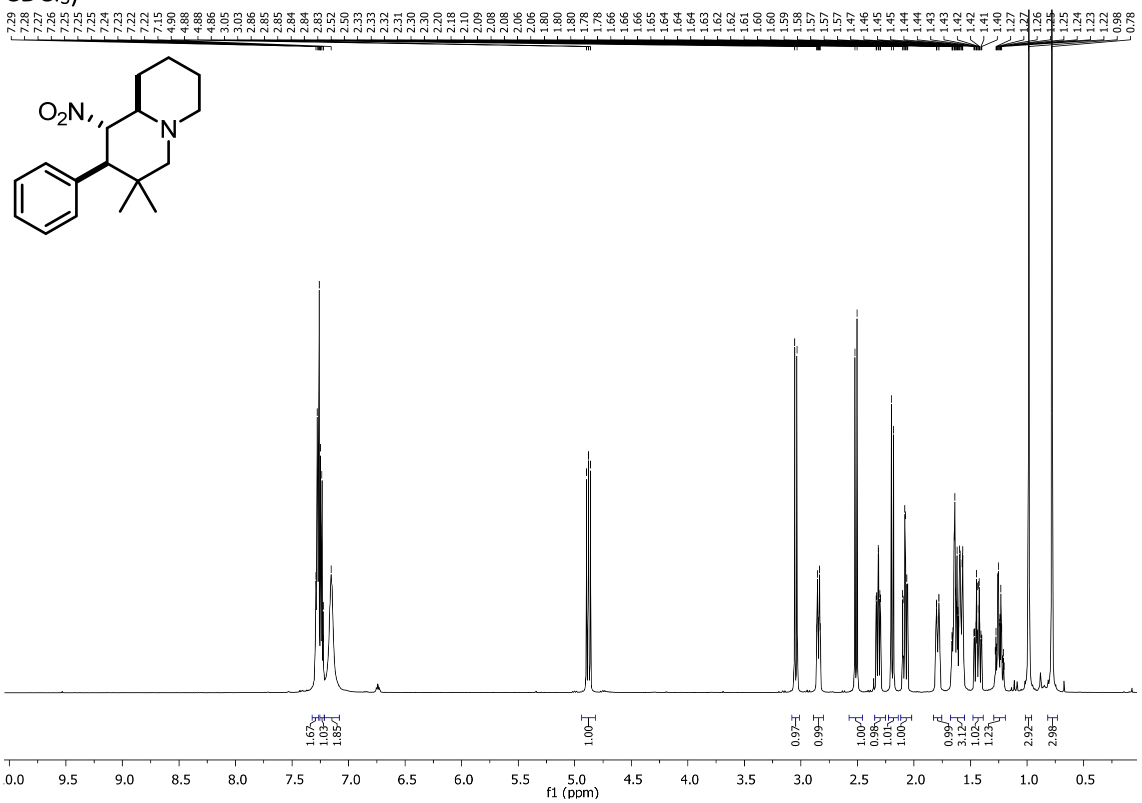

5b – 6,6-dimethyl-8-nitro-7-phenyloctahydroindolizine – ^{13}C NMR (151 MHz, $CDCl_3$)

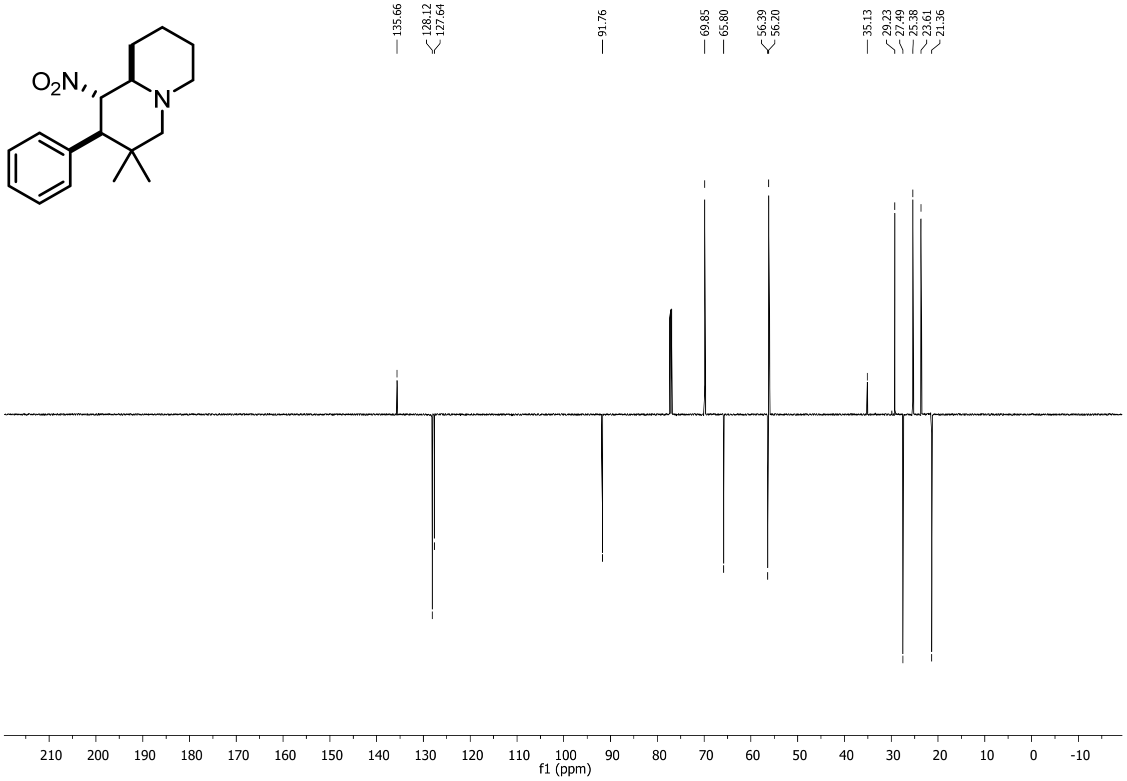

6.1.1.27 5c – 3,3-dimethyl-1-nitro-2-phenyldecahydropyrido[1,2-a]azepine – ^{1}H NMR (600 MHz, $CDCl_3$)

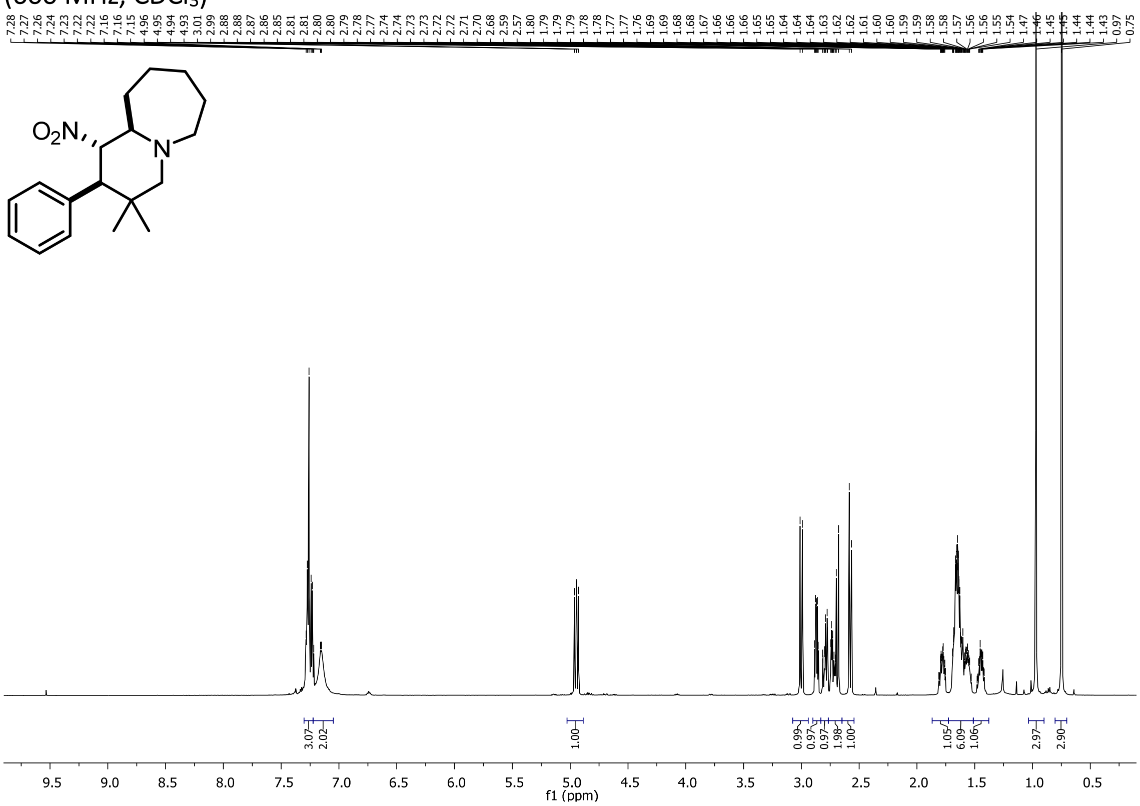

5c – 3,3-dimethyl-1-nitro-2-phenyldecahydropyrido[1,2-a]azepine – ^{13}C NMR (151 MHz, $CDCl_3$)

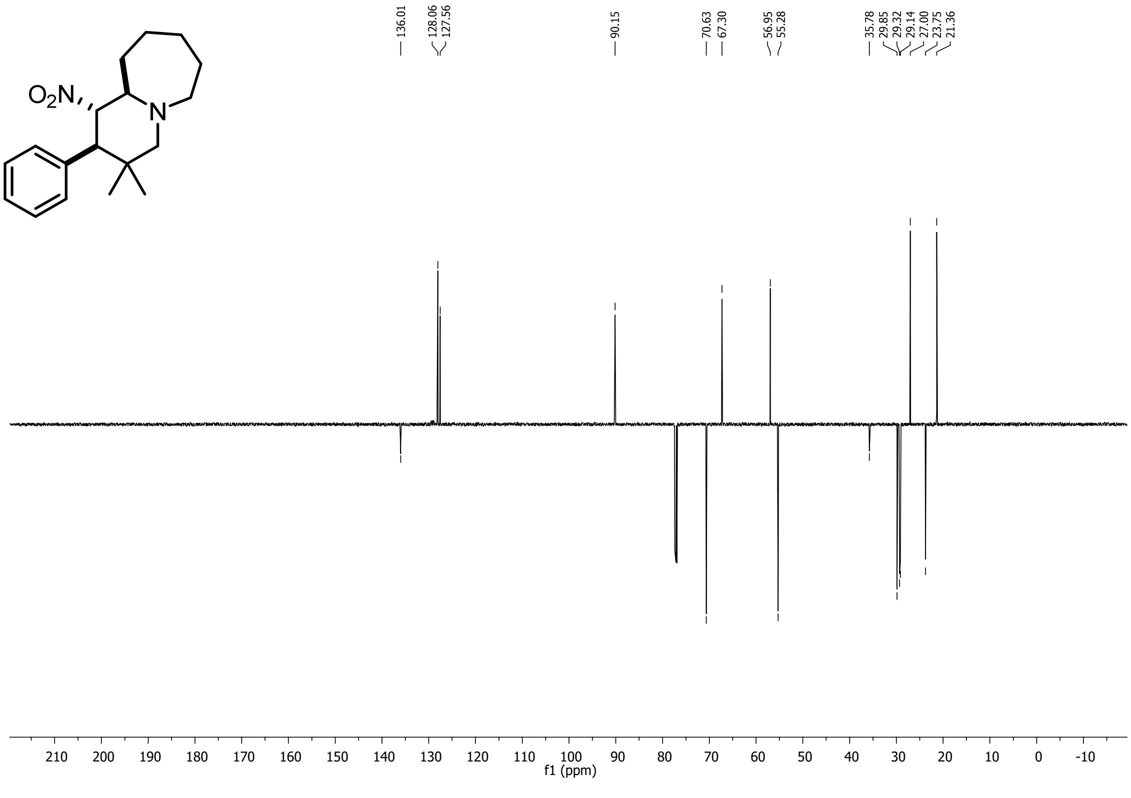

6.1.1.28 5d – 3,3-dimethyl-1-nitro-2-phenyldodecahydropyrido[2,1-a]isoindole – ^{1}H NMR (600 MHz, $CDCl_3$)

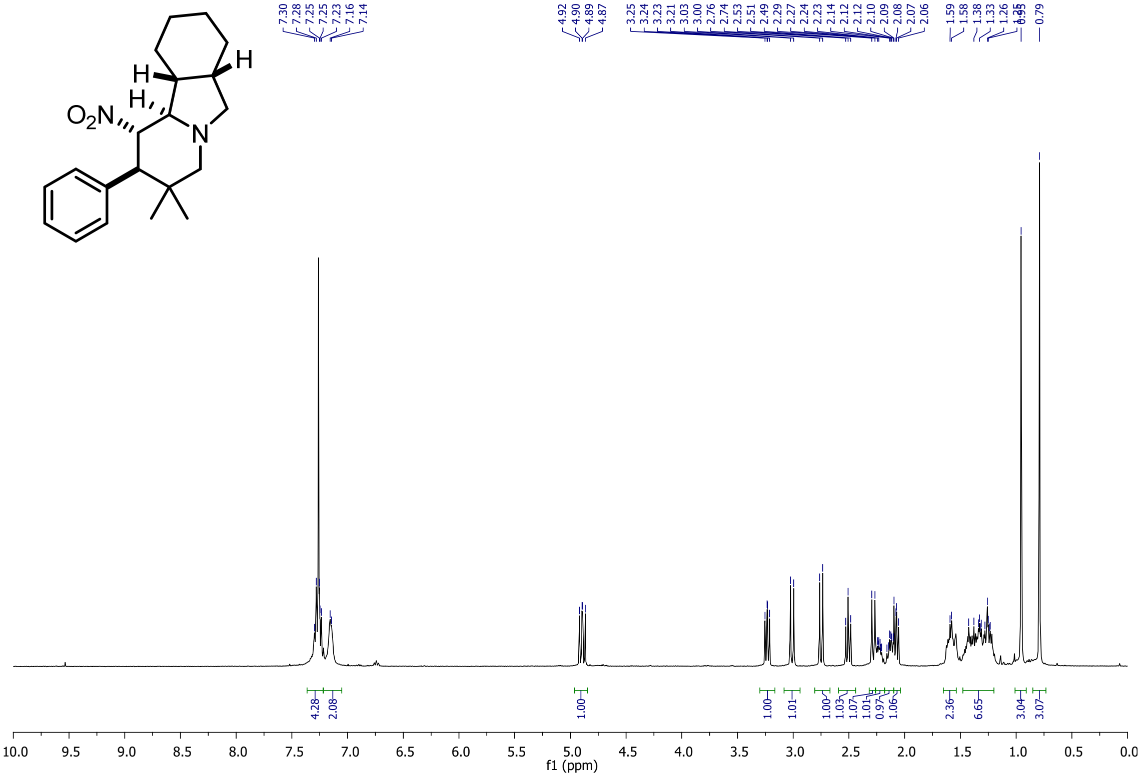

5d – 3,3-dimethyl-1-nitro-2-phenyldodecahydropyrido[2,1-a]isoindole – ^{13}C NMR (101 MHz, $CDCl_3$)

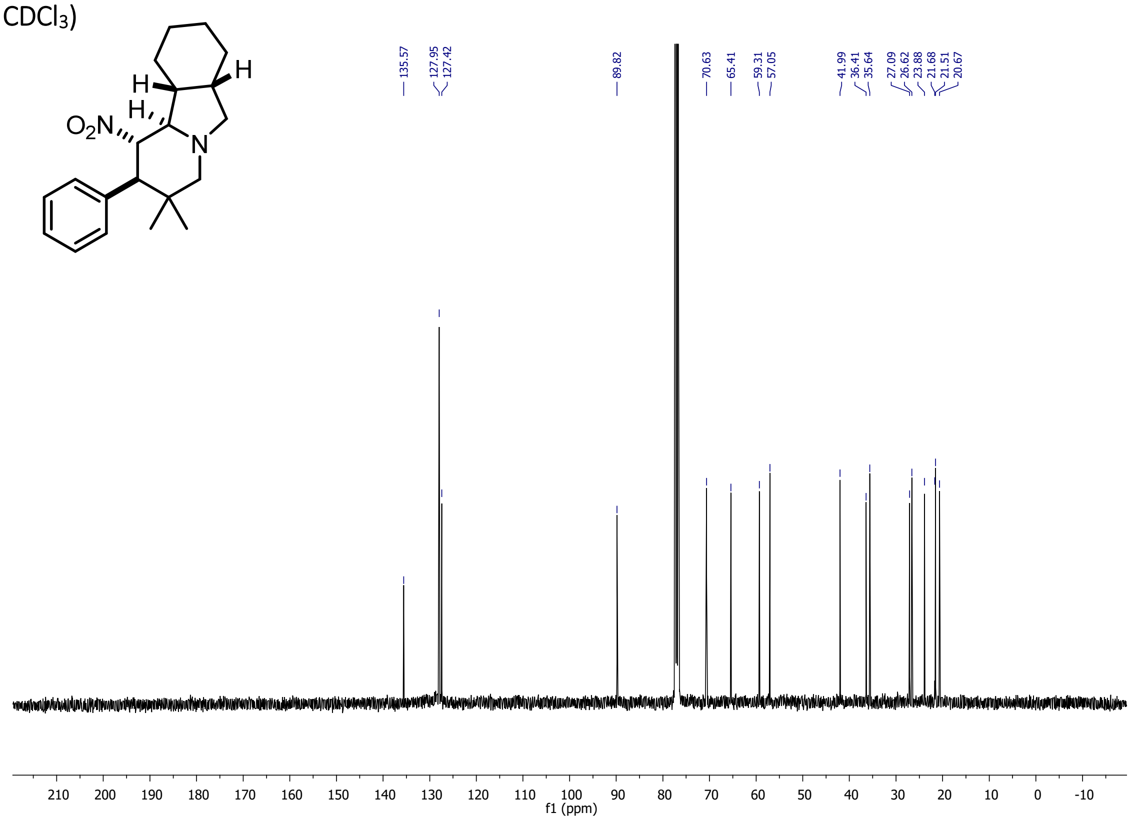

6.1.1.29 5e – 3,3-dimethyl-1-nitro-2-phenyl-1,3,4,6,11,11a-hexahydro-2H-pyrido[1,2-b]isoquinoline – ^{1}H NMR (600 MHz, $CDCl_3$)

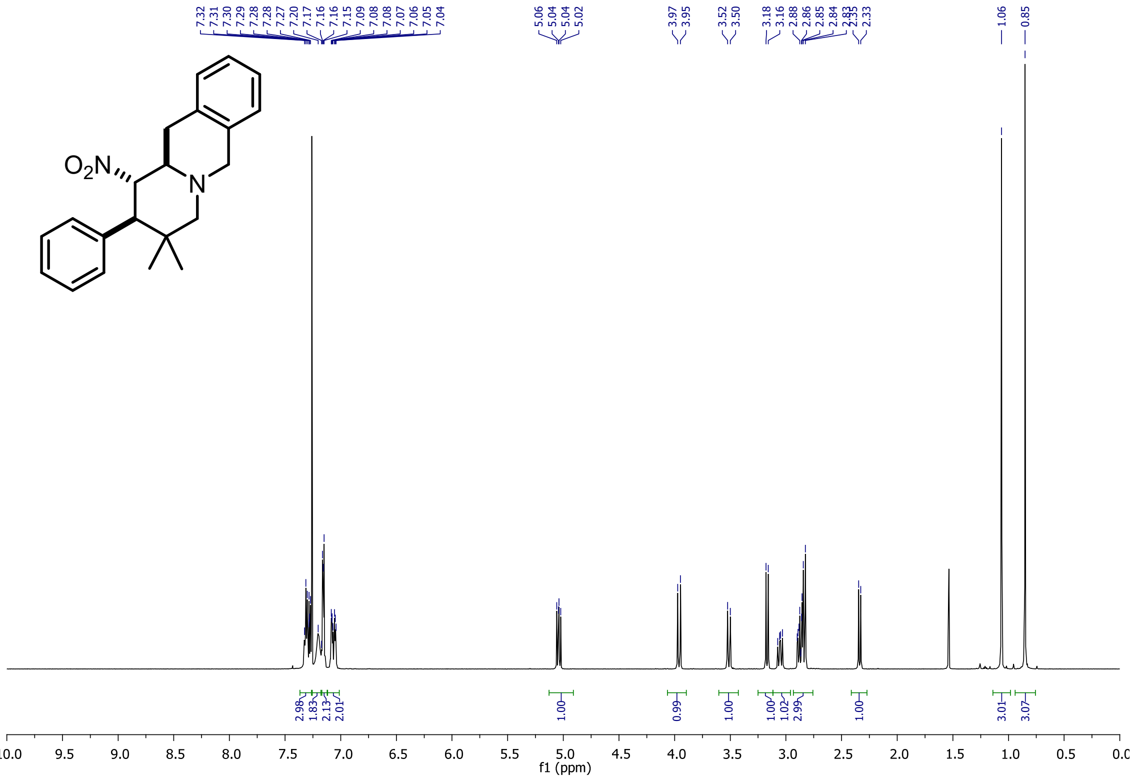

5e – 3,3-dimethyl-1-nitro-2-phenyl-1,3,4,6,11,11a-hexahydro-2H-pyrido[1,2-b]isoquinoline – ^{13}C NMR (151 MHz, $CDCl_3$)

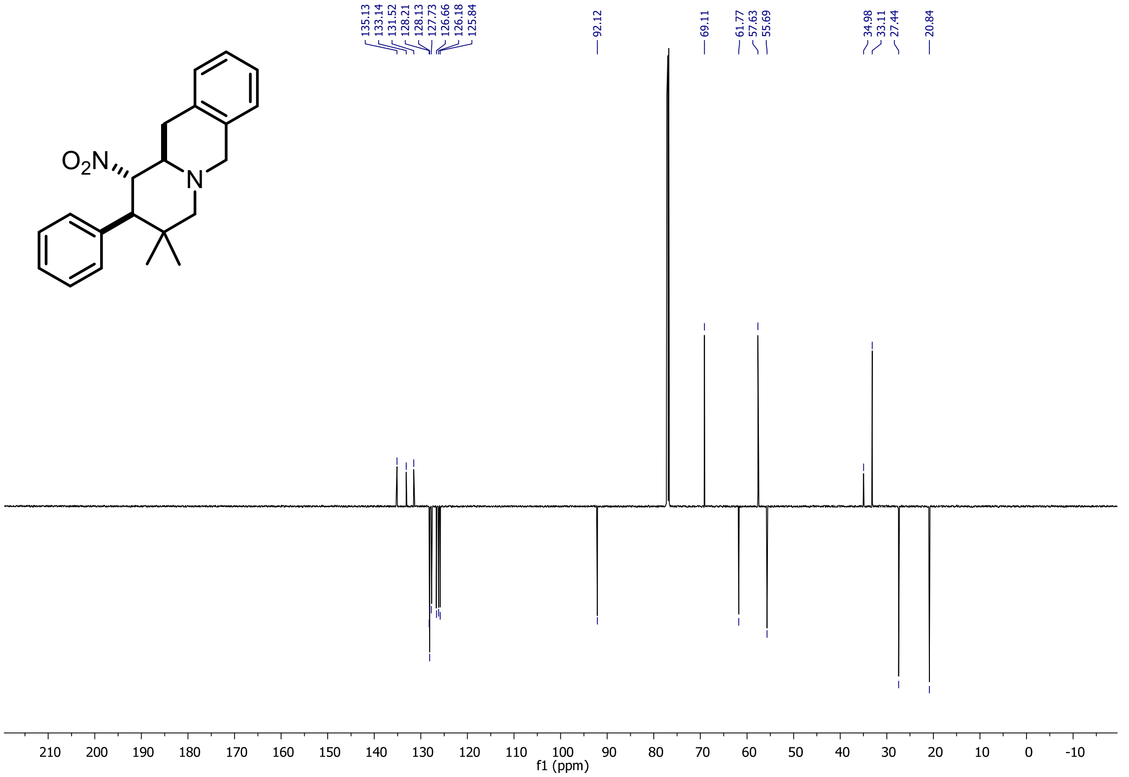

6.1.1.30 5e′ – 3,3-dimethyl-1-nitro-2-phenyl-1,3,4,6,7,11b-hexahydro-2H-pyrido[2,1-a]isoquinoline – ^{1}H NMR (600 MHz, $CDCl_3$)

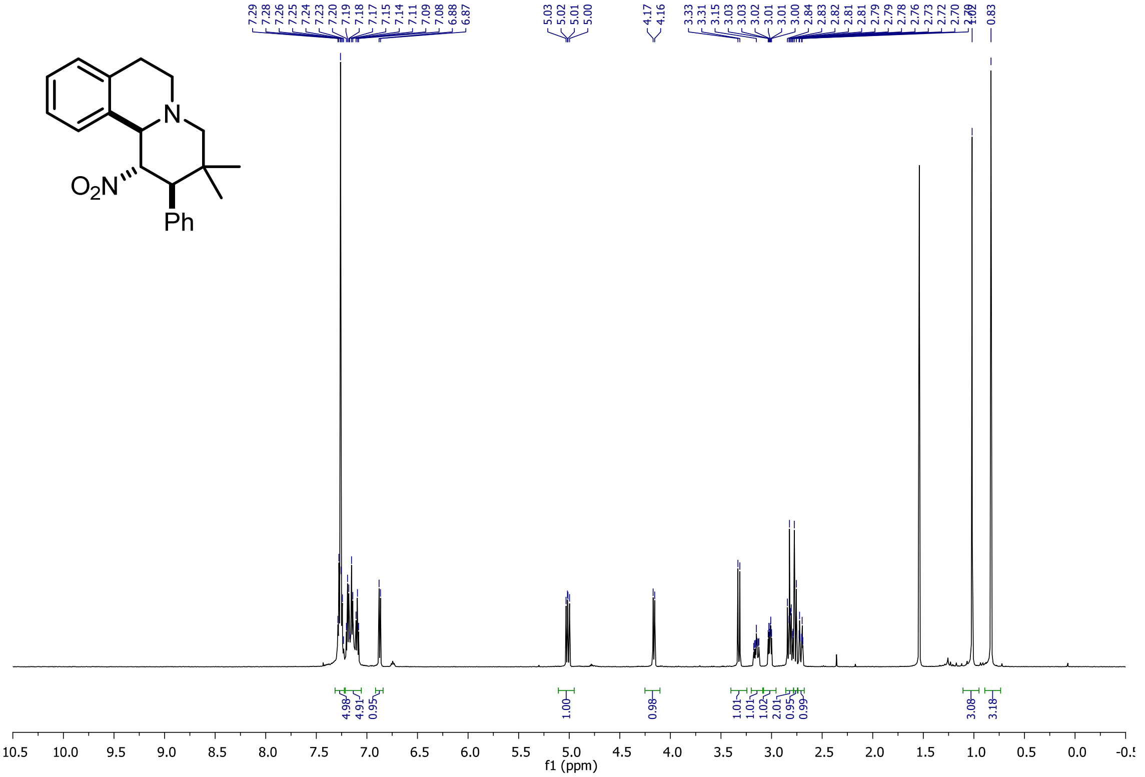

5e′ – 3,3-dimethyl-1-nitro-2-phenyl-1,3,4,6,7,11b-hexahydro-2H-pyrido[2,1-a]isoquinoline – ^{13}C NMR (151 MHz, $CDCl_3$)

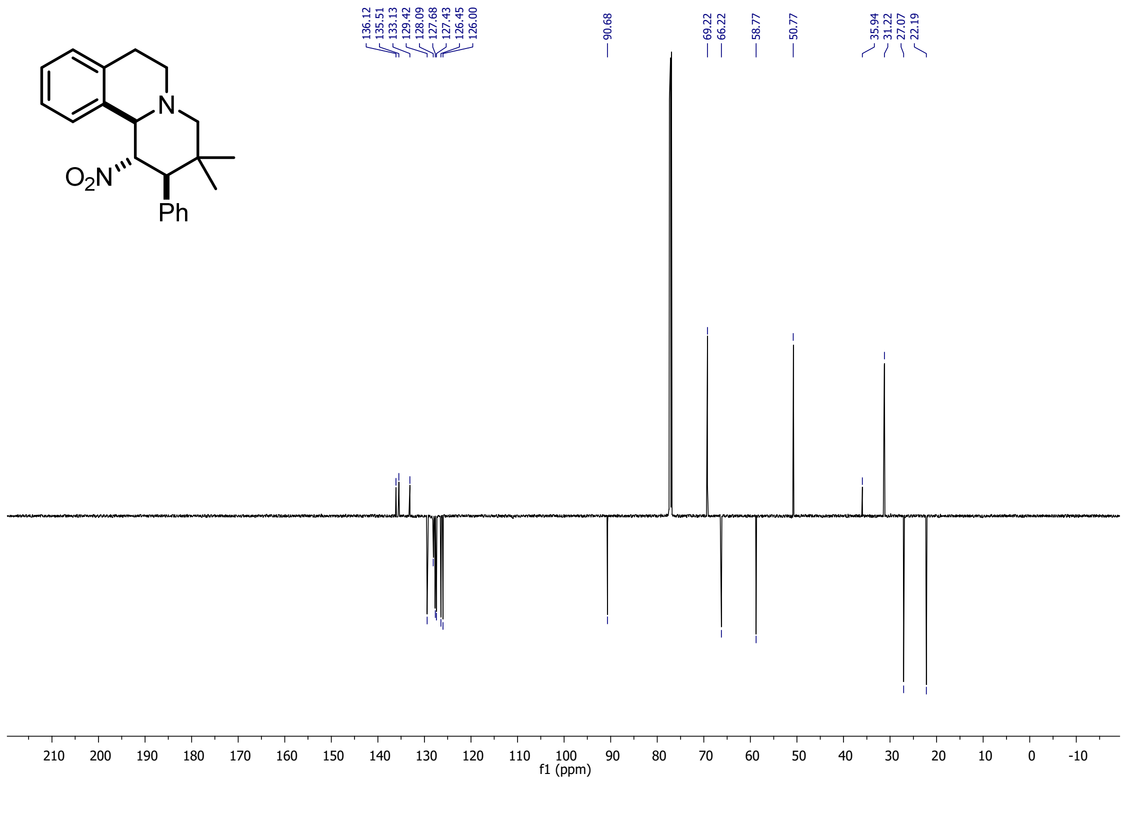

6.1.1.31 5f – 8-((tert-butyldimethylsilyl)oxy)-3,3-dimethyl-1-nitro-2-phenyloctahydro-2H-quinolizine– ^{1}H NMR (600 MHz, $CDCl_3$)

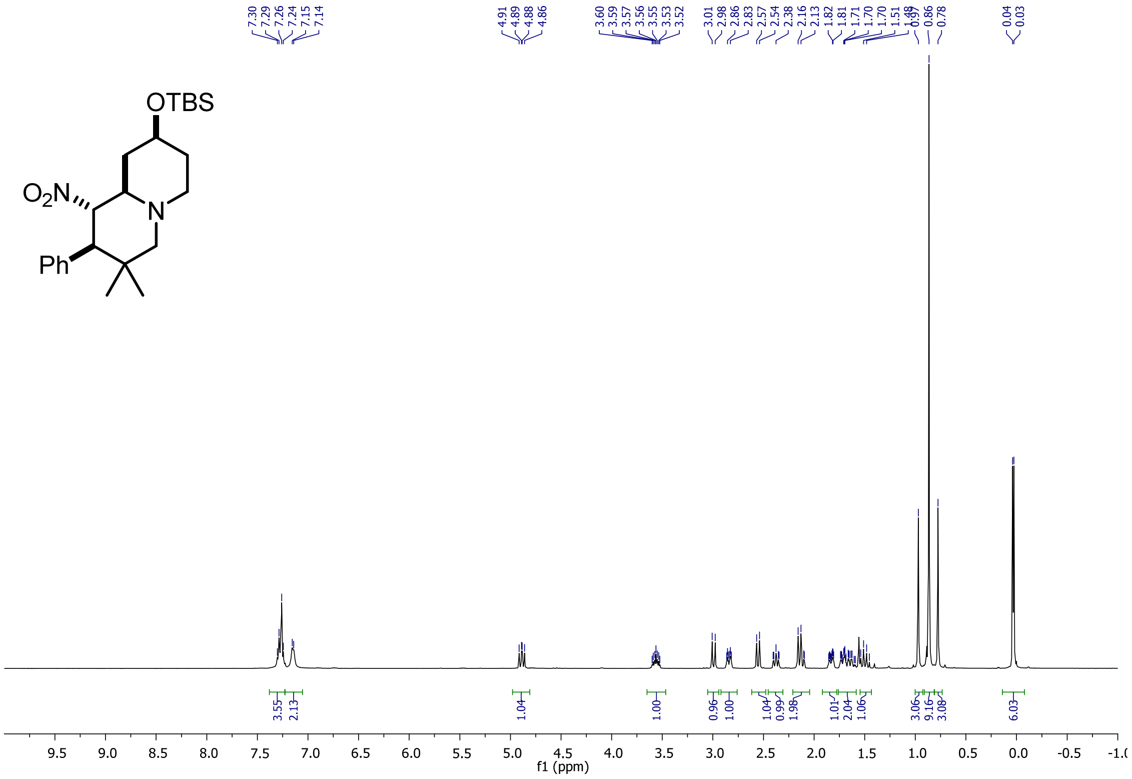

5f – 8-((tert-butyldimethylsilyl)oxy)-3,3-dimethyl-1-nitro-2-phenyloctahydro-2H-quinolizine– ^{13}C NMR (151 MHz, $CDCl_3$)

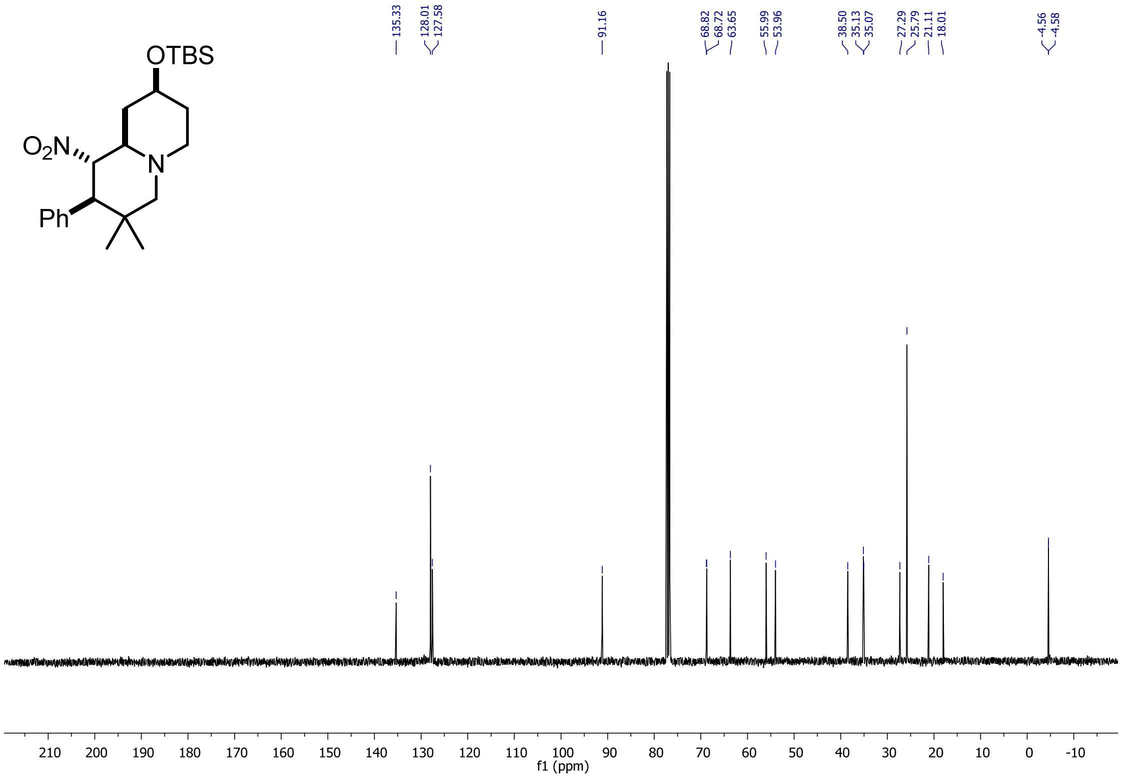

6.1.1.32 5g – 3,3,6-trimethyl-1-nitro-2-phenyloctahydro-2H-quinolizine– ^{1}H NMR (600 MHz, $CDCl_3$)

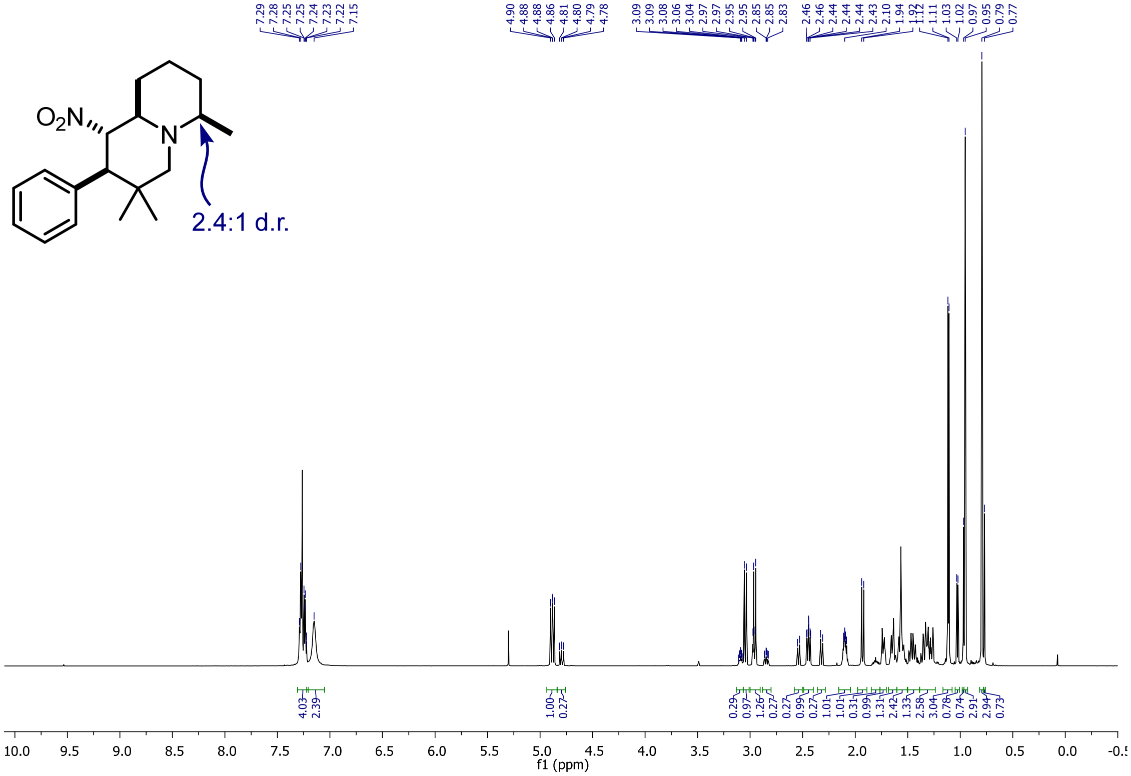

5g – 3,3,6-trimethyl-1-nitro-2-phenyloctahydro-2H-quinolizine – ^{13}C NMR (151 MHz, $CDCl_3$)

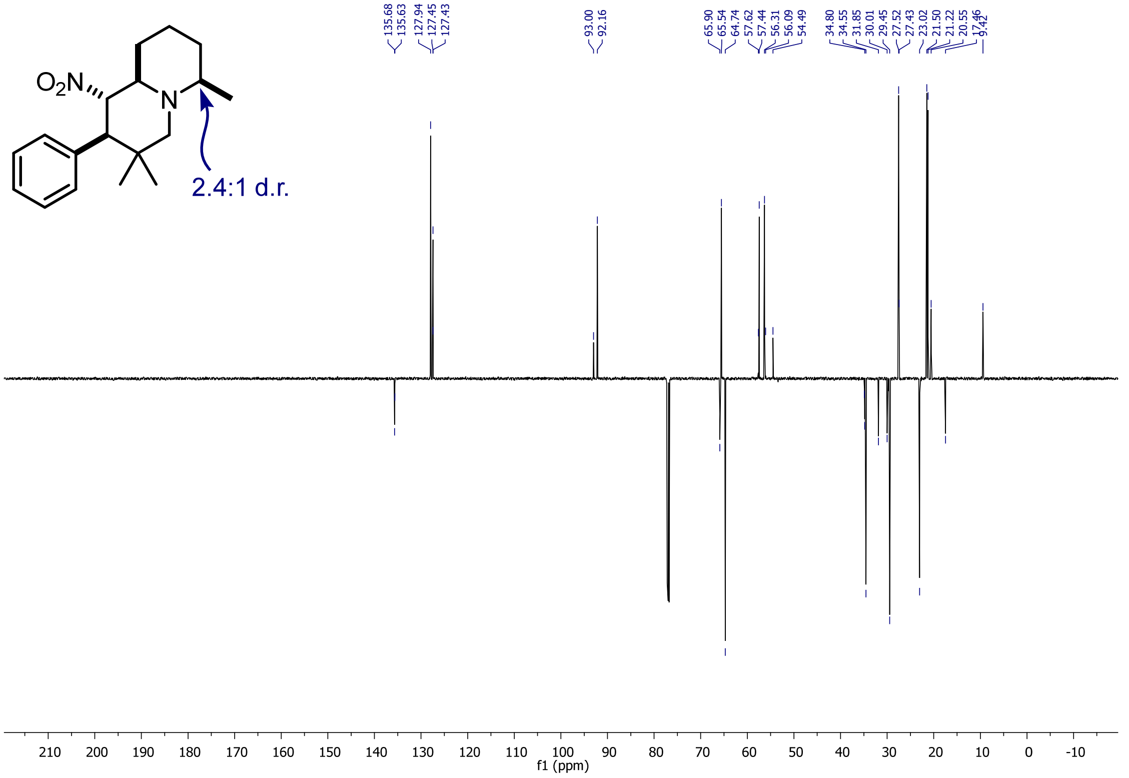

6.1.1.33 5h – 1-ethyl-2,5,5-trimethyl-3-nitro-4-phenylpiperidine – ^{1}H NMR (600 MHz, $CDCl_3$)

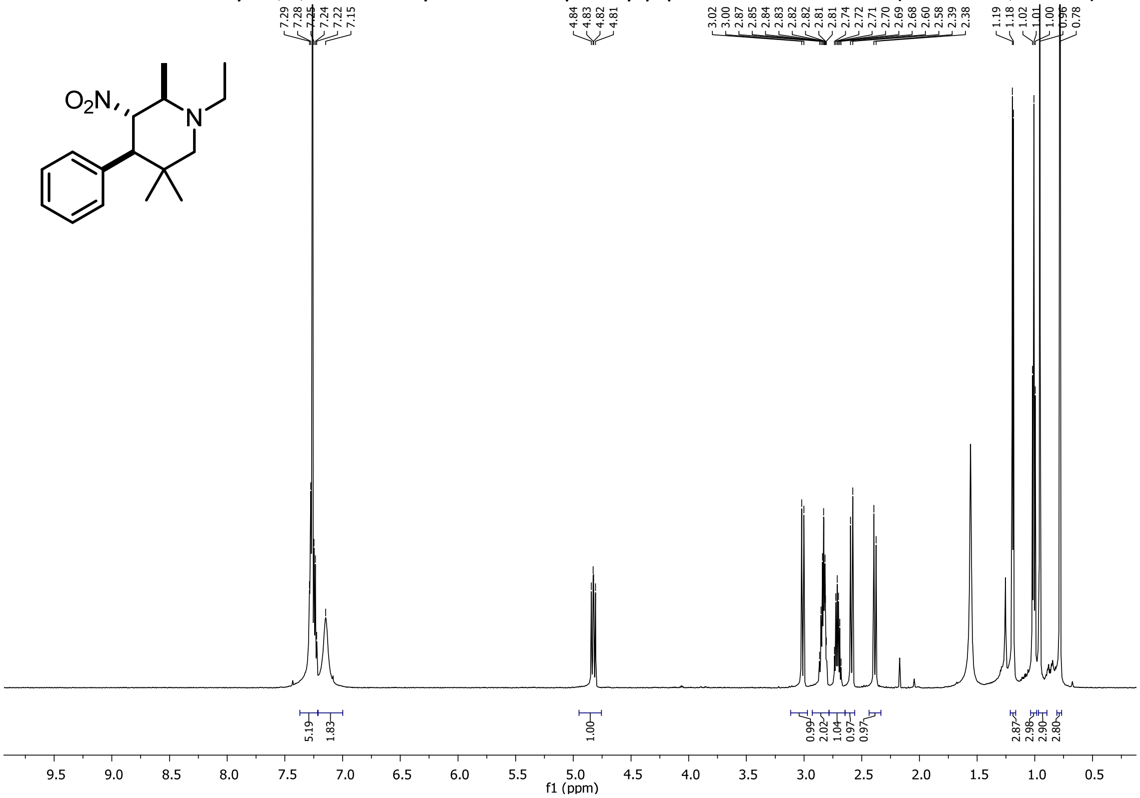

5h – 1-ethyl-2,5,5-trimethyl-3-nitro-4-phenylpiperidine – ^{13}C NMR (151 MHz, $CDCl_3$)

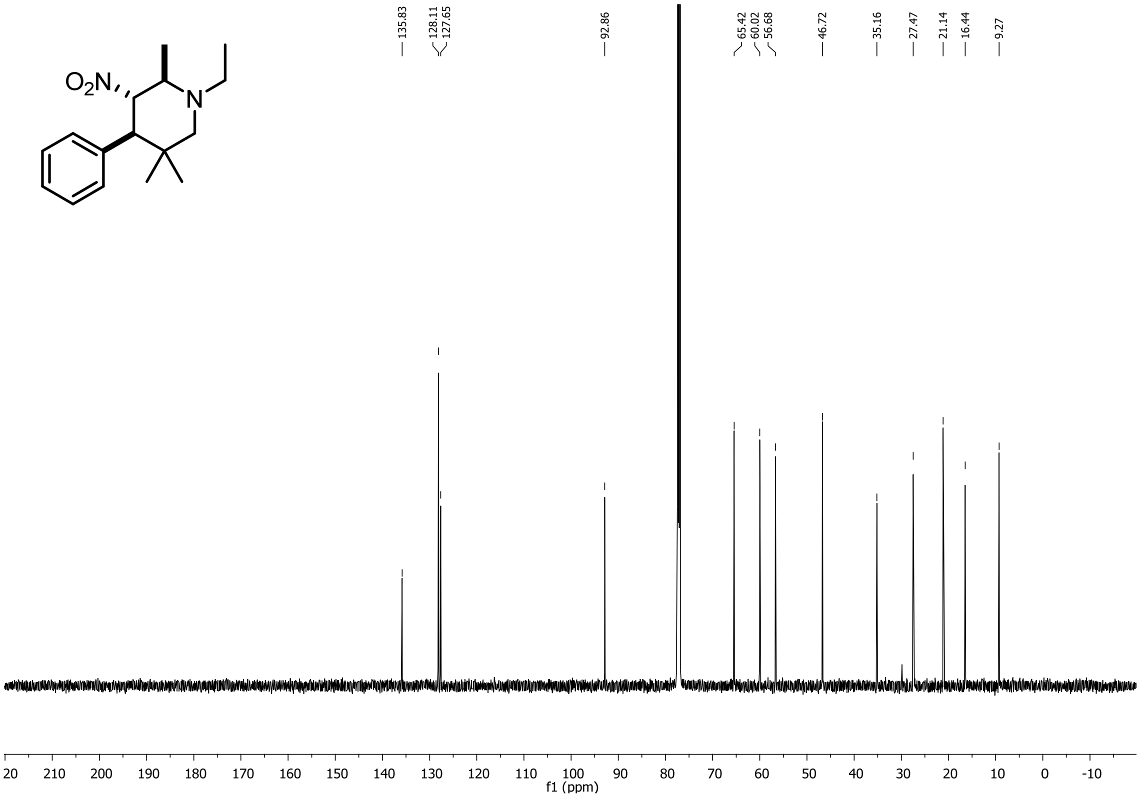

6.1.1.34 5i – 6-methyl-8-nitro-7-phenyl-6-propyloctahydroindolizine – 1H NMR (600 MHz, $CDCl_3$)

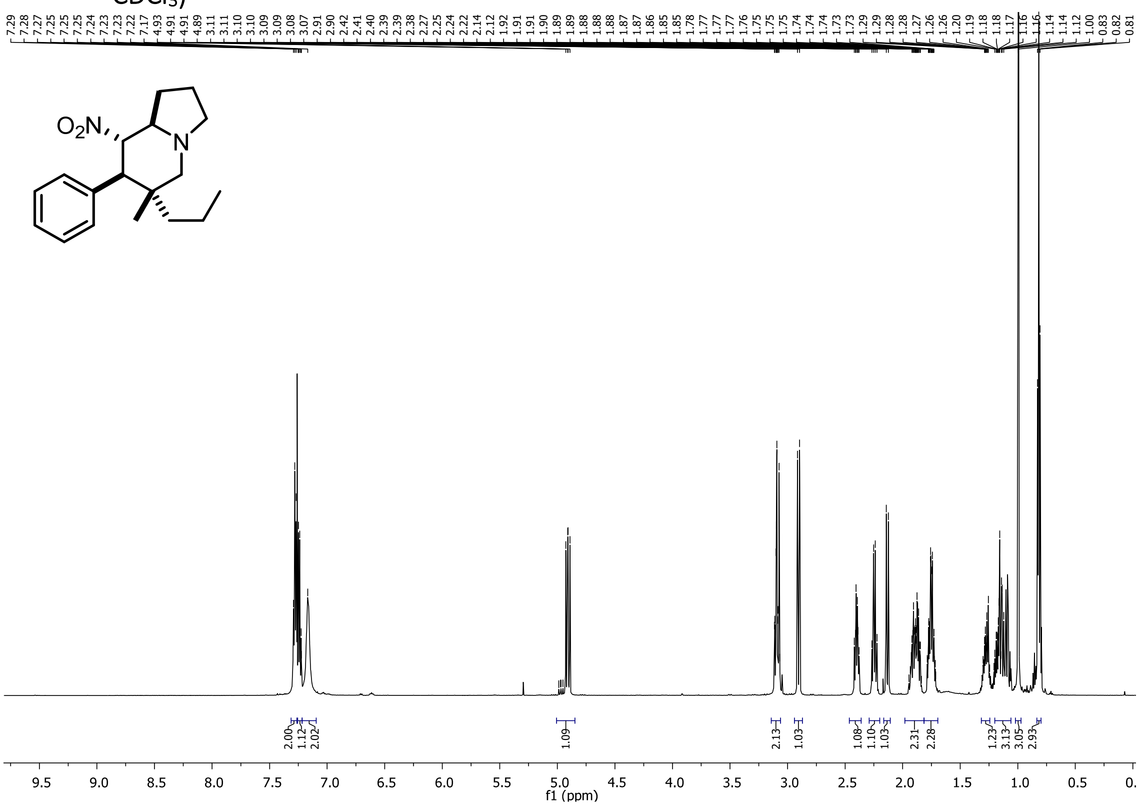

5i – 6-methyl-8-nitro-7-phenyl-6-propyloctahydroindolizine – ^{13}C NMR (151 MHz, $CDCl_3$)

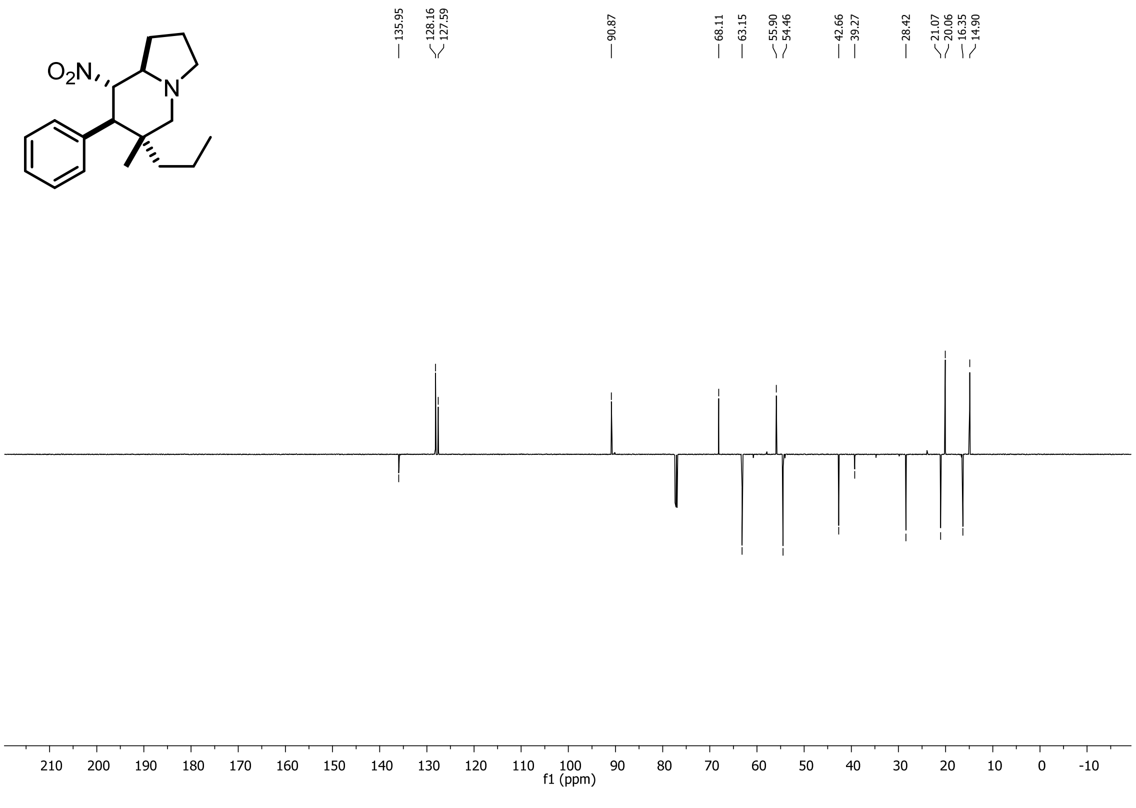

6.1.1.35 5j – 6-methyl-6-(4-methylpent-3-en-1-yl)-8-nitro-7-phenyloctahydroindolizine – ^{1}H NMR (600 MHz, $CDCl_3$)

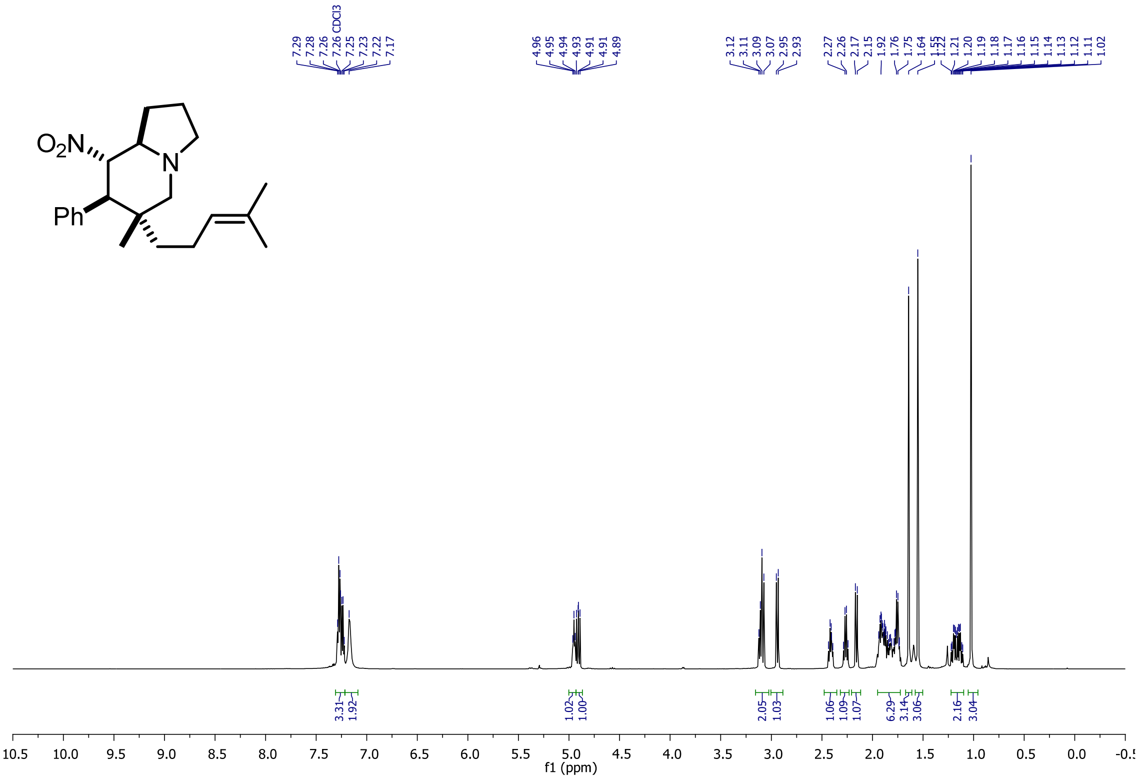

5j – 6-methyl-6-(4-methylpent-3-en-1-yl)-8-nitro-7-phenyloctahydroindolizine – ^{13}C NMR (151 MHz, $CDCl_3$)

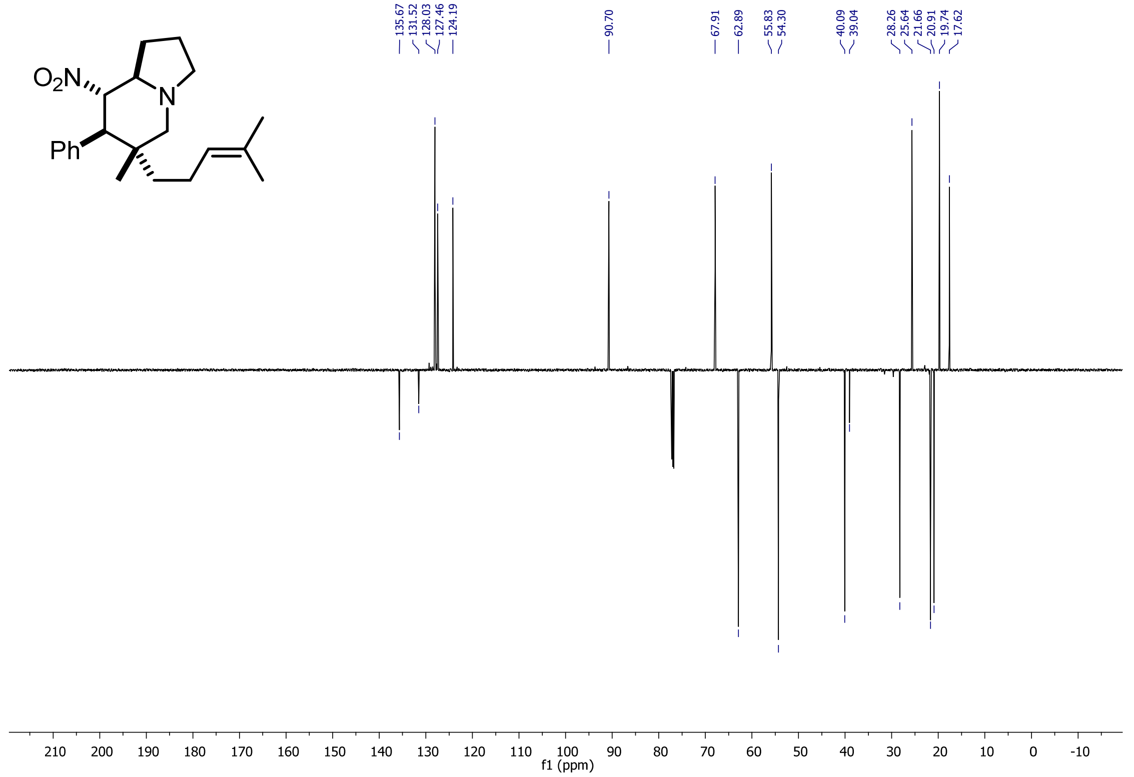

6.1.1.36 5k – 6-isopropyl-8-nitro-7-phenyloctahydroindolizine – ^{1}H NMR (400 MHz, $CDCl_3$)

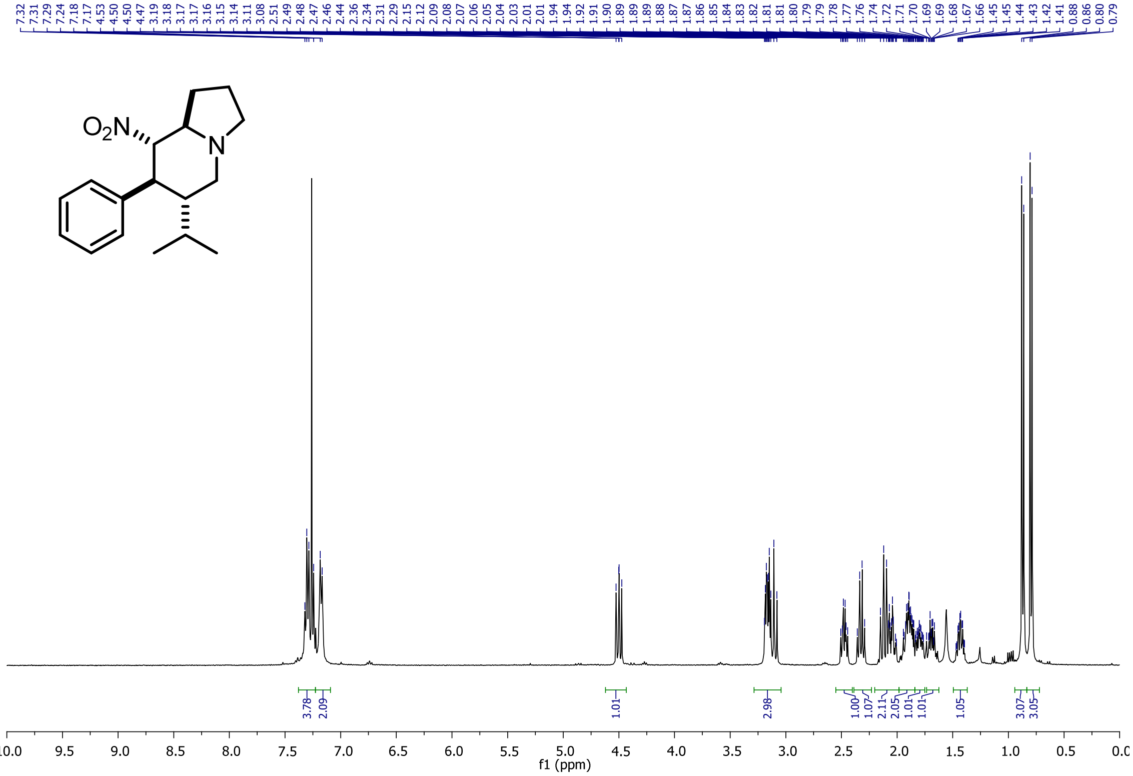

5k – 6-isopropyl-8-nitro-7-phenyloctahydroindolizine – ^{13}C NMR (101 MHz, $CDCl_3$)

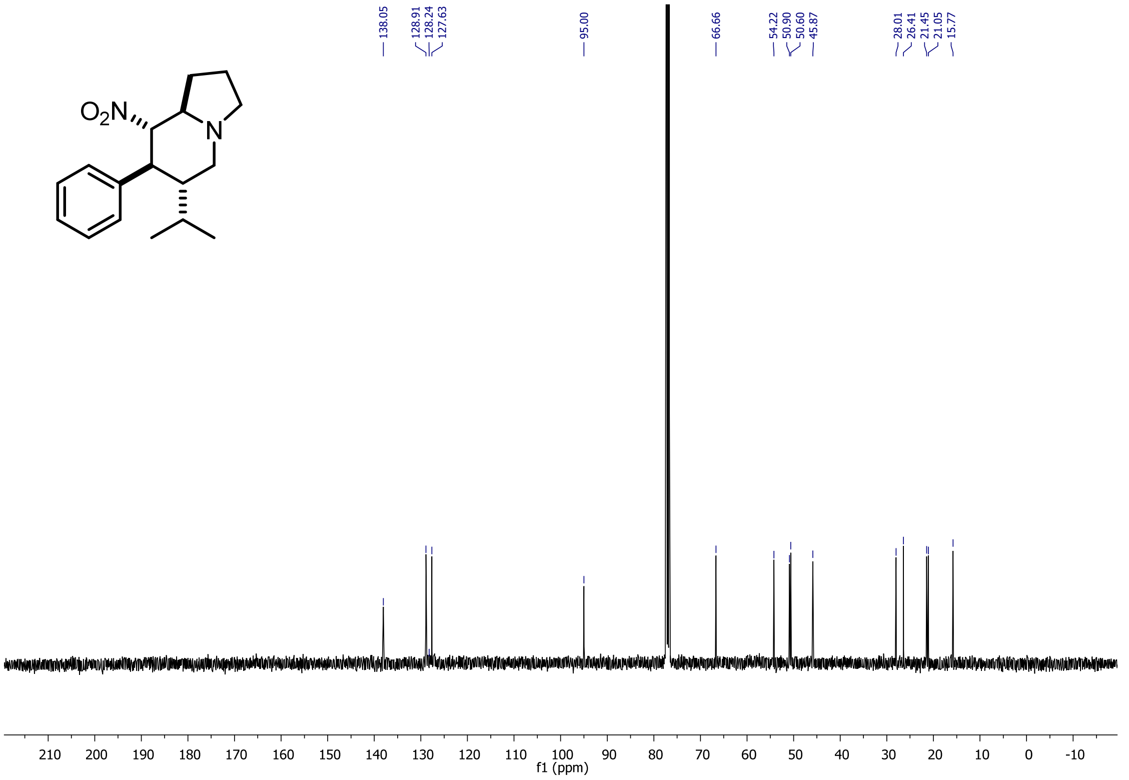

6.1.1.37 5l – 8'-nitro-7'-phenylhexahydro-5'H-spiro[cyclohexane-1,6'-indolizine] – ^{1}H NMR (400 MHz, $CDCl_3$)

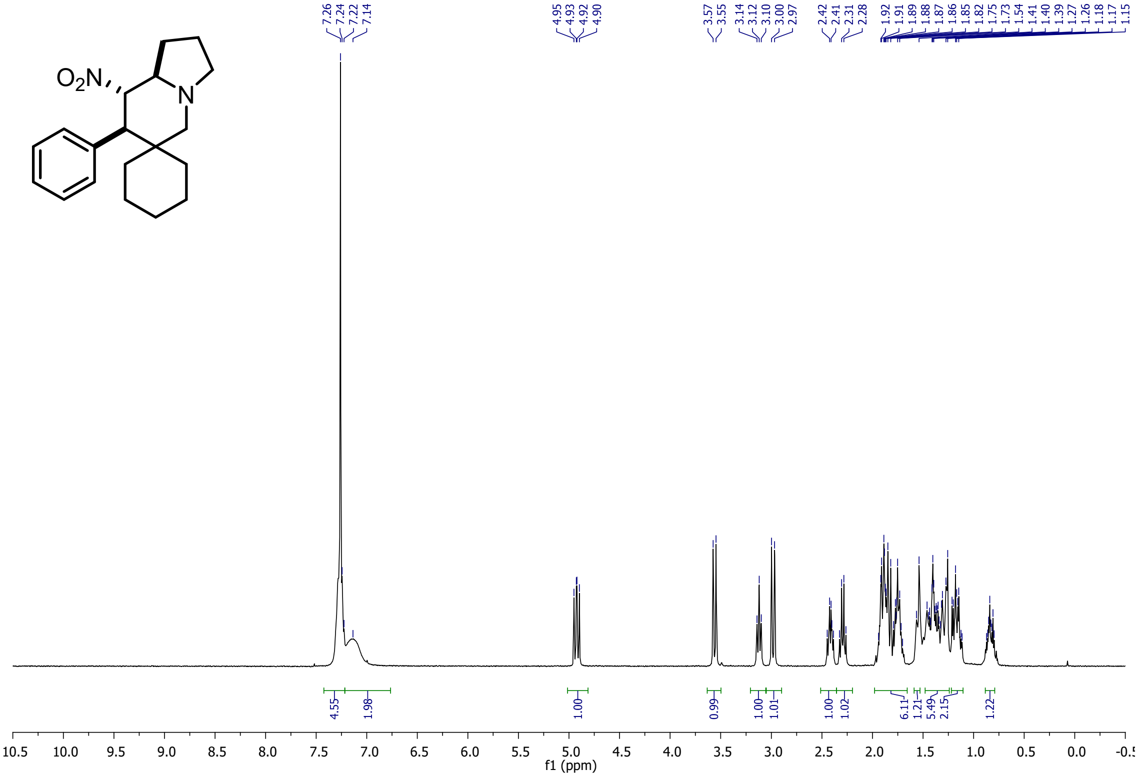

5l – 8'-nitro-7'-phenylhexahydro-5'H-spiro[cyclohexane-1,6'-indolizine] – ^{13}C NMR (101 MHz, $CDCl_3$)

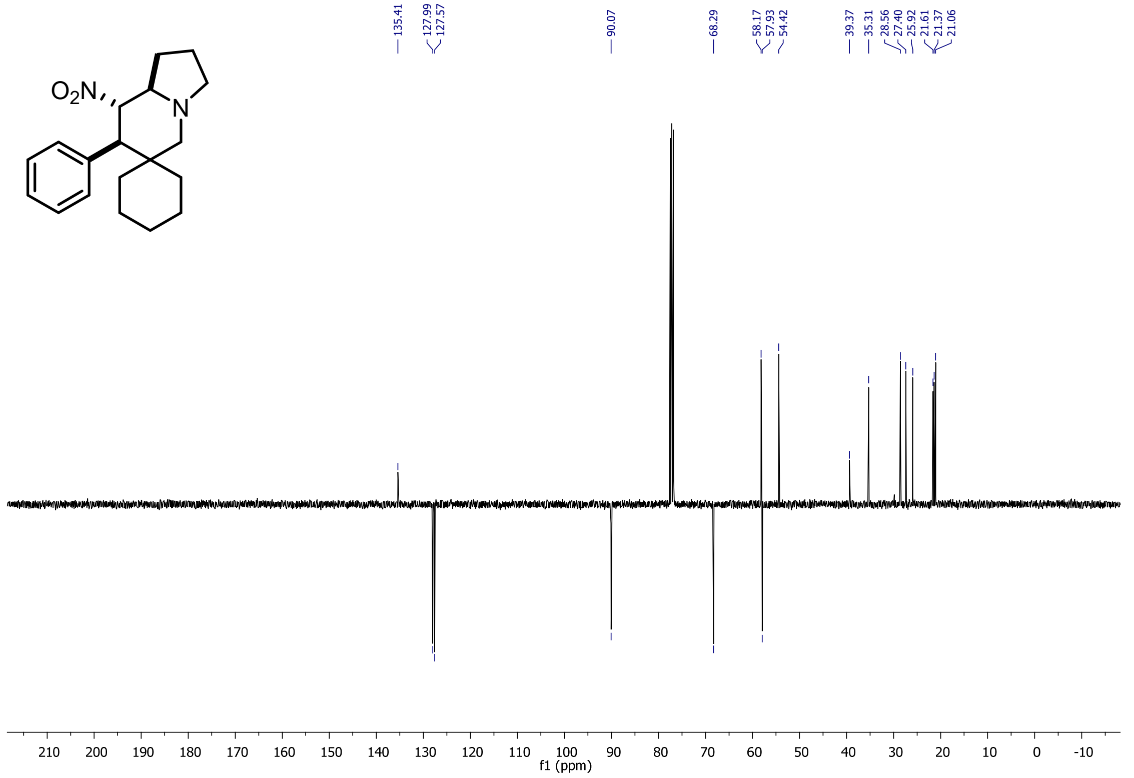

6.1.1.38 5m – 6,6-dimethyl-8-nitro-7-(*p*-tolyl)octahydroindolizine – ^{1}H NMR (600 MHz, $CDCl_3$)

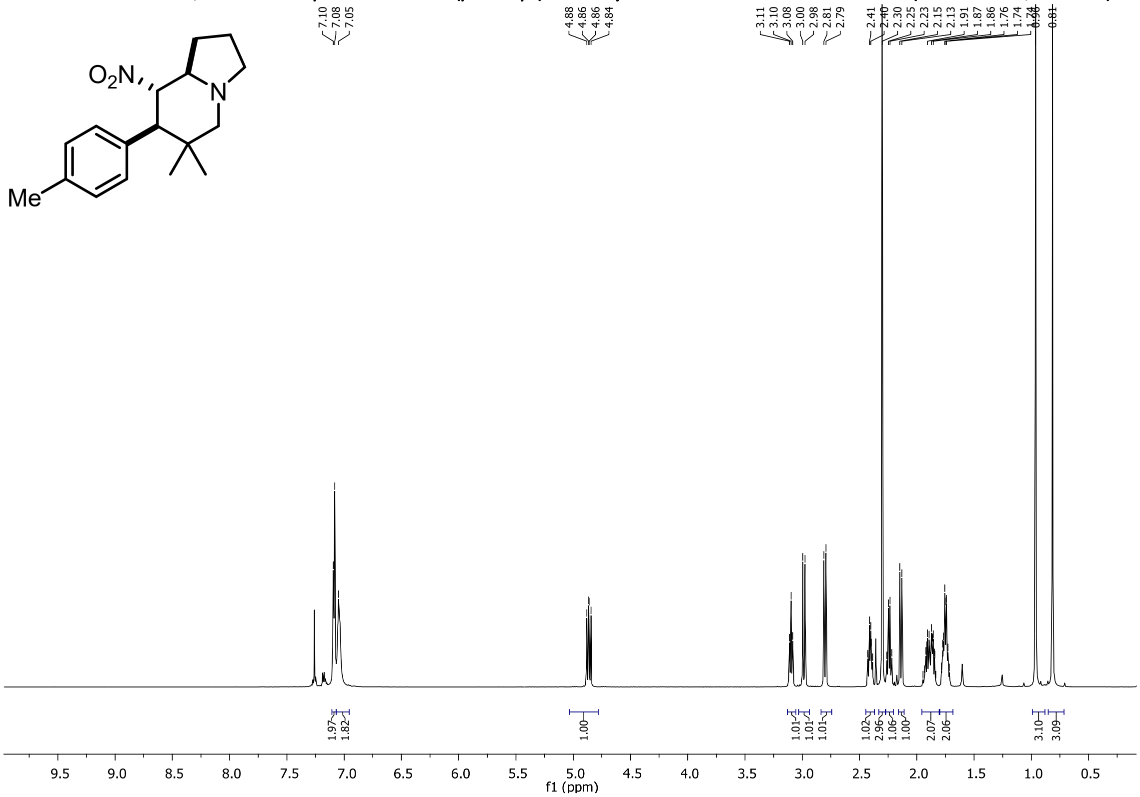

5m – 6,6-dimethyl-8-nitro-7-(*p*-tolyl)octahydroindolizine – ^{13}C NMR (151 MHz, $CDCl_3$)

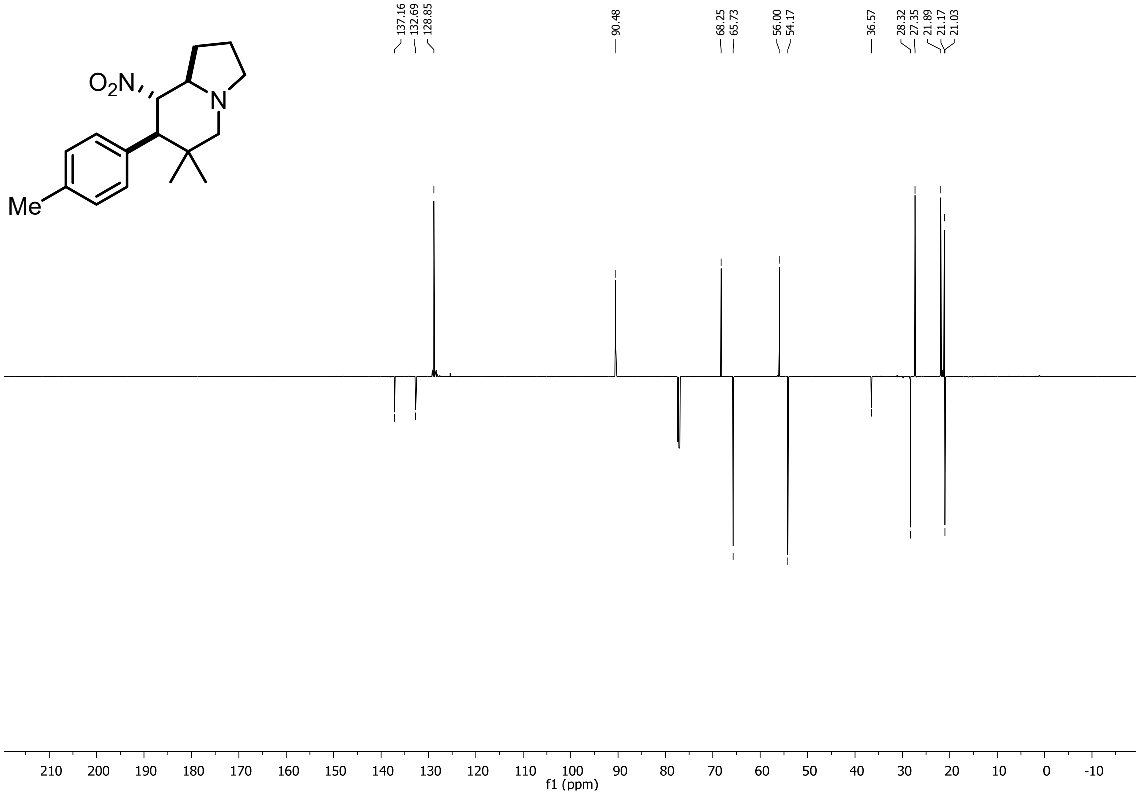

6.1.1.39 5n – 7-(4-methoxyphenyl)-6,6-dimethyl-8-nitrooctahydroindolizine – ^{1}H NMR (600 MHz, $CDCl_3$)

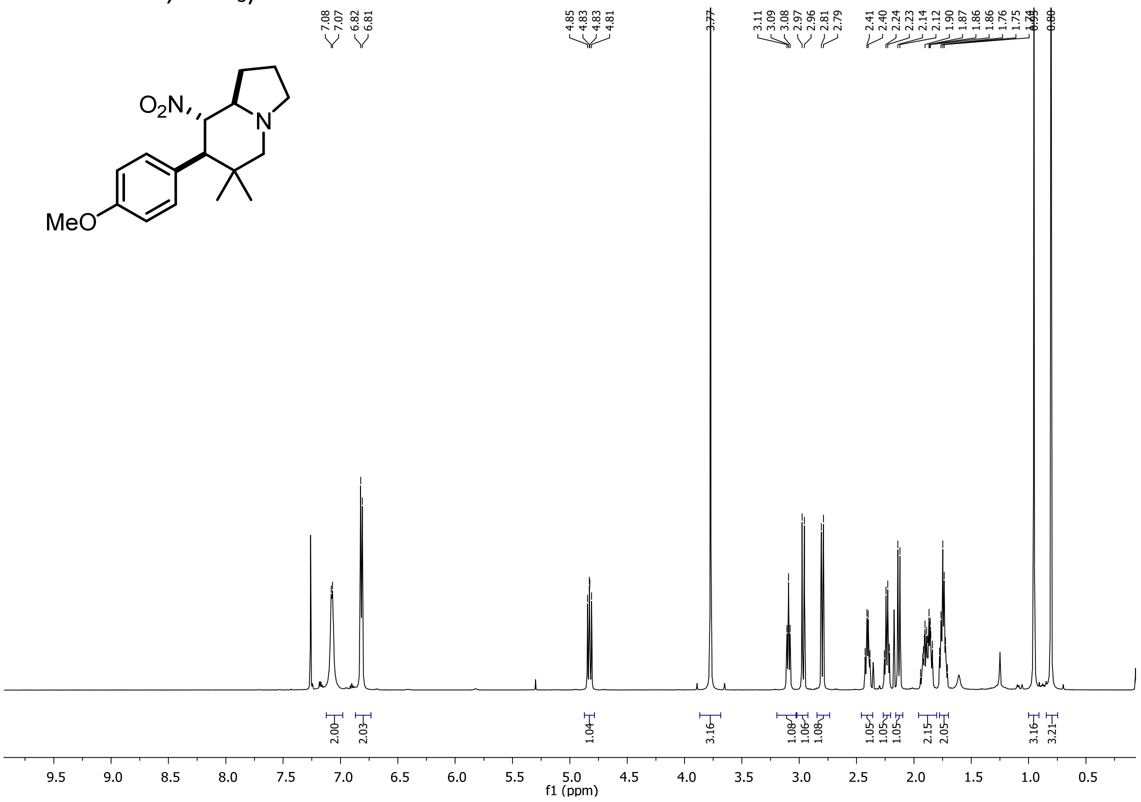

5n – 7-(4-methoxyphenyl)-6,6-dimethyl-8-nitrooctahydroindolizine – ^{13}C NMR (151 MHz, $CDCl_3$)

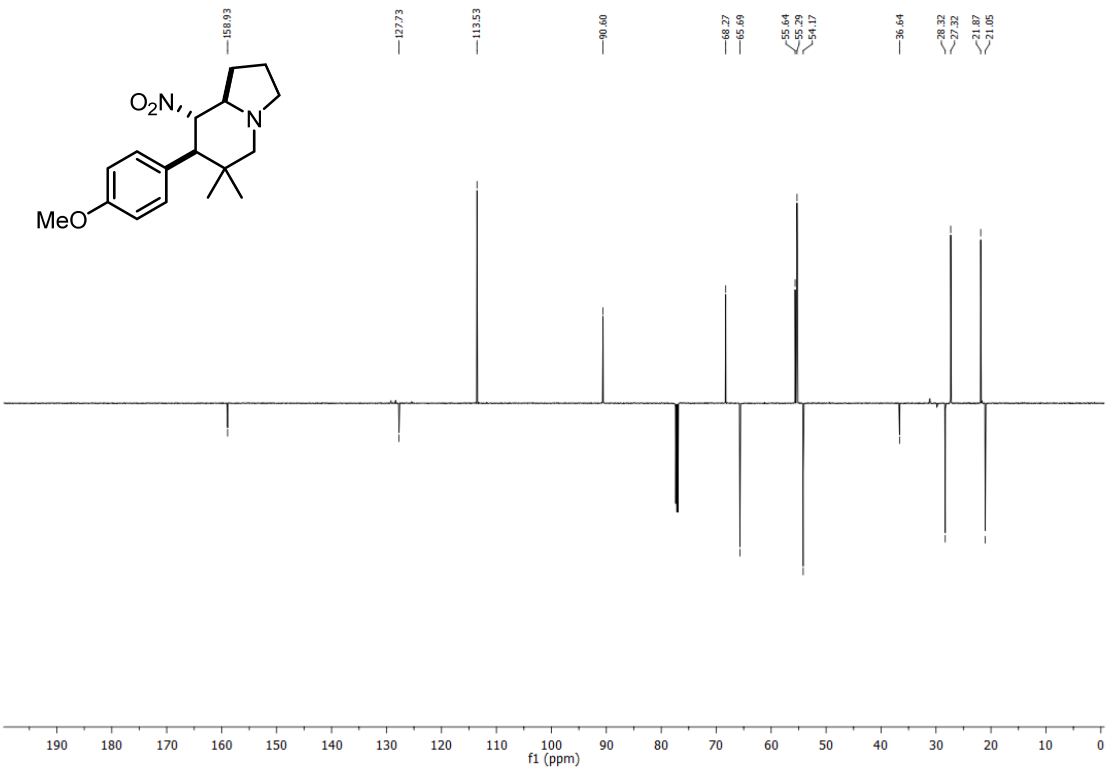

6.1.1.40 5o – 7-(2-(benzyloxy)phenyl)-6,6-dimethyl-8-nitrooctahydroindolizine – ^{1}H NMR (600 MHz, $CDCl_3$)

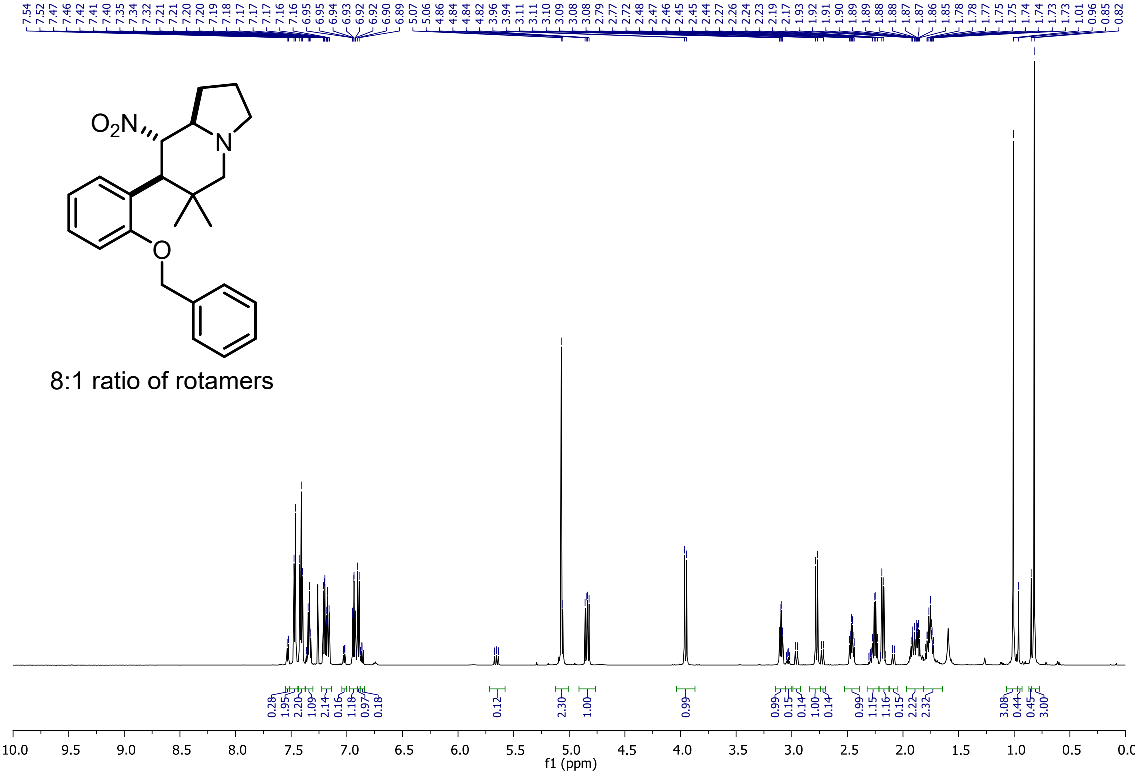

5o – 7-(2-(benzyloxy)phenyl)-6,6-dimethyl-8-nitrooctahydroindolizine – ^{13}C NMR (151 MHz, $CDCl_3$)

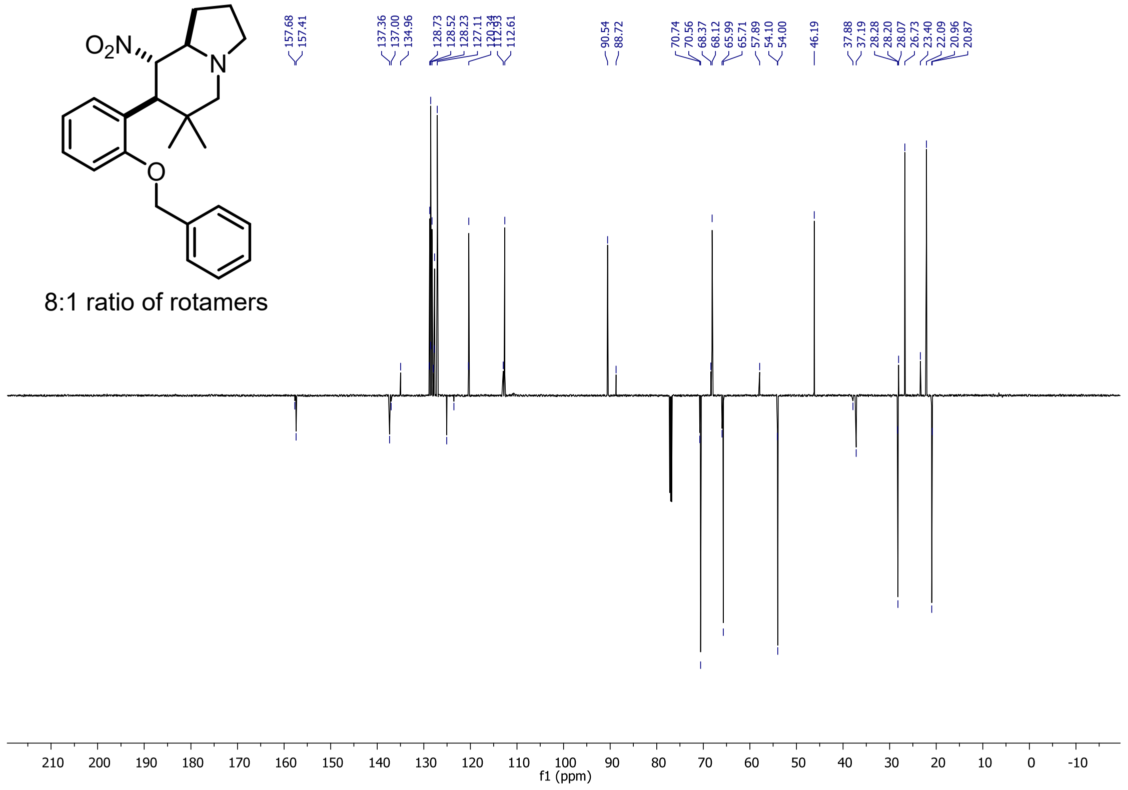

5o – 7-(2-(benzyloxy)phenyl)-6,6-dimethyl-8-nitrooctahydroindolizine – 2D-NOESY Spectrum

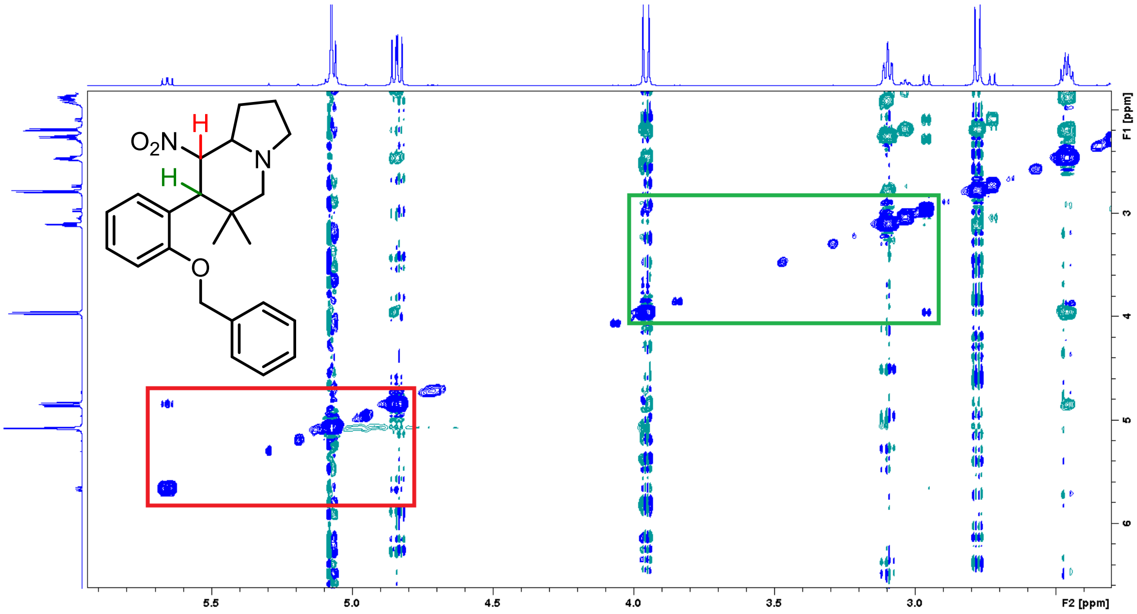

6.1.1.41 5p – 7-(2,4-dimethoxyphenyl)-6,6-dimethyl-8-nitrooctahydroindolizine – ^{1}H NMR (700 MHz, $CDCl_3$)

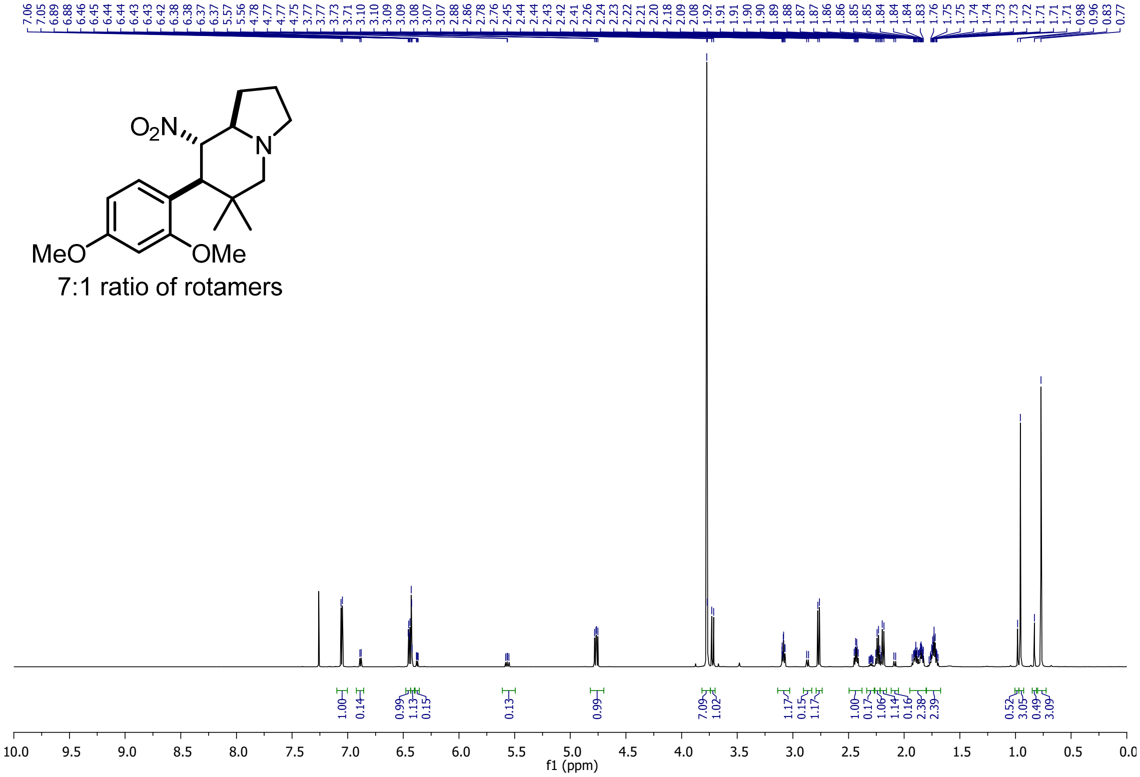

5p – 7-(2,4-dimethoxyphenyl)-6,6-dimethyl-8-nitrooctahydroindolizine – ^{13}C NMR (176 MHz, $CDCl_3$)

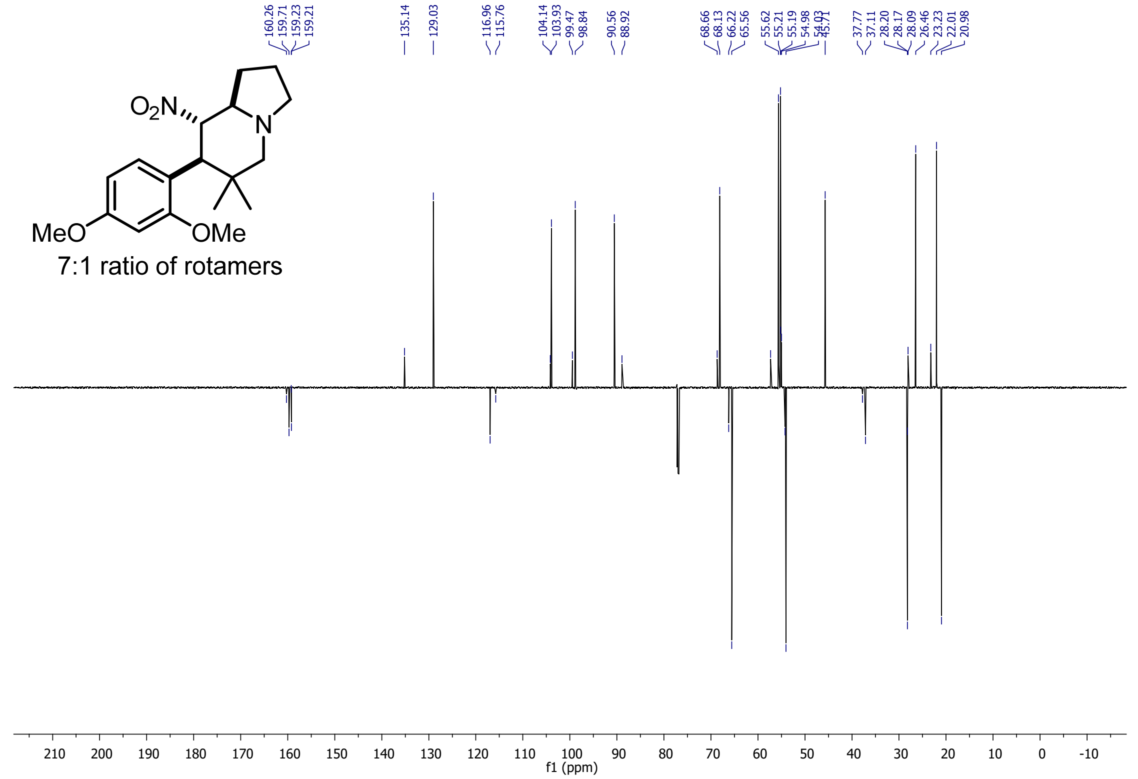

5p – 7-(2,4-dimethoxyphenyl)-6,6-dimethyl-8-nitrooctahydroindolizine – 2D-NOESY Spectrum

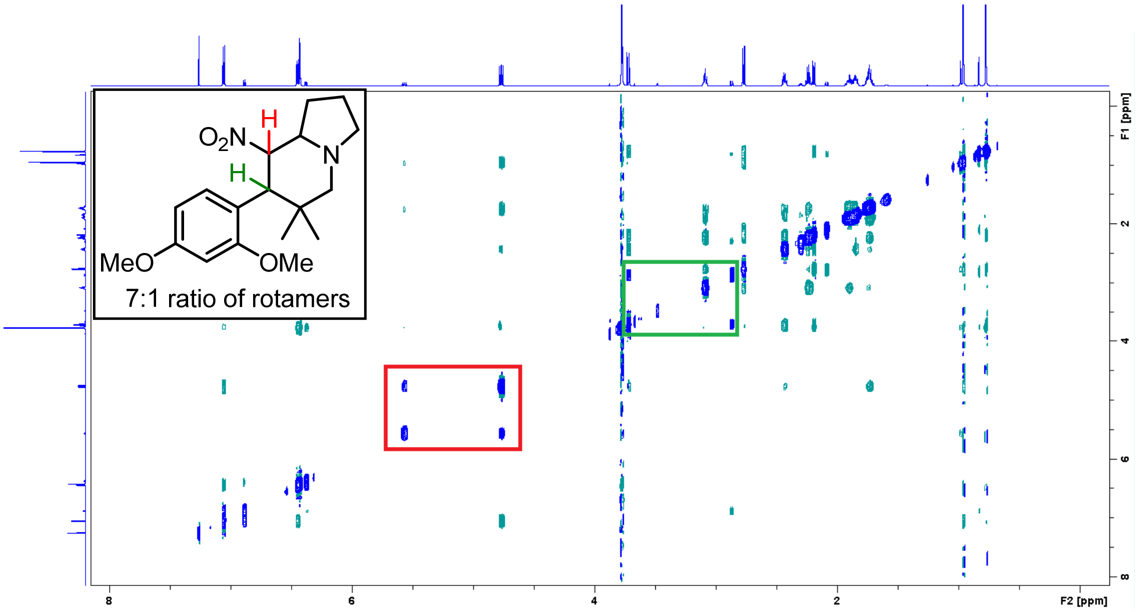

6.1.1.42 5q – 6,6-dimethyl-8-nitro-7-(4-nitrophenyl)octahydroindolizine – ^{1}H NMR (600 MHz, $CDCl_3$)

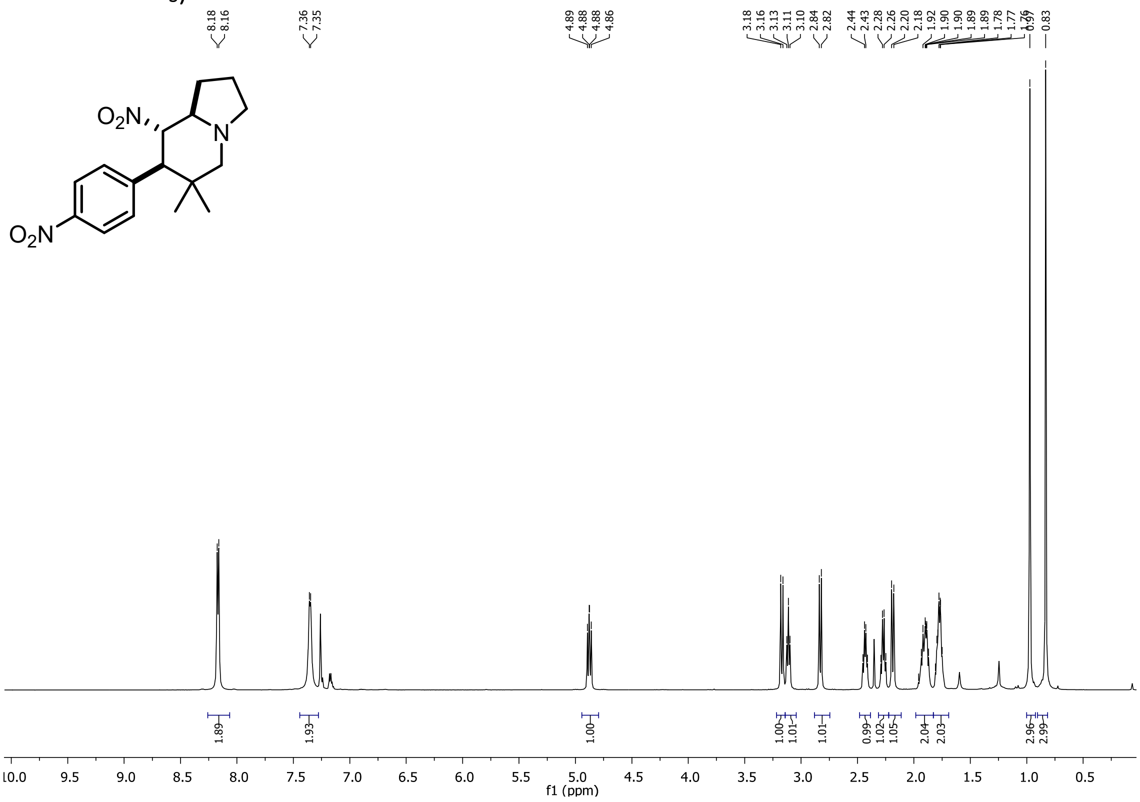

5q – 6,6-dimethyl-8-nitro-7-(4-nitrophenyl)octahydroindolizine – ^{13}C NMR (151 MHz, $CDCl_3$)

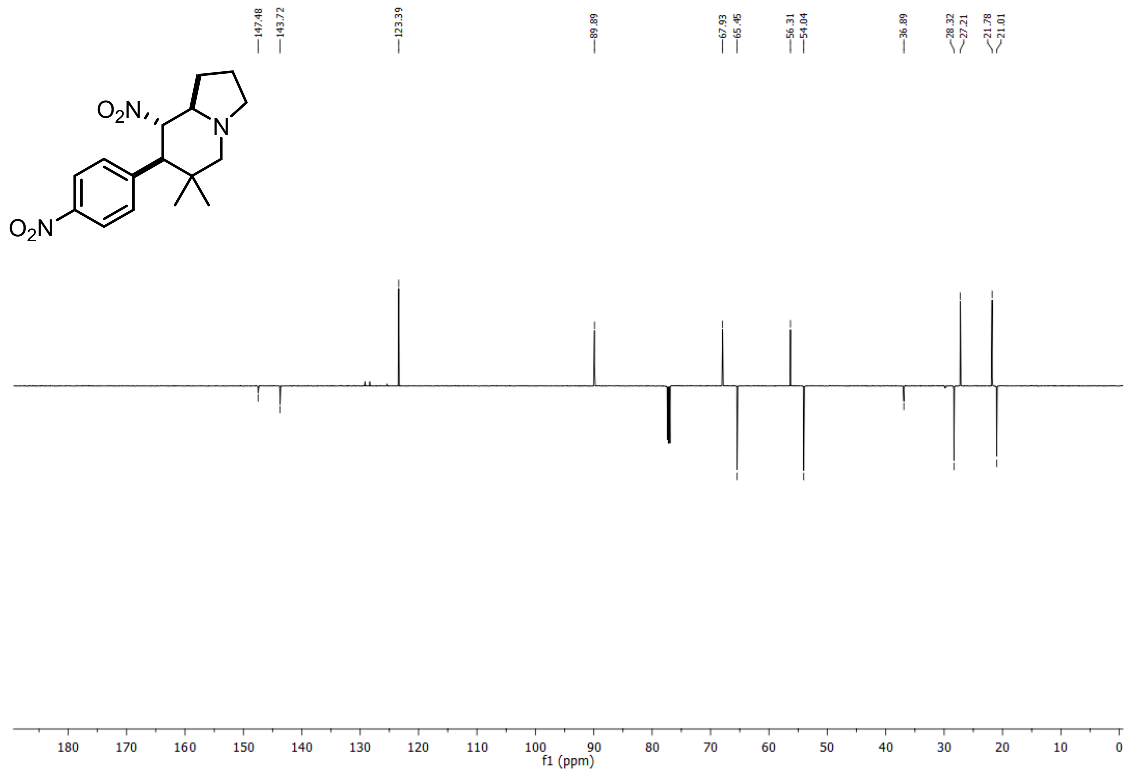

6.1.1.43 5r – 7-(4-fluorophenyl)-6,6-dimethyl-8-nitrooctahydroindolizine – ^{1}H NMR (600 MHz, $CDCl_3$)

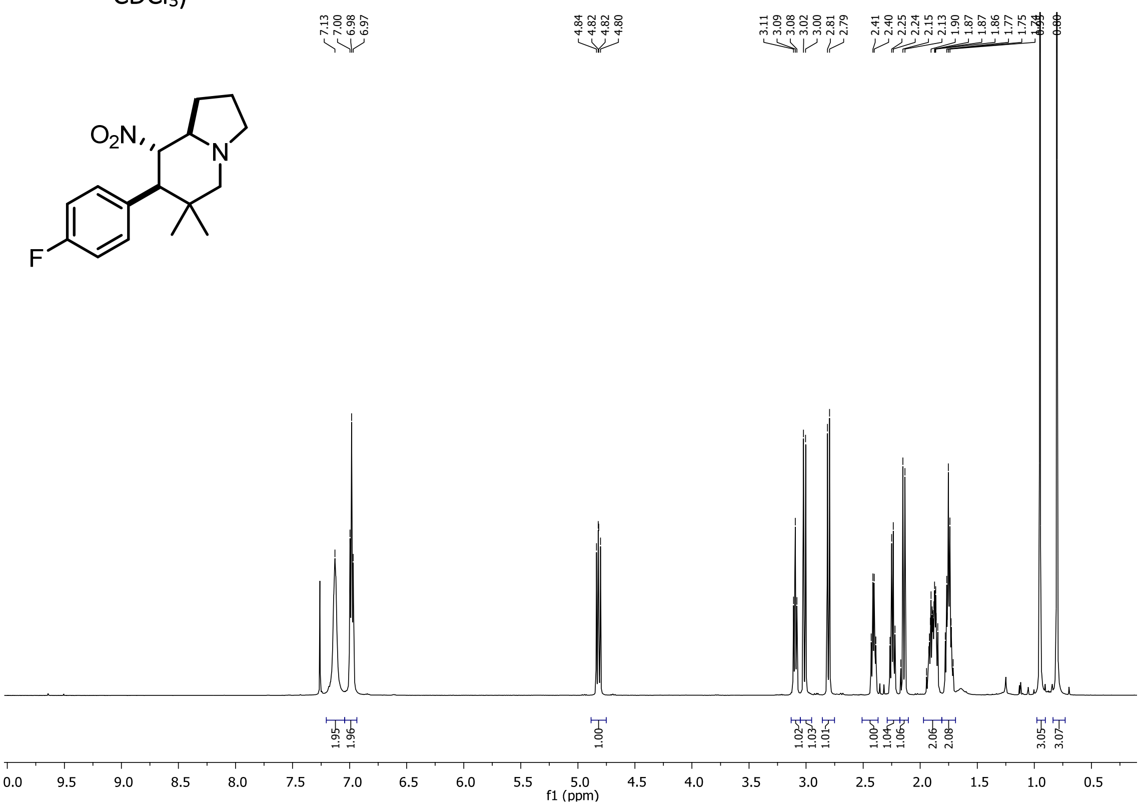

5r – 7-(4-fluorophenyl)-6,6-dimethyl-8-nitrooctahydroindolizine – ^{13}C NMR (151 MHz, $CDCl_3$)

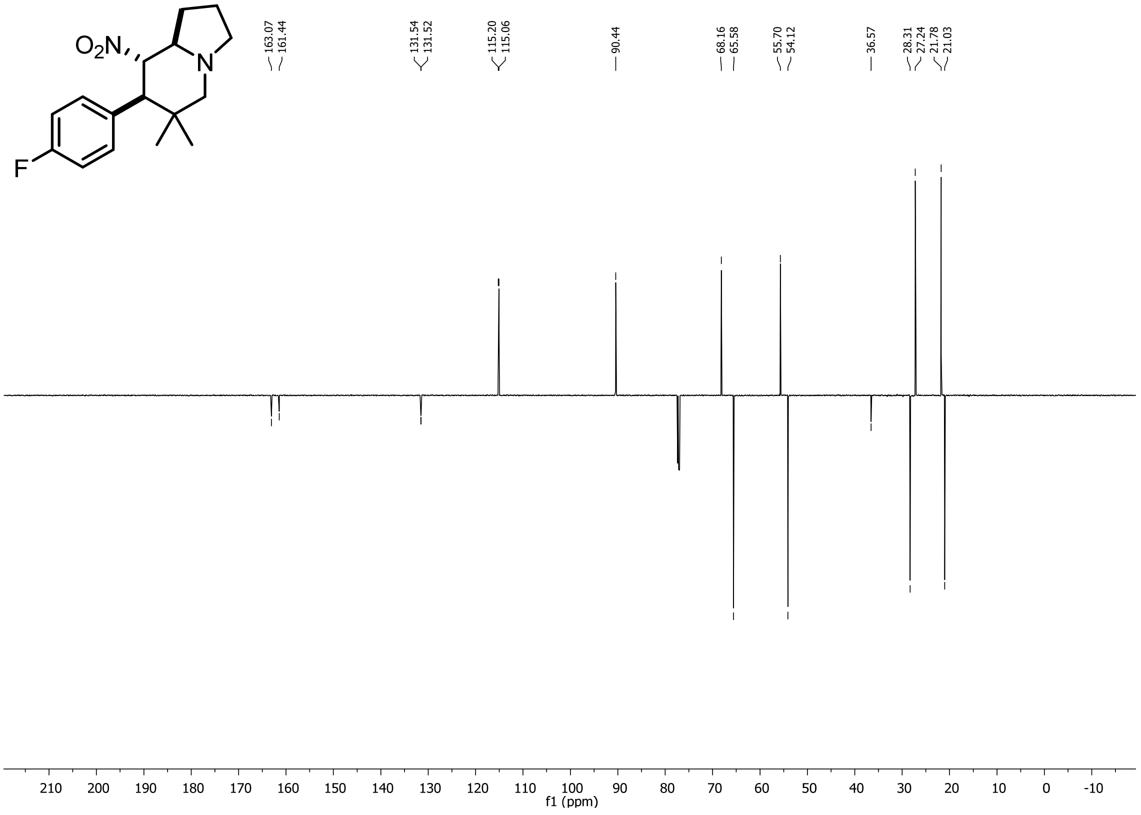

5r – 7-(4-fluorophenyl)-6,6-dimethyl-8-nitrooctahydroindolizine – ^{19}F NMR (565 MHz, $CDCl_3$)

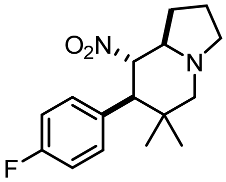

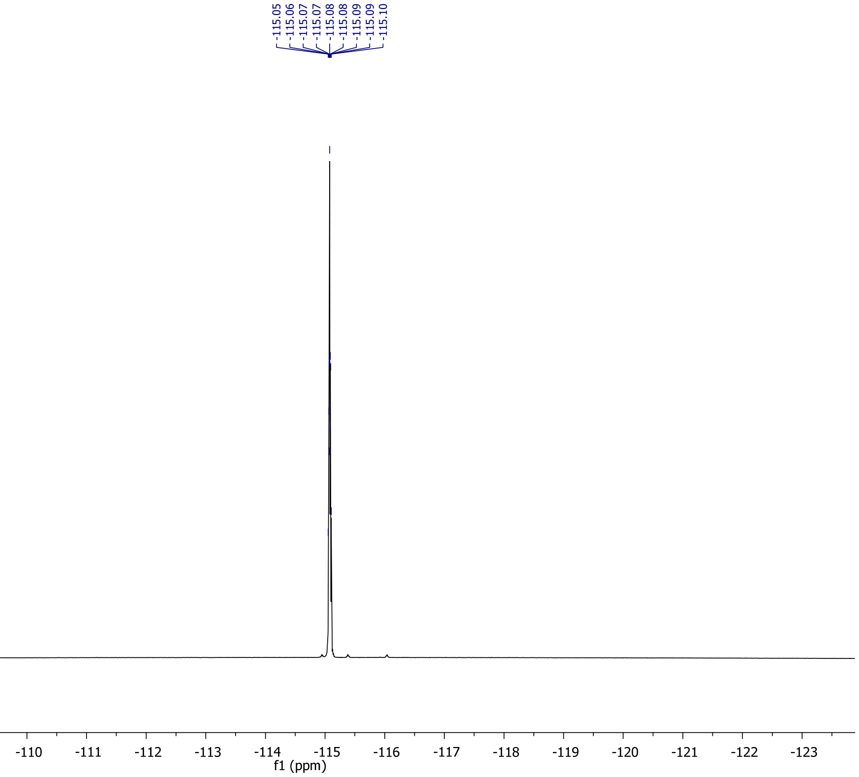

6.1.1.44 5s – 7-(4-chlorophenyl)-6,6-dimethyl-8-nitrooctahydroindolizine – ^{1}H NMR (600 MHz, $CDCl_3$)

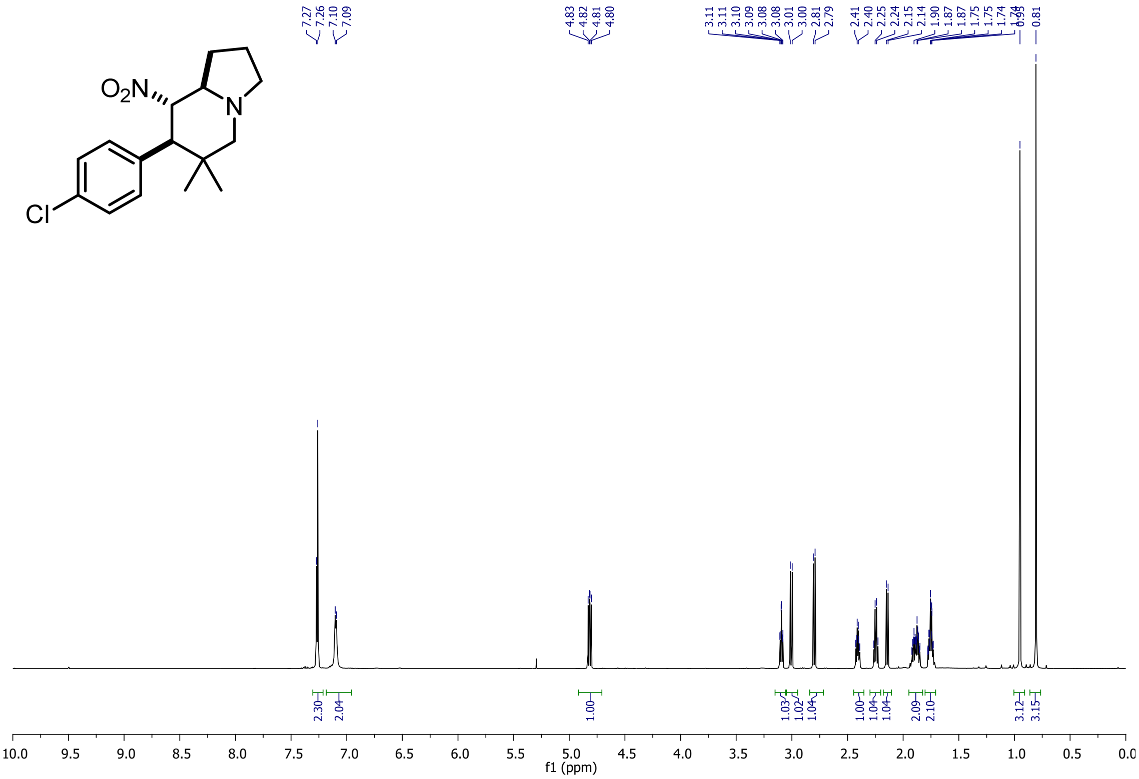

5s – 7-(4-chlorophenyl)-6,6-dimethyl-8-nitrooctahydroindolizine – ^{13}C NMR (151 MHz, $CDCl_3$)

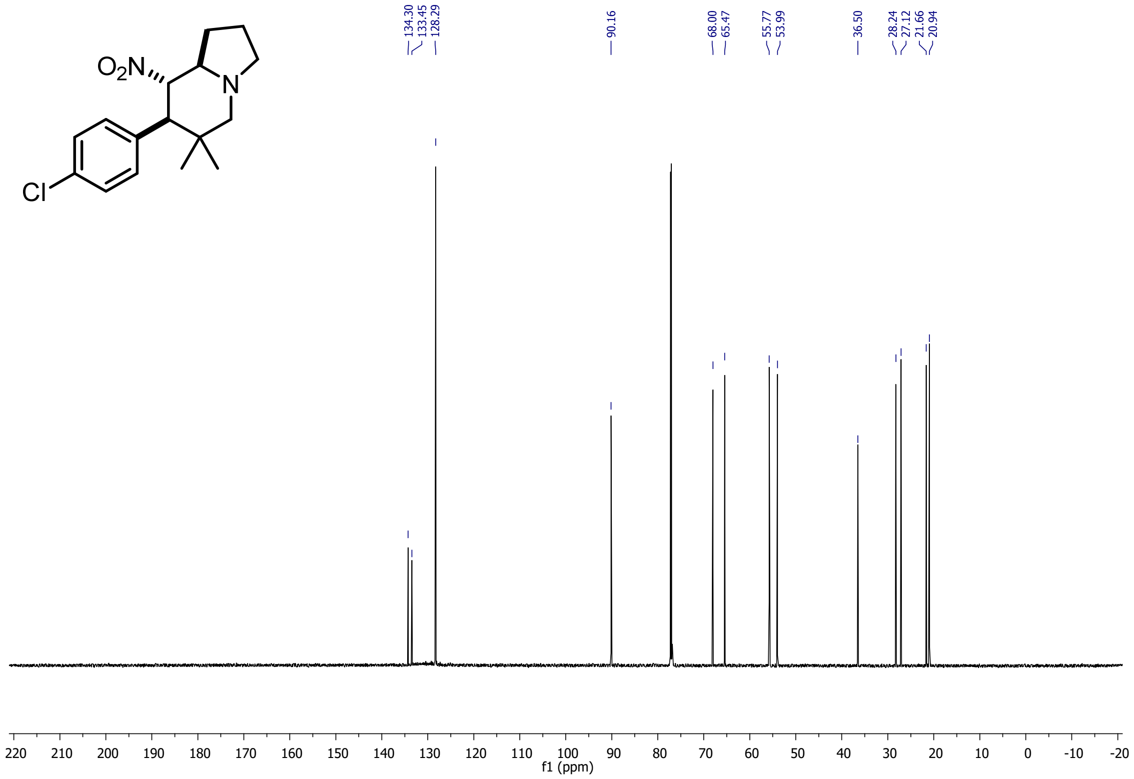

6.1.1.45 5t – 7-(2-bromophenyl)-6,6-dimethyl-8-nitrooctahydroindolizine – ^{1}H NMR (600 MHz, $CDCl_3$)

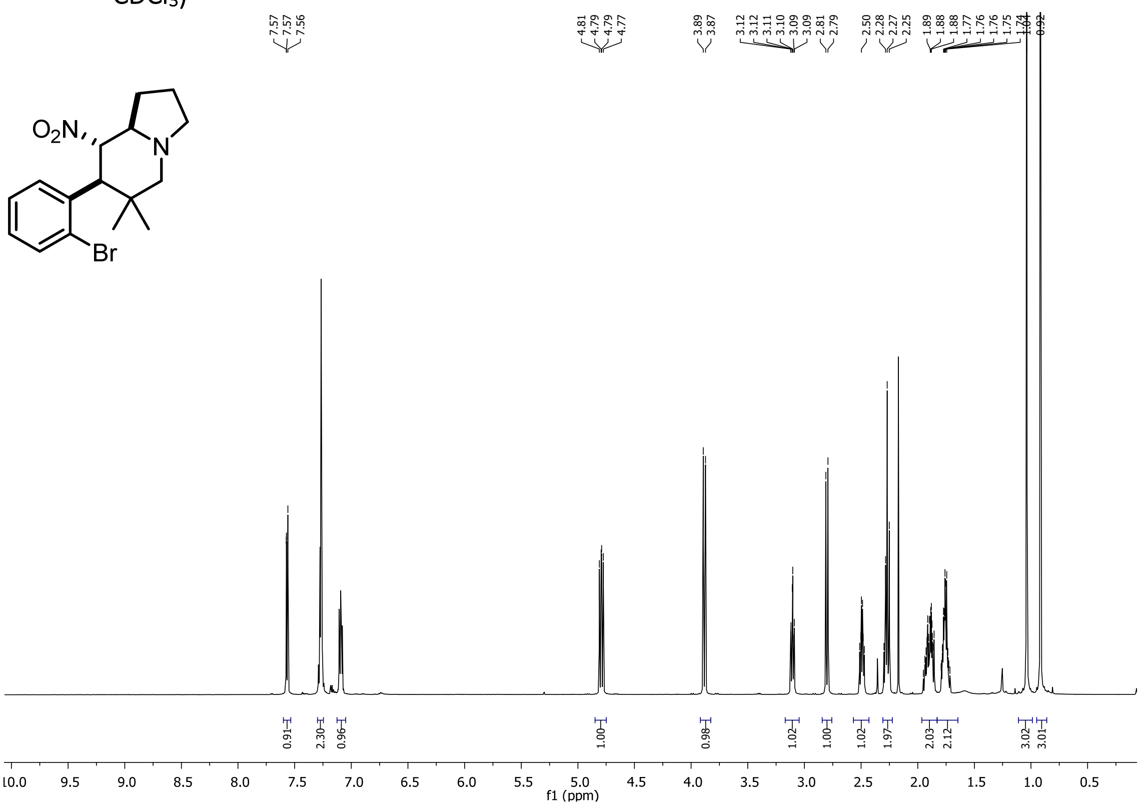

5t – 7-(2-bromophenyl)-6,6-dimethyl-8-nitrooctahydroindolizine – ^{13}C NMR (151 MHz, $CDCl_3$)

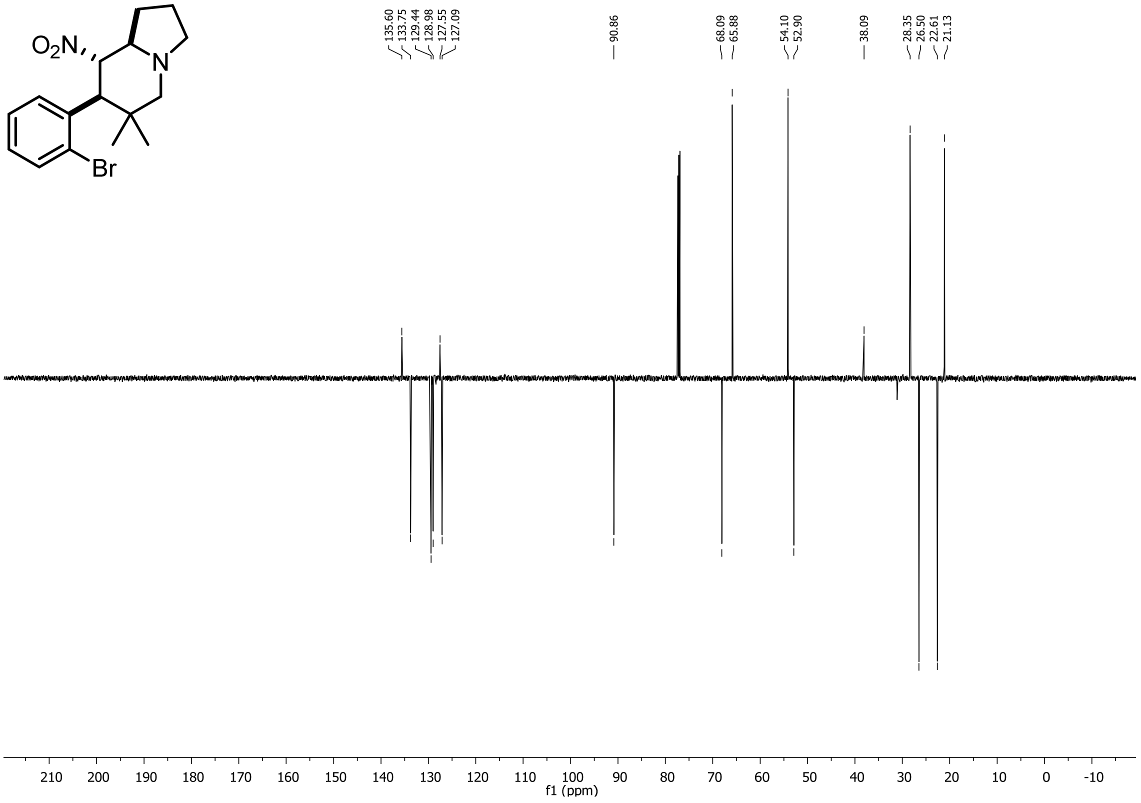

6.1.1.46 5u – tert-butyl 2-(6,6-dimethyl-8-nitrooctahydroindolizin-7-yl)-1H-pyrrole-1-carboxylate – ^{1}H NMR (600 MHz, $CDCl_3$)

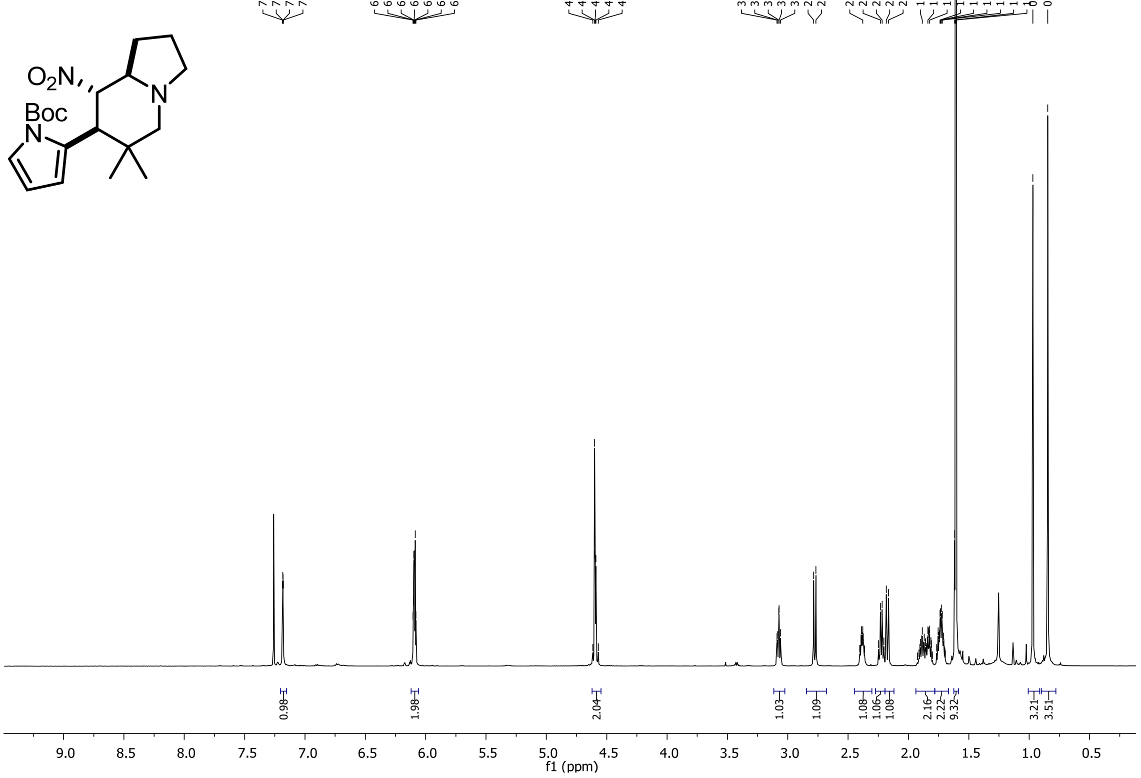

5u – tert-butyl 2-(6,6-dimethyl-8-nitrooctahydroindolizin-7-yl)-1H-pyrrole-1-carboxylate – ^{13}C NMR (151 MHz, $CDCl_3$)

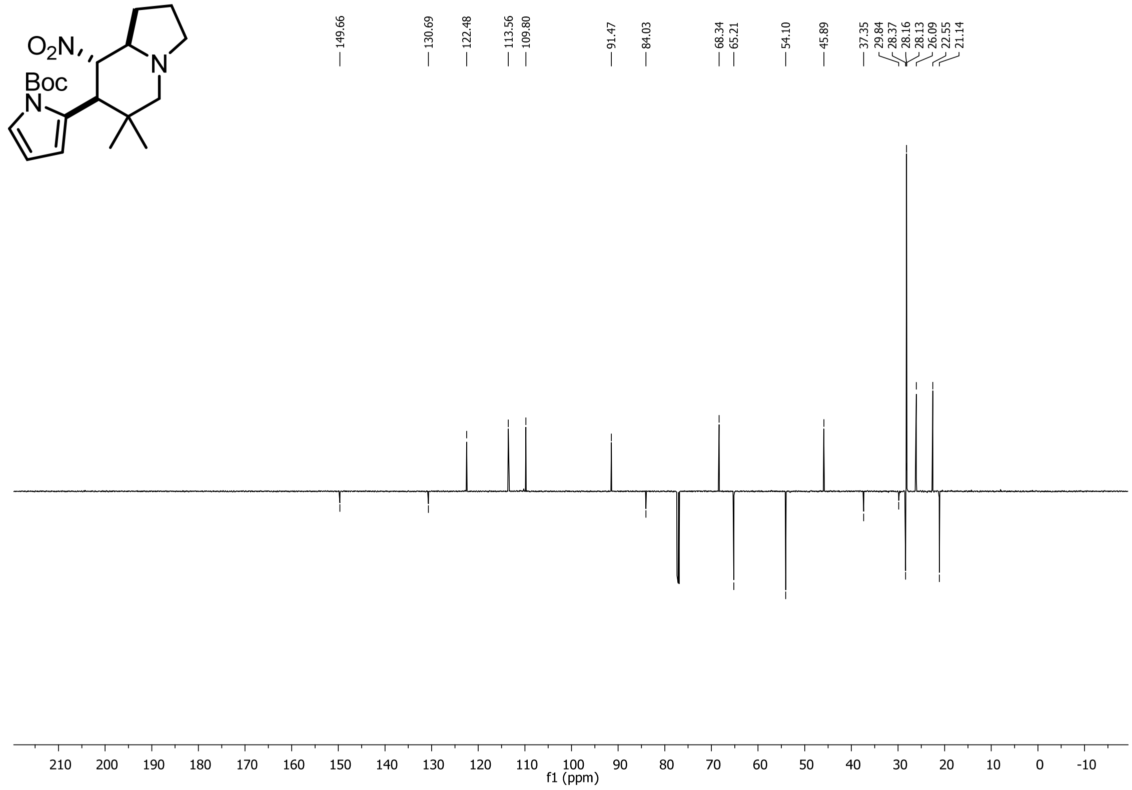

6.1.1.47 5v – 6,6-dimethyl-8-nitro-7-(thiophen-2-yl)octahydroindolizine – ^{1}H NMR (600 MHz, $CDCl_3$)

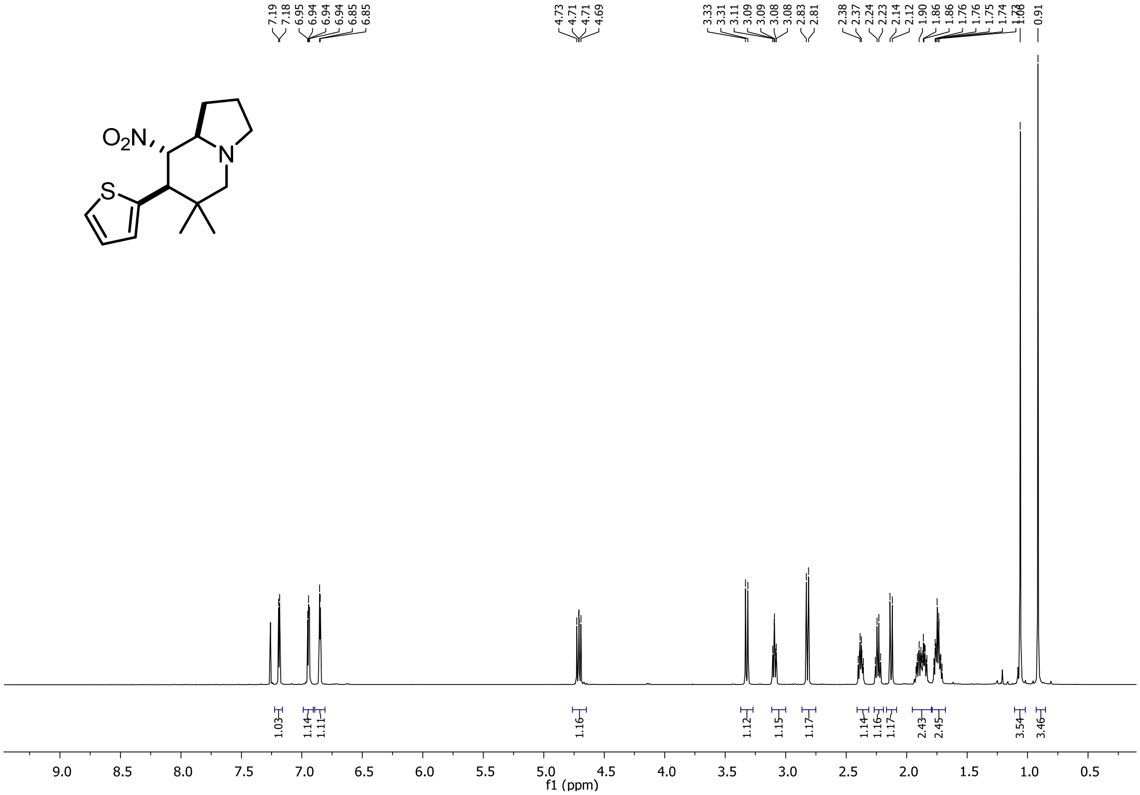

5v – 6,6-dimethyl-8-nitro-7-(thiophen-2-yl)octahydroindolizine – ^{13}C NMR (151 MHz, $CDCl_3$)

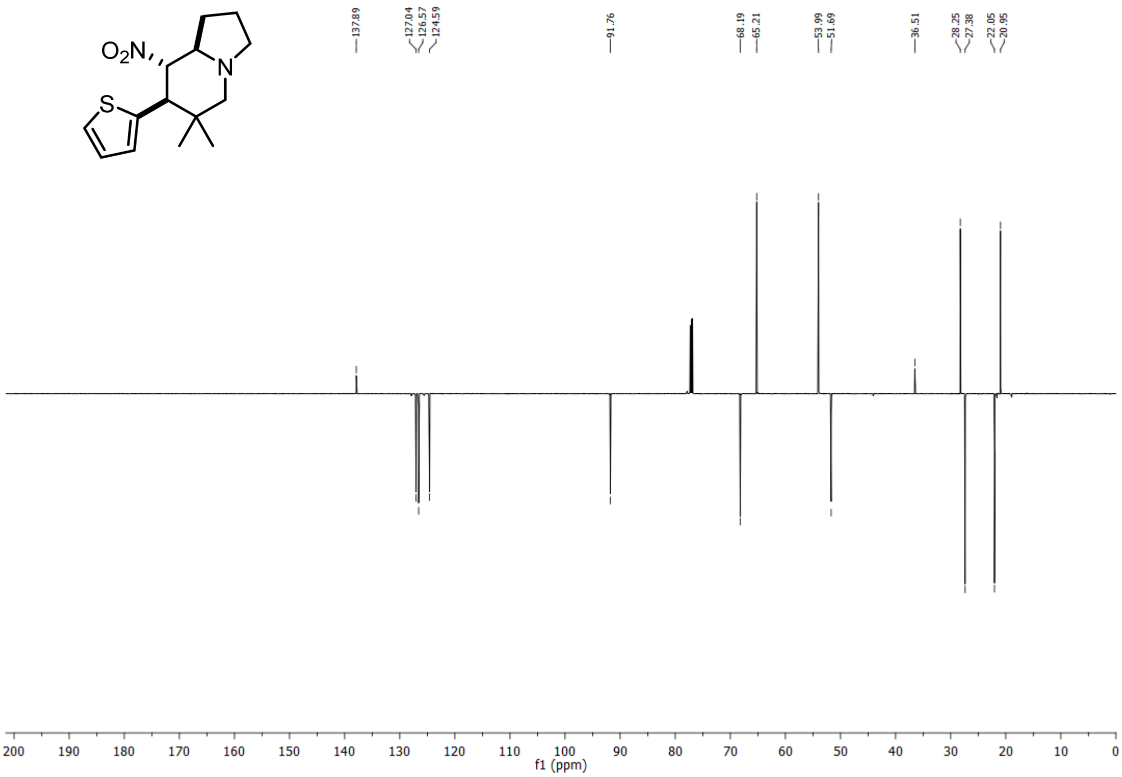

6.1.1.48 5w – 3-(6,6-dimethyl-8-nitrooctahydroindolizin-7-yl)-5-methylisoxazole – ^{1}H NMR (400 MHz, $CDCl_3$)

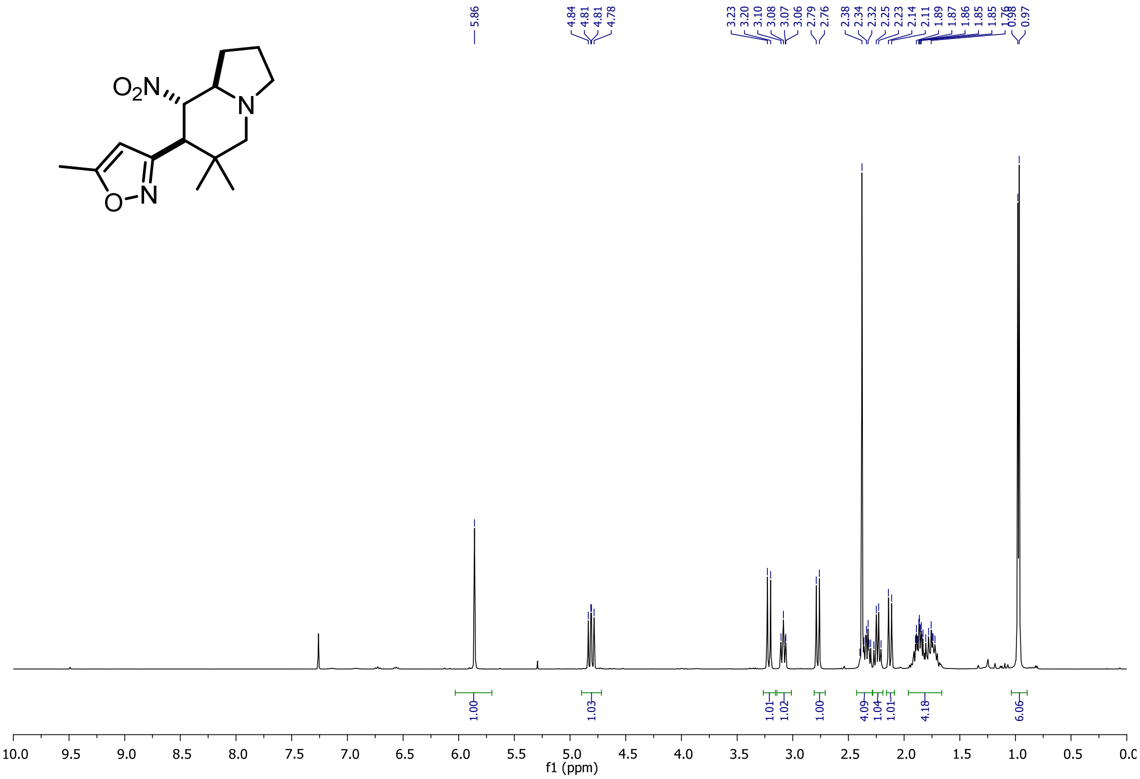

5w – 3-(6,6-dimethyl-8-nitrooctahydroindolizin-7-yl)-5-methylisoxazole – ^{13}C NMR (151 MHz, $CDCl_3$)

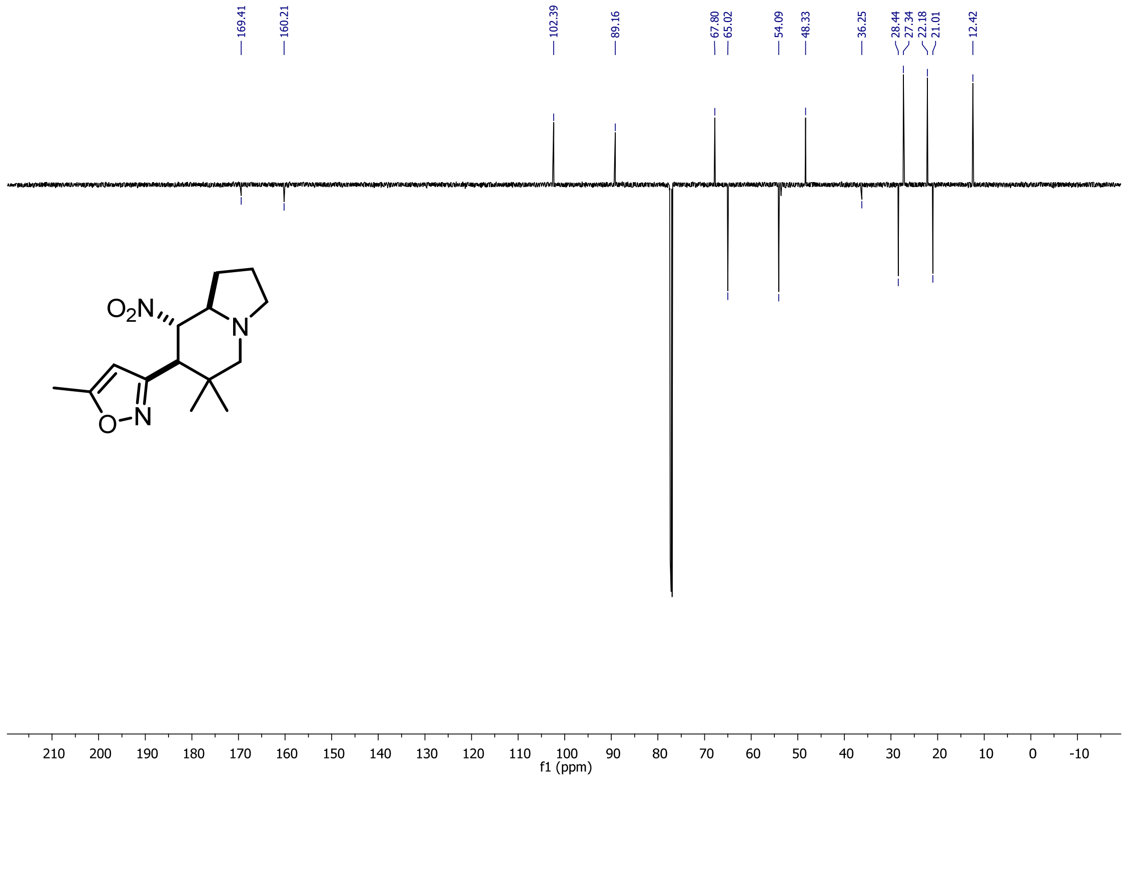

6.1.1.49 5x – 7-cyclopropyl-6,6-dimethyl-8-nitrooctahydroindolizine – ^{1}H NMR (600 MHz, $CDCl_3$)

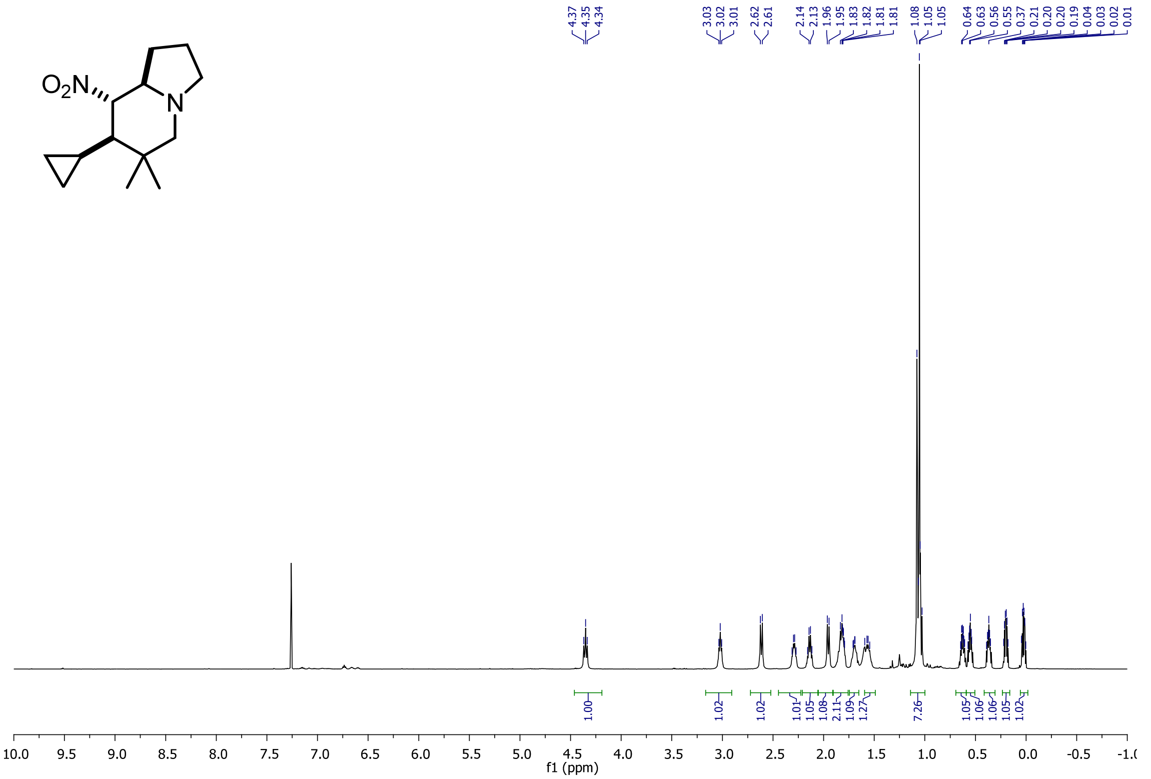

5x – 7-cyclopropyl-6,6-dimethyl-8-nitrooctahydroindolizine – ^{13}C NMR (151 MHz, $CDCl_3$)

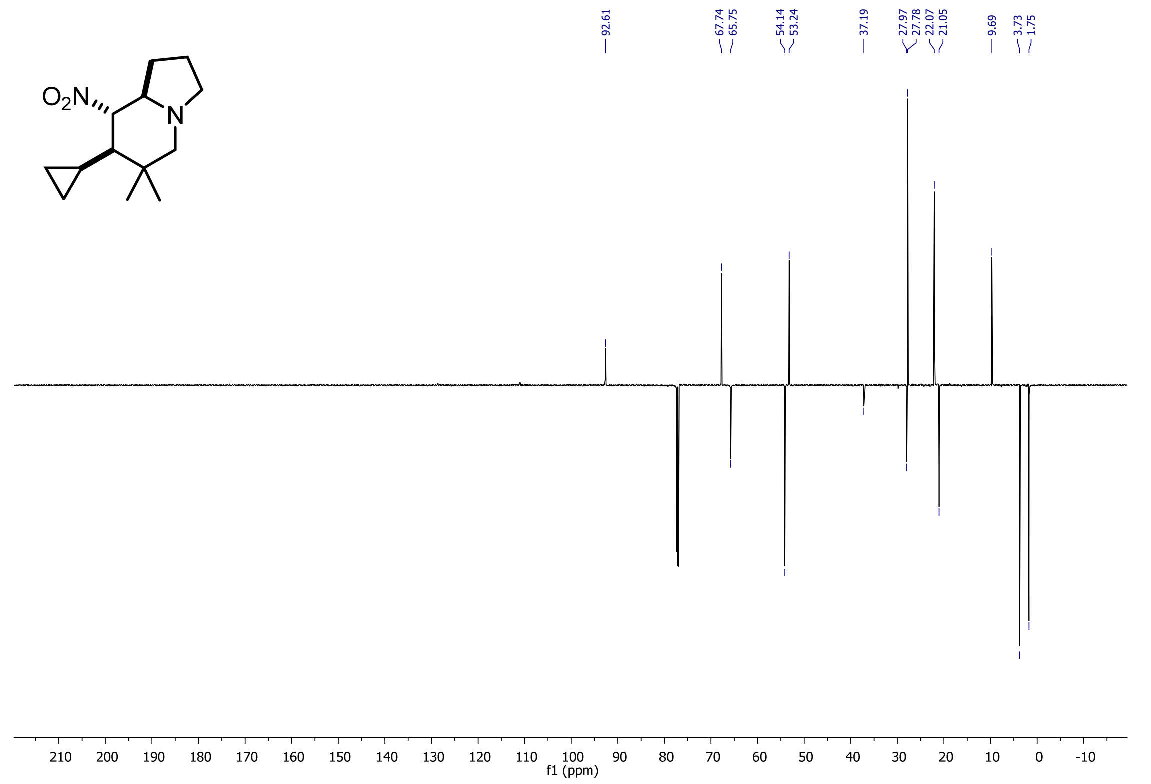

6.1.1.50 5y – 6,6-dimethyl-8-nitro-7-((E)-styryl)octahydroindolizine – ^{1}H NMR (600 MHz, $CDCl_3$)

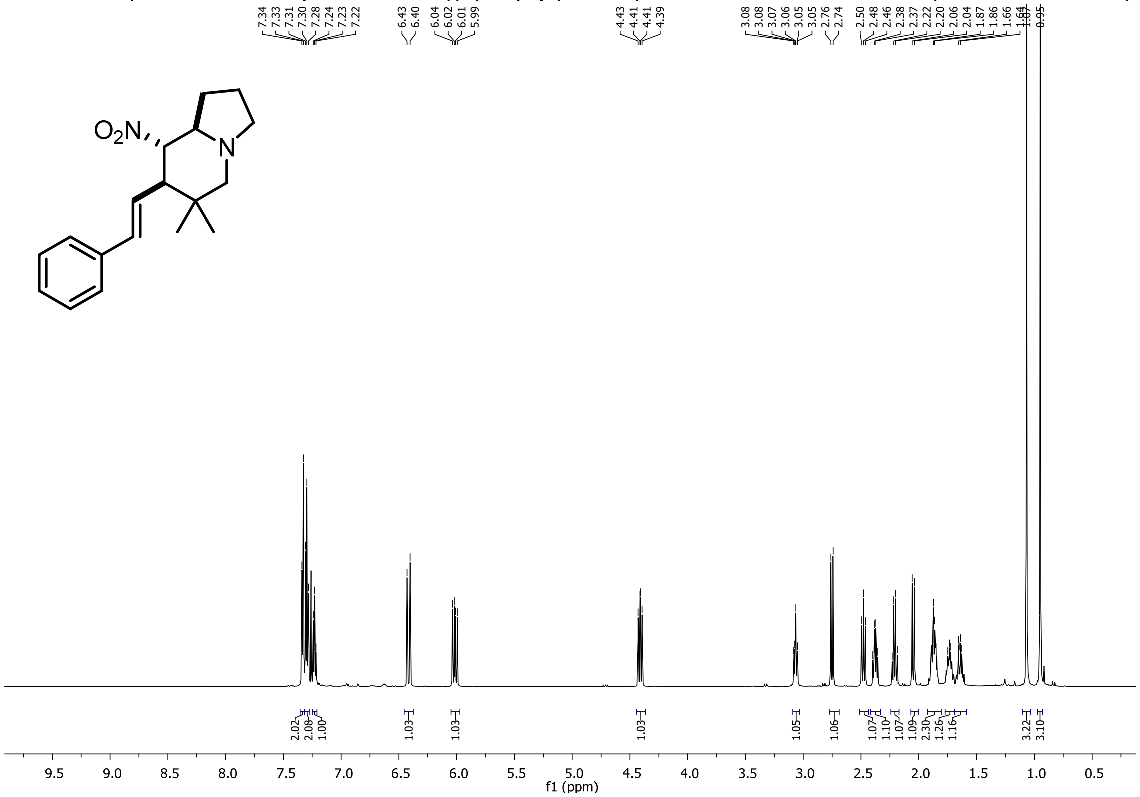

5y – 6,6-dimethyl-8-nitro-7-((E)-styryl)octahydroindolizine – ^{13}C NMR (151 MHz, $CDCl_3$)

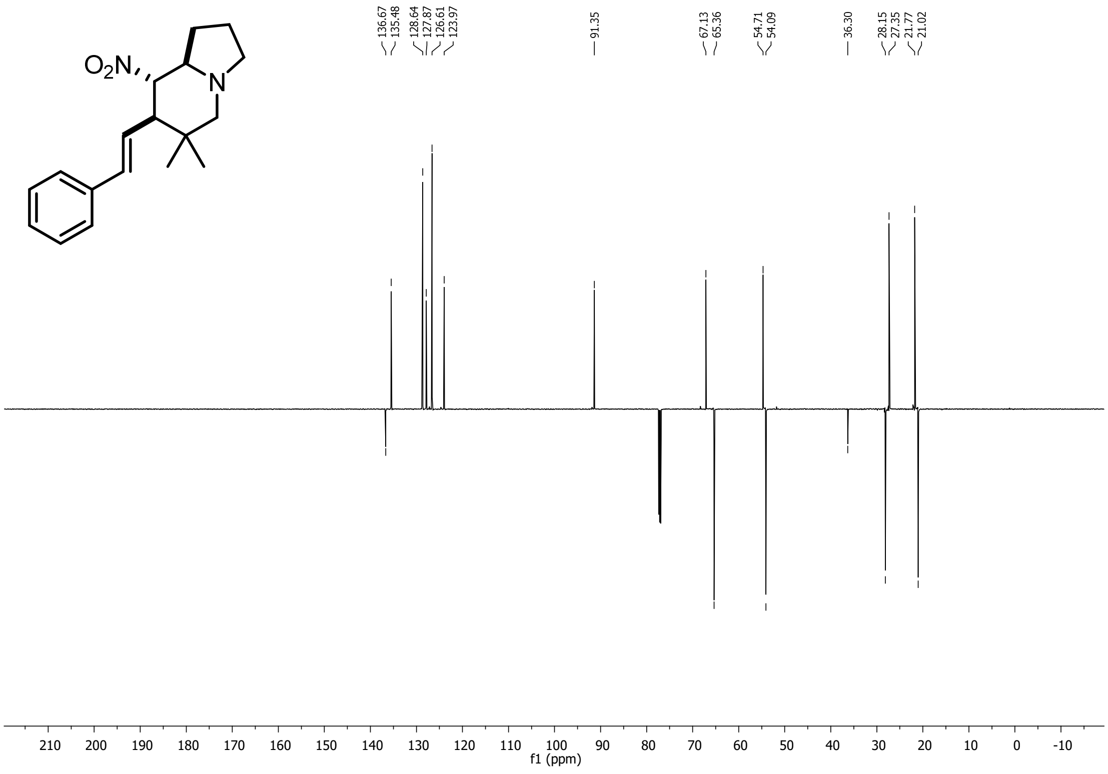

6.1.1.51 5z – 6,6-dimethyl-8-nitro-7-(4-phenylbut-1-yn-1-yl)octahydroindolizine – ^{1}H NMR (600 MHz, $CDCl_3$)

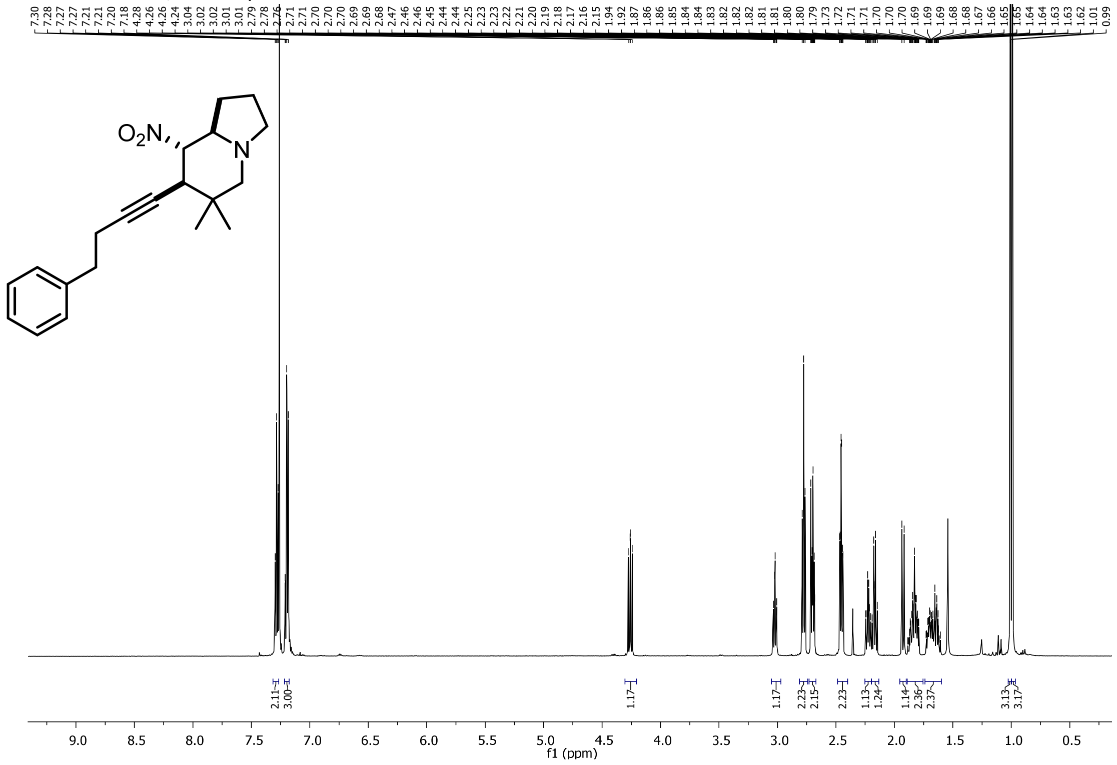

5z – 6,6-dimethyl-8-nitro-7-(4-phenylbut-1-yn-1-yl)octahydroindolizine – ^{13}C NMR (151 MHz, $CDCl_3$)

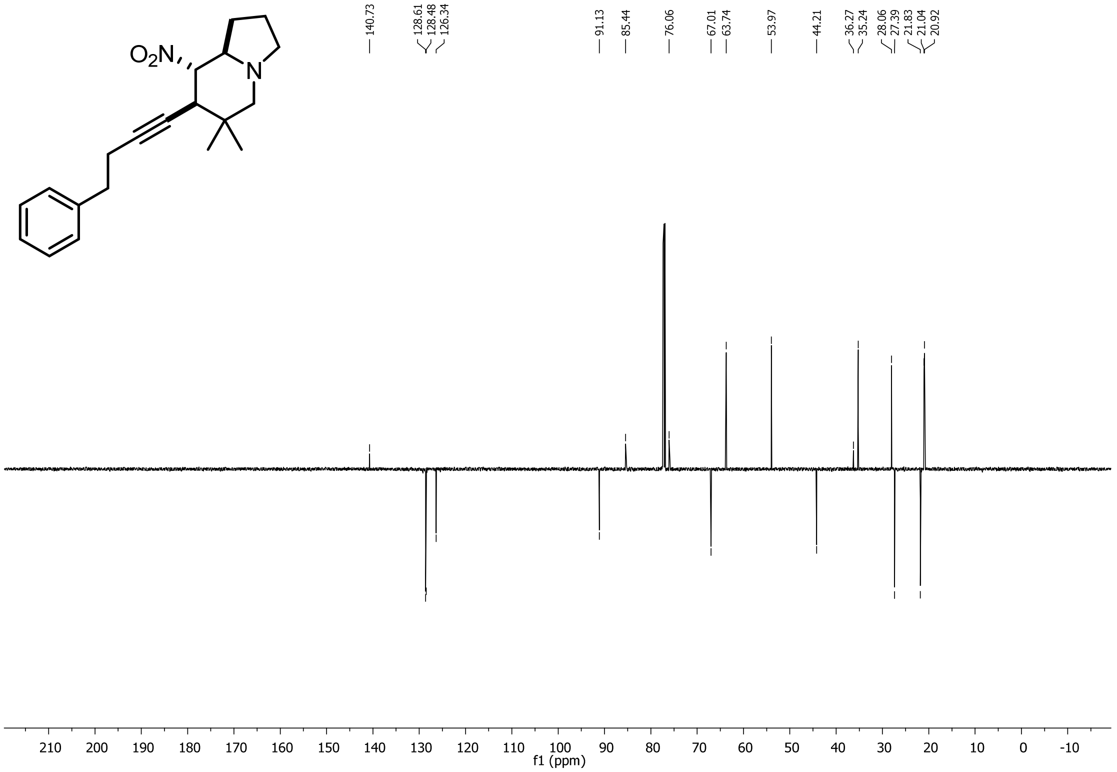

6.1.1.52 5aa – 3,3-dimethyl-1-nitro-2,7-diphenyloctahydro-2H-quinolizine – ^{1}H NMR (600 MHz, $CDCl_3$)

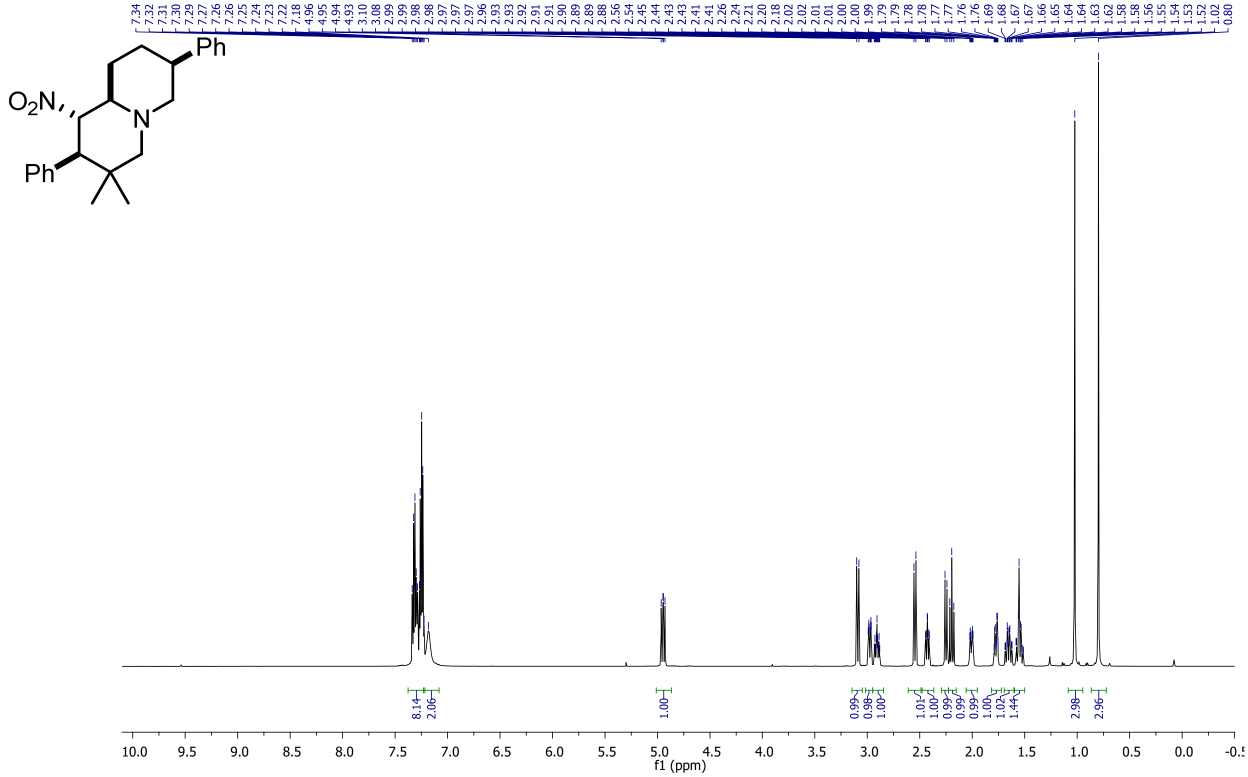

5aa – 3,3-dimethyl-1-nitro-2,7-diphenyloctahydro-2H-quinolizine – ^{13}C NMR (151 MHz, $CDCl_3$)

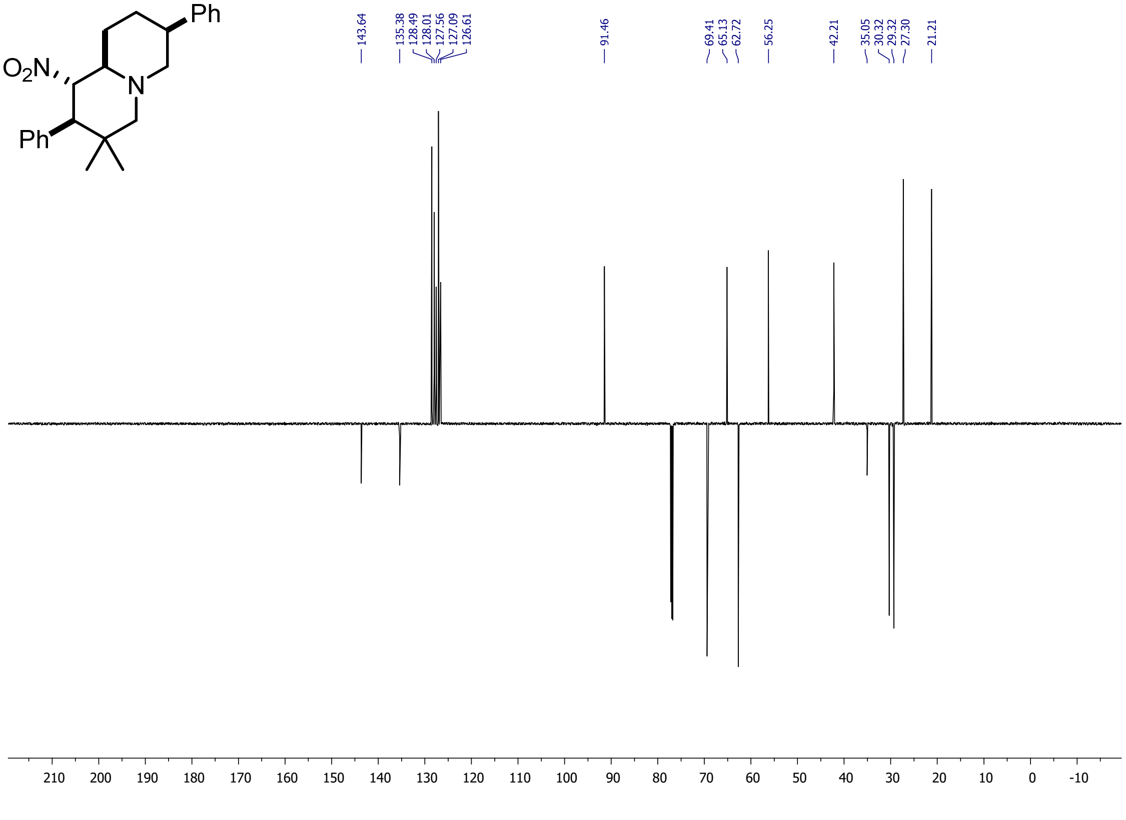

6.1.1.1 S2a – (*S*)-2,2-dimethyl-4-nitro-3-phenylbutanal – ^{1}H NMR (600 MHz, $CDCl_3$)

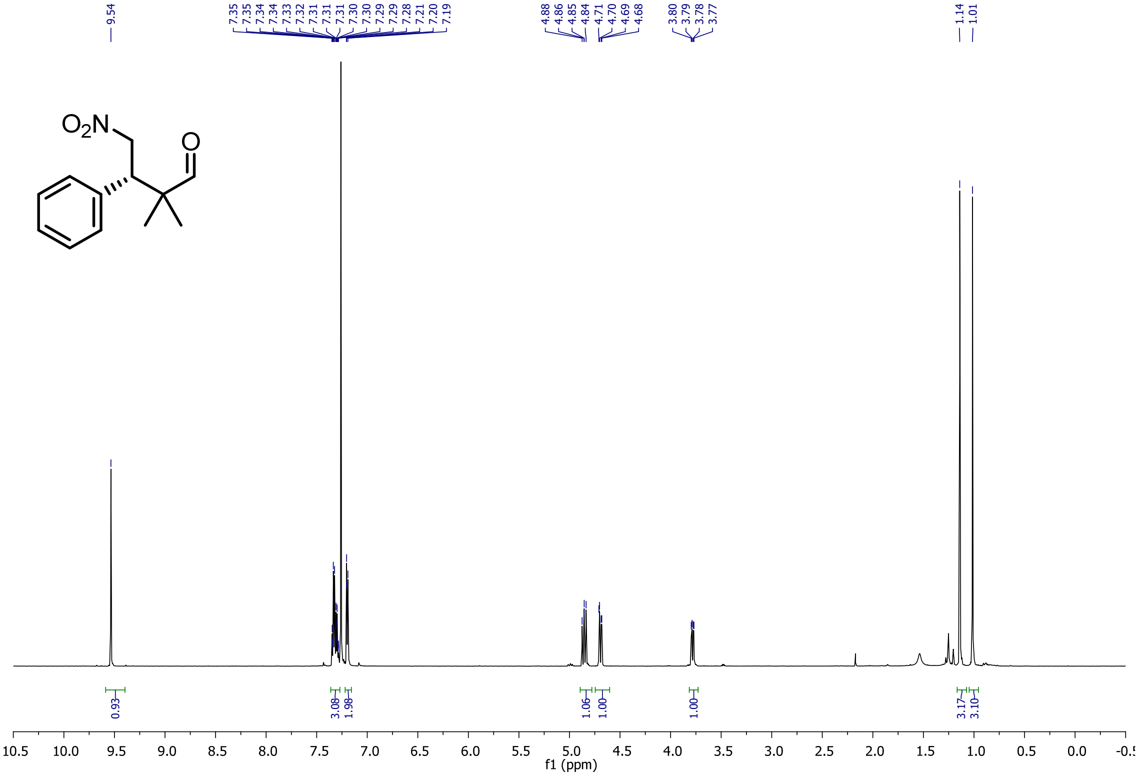

S2a – (*S*)-2,2-dimethyl-4-nitro-3-phenylbutanal – ^{13}C NMR (151 MHz, $CDCl_3$)

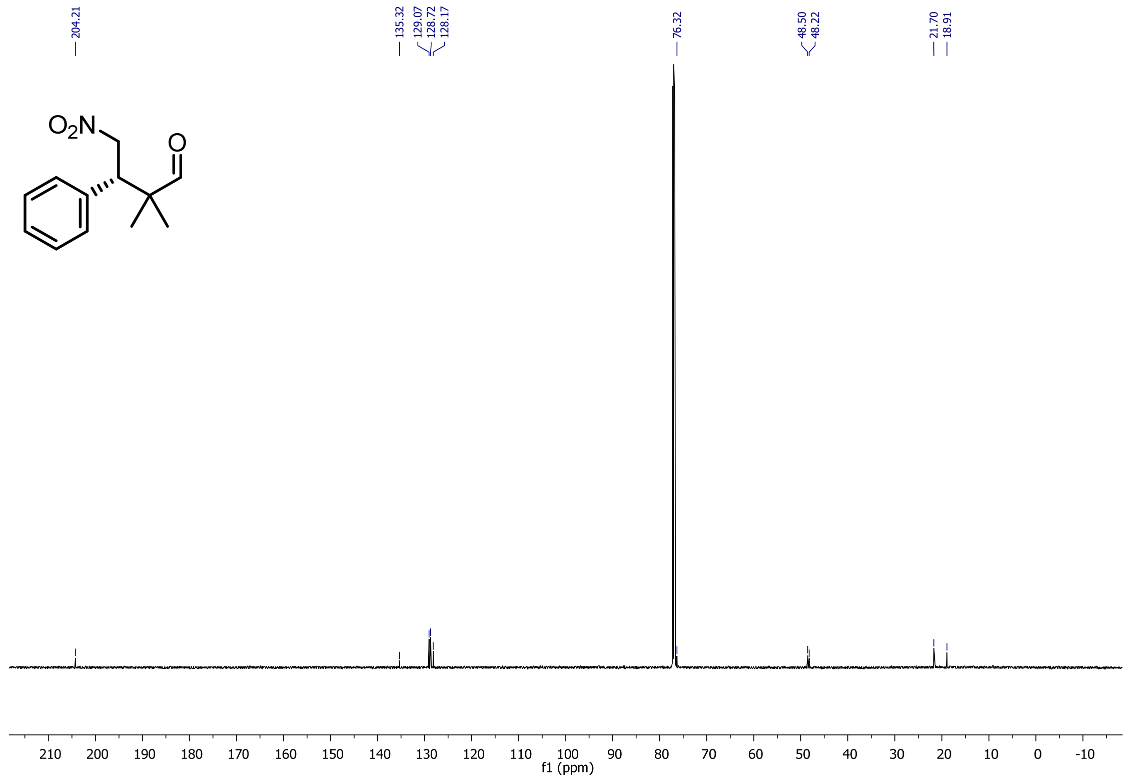

6.1.1.2 S2b – (*S*)-3-(4-methoxyphenyl)-2,2-dimethyl-4-nitrobutanal – ^{1}H NMR (700 MHz, $CDCl_3$)

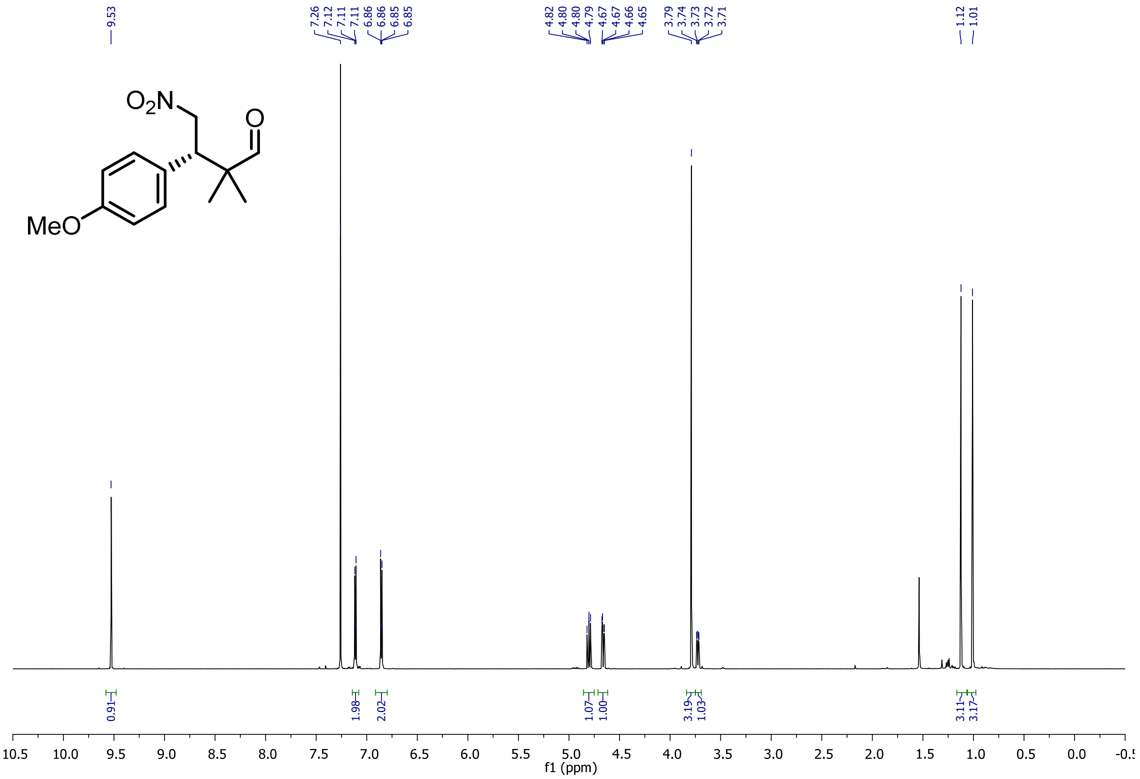

S2b – (*S*)-3-(4-methoxyphenyl)-2,2-dimethyl-4-nitrobutanal – ^{13}C NMR (176 MHz, $CDCl_3$)

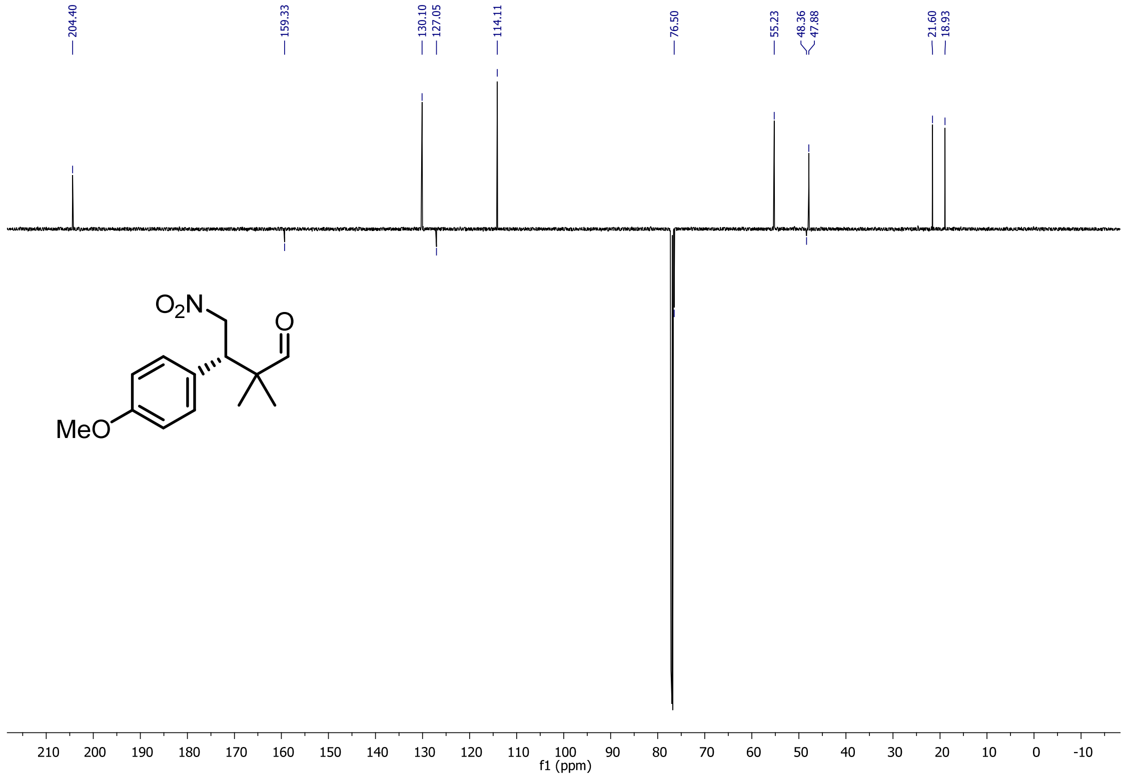

6.1.1.3 S2c – (*S*)-2,2-dimethyl-4-nitro-3-(4-nitrophenyl)butanal – ^{1}H NMR (700 MHz, $CDCl_3$)

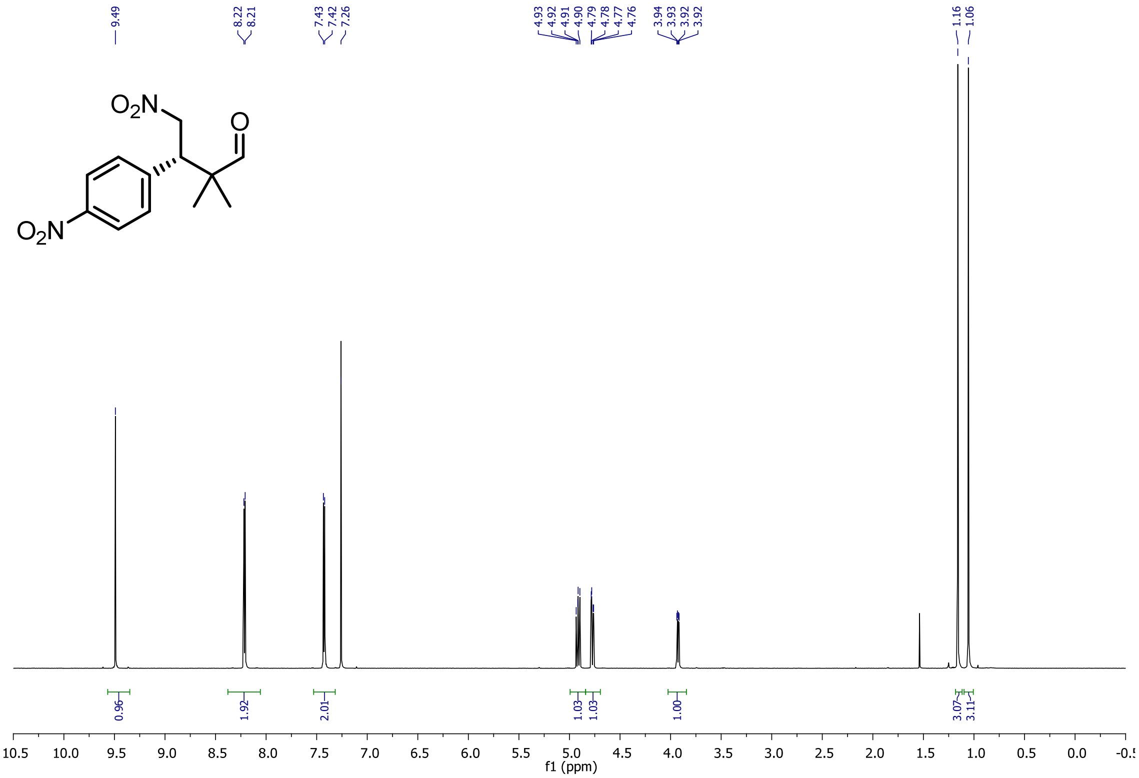

S2c – (*S*)-2,2-dimethyl-4-nitro-3-(4-nitrophenyl)butanal – ^{13}C NMR (176 MHz, $CDCl_3$)

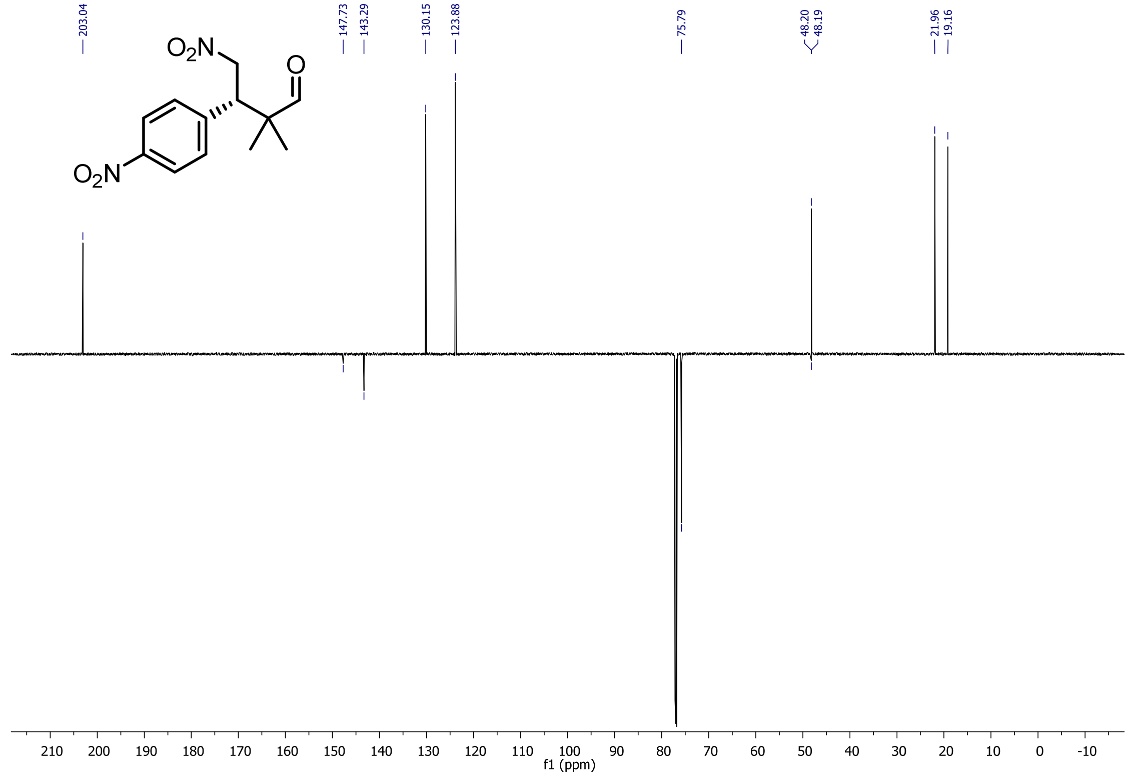

6.1.1.4 S2d – (*S*)-3-(4-fluorophenyl)-2,2-dimethyl-4-nitrobutanal – ^{1}H NMR (700 MHz, $CDCl_3$)

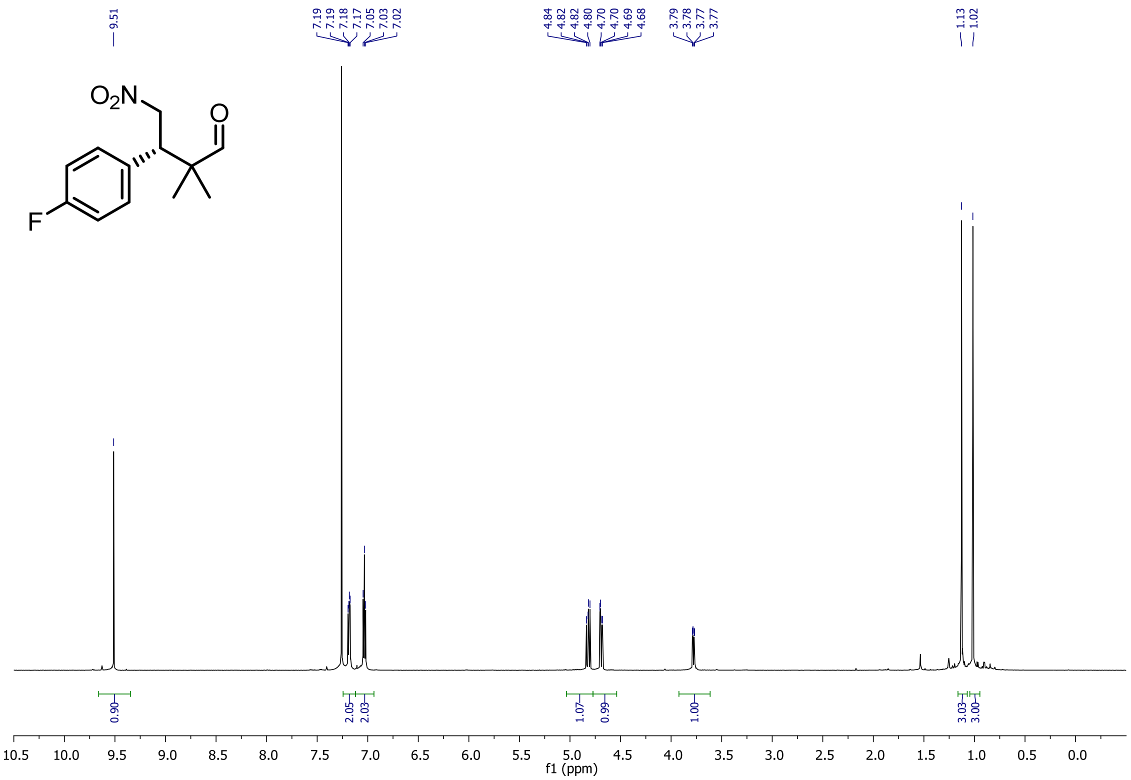

S2d – (*S*)-3-(4-fluorophenyl)-2,2-dimethyl-4-nitrobutanal – ^{13}C NMR (176 MHz, $CDCl_3$)

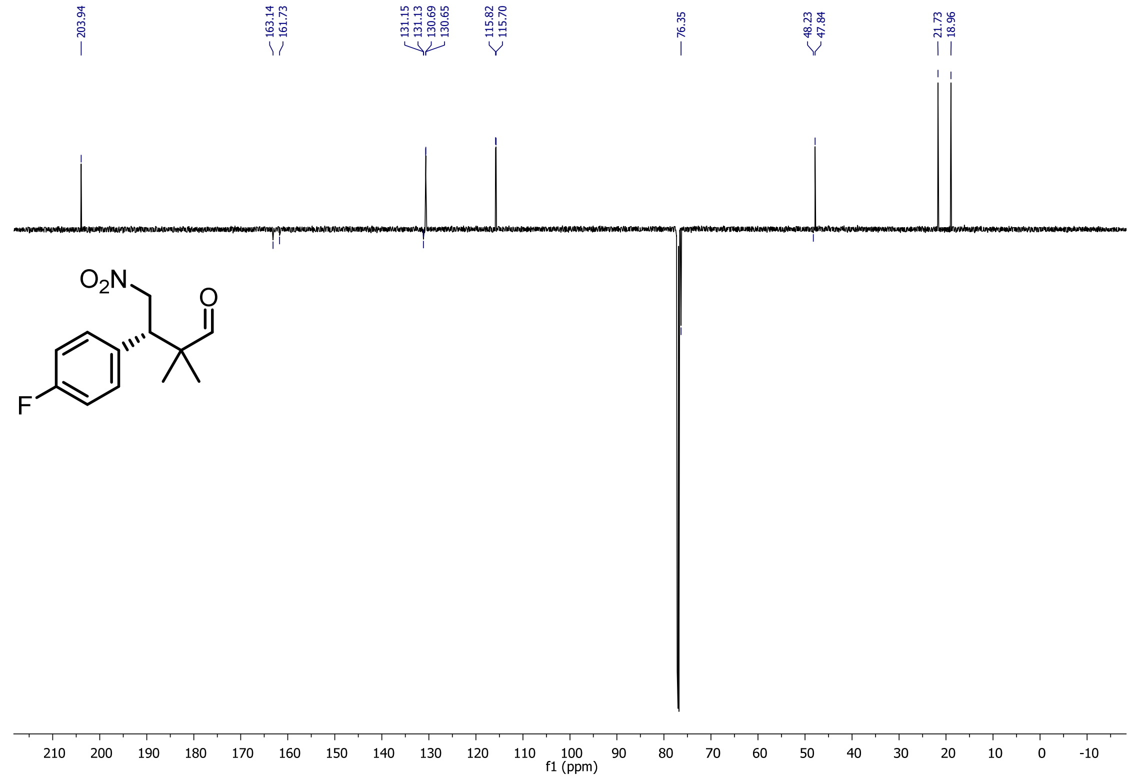

S2d – (*S*)-3-(4-fluorophenyl)-2,2-dimethyl-4-nitrobutanal – ^{19}F NMR (659 MHz, $CDCl_3$)

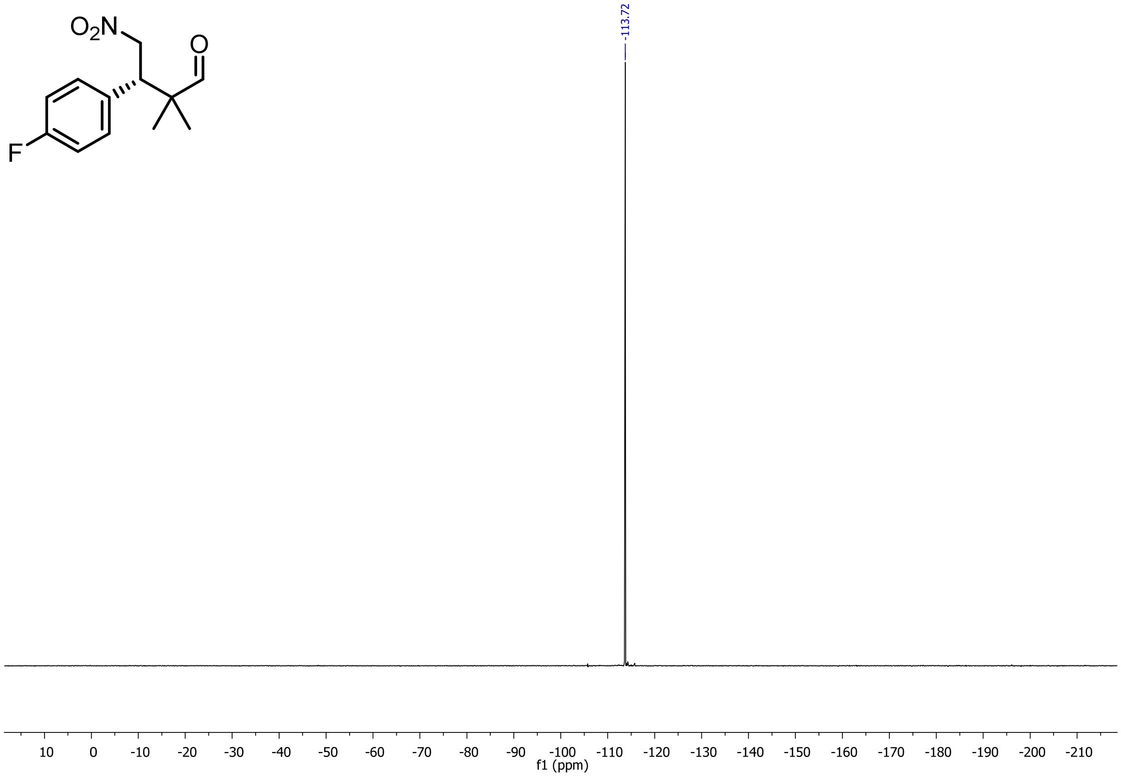

6.1.1.5 8c – 1-(2,2-dimethyl-4-nitro-3-phenylcyclobutyl)pyrrolidine – ^{1}H NMR (400 MHz, $CDCl_3$)

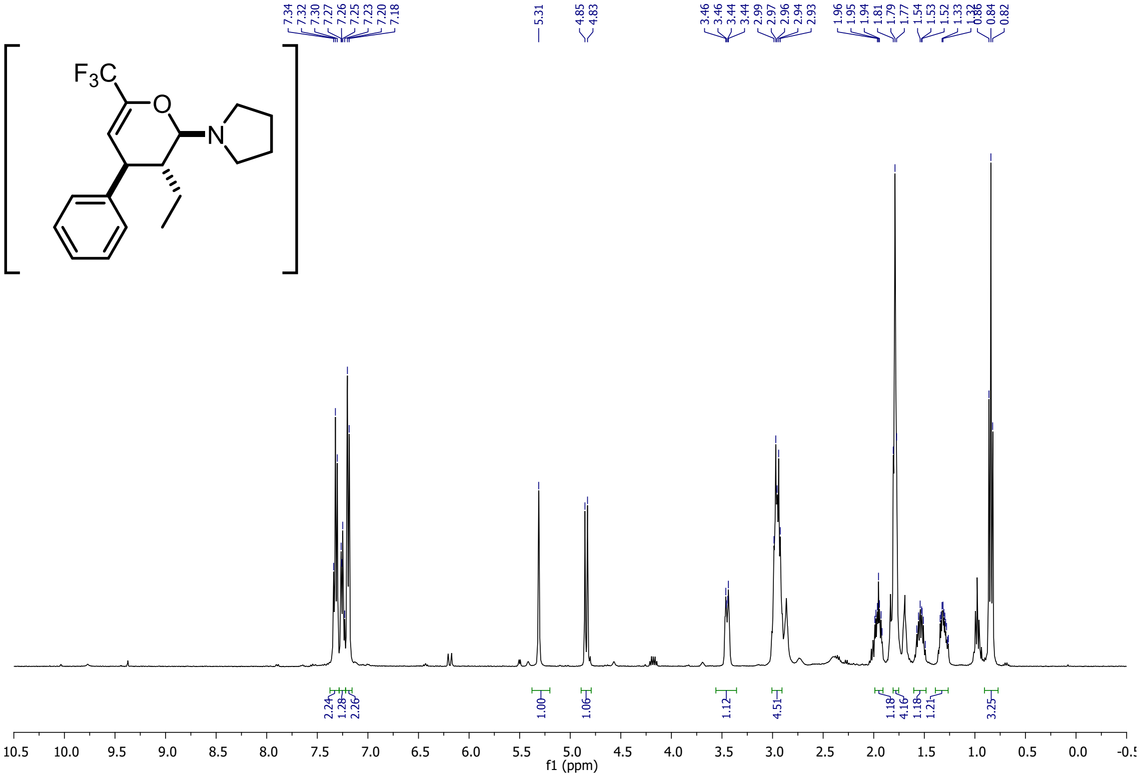

8c – 1-(2,2-dimethyl-4-nitro-3-phenylcyclobutyl)pyrrolidine – ^{19}F NMR (376 MHz, $CDCl_3$)

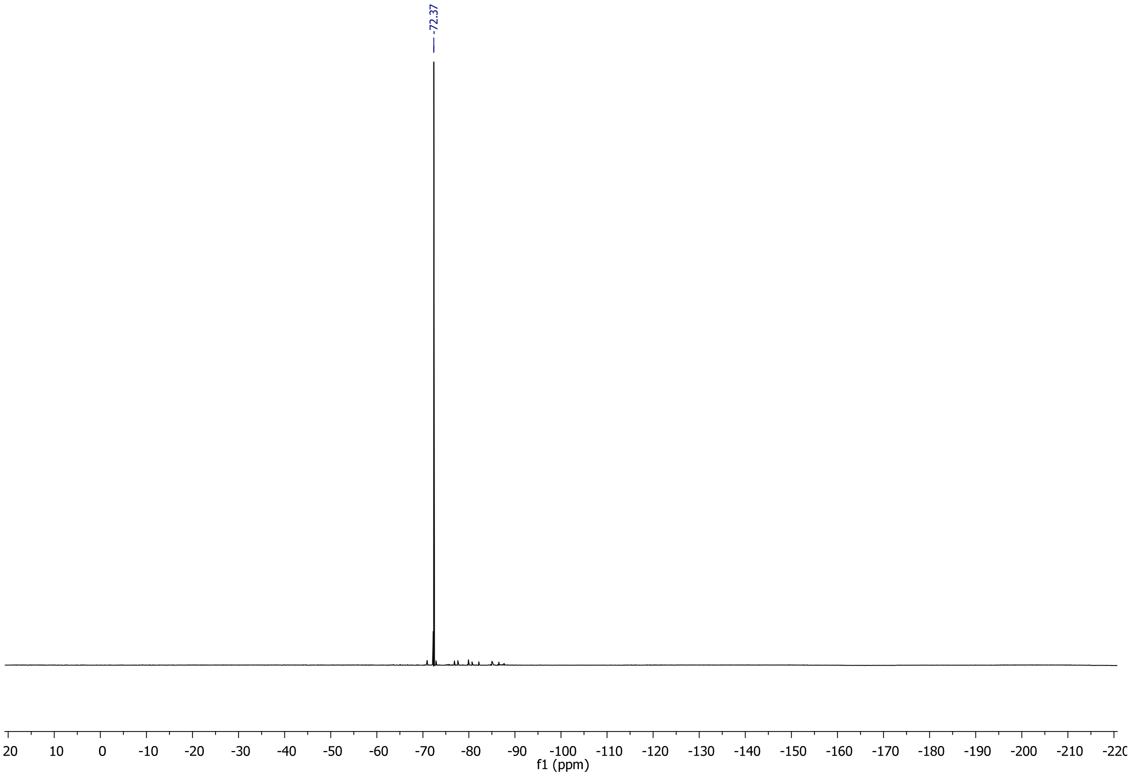

6.1.1.6 7a – 1-(6,6-dimethyl-7-phenyloctahydroindolizin-8-yl)-2,2,2-trifluoroethan-1-one – ^{1}H NMR (600 MHz, $CDCl_3$)

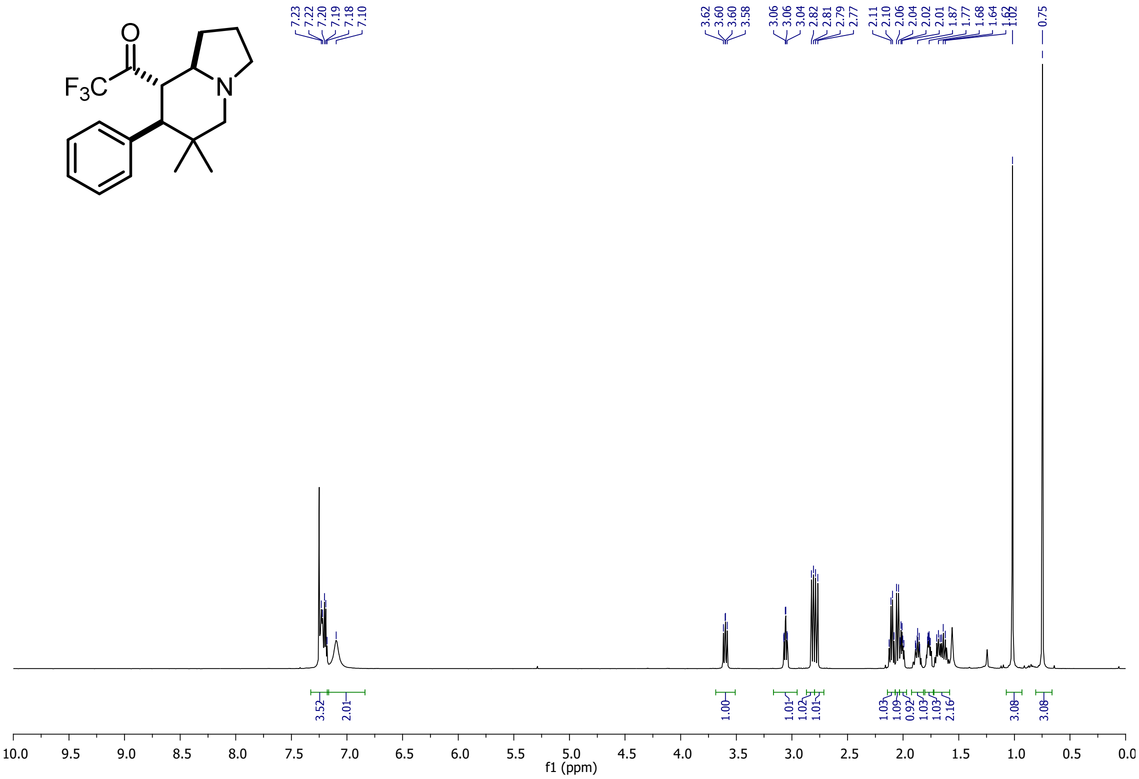

7a – 1-(6,6-dimethyl-7-phenyloctahydroindolizin-8-yl)-2,2,2-trifluoroethan-1-one – ^{13}C NMR (151 MHz, $CDCl_3$)

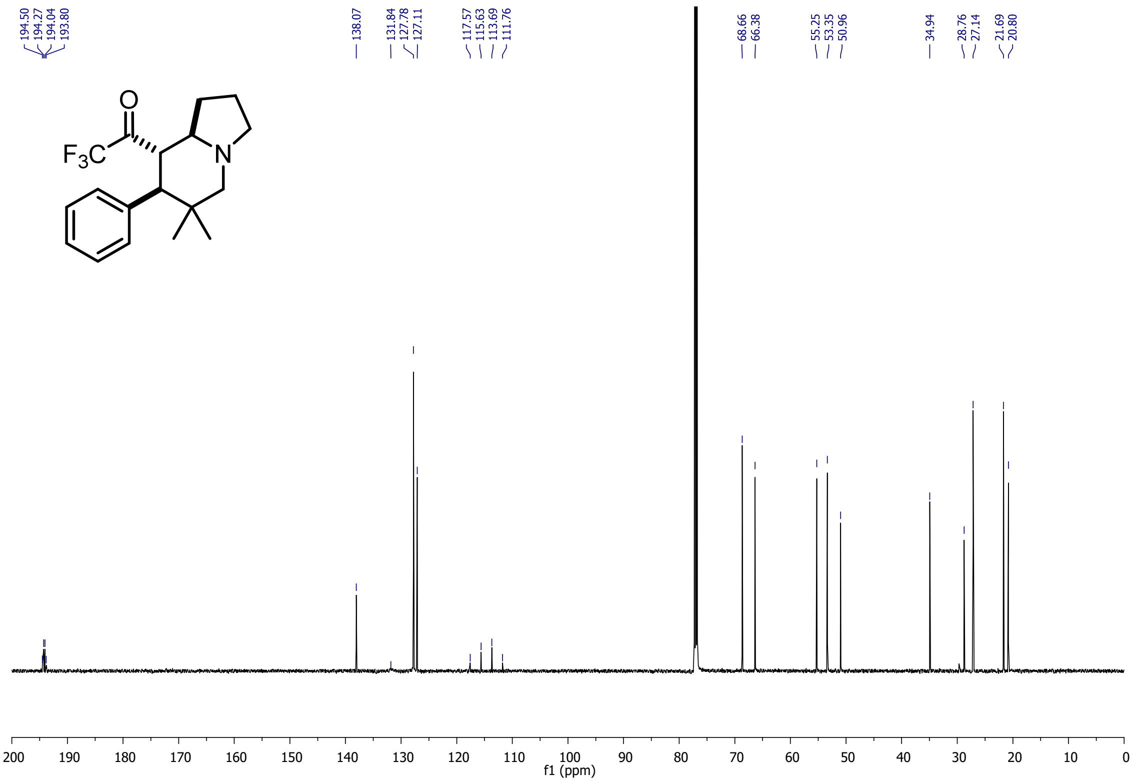

7a – 1-(6,6-dimethyl-7-phenyloctahydroindolizin-8-yl)-2,2,2-trifluoroethan-1-one – ^{19}F NMR (565 MHz, $CDCl_3$)

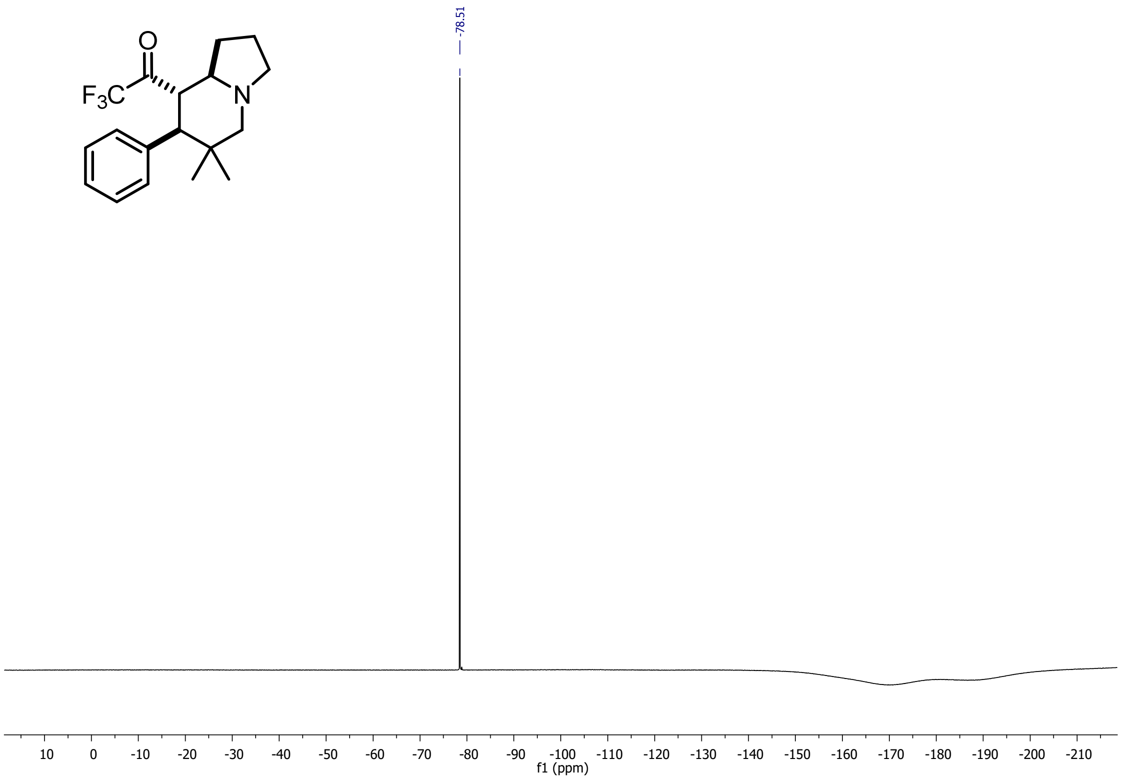

6.1.1.7 7b – 1-(6,6-dimethyl-7-((E)-styryl)octahydroindolizin-8-yl)-2,2,2-trifluoroethan-1-one – ^{1}H NMR (700 MHz, $CDCl_3$)

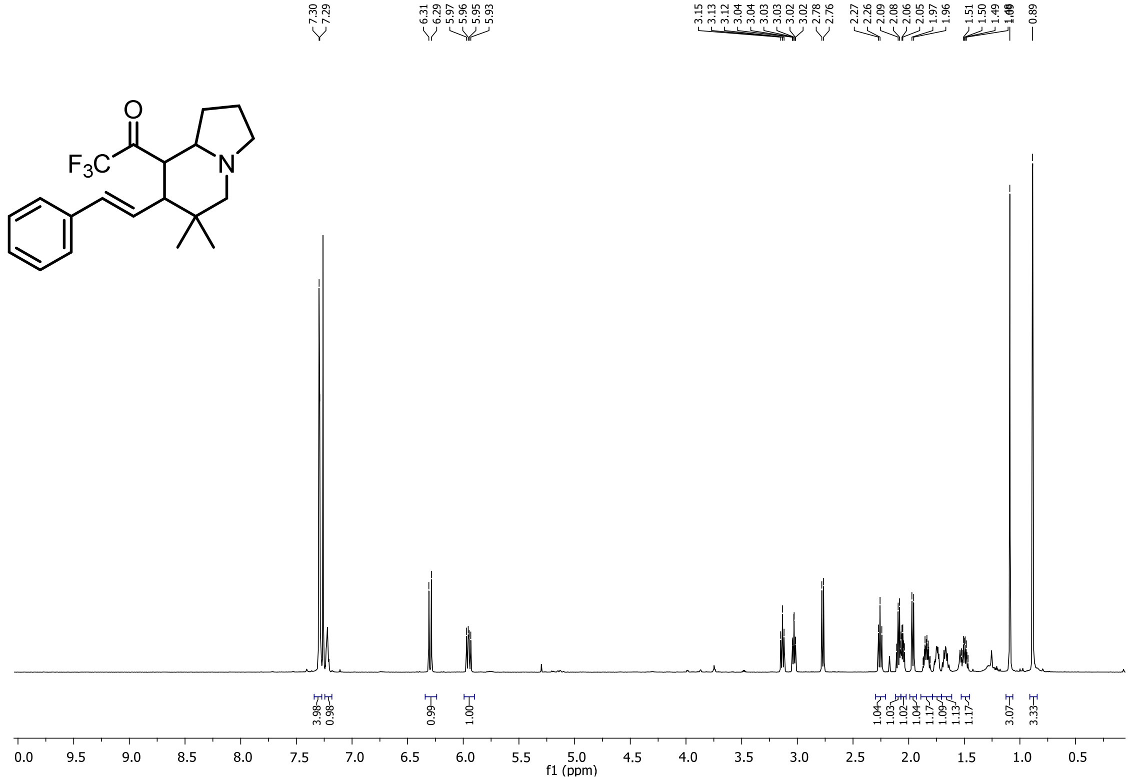

7b – 1-(6,6-dimethyl-7-((E)-styryl)octahydroindolizin-8-yl)-2,2,2-trifluoroethan-1-one – ^{13}C NMR (151 MHz, $CDCl_3$)

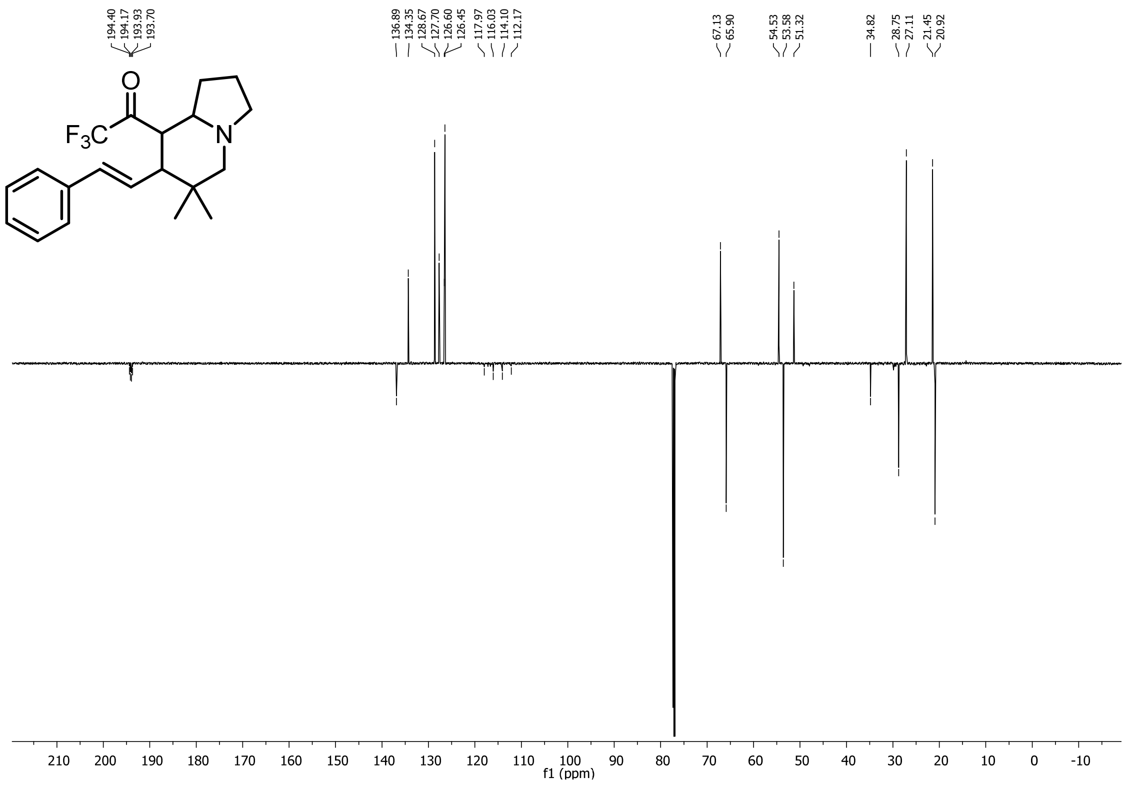

7b – 1-(6,6-dimethyl-7-((E)-styryl)octahydroindolizin-8-yl)-2,2,2-trifluoroethan-1-one – ^{19}F NMR (659 MHz, $CDCl_3$)

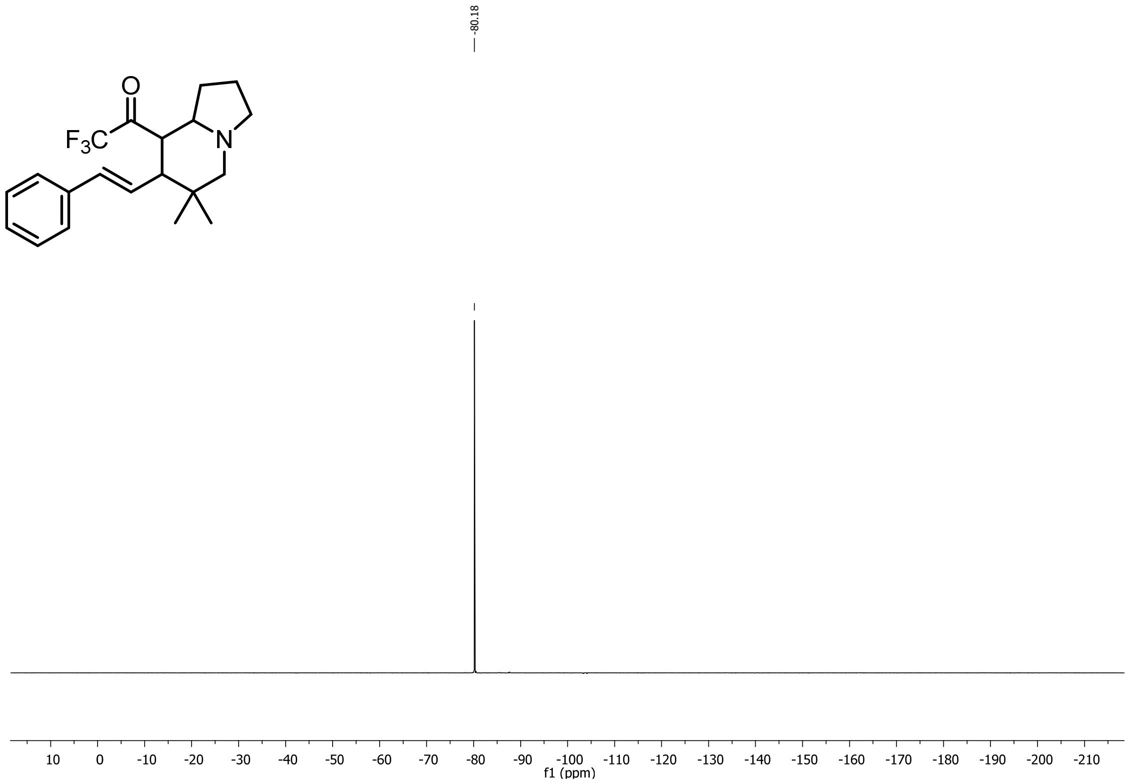

6.1.1.8 7c – 1-(6-ethyl-7-phenyloctahydroindolizin-8-yl)-2,2,2-trifluoroethan-1-one – ^{1}H NMR (600 MHz, $CDCl_3$)

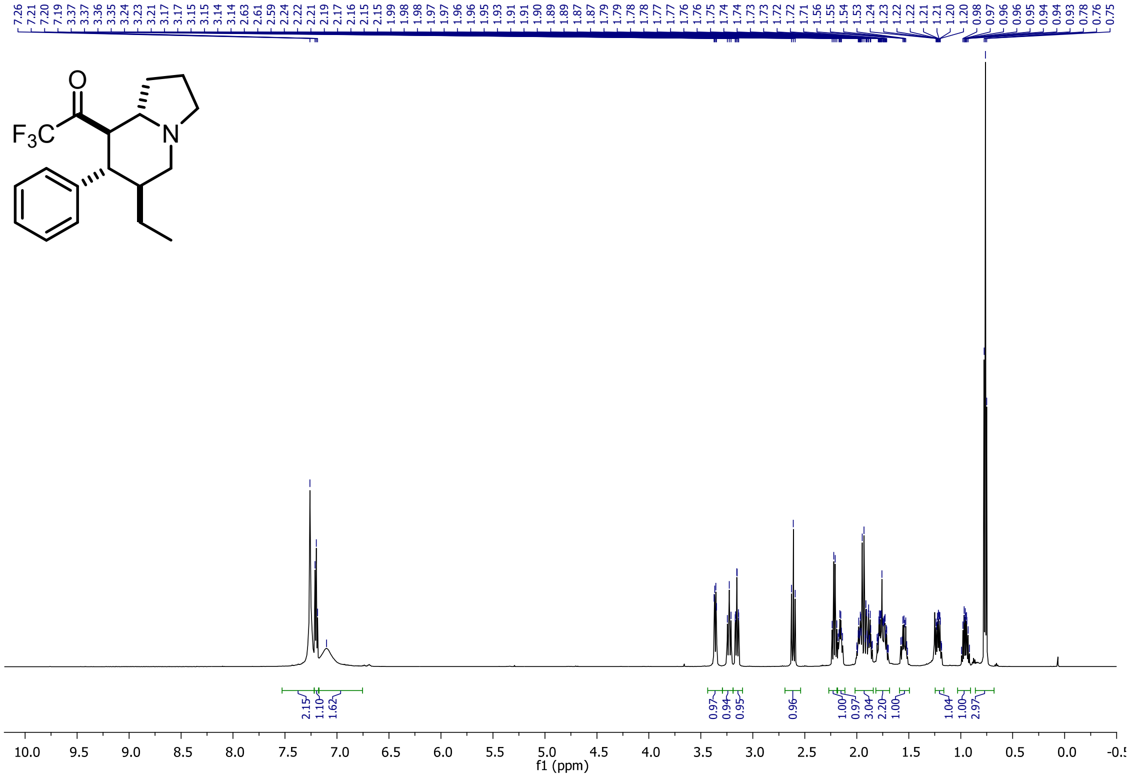

7c – 1-(6-ethyl-7-phenyloctahydroindolizin-8-yl)-2,2,2-trifluoroethan-1-one – ^{13}C NMR (151 MHz, $CDCl_3$)

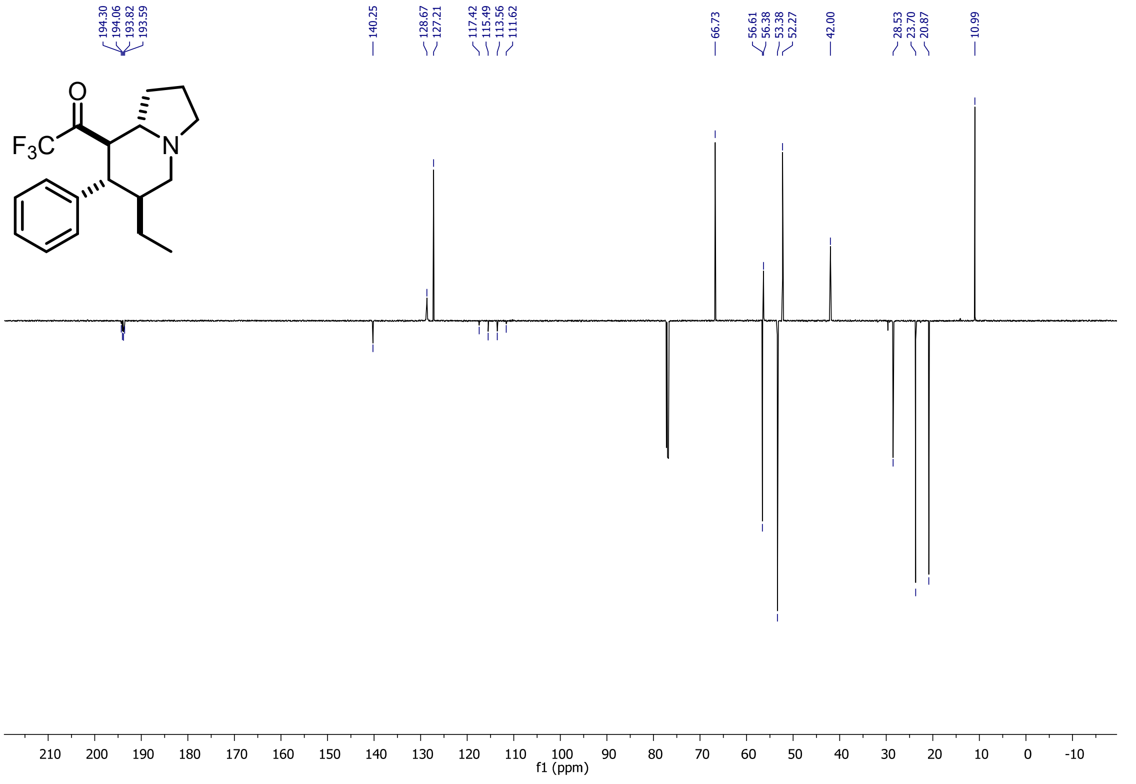

7c – 1-(6-ethyl-7-phenyloctahydroindolizin-8-yl)-2,2,2-trifluoroethan-1-one – ^{19}F NMR (565 MHz, $CDCl_3$)

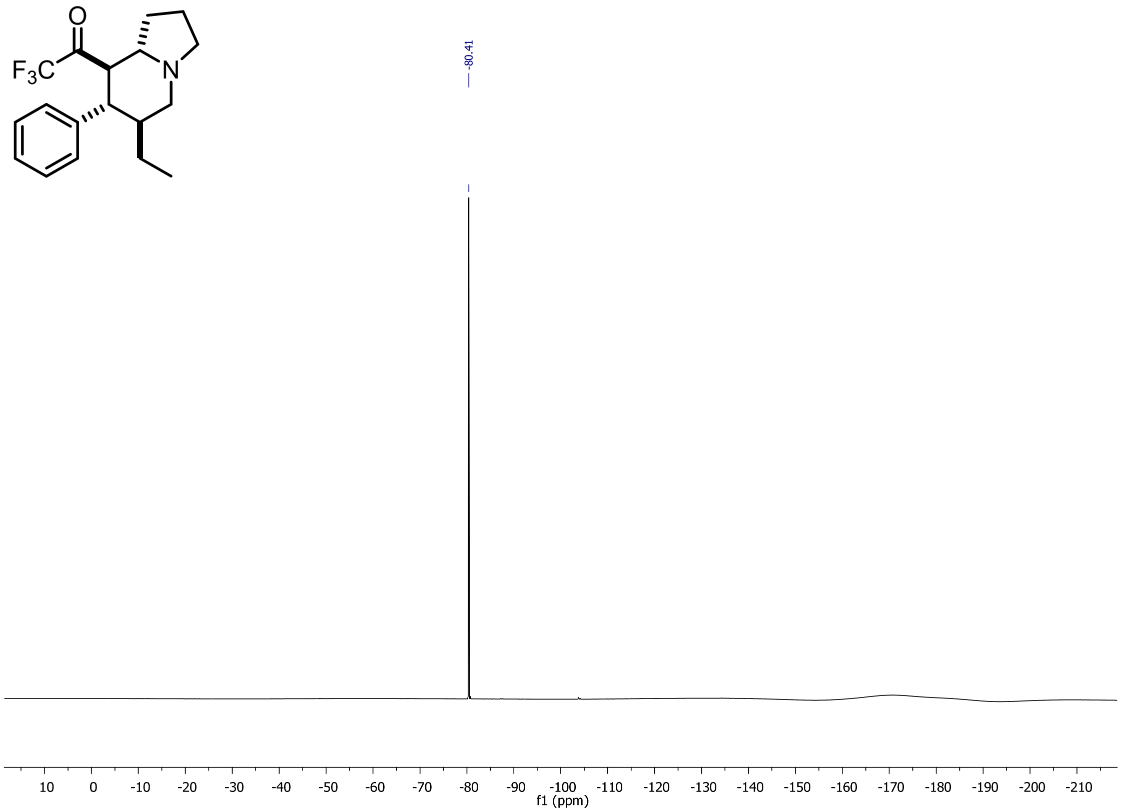

6.1.1.9 7d – 1-(3-ethyl-2-phenyloctahydro-2H-quinolizin-1-yl)-2,2,2-trifluoroethan-1-one – ^{1}H NMR (500 MHz, $CDCl_3$)

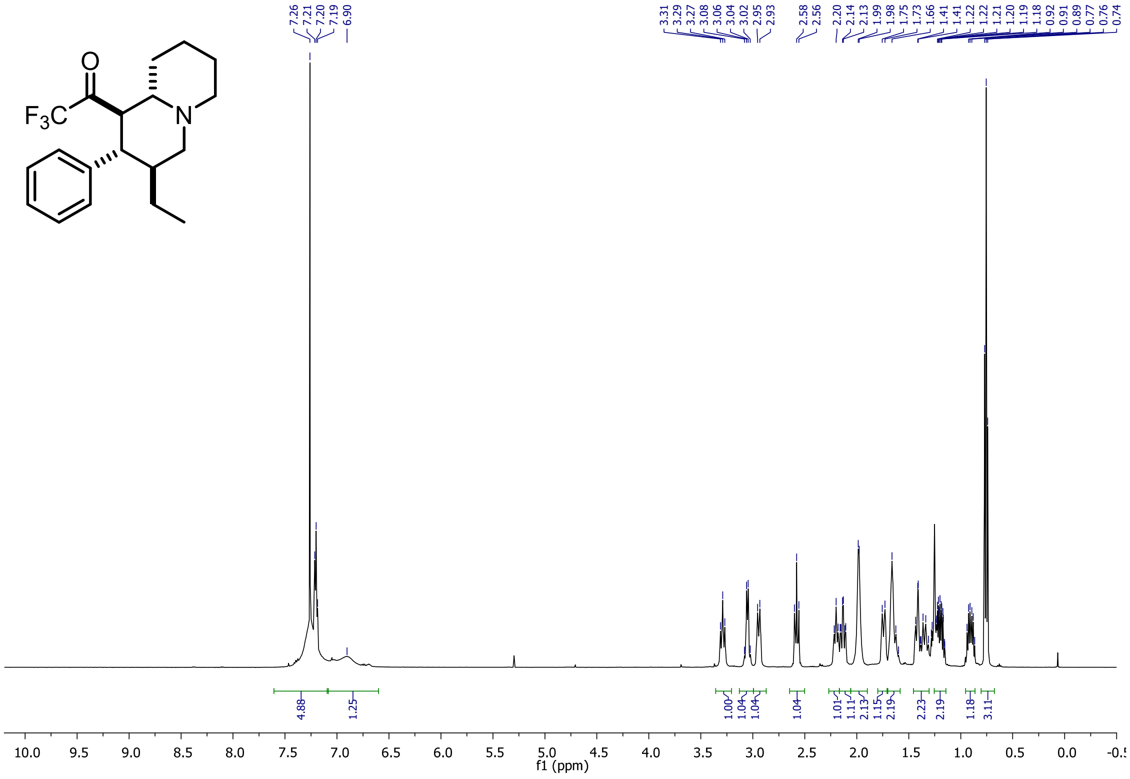

7d – 1-(3-ethyl-2-phenyloctahydro-2H-quinolizin-1-yl)-2,2,2-trifluoroethan-1-one – ^{13}C NMR (126 MHz, $CDCl_3$)

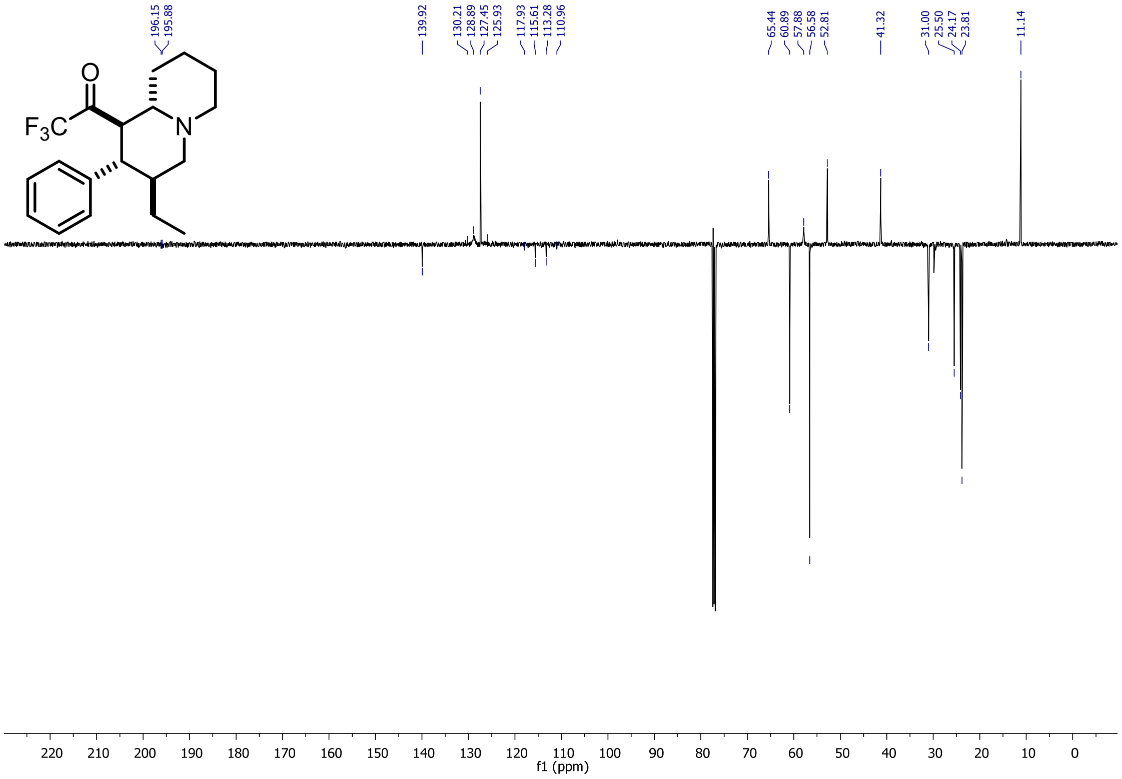

7d – 1-(3-ethyl-2-phenyloctahydro-2H-quinolizin-1-yl)-2,2,2-trifluoroethan-1-one – ^{19}F NMR (565 MHz, $CDCl_3$)

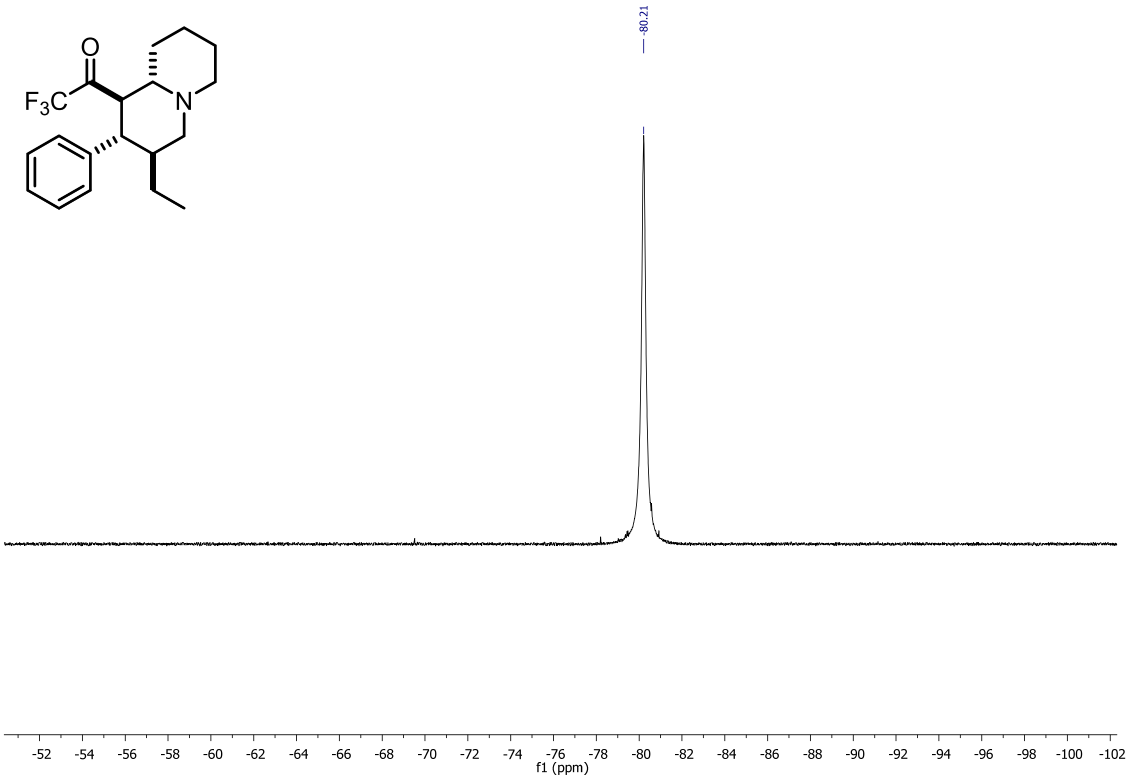

6.1.1.10 S3 – 6,6-dimethyl-7-phenyloctahydroindolizin-8-amine – ^{1}H NMR (400 MHz, CDCl$_3$)

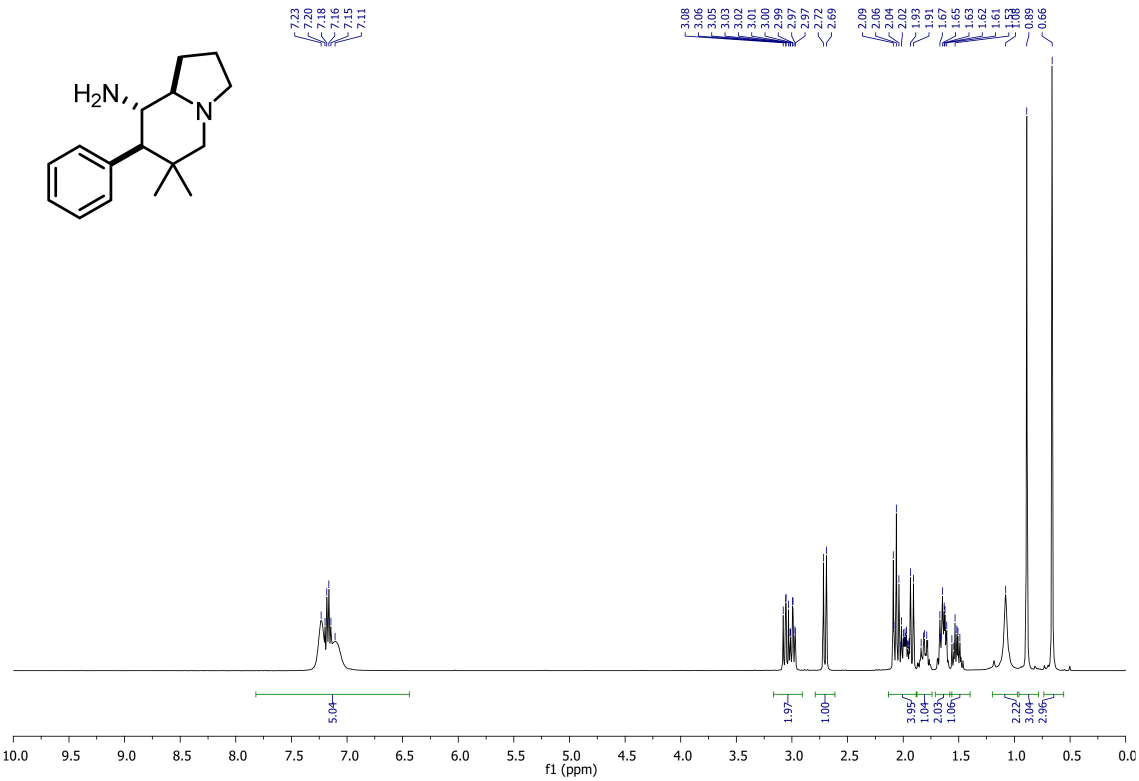

S3 – 6,6-dimethyl-7-phenyloctahydroindolizin-8-amine – ^{13}C NMR (101 MHz, CDCl$_3$)

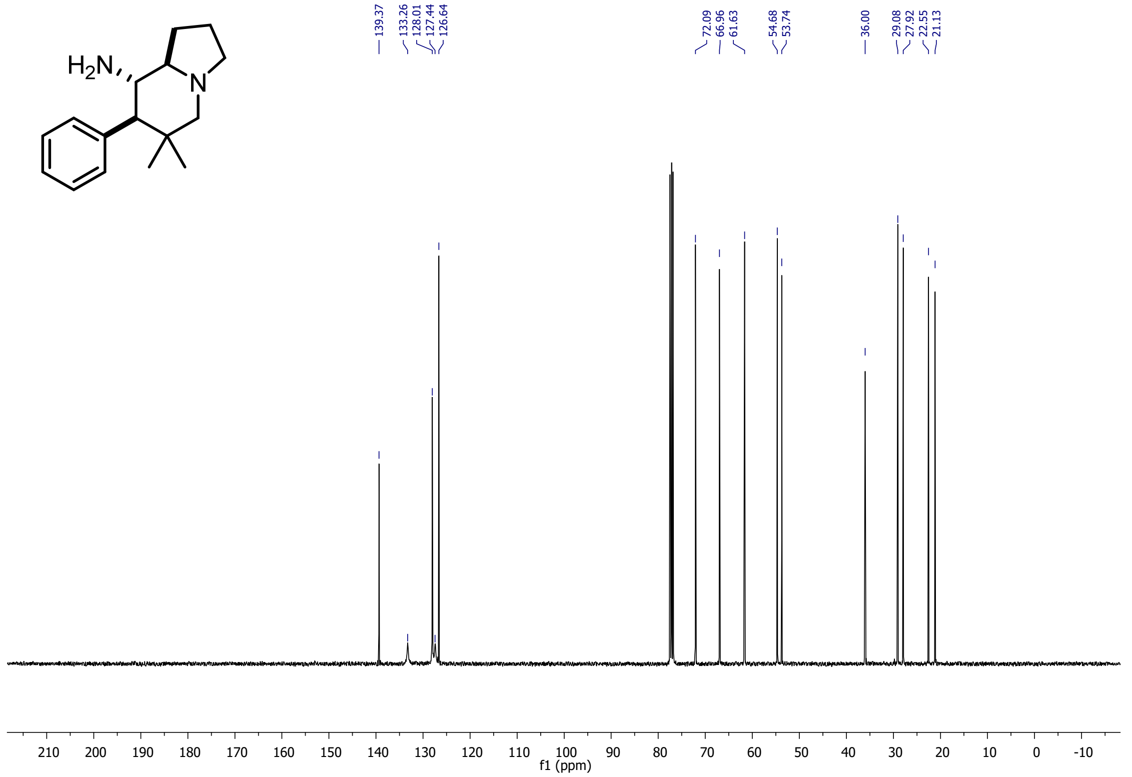

6.1.1.11 9 – 2,2-dimethylhexahydro-1H-pyrrolizin-1-yl)(phenyl)methanol– ^{1}H NMR (400 MHz, $CDCl_3$)

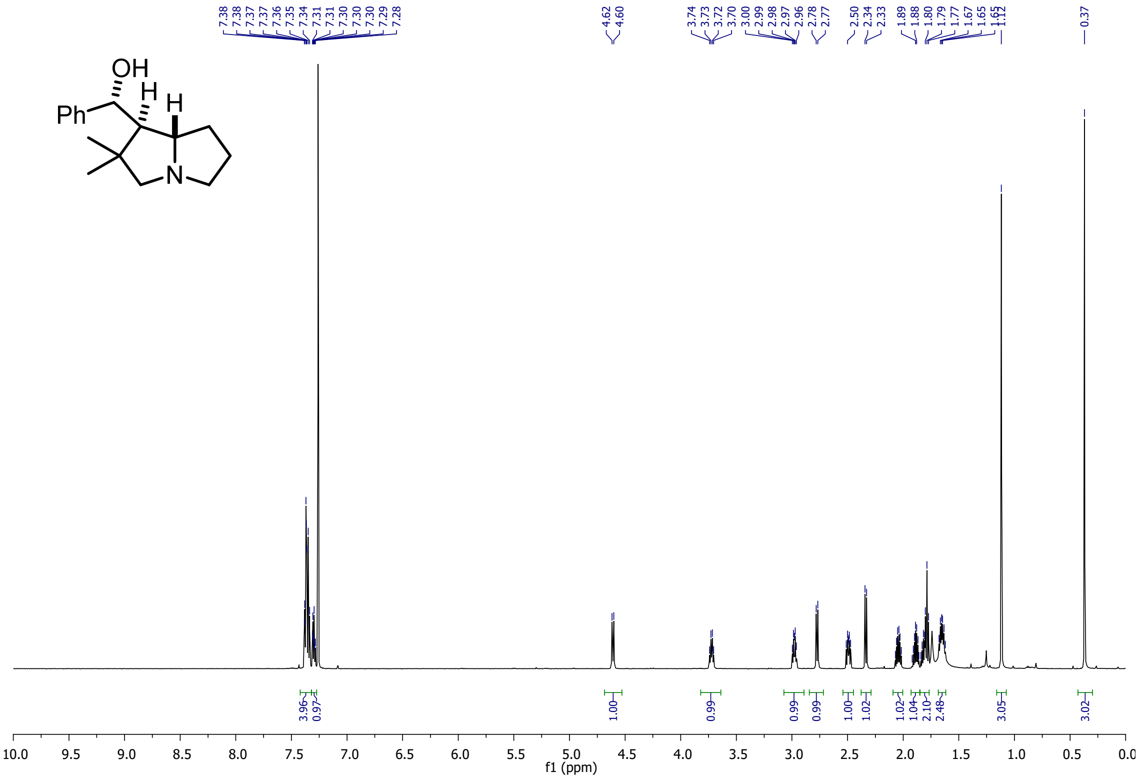

9 – 2,2-dimethylhexahydro-1H-pyrrolizin-1-yl)(phenyl)methanol– ^{13}C NMR (151 MHz, $CDCl_3$)

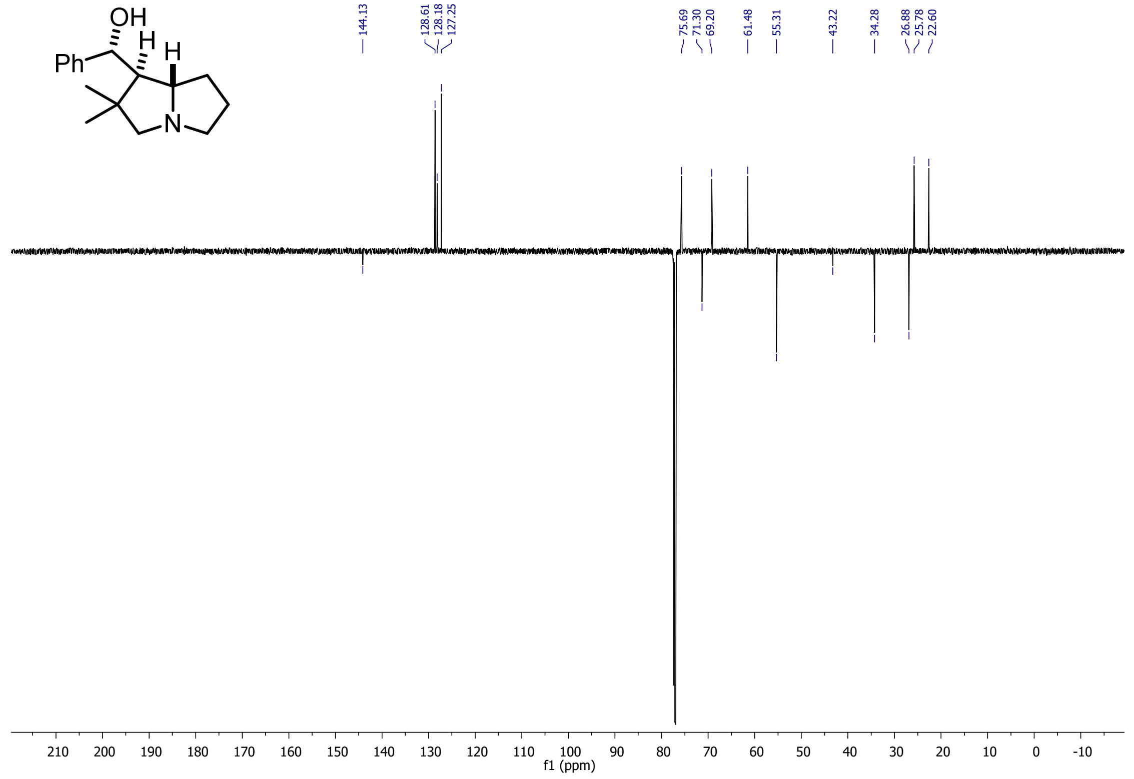

6.1.1.12 10 – 6,6-dimethyl-5-(2-methylallyl)-8-nitro-7-phenyloctahydroindolizine – ^{1}H NMR (600 MHz, $CDCl_3$)

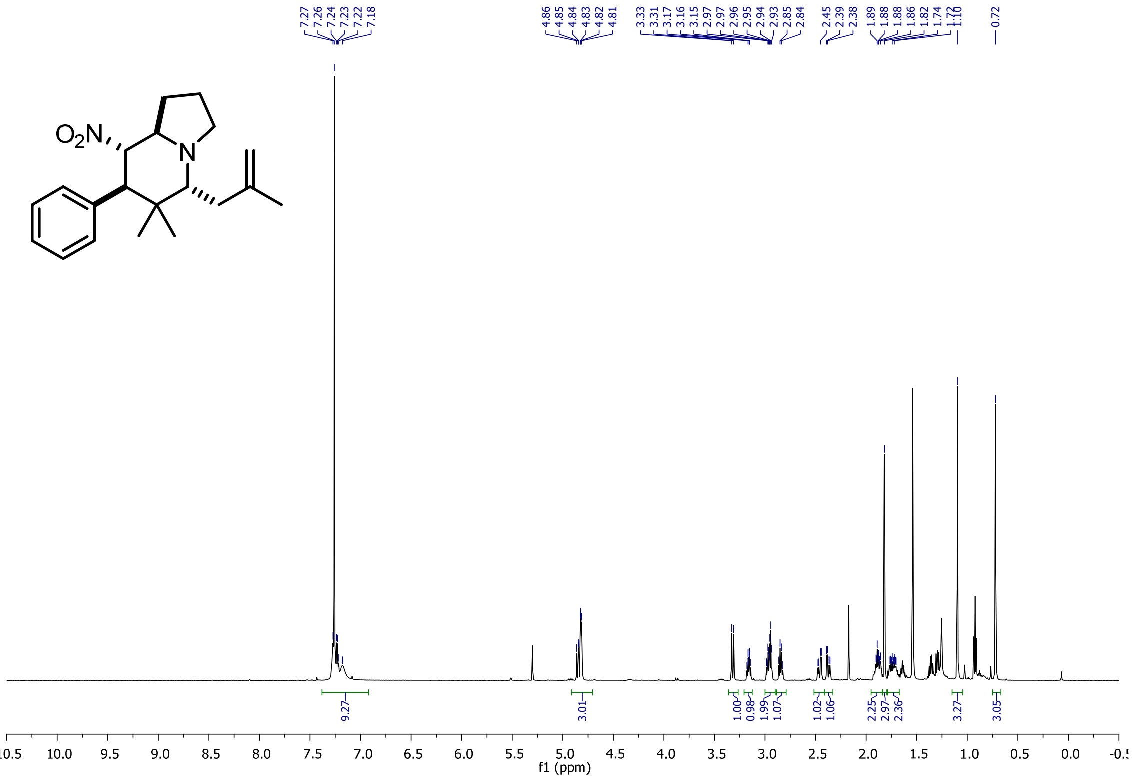

10 – 6,6-dimethyl-5-(2-methylallyl)-8-nitro-7-phenyloctahydroindolizine – ^{13}C NMR (151 MHz, $CDCl_3$)

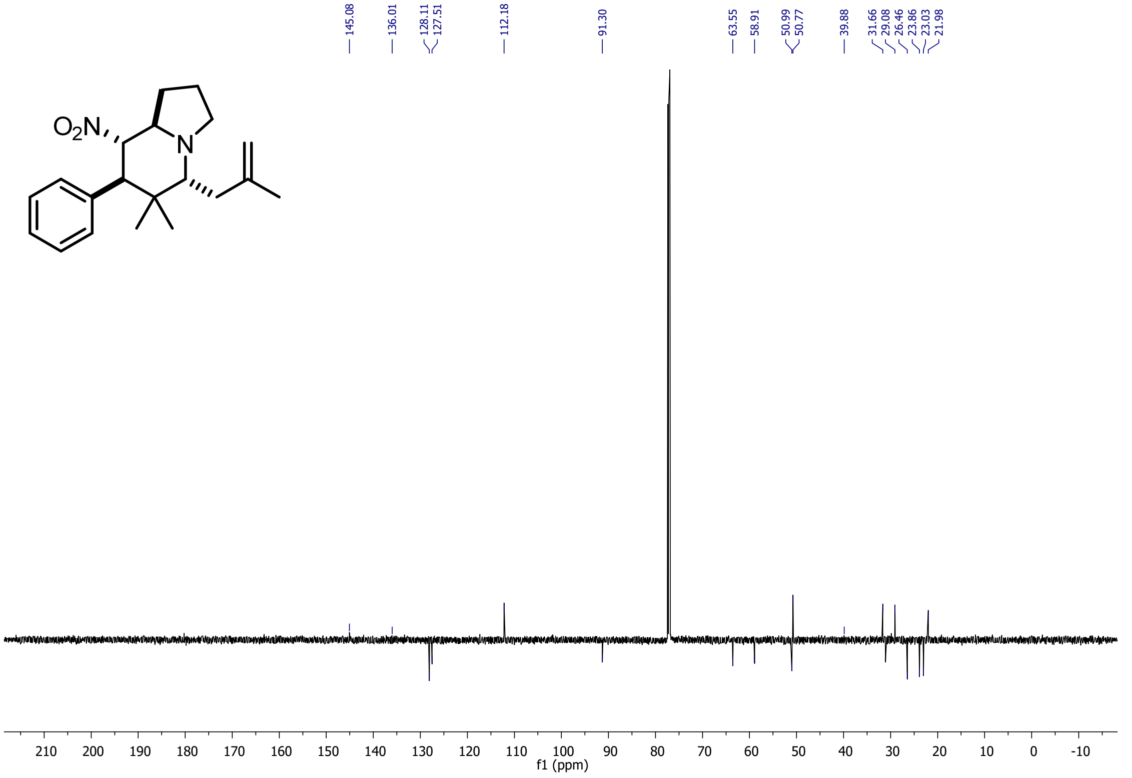

6.1.1.13 11 – 6,6-dimethyl-8-nitro-7-phenyloctahydroindolizine-5-carbonitrile – ^{1}H (600 MHz, $CDCl_3$)

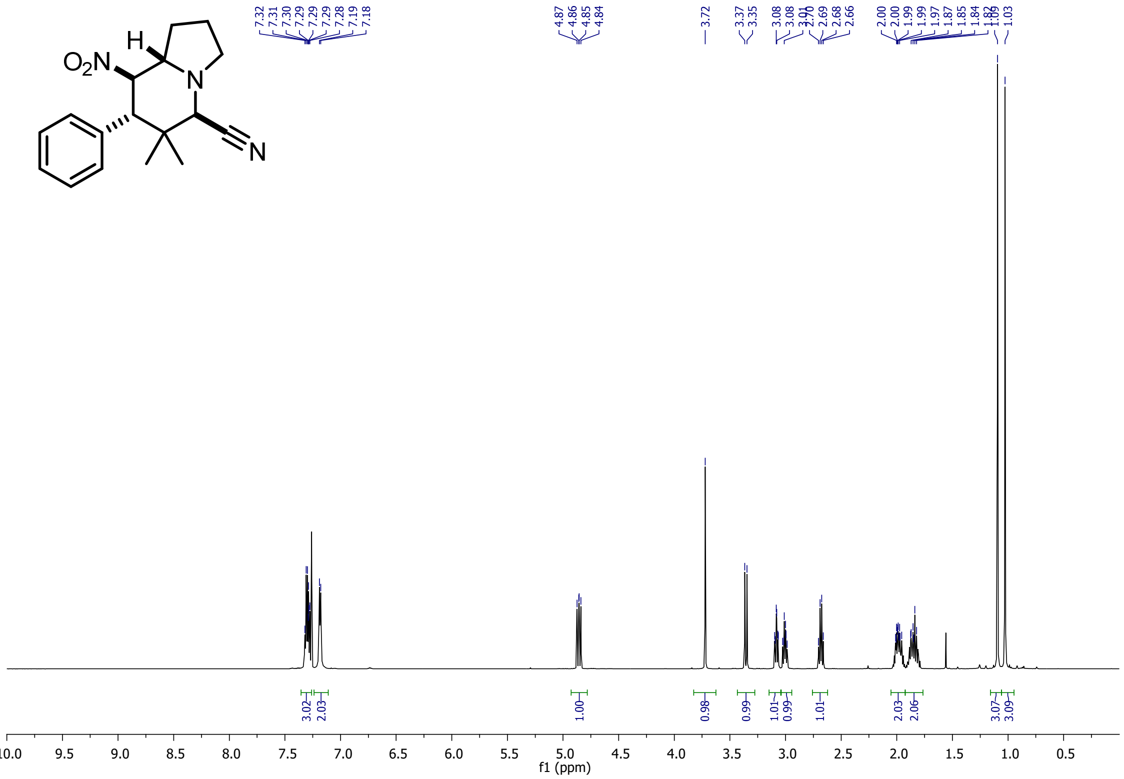

11 – 6,6-dimethyl-8-nitro-7-phenyloctahydroindolizine-5-carbonitrile – ^{13}C (151 MHz, $CDCl_3$)

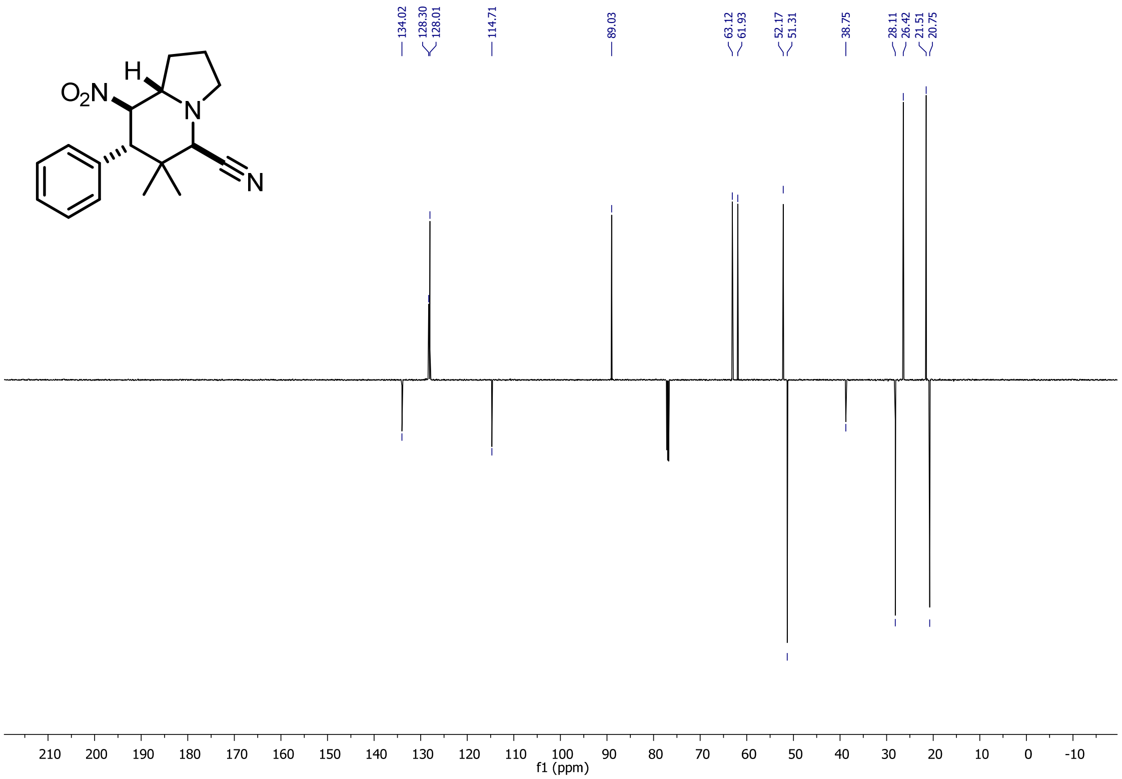

6.1.1.14 12 – N-3,3-dimethyl-2-phenyloctahydro-2H-quinolizin-1-yl)acetamide – ^{1}H NMR (600 MHz, $CDCl_3$)

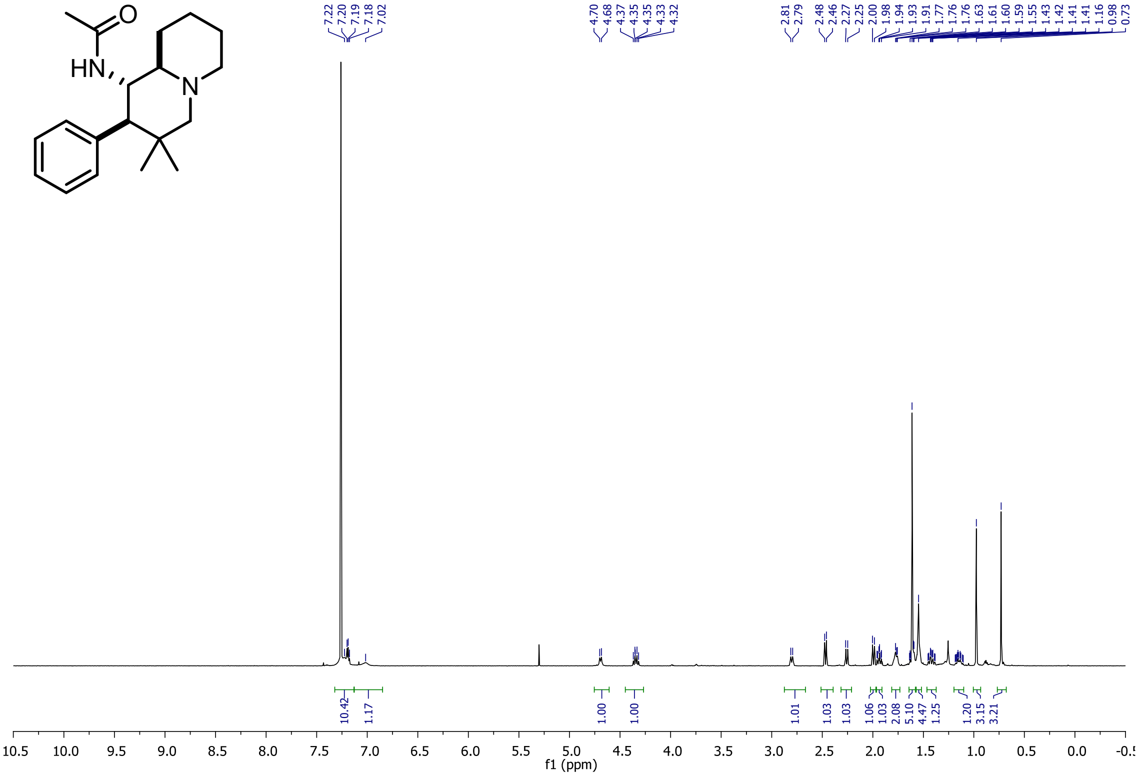

12 – N-3,3-dimethyl-2-phenyloctahydro-2H-quinolizin-1-yl)acetamide – ^{13}C NMR (151 MHz, $CDCl_3$)

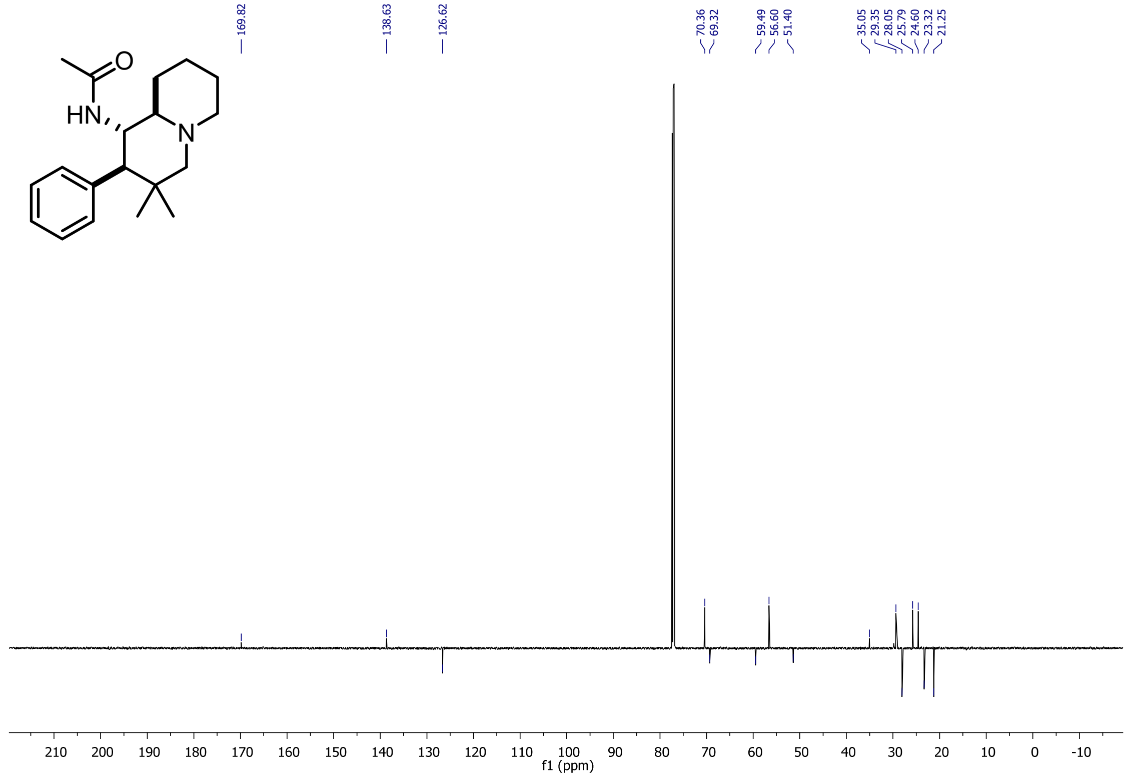

6.1.1.15 13– 1-(3-ethyl-2-phenyloctahydro-2H-quinolizin-1-yl)-2,2,2-trifluoroethan-1-ol – ^{1}H NMR (700 MHz, $CDCl_3$)

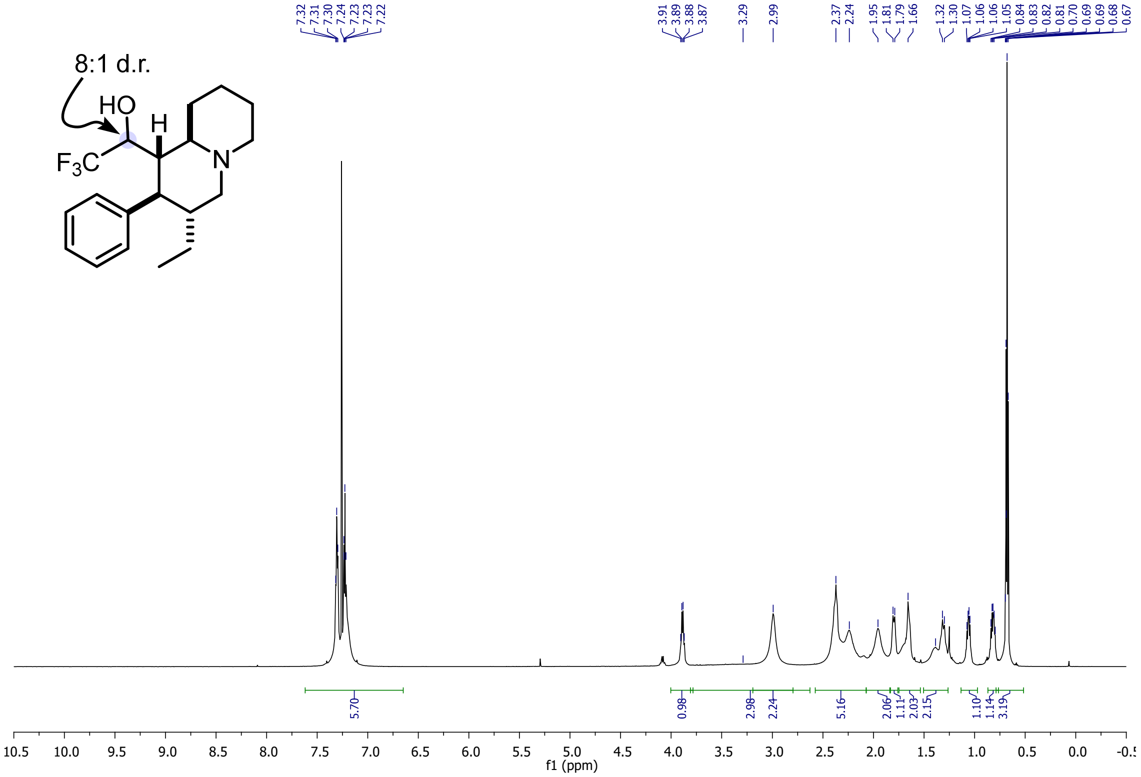

13– 1-(3-ethyl-2-phenyloctahydro-2H-quinolizin-1-yl)-2,2,2-trifluoroethan-1-ol – ^{13}C NMR (101 MHz, $CDCl_3$)

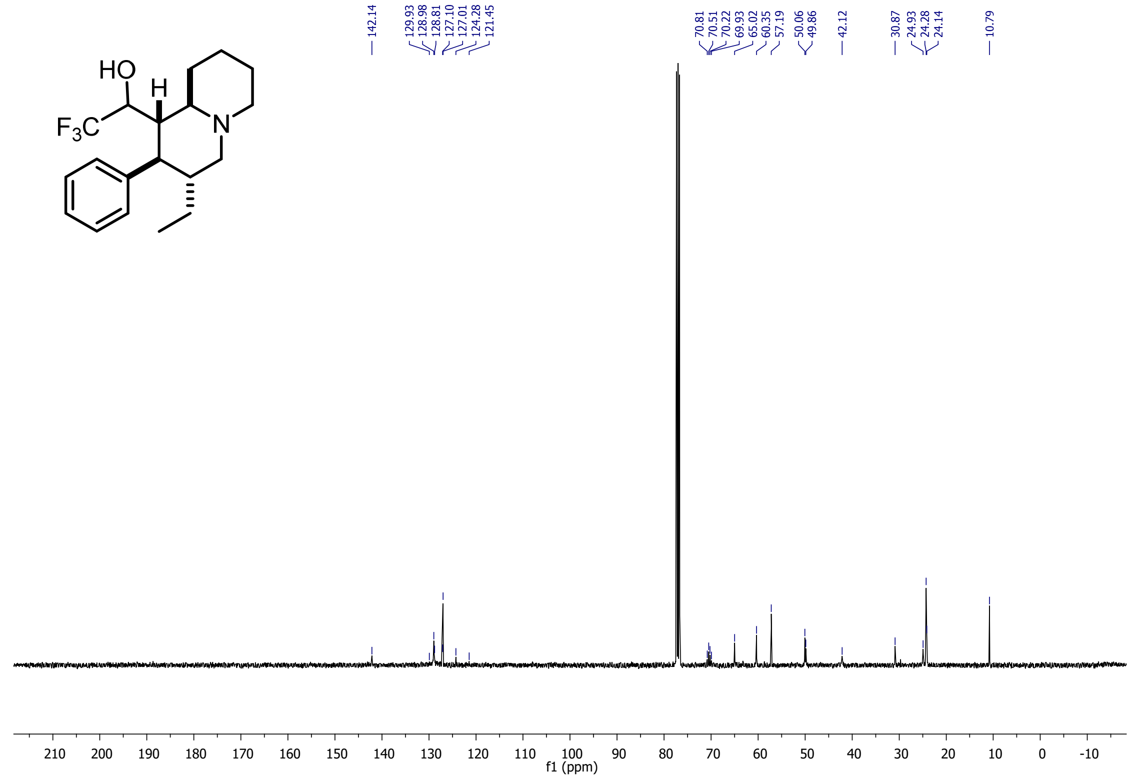

13 – 1-(3-ethyl-2-phenyloctahydro-2H-quinolizin-1-yl)-2,2,2-trifluoroethan-1-ol – ^{19}F NMR (565 MHz, CDCl$_3$)

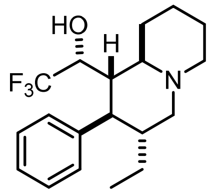

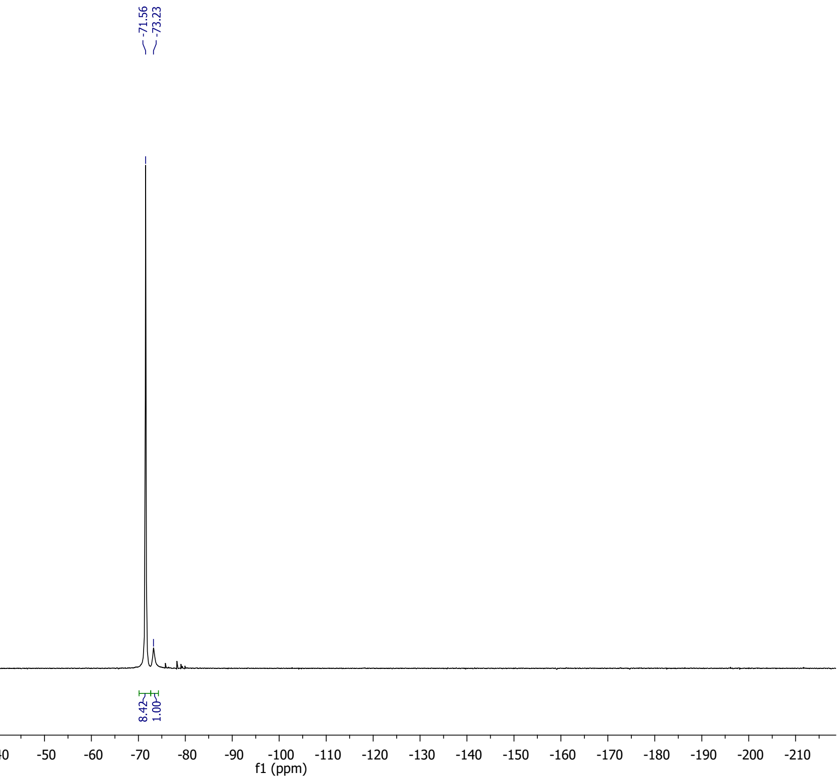

6.1.1.16 14 – 1-(6,6-dimethyl-8-nitro-7-phenyloctahydroindolizin-5-yl)propan-2-one – ^{1}H NMR (600 MHz, $CDCl_3$)

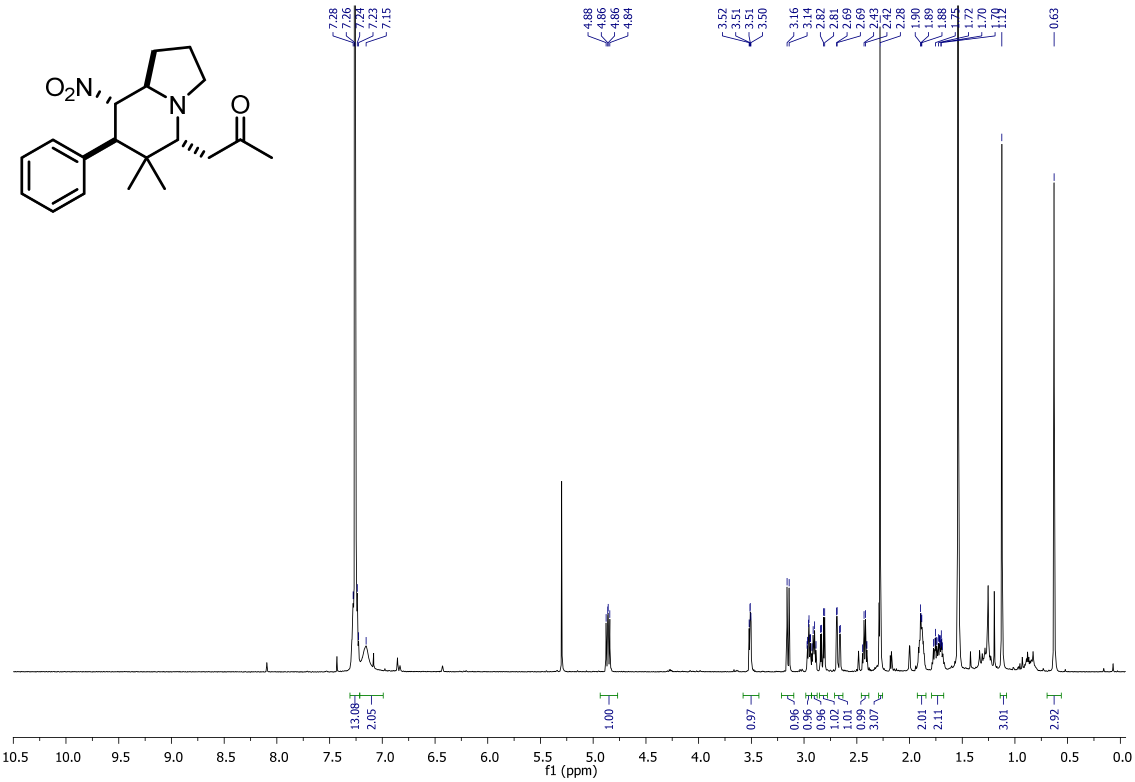

14 – 1-(6,6-dimethyl-8-nitro-7-phenyloctahydroindolizin-5-yl)propan-2-one – ^{13}C NMR (101 MHz, $CDCl_3$)

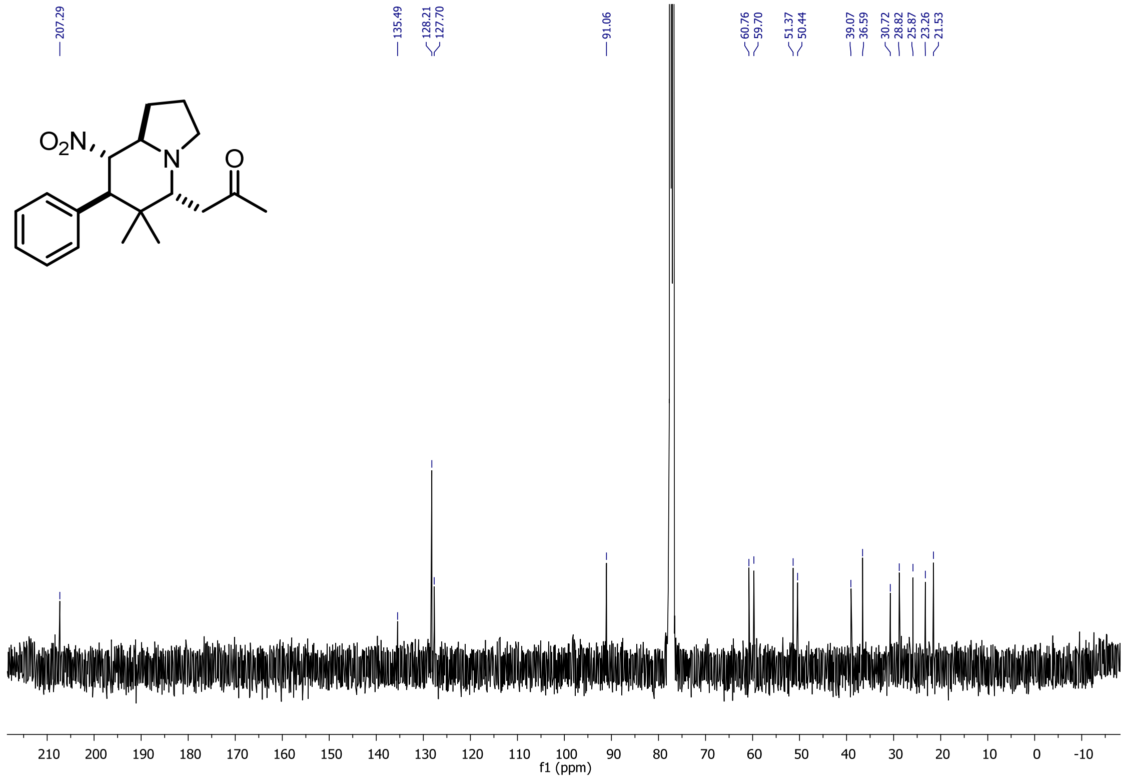

7 X-ray Analysis

The X-ray intensity data were measured on Bruker D8 Venture and Bruker X8 diffractometer equipped with multilayer monochromator, Mo K/α INCOATEC micro focus sealed tube, Kryoflex and Oxford cooling systems. The structures were solved by *Patterson Method and Charge Flipping*. Non-hydrogen atoms were refined with *anisotropic displacement parameters*. Hydrogen atoms were inserted at calculated positions and refined with riding model. The following software was used: *Bruker SAINT software package*[i] using a narrow-frame algorithm for frame integration, *SADABS*[ii] for absorption correction, *OLEX2*[iii] for structure solution, refinement, molecular diagrams and graphical user-interface, *Shelxle*[iv] for refinement and graphical user-interface *SHELXS-2015*[v] for structure solution, *SHELXL-2015*[vi] for refinement, *Platon*[vii] for symmetry check. Experimental data and CCDC-Codes Experimental data (Available online: http://www.ccdc.cam.ac.uk/conts/retrieving.html) can be found in Table S4. Crystal data, data collection parameters, and structure refinement details are given in Tables S5 to S8. Asymmetric Unit visualised in Figures S5 and S7.

Table S4 Experimental parameter and CCDC-Code.

Sample	Machine	Source	Temp.	Detector Distance	Time/ Frame	#Frames	Frame width	CCDC
	Bruker		[K]	[mm]	[s]		[°]	
**DaKl284**	X8	Mo	130	40	50	500	0.500	1973786
**Klose_K2141**	D8	Mo	100	30	20	2672	0.360	2079162

6,6-dimethyl-8-nitro-7-phenyloctahydroindolizine (CCDC – 1973786)

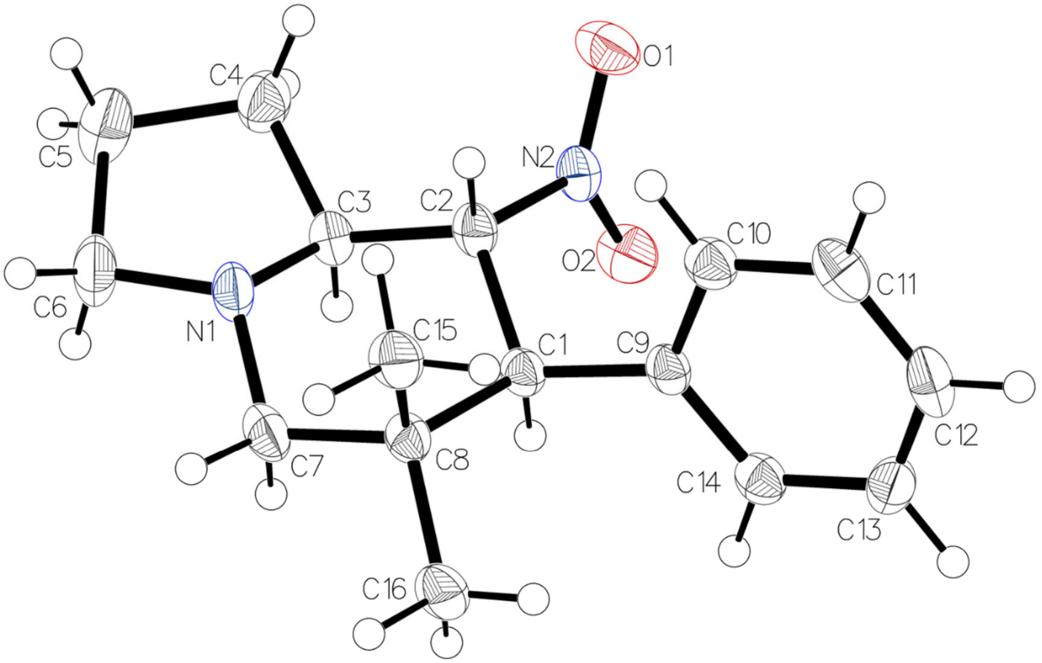

Figure S5 Asymmetric Unit drawn with 50% displacement ellipsoid. The bond precision for C-C single bonds is 0.0027Å.

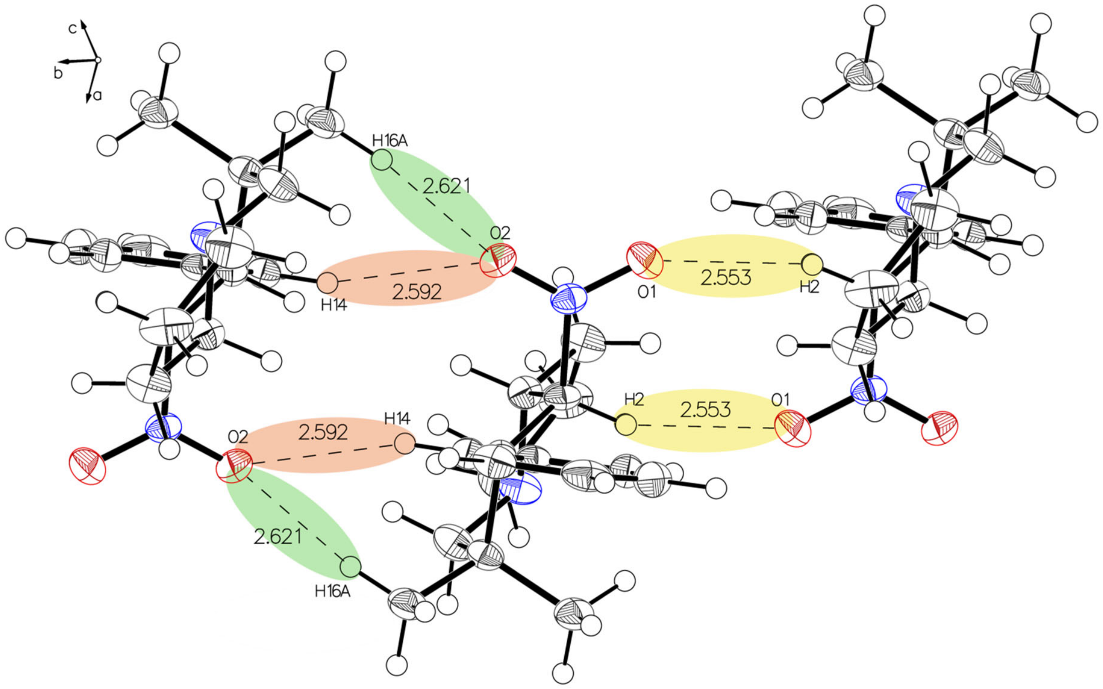

Figure S6 Visualisation of weak interactions along axes b. Three different could be detected.

Table S5 Sample and crystal data.

Radiation [Å]	MoKα (λ = 0.71073)	Z	8	Measurement method	\f and \w scans
Crystal habit	clear colourless needle	a [Å]	16.1806(16)		
Crystal size [mm^3]	0.225 × 0.05 × 0.03	b [Å]	10.8069(11)	Abs. correction type	multiscan
Empirical formula	C16H22N2O2	c [Å]	16.7368(14)	Abs. correction Tmin	0.4297
Formula weight [g/mol]	274.35	α [°]	90	Abs. correction Tmax	0.7460
Temperature [K]	130.0	β [°]	90	Density (calculated) [g/cm3]	1.245
Crystal system	Orthorhombic	γ [°]	90	Absorption coefficient [mm^{-1}]	0.083
Space group	Pbca	Volume [Å^3]	2926.6(5)	F (000) [e$^-$]	1184.0

Table S6 Data collection and structure refinement.

2Θ range for data collection [°]	4.868 to 60.074	Index ranges		Goodness-of-fit on F^2	1.026
Reflections collected	15352	h	-20 ≤ h ≤ 22	Diff. peak and hole [e$^-$Å^{-3}]	0.29/-0.23
Data / restraints / parameters	4193/0/183	k	-13 ≤ k ≤ 15		
Refinement method	Charge Flipping	l	-23 ≤ l ≤ 18	Function minimised	Σ w (F_o^2 - F_c^2)^2
		all data	R1 = 0.1195, wR2 = 0.1582	Weighting scheme	where
		I>2σ(I)	R1 = 0.0594, wR2 = 0.1332	w=1/[σ^2(Fo^2) + (0.0425P)^2 + 0.6152P]	P=(F_o^2+2F_c^2)/3

(±)-(*R*)-((1*R*,7a*S*)-2,2-dimethylhexahydro-1H-pyrrolizin-1-yl)(phenyl)methanol (CCDC – 2079162)

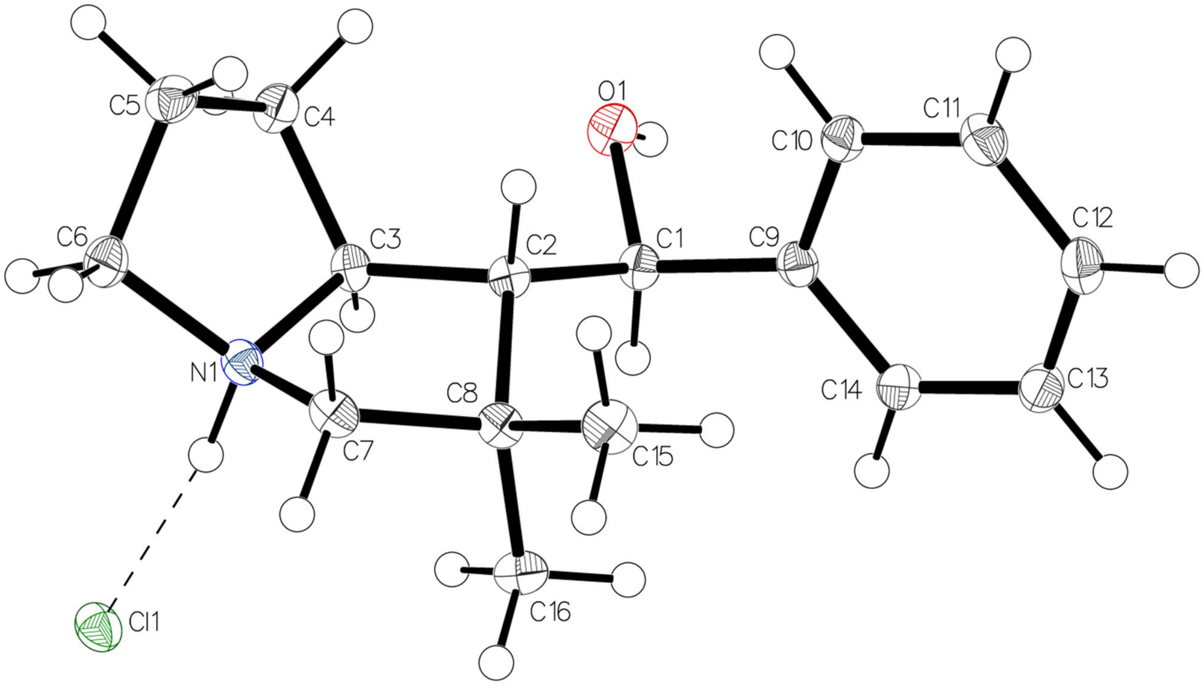

Figure S7 Asymmetric Unit drawn with 50% displacement ellipsoid. The bond precision for C-C single bonds is 0.0012Å.

Table S7 Sample and crystal data.

Radiation [Å]	MoKα (λ = 0.71073)	Z	4	Measurement method	\f and \w scans
Crystal habit	clear colourless block	a [Å]	15.1296(5)		
Crystal size [mm³]	0.302 × 0.139 × 0.134	b [Å]	9.6664(3)	Abs. correction type	multiscan
Empirical formula	C16H24ClNO	c [Å]	10.8136(3)	Abs. correction Tmin	0.5445
Formula weight [g/mol]	1.229	α [°]	90	Abs. correction Tmax	0.5642
Temperature [K]	100.0	β [°]	105.6873(14)	Density (calculated) [g/cm3]	1.229
Crystal system	Monoclinic	γ [°]	90	Absorption coefficient [mm^{-1}]	0.244
Space group	P21/c	Volume [$Å^3$]	1522.56(9)	F (000) [e^-]	608.0

Table S8 Data collection and structure refinement.

2Θ range for data collection [°]	5.058 to 60.102	Index ranges		Goodness-of-fit on F^2	1.071
Reflections collected	62412	h	$-21 \leq h \leq 21$	Diff. peak and hole [$e^{-}Å^{-3}$]	0.35/-0.23
Data / restraints / parameters	4468/0/179	k	$-13 \leq k \leq 13$		
Refinement method	Patterson Method	l	$-14 \leq l \leq 15$	Function minimised	$\Sigma\ w\ (F_o^2 - F_c^2)^2$
		all data	R1 = 0.0326, wR2 = 0.0871	Weighting scheme	where
		I>2σ(I)	R1 = 0.0300, wR2 = 0.0856	$w=1/[\sigma^2(Fo^2) + (0.0499P)^2 + 0.3732P]$	$P=(F_o^2+2F_c^2)/3$

[i] Bruker SAINT v8.38B Copyright © 2005-2019 Bruker AXS

[ii] Sheldrick, G. M. (1996). *SADABS.* University of Göttingen, Germany.

[iii] Dolomanov, O.V., Bourhis, L.J., Gildea, R.J, Howard, J.A.K. & Puschmann, H. , OLEX2, (2009), J. Appl. Cryst. 42, 339-341

[iv] C. B. Huebschle, G. M. Sheldrick and B. Dittrich, ShelXle: a Qt graphical user interface for SHELXL, J. Appl. Cryst., 44, (2011) 1281-1284

[v] Sheldrick, G. M. (2015). *SHELXS v 2016/4* University of Göttingen, Germany.

[vi] Sheldrick, G. M. (2015). *SHELXL v 2016/4* University of Göttingen, Germany.

[vii] A. L. Spek, Acta Cryst. 2009, D65, 148-155
